# Asymmetric Carbonium Ion Catalysis: The Intramolecular Hydroalkoxylation of Cyclopropanes

**DOI:** 10.1021/jacs.5c20876

**Published:** 2026-01-05

**Authors:** Fuxing Shi, Markus Leutzsch, Nils Frank, Chendan Zhu, Nobuya Tsuji, Guanwei Zhang, Benjamin List

**Affiliations:** † 28314 Max-Planck-Institut für Kohlenforschung, Kaiser-Wilhelm-Platz 1, 45470, Mülheim an der Ruhr, Germany; ‡ Institute for Chemical Reaction Design and Discovery (WPI-ICReDD), 12810 Hokkaido University, Sapporo 001-0021, Japan

## Abstract

Cyclopropanes serve as valuable synthetic intermediates in drug discovery and natural product synthesis. However, the stereoselective functionalization of cyclopropanes remains a fundamental challenge, traditionally necessitating the prior activation of the parent hydrocarbon. Here, we report an organocatalytic asymmetric intramolecular hydroalkoxylation of cyclopropanes that overcomes this constraint using chiral imidodiphosphorimidate catalysts. Our strategy directly affords enantioenriched substituted tetrahydrofurans with high enantioselectivity (up to 96.5:3.5 er). Kinetic analyses reveal a zero-order dependence on the substrate and support catalyst saturation. Combined experimental and computational studies suggest that the prereaction catalyst–substrate ion pair is stabilized through noncovalent interactions leading to the formation of a transient cycloproponium ion-like transition state. This work provides a potentially general platform for stereoselective cyclopropane functionalization.

## Graphical abstract

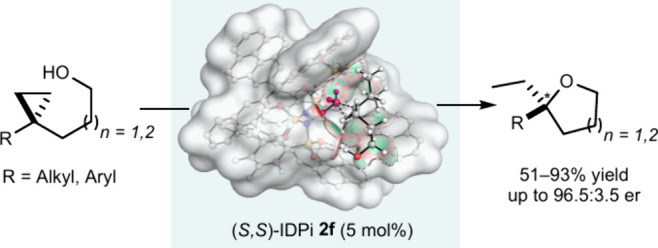

Since Staudinger and Ružicka discovered (+)-*trans*-chrysanthemic acid—the first natural product containing a cyclopropane ring—in 1924,^1^ cyclopropanes have served as pivotal reactive intermediates in chemical synthesis.^2–11^ The high ring strain of cyclopropanes (∼27.5 kcal mol^–1^)^12,13^ enables unusual cycloalkane reactivity, which has been exploited in various transformations. In particular, donor–acceptor activated cyclopropanes readily undergo stereoselective transformations including cycloadditions,^14–18^ 1,3-difunctionalizations,^19–21^ and nucleophilic additions with metal catalysts.^22–25^ In contrast, unfunctionalized cyclopropanes represent a significant challenge to selective catalysis and have been much less explored, as the initial C–C bond cleavage is associated with a substantial barrier.^26,27^ Nonetheless, protonation with Brønsted acids can furnish fascinating nonclassical carbocations, including the historically proposed corner- and edge-protonated cycloproponium ions, as well as methyl-bridged species that arise via subsequent rearrangements (Figure 1A).^28–31^ These debated carbonium ions are intrinsically unstable and prone to uncontrolled side reactions,^32–35^ and their enantioselective control is challenged by the absence of electronic or stereochemical bias.^36–38^

**Figure 1:**
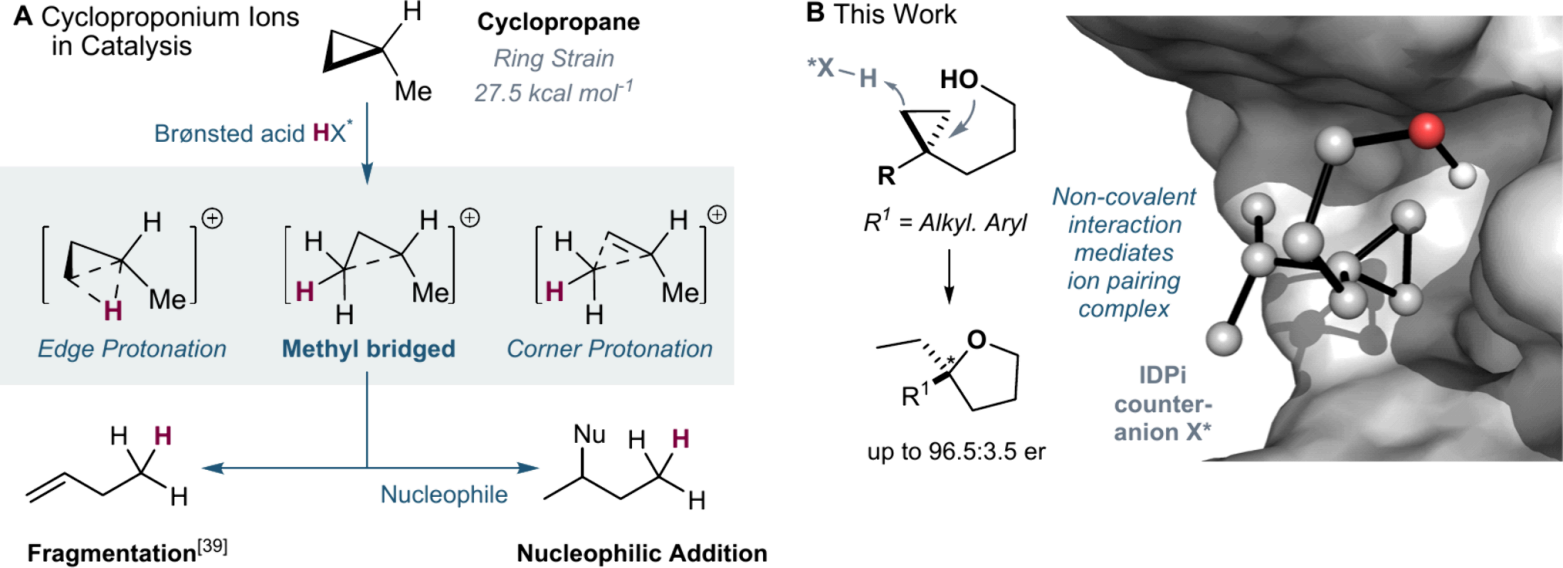
(**A**) Cycloproponium ion catalysis. (**B**) This work: intramolecular hydroalkoxylation of cyclopropanes enabled by IDPi.

Imidodiphosphorimidates (IDPis) have emerged as versatile organocatalysts, combining high Brønsted acidity with enzyme-like, confined active pockets that enable the activation and preorganization even of unfunctionalized substrates.^39^ As a result, IDPis can exert high levels of enantiocontrol in transformations that typically require superacidic conditions, such as olefin activation and Wagner–Meerwein rearrangements.^40–42^ Our research group recently developed the first asymmetric catalytic fragmentation of purely aliphatic cyclopropanes via cycloproponium ion intermediates using IDPi catalysis.^43^ Building on this, a further challenge lies in harnessing nucleophilic additions to cyclopropanes as another mode of cycloproponium ions. We envisioned applying the IDPi catalysis platform to resolve the long-standing challenge of controlling the enantioselectivity of nucleophilic additions to cyclopropanes. We hypothesized that stereochemistry can be established by stabilizing the cyclopropyl alcohol ion within the enzyme-like chiral environment of the IDPi catalyst. Here we report an enantioselective intramolecular hydroalkoxylation of cyclopropanes, yielding enantioenriched substituted tetrahydrofuran products with up to 96.5:3.5 enantiomeric ratio (Figure 1B).

At the onset of our study, we investigated several organic chiral Brønsted acids for the targeted ring-opening of substrate **1k** (Figure 2). As anticipated, the mild, unconfined chiral phosphoric acid (CPA) **2a** and the confined imidodiphosphorimidate (IDP) catalyst **2b** showed no reactivity. In contrast, our highly acidic and confined IDPi catalyst **2c** afforded product **3k** in excellent yield, albeit with near-racemic enantioselectivity. Leveraging the inherent tunability of IDPi catalysts, we conducted a systematic catalyst screening campaign. Specifically, we postulated that substituents at the 3,3′-positions of the 1,1′-bi-2-naphthol (BINOL) scaffold critically define the shape of the catalyst’s chiral microenvironment, potentially aided by dispersion forces. Our further screening identified IDPi catalyst **2d**, bearing naphthyl groups at the BINOL 3,3′-positions designed to engage in π-σ interactions.^44^ Catalyst **2d** demonstrated promising enantioselectivity in the formation of **3k** (78:22 er). Subsequently, to enhance conformational rigidity via potential π-σ interactions, we replaced the trifluoromethanesulfonyl groups at the catalytically active site of IDPi with arylsulfonyl groups. This modification yielded IDPi catalyst **2e**, which readily provided product **3k** with improved enantiocontrol (82:18 er). Further development led to pyrene-substituted IDPi catalyst **2f**, delivering desired product **3k** in very good yield with excellent enantioselectivity (92% yield, 95:5 er).

**Figure 2:**
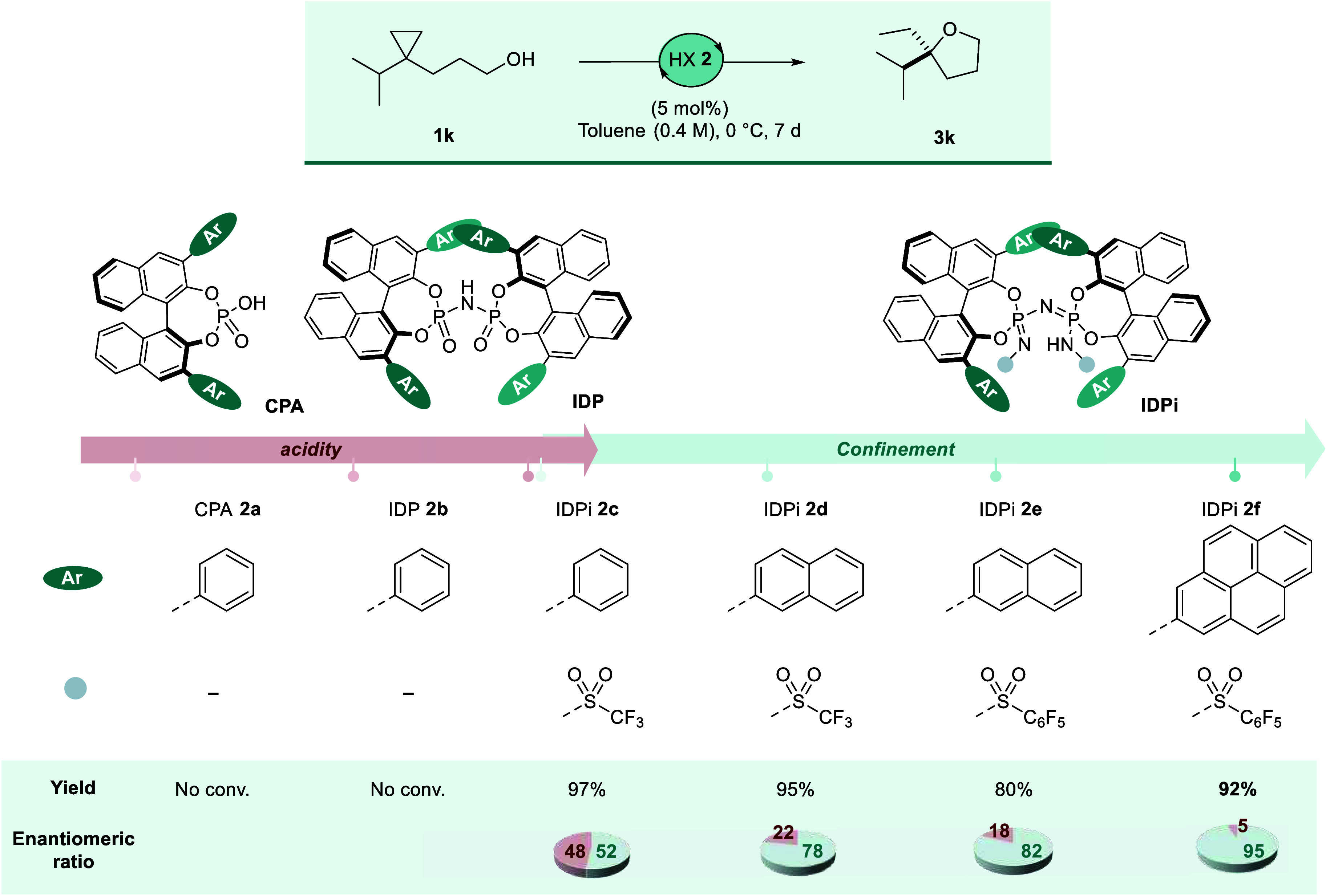
Yields were determined by ^1^H NMR, using CH_2_Br_2_ as an internal standard. Enantiomeric ratio was determined by gas chromatography. Reaction was performed in a 0.4 M solution at 0 °C for 7 days.

With the optimal catalyst and conditions in hand, we started investigating the scope and limitation of this hydroalkoxylation reaction (Figure 3). The investigation began with a series of cycloalkyl-substituted substrates. For cyclopropane bearing seven- to four-membered rings (**3a**–**3d**), THF formation proceeded with excellent reactivity (yields up to 92%) and enantioselectivity (up to 96.5:3.5). In contrast, the transformation leading to six-membered tetrahydropyran **3e** showed a marked decrease in both reactivity and selectivity, requiring elevated temperatures to reach completion. Linear alkyl-substituted substrates (**1f**–**1h**) were also tolerated in the reaction (65–96% yield, 92:8 to 94.5:5.5 er). Even the minimally substituted methyl derivative **1i** afforded product **3i** with commendable enantiocontrol (84:16 er). Branched alkyl-substituted substrates (**1j** and **1k**) proceeded smoothly to give good yields with excellent enantioselectivities (er >95:5), while the bulky *tert*-butyl substrate **1l** suffered from diminished reactivity and enantioselectivity (84:16 er). In contrast, phenyl-substituted substrate **1m** displayed reduced reactivity and required heating to 60 °C to achieve a 78% yield with moderate stereoselectivity (80:20 er). This behavior suggests the inductive electron-withdrawing effect of the aryl group, which lowers the effective basicity of the cyclopropyl group under acidic conditions. Arylalkyl-substituted substrates (**1n** and **1o**) gave slightly diminished enantioselectivities and reactivity compared to their aliphatic counterparts, whereas a notable exception was substrate **1p**, bearing an Indane-2-yl group, which underwent cyclization with excellent enantioselectivity (94:6 er) and 93% yield. The reaction also exhibited a good tolerance toward carbonyl- and heteroarene-containing substrates. Specifically, substrates **1q** and **1r** were smoothly transformed to the corresponding products **3q** and **3r** in excellent yields and good enantioselectivities (**3q**: 87% yield, 88:12 er; **3r**: 98% yield, 85:15 er). 2-Ethyl-2-alkyltetrahydrofurans constitute an important substructure of natural products such as monensin, lasalocid, and other targets.^45,46^ To highlight the synthetic applicability of our method, product **3g** was treated with RuCl_3_/NaIO_4_ to efficiently deliver lactone **4**, an industrial fragrance compound known for its coconut-like aroma.^47^ The absolute configuration was determined by comparing the known **3m** with an enantiomerically enriched authentic sample.^48^ With substrate **1k**, either product **3k** (95:5 er) or (*ent*)**-3k** (94.5:5.5 er) was obtained using the enantioermic catalyst (*S,S*)- or (*R,R*)-IDPi **2f**, respectively.

**Figure 3:**
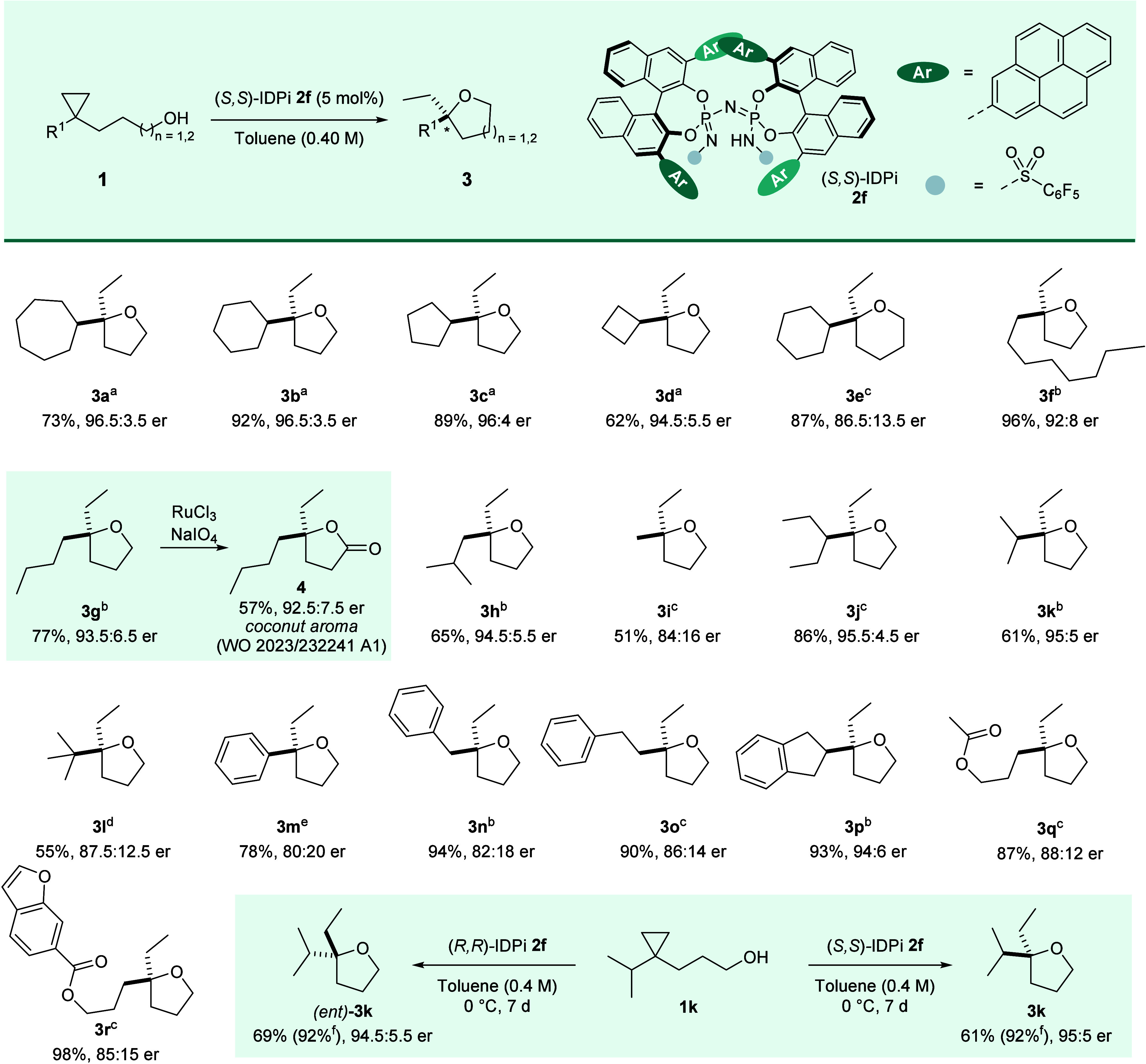
Reactions were performed at 0.20 mmol scale and 0.40 M concentration in toluene at ^a^–10 °C, ^b^0 °C, ^c^25 °C, ^d^40 °C, and ^e^60 °C for 7 days; isolated yields determined after chromatographic purification. ^f^Because of the volatility of substrates **3k** and (*ent*)**-3k**, the yields were also determined by ^1^H NMR using CH_2_Br_2_ as an internal standard. The er was determined by high-performance liquid or gas chromatography analysis.

Mechanistically, four reasonable pathways are envisioned (Figure 4B): a stepwise mechanism involving protonation of **1k** to form a tertiary carbocation intermediate (**INT-A**) or a cycloproponium ion (**INT-B**) followed by intramolecular hydroxyl attack; a covalent mechanism involving the formation of substrate-catalyst adduct (**INT-C**); or a concerted pathway mediated by noncovalent interactions between substrate **1k** and IDPi **2f** (**INT-D**).^42,49^ A control experiment using tertiary alcohol substrate **5**—which should react through a tertiary carbocation intermediate—yielded the same product **3k** but with significantly diminished enantioselectivity (66:34 er).^42^ This is in stark contrast to the high enantioselectivity observed for cyclopropane substrate **1k** in the presence of 1 equiv of water at room temperature (93.5:6.5 er). These results are inconsistent with a mechanism in which the ring-opening reaction involves a long-lived, freely diffusing tertiary carbocation (Figure 4A). Kinetic studies on substrate **1k** demonstrated zero-order kinetics over the initial 4–5 h (Figure 4C). This indicates that the reaction rate becomes independent of substrate concentration under standard conditions, suggesting catalyst saturation by the substrate and formation of a strongly bound prereaction complex before the rate-determining step. In ^1^H NMR monitoring, the OH resonance of **1k** shifts from 2.5 to 4.1 ppm as the reaction proceeds, while the ^31^P resonance of the catalyst shows a minor shift upon substrate binding (15.2 → 14.7 ppm) (*see* Supporting Information Figures S6–S8). However, mass spectrometry showed the absence of covalent substrate-catalyst adducts (*see* Supporting Information Figures S9 and S10). Together, these observations suggest that saturation occurs through strong noncovalent interactions (**INT-D**) rather than covalent intermediate formation (**INT-C**). Rate proportionality to **2f** under zero-order kinetics (2 mol % IDPi **2f**) established catalyst concentration as the major rate-determining variable (see Supporting Information, Figure S5). Catalyst comparison shows that with IDPi **2g**, full conversion requires 80 h, suggesting a significant contribution of noncovalent interactions involving the pyrene wing in **2f** to both enantioselectivity and reactivity (Figure 4C). Inhibition by ethanol (1.0 equiv) progressively attenuated the reaction rate concomitant with **1k** consumption and loss of zero-order kinetics (Figure 4C), suggesting direct competition in catalyst binding. Collectively, these results exclude the stepwise (**INT-A**) and covalent (**INT-C**) binding pathways and suggest that the operative mechanism involves strong hydrogen bonding between the hydroxyl of **1k** and a Brønsted acid site of IDPi **2f**, facilitating enantioselective ring-opening via a tightly associated concerted transition state (**INT-D**).

**Figure 4:**
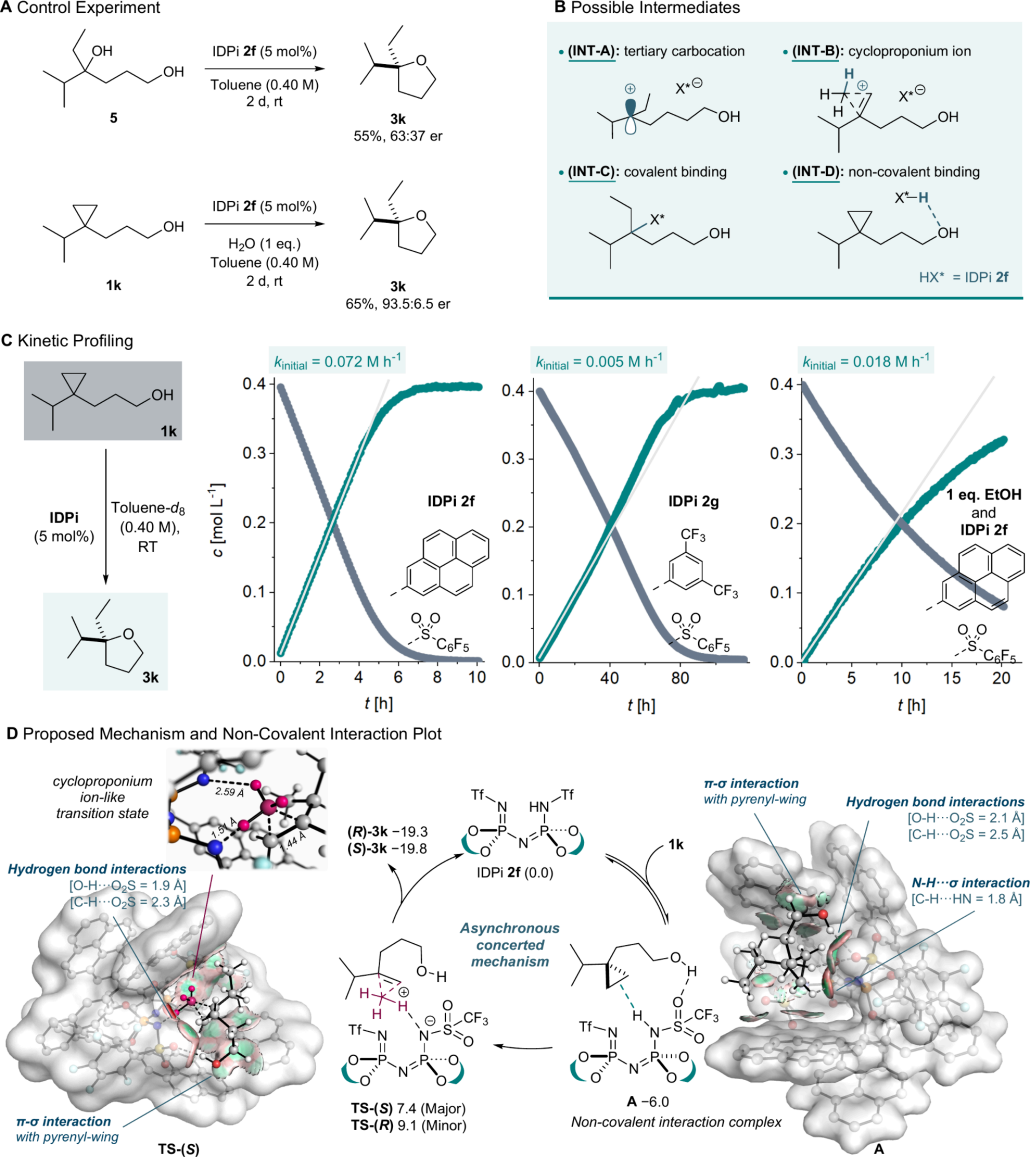
(**A**) Hydroalkoxylation with tertiary alcohol substrate **5**. (**B**) Proposed possible intermediates. (**C**) Reaction profiles monitored by ^1^H NMR spectroscopy. (**D**) A plausible catalytic cycle and DFT study of the reaction at the CPCM­(Toluene)-PBE0-D3­(BJ)/def2-TZVP//CPCM­(Toluene)-PBE0-D3­(BJ)/def2-SVP level of theory (298 K/1 M), Gibbs free energies given in kcal mol^–1^. The IGMH map of the catalyst-substrate precomplex **A** and transition state **TS-(** *S* **)**. A green isosurface represents the dispersion interactions; a blue isosurface represents the hydrogen bond interactions (isovalue: 0.004). Some nonpolar hydrogens are omitted for clarity.

To obtain a further understanding of the reaction mechanism, DFT (density functional theory) studies were performed at CPCM­(Toluene)-PBE0-D3­(BJ)/def2-TZVP//CPCM­(Toluene)-PBE0-D3­(BJ)/def2-SVP level of theory (298 K/1 M) (see Supporting Information).^50–52^ Geometry optimizations starting from the **INT-A** tertiary carbocation/IDPi anion complexes converge to protonated tetrahydrofuran cation structures. Within the explored conformational space, a stable tertiary carbocation minimum was not identified, which is consistent with experimental observations suggesting an asynchronous concerted mechanism (Figure 4D). The IGMH (independent gradient model based on Hirshfeld partition) analysis indicates that the catalyst-substrate prereaction complex **A** and transition state **TS-(** *S* **)** were both stabilized within the catalyst pocket through noncovalent interactions, including C–H···H–N, hydrogen bonding interactions, and π-σ interaction with the pyrenyl-wing (Figure 4D).^53^ The formation of **A** exhibits a Gibbs free energy Δ*G* of – 6.0 kcal mol^–1^, rationalizing the experimentally observed zero-order kinetics attributed to this preassociation. Subsequently, the transition state **TS** connecting intermediate **A** to product **3k** resembles a transient cycloproponium ion-like structure.^30^ Interestingly, enantiodifferentiation arises as the hydroxy group preferentially attacks the quaternary carbon center opposite that of the enantiotopic methylene group of the cyclopropane, which is protonated. The transition state **TS-(** *S* **)** conformer leading to **(** *S* **)-3k** is energetically favored by 1.7 kcal mol^–1^ relative to **TS-(** *R* **)** for **(** *R* **)-3k**, which aligns well with the experimental enantiomeric ratio of 95:5.

In summary, we have developed the first enantioselective nucleophilic addition to unfunctionalized cyclopropanes using confined and highly acidic IDPi **2f**. Mechanistic studies reveal that noncovalent interactions within the IDPi pocket stabilize the catalyst–substrate prereaction complex, resulting in zero-order dependence on the cyclopropane substrate and enabling excellent enantiocontrol. DFT calculations further support the involvement of a transient cycloproponium ion-like transition state in the catalytic cycle.

## Supplementary Material



## Notes

Additional detailed synthetic protocols, analytical data for all compounds and the computational strategy (PDF)
