# Supplementary material for: Asymmetric Carbonium Ion Catalysis: The Intramolecular Hydroalkoxylation of Cyclopropanes
Source: J Am Chem Soc. 2026 Jan 5;148(2):2126–32. doi: 10.1021/jacs.5c20876 (PMC12833807; doi:10.1021/jacs.5c20876)
Supplement: Supplementary file 1 [file ja5c20876_si_001.pdf]

Supplementary Information

Asymmetric Carbonium Ion Catalysis: The Intramolecular Hydroalkoxylation of Cyclopropanes

Fuxing Shi[1], Markus Leutzsch[1], Nils Frank[1], Chendan Zhu[1], Nobuya Tsuji[2], Guanwei Zhang[1], Benjamin List[1,2]*

[1]Max-Planck-Institut für Kohlenforschung, Kaiser-Wilhelm-Platz 1, 45470, Mülheim an der Ruhr, Germany.
[2]Institute for Chemical Reaction Design and Discovery (WPI-ICReDD), Hokkaido University, Sapporo 001-0021, Japan.

*e-mail: list@kofo.mpg.de

Table of Contents

1. General Information and Instrumentation 2
2. Synthesis and Characterization of Cyclopropyl Alcohol Substrate (1a-1p) 4
3. Procedure for Reaction Development 10
4. Enantioselective Intramolecular Hydroalkoxylation of Cyclopropane 11
5. Synthesis and Characterization of Catalysts 17
6. Mechanistic Study 19
   6.1 Deuteration NMR Study 19
   6.2 Reaction Monitoring 22
   6.3 MS detection 24
   6.4 Procedure of etherification of the corresponding alcohol and ester substrate 25
7. GC and HPLC Traces of the Products 26
   7.1. GC trace 26
   7.2. HPLC trace 39
8. ^{1}H, ^{13}C, ^{31}P and ^{19}F NMR spectra of substrates and products 45
9. Computational Studies 84
   9. 1. General 84
   9. 2. Independent gradient model based on Hirshfeld partition (IGMH) 84
   9. 3 List of atomic coordinates 86
References 104

1. General Information and Instrumentation

Chemicals: Unless otherwise indicated, starting materials were obtained from Sigma-Aldrich, ABCR-GmbH, TCI. Commercially available reagents were used without additional purification. The chiral imidodiphosphorimidate acids (IDP, IDPis) **2b–2g** were synthesized according to literature procedures.[1]

Solvents: Solvents (Pentane, Et_2O, THF, CH_2Cl_2, $CHCl_3$ and Toluene) were dried by distillation from an appropriate drying agent in the technical department of the Max-Planck-Institut für Kohlenforschung and received in Schlenk flasks under argon. In addition, more solvents (MTBE and MeCN) were purchased from commercial suppliers and dried over molecular sieves.

Inert Gas: Dry argon was purchased from Air Liquide with >99.5% purity.

Thin Layer Chromatography: Thin-layer chromatography (TLC) was performed using silica gel pre-coated plastic sheets (Polygram SIL G/UV_{254}, 0.2 mm, with fluorescent indicator; Macherey-Nagel) which was visualized with a UV lamp (254 nm) and/or phosphomolybdic acid (PMA), and/or Cerium Ammonium Molybdate (CAM), and/or ninhydrin. PMA stain: PMA (20 g) in EtOH (200 mL). CAM stain: Ammonium molybdate tetrahydrate (2.5 g), Cerium ammonium sulfate dihydrate (1 g) and Sulfuric acid (10 mL) in Water (90 mL). Ninhydrin stain: ninhydrin (1.5 g) in EtOH (200 mL) with AcOH (3 mL).

Column Chromatography: Column chromatography was carried out using Merck silica gel (60 Å, 230–400 mesh, particle size 0.040–0.063 mm) using technical grade solvents. Elution was accelerated using compressed argon. All reported yields, unless otherwise specified, refer to spectroscopically and chromatographically pure compounds.

Nomenclature: Nomenclature follows the suggestions proposed by the computer program ChemBioDraw (12.0.3.1216) of CBD/cambridgesoft.

Nuclear Magnetic Resonance Spectroscopy: ^{1}H, ^{13}C, ^{19}F, ^{31}P Nuclear magnetic resonance (NMR) spectra for compound characterization were recorded on Bruker AVIII-500 MHz, NMR spectrometer in a suitable deuterated solvent. The solvent employed and the respective measuring frequency are indicated for each experiment. Chemical shifts are reported with $CDCl_3$ serving as a universal reference of all nuclides. The resonance multiplicity is described as s (singlet), d (doublet), t (triplet), q (quadruplet), p (pentet), h (heptet), m (multiplet), and br (broad). All spectra were recorded at 298 K, processed with MestReNova 15.0.0 suits of program, and coupling constants are reported as observed. Signals are reported as follows: chemical shift δ in ppm (multiplicity, coupling constant *J* in Hz, number of protons). All X-nuclei spectra were acquired proton decoupled unless otherwise noted.

Mass Spectrometry: Electrospray ionization (ESI) mass spectrometry was conducted on a Bruker ESQ 3000 spectrometer. High resolution mass spectrometry (HRMS) was performed on a Finnigan MAT 95 (EI) or Bruker APEX III FTMS (7 T magnet, ESI). The ionization method and mode of detection employed is indicated for the respective experiment. The ionization method and mode of detection employed is indicated for the respective experiment and all masses are reported in atomic units per elementary charge (*m/z*) with an intensity normalized to the most intense peak.

Specific Rotations: Specific rotations () were measured with a Rudolph RA Autopol IV Automatic Polarimeter at the indicated temperature with a sodium lamp (sodium D line, λ = 589 nm). Measurements were performed in an acid resistant 1 mL cell (50 mm length) with concentrations (g/(100 mL)) reported in the corresponding solvent.

High Performance Liquid Chromatography: High performance liquid chromatography (HPLC) was performed on a Shimadzu LC-20AD liquid chromatograph SIL-20AC auto sampler, CMB-20A using Daicel/Merck columns with a chiral stationary phase. All solvents used were HPLC-grade solvents purchased from Sigma-Aldrich. The column employed and the respective solvent mixture are indicated for each experiment.

Abbreviations: e.r. = enantiomeric ratio, TLC = thin layer chromatography, THF = tetrahydrofuran, MTBE = methyl *tert*-butyl ether, MeCN = acetonitrile, Tf = SO_2CF_3, MOM = methoxymethyl ether.

X-Ray Crystallography: Single crystals suitable for X-ray diffraction were grown as specified in the respective experiment. X-ray crystal structure analyses were performed on a Bruker AXS Enraf-Nonius KappaCCD diffractometer with a FR591 rotating Mo-anode X-ray source Incoatec Helios focusing multilayer optics Bruker-AXS Kappa Mach3 with APEX-II detector and FR591 rotating anode X-ray source with Incoatec Helios mirrors Bruker-AXS Kappa Mach3 with APEX-II detector and lµS microfocus Mo-anode X-ray source and Incoatec Helios mirrors. Data were faceindexed absorption corrected and scaled using the program SADABS (Bruker AXS, 2014). The structure was refined using the programs SHELXS and SHELXL, both programs from G. M. Sheldrick or OLEX29 and NoSpherA210 routine. The X-ray crystal structure analyses were performed in the X-ray department of the Max-Planck-Institut für Kohlenforschung. Crystal structures were visualized and rendered using the program Mercury using version 4.2.0 developed by The Cambridge Crystallographic Data Centre (CCDC).

2. Synthesis and Characterization of Cyclopropyl Alcohol Substrate (1a-1p)

Substrates

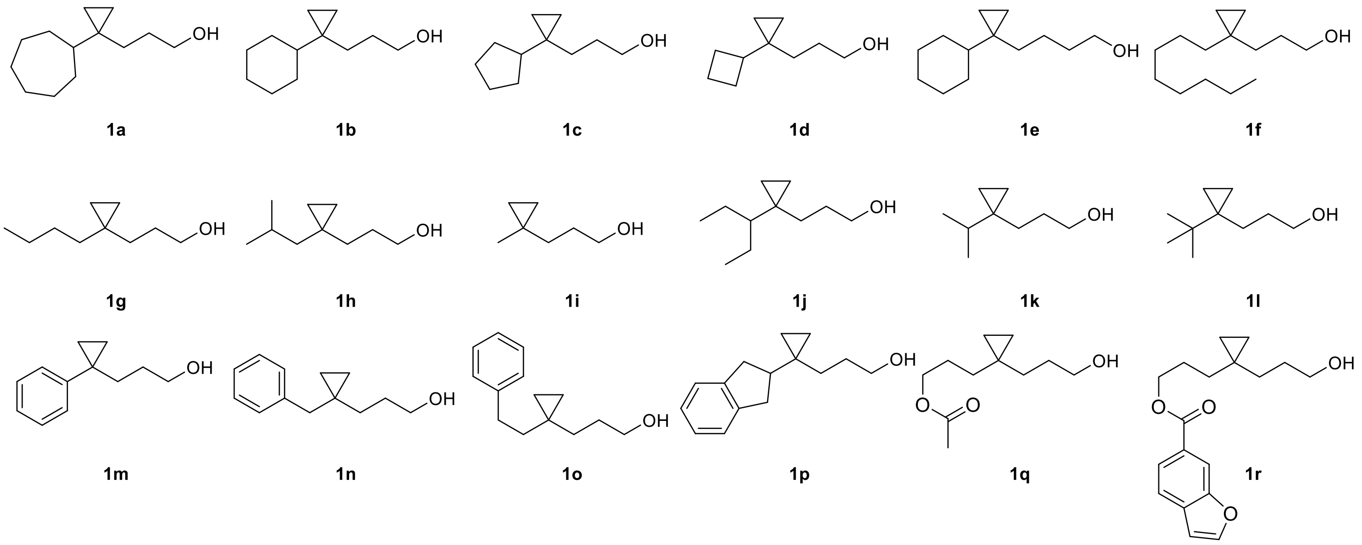

General procedure of substrate synthesis: 1a-1p

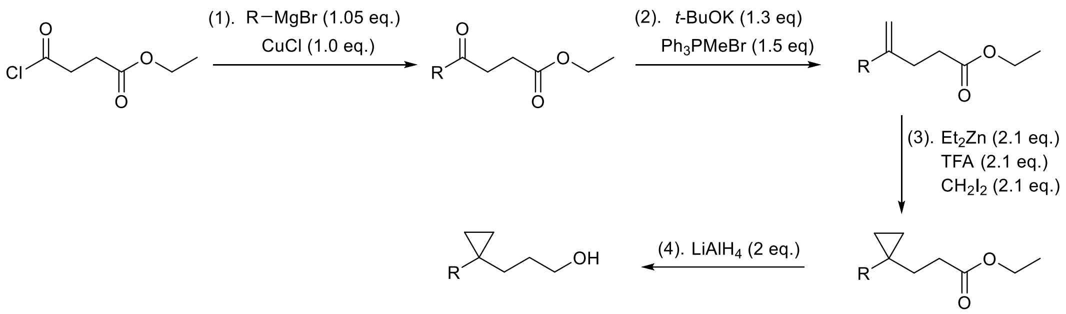

General Procedure: 1a-1p

Preparation of ketone esters (step (1)): Synthesis according to the known procedure.[2] Added a 1 M THF solution of RMgBr (1.05 eq.) dropwised to a solution of 5-chlorovaleryl chloride (1.0 eq.) and CuCl (1.0 eq.) in THF (1 M) via syringe under argon atmosphere at 0 °C. Allow the reaction mixture to warmed to room temperature. Then stirred the reaction mixture overnight.

Quenched the reaction with ice water. Filtered the mixture through a pad of Celite. Washed the organic layer of resulting filtrates with 1 N HCl aqueous three times. Dry the organic layer then concentrate the organic layer. Purified the crude product by silica gel column chromatography (hexane/EtOAc = 10:1).

Preparation of unsaturated ester (step (2)): Dissolved methyl triphenyl phosphonium bromide (1.5 eq.) in THF (0.5 M) in an oven dried round bottom flask. Then, added potassium *tert*-butoxide (1.3 eq.) at 0 °C. Stirred the mixture for 30 min. Added ketone ester starting material (1.0 equiv.) to the suspension at 0 °C. Warmed the mixture to room temperature. Stirred for 16 h then filtered the mixture through a pad of Celite. Dried the organic layer and concentrate the organic layer. Purified the crude product by silica gel column chromatography (hexane/EtOAc = 20:1).

Preparation of cyclopropyl ester (step (3)): According to the known literature.[3] The Et_2Zn (2 eq.) added in CH_2Cl_2 (0.2 M) under argon in a two-neck flask. Cooled to 0 °C, then CF_3COOH (2 eq.) in CH_2Cl_2 (0.5 M) was then dropped

in 1 h, then stir for 20 mins. Then CH_2I_2 (2 eq.) in CH_2Cl_2 (1 M) was added, then stir for 20 mins. The substrate (1 eq.) in CH_2Cl_2 (1 M) was slowly added, then the ice removed and stir for 30 mins. Quenched the reaction mixture with 0.1 NH4Cl, then separated. Aqueous layer extracted with hexanes organic layer washed with saturated $NaHCO_3$. Dried by Na_2SO_4, then filtered, concentrated. Purify the crude product by silica gel column chromatography (hexane/EtOAc = 20:1).

Preparation of cyclopropyl alcohol (step (4)): Added lithium aluminum hydride (2 eq., 1 M solution in THF) dropwise to a round bottomed flask containing ester starting material (1 eq.) and THF (0.4 M). Stir the reaction mixture at 0 °C for 20 minutes and at room temperature for 40 minutes. Quenched the reaction mixture with H_2O (0.7 mL). Filtered the reaction mixture through Celite. Removed the solvent under reduced pressure. Purified the resulting colorless oil by chromatography with hexane/EtOAc (10:1).

3-(1-cycloheptylcyclopropyl)propan-1-ol (1a)

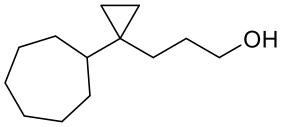

Prepared according to the general procedure as colorless oil (1.21 g, 6.3 mmol, 31% yield after 4 steps). **^{1}H NMR (501 MHz, $CDCl_3$)** δ 3.59 (t, J = 6.4 Hz, 2H), 1.71 (m, J = 16.5, 12.8, 6.3, 3.6 Hz, 4H), 1.56–1.51 (m, 2H), 1.46 (m, J = 12.2, 8.9, 6.0, 3.8 Hz, 4H), 1.41 – 1.28 (m, 6H), 1.21 (s, 1H), 0.76 (tt, J = 10.1, 3.3 Hz, 1H), 0.32 – 0.19 (m, 4H). **^{13}C NMR (126 MHz, $CDCl_3$)** δ 63.8, 48.5, 32.6, 30.3, 28.9, 28.0, 27.8, 25.3, 11.4. **HRMS** m/z (ESI): calculated for $C_{13}H_{24}O$. $[M+H]^+$: 197.1899, found 197.1900.

3-(1-cyclohexylcyclopropyl)propan-1-ol (1b)

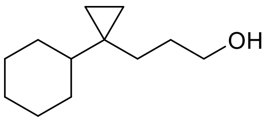

Prepared according to the general procedure as colorless oil (0.59 g, 3.4 mmol, 29% yield after 4 steps). **^{1}H NMR (501 MHz, $CDCl_3$)** δ 3.60 (t, J = 6.7 Hz, 2H), 1.77 – 1.69 (m, 2H), 1.67 – 1.60 (m, 3H), 1.55 – 1.47 (m, 2H), 1.38 – 1.31 (m, 2H), 1.24 – 0.90 (m, 7H), 0.30 – 0.24 (m, 2H), 0.22 – 0.15 (m, 2H). **^{13}C NMR (126 MHz, $CDCl_3$)** δ 63.7, 43.6, 30.5, 30.3, 30.2, 27.1, 26.8, 23.5, 10.0. **HRMS** m/z (ESI): calculated for $C_{12}H_{22}O$. $[M+H]^+$: 183.1743, found 183.1744.

3-(1-cyclopentylcyclopropyl)propan-1-ol (1c)

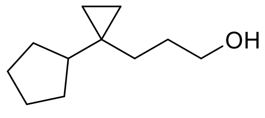

Prepared according to the general procedure as colorless oil (1.1 g, 6.51 mmol, 22% yield after 4 steps). **^{1}H NMR (501 MHz, $CDCl_3$)** δ 3.63 (t, J = 6.7 Hz, 2H), 2.02 (tt, J = 10.4, 7.4 Hz, 1H), 1.58 (s, 6H), 1.52 – 1.46 (m, 2H), 1.38 – 1.32 (m, 2H), 1.31 – 1.13 (m, 1H), 1.00 (m, J = 11.3, 9.3, 7.3 Hz, 2H), 0.38 – 0.30 (m, 2H), 0.20 – 0.12 (m, 2H). **^{13}C NMR (126 MHz, $CDCl_3$)** δ 63.6, 42.5, 34.0, 30.0, 29.1, 25.3, 21.5, 9.3. **HRMS** m/z (ESI): calculated for $C_{11}H_{20}O$. $[M+H]^+$: 169.1587, found 169.1587.

3-(1-cyclobutylcyclopropyl)propan-1-ol (1d)

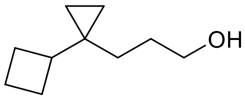

Prepared according to the general procedure as colorless oil (1.0 g, 6.47 mmol, 32% yield after 4 step). **^{1}H NMR (501 MHz, $CDCl_3$)** δ 3.61 (t, J = 6.6 Hz, 2H), 2.64 (tt, J = 9.2, 7.5 Hz, 1H), 1.85 – 1.70 (m, 3H), 1.64 – 1.60 (m, 1H), 1.59 – 1.56 (m, 2H), 1.54 – 1.45 (m, 2H), 1.30

– 1.25 (m, 2H), 1.21 (s, 1H), 0.42 – 0.34 (m, 2H), 0.17 – 0.09 (m, 2H). **^{13}C NMR (126 MHz, CDCl$_3$)** δ 63.6, 38.7, 33.6, 30.3, 25.4, 21.8, 17.7, 8.1. **HRMS** m/z (ESI): calculated for $C_{10}H_{18}O$. $[M+H]^+$: 155.1430, found 155.1431.

4-(1-cyclohexylcyclopropyl)butan-1-ol (1e)

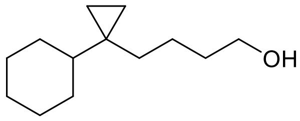

Prepared according to the general procedure as colorless oil (2.24 g, 11.1 mmol, 25% yield after 4 step). **^{1}H NMR (501 MHz, CDCl$_3$)** δ 3.65 (t, *J* = 6.6 Hz, 2H), 1.75 (dt, *J* = 12.7, 3.0 Hz, 2H), 1.68 – 1.62 (m, 3H), 1.57 – 1.51 (m, 2H), 1.32 (dd, *J* = 5.7, 3.9 Hz, 4H), 1.24 – 0.89 (m, 7H), 0.27 (t, *J* = 2.8 Hz, 2H), 0.22 – 0.17 (m, 2H). **^{13}C NMR (126 MHz, CDCl$_3$)** δ 63.3, 43.6, 34.4, 33.5, 30.2, 27.1, 26.8, 23.8, 23.2, 9.9. **HRMS** m/z (ESI): calculated for $C_{13}H_{24}O$. $[M+H]^+$: 214.2165, found 214.2165.

3-(1-octylcyclopropyl)propan-1-ol (1f)

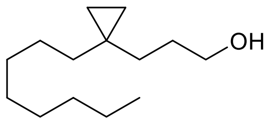

Prepared according to the general procedure as colorless oil (0.52 g, 2.2 mmol, 28% yield after 4 step). **^{1}H NMR (501 MHz, CDCl$_3$)** δ 3.64 (t, *J* = 6.7 Hz, 2H), 1.66 – 1.59 (m, 2H), 1.35 – 1.17 (m, 17H), 0.88 (t, *J* = 6.9 Hz, 3H), 0.22 (dt, *J* = 4.4, 1.5 Hz, 4H). **^{13}C NMR (126 MHz, CDCl$_3$)** δ 63.5, 36.1, 32.3, 32.1, 30.1, 30.1, 29.9, 29.5, 26.7, 22.8, 19.1, 14.3, 12.2. **HRMS** m/z (ESI): calculated for $C_{14}H_{28}O$. $[M+H]^+$: 213.2213, found 213.2215.

3-(1-butylcyclopropyl)propan-1-ol (1g)

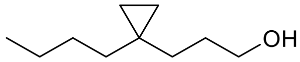

Prepared according to the general procedure as colorless oil (0.41 g, 2.5 mmol, 19% yield after 4 step). **^{1}H NMR (501 MHz, CDCl$_3$)** δ 3.64 (t, *J* = 6.7 Hz, 2H), 1.65 – 1.59 (m, 2H), 1.35 – 1.17 (m, 9H), 0.89 (t, *J* = 6.9 Hz, 3H), 0.22 (dt, *J* = 4.4, 1.5 Hz, 4H). **^{13}C NMR (126 MHz, CDCl$_3$)** δ 63.5, 35.8, 32.3, 30.0, 28.9, 23.2, 19.1, 14.3, 12.2. **HRMS** m/z (ESI): calculated for $C_{10}H_{20}O$. $[M+H]^+$: 157.1587, found 157.1587.

3-(1-isobutylcyclopropyl)propan-1-ol (1h)

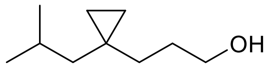

Prepared according to the general procedure as colorless oil (0.64 g, 3.6 mmol, 17% yield after 4 step). **^{1}H NMR (501 MHz, CDCl$_3$)** δ 3.64 (t, *J* = 6.7 Hz, 2H), 1.76 (m, *J* = 13.6, 6.8 Hz, 1H), 1.66 – 1.60 (m, 2H), 1.57 (s, 1H), 1.29 – 1.25 (m, 2H), 1.12 (d, *J* = 7.3 Hz, 2H), 0.90 (d, *J* = 6.6 Hz, 6H), 0.22 (dt, *J* = 6.2, 1.6 Hz, 4H). **^{13}C NMR (126 MHz, CDCl$_3$)** δ 63.5, 45.0, 32.1, 30.0, 26.2, 23.2, 17.4, 12.4. **HRMS** m/z (ESI): calculated for $C_{10}H_{20}O$. $[M+H]^+$: 157.1587, found 157.1588.

3-(1-methylcyclopropyl)propan-1-ol (1i)

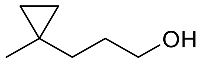

Prepared according to the general procedure as colorless oil (0.21 g, 2.1 mmol, 11% yield after 4 step). **^{1}H NMR (501 MHz, CDCl$_3$)** δ 3.65 (td, *J* = 6.7, 1.0 Hz, 2H), 1.70 – 1.63 (m, 2H), 1.59 (s, 1H), 1.30 – 1.26 (m, 2H), 1.03 (d, *J* = 0.9 Hz, 3H), 0.34 – 0.20 (m, 4H). **^{13}C NMR (126 MHz, CDCl$_3$)** δ 63.4, 35.6, 30.4, 22.8, 15.1, 13.1. **HRMS** m/z (ESI): calculated for $C_7H_{14}O$. $[M+H]^+$: 115.1117, found 115.1119.

3-(1-(pentan-3-yl)cyclopropyl)propan-1-ol (1j)

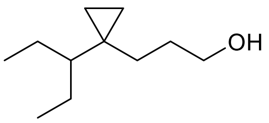

Prepared according to the general procedure as colorless oil (0.23 g, 1.4 mmol, 19% yield after 4 step). **^{1}H NMR (501 MHz, $CDCl_3$)** δ 3.59 (td, *J* = 6.6, 3.9 Hz, 2H), 1.49 – 1.42 (m, 2H), 1.41 – 1.34 (m, 6H), 1.23 – 1.16 (m, 1H), 0.91 (t, *J* = 7.5 Hz, 6H), 0.52 (p, *J* = 6.9 Hz, 1H), 0.32 – 0.19 (m, 4H). **^{13}C NMR (126 MHz, $CDCl_3$)** δ 63.6, 49.9, 40.0, 30.0, 28.3, 25.7, 13.1, 10.7. **HRMS** m/z (ESI): calculated for $C_{11}H_{22}O$. $[M+H]^+$: 171.1743, found 171.1742.

3-(1-isopropylcyclopropyl)propan-1-ol (1k)

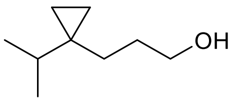

Prepared according to the general procedure as colorless oil (0.88 g, 6.2 mmol, 24% yield after 4 step). **^{1}H NMR (501 MHz, $CDCl_3$)** δ 3.61 (t, *J* = 6.6 Hz, 2H), 1.55 – 1.49 (m, 2H), 1.42 – 1.24 (m, 4H), 0.87 (d, *J* = 6.9 Hz, 6H), 0.29 – 0.18 (m, 4H). **^{13}C NMR (126 MHz, $CDCl_3$)** δ 63.7, 33.1, 30.1, 29.9, 23.8, 19.6, 10.1. **HRMS** m/z (ESI): calculated for $C_9H_{18}O$. $[M+H]^+$: 143.1430, found 143.1431.

3-(1-(tert-butyl)cyclopropyl)propan-1-ol (1l)

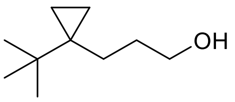

Prepared according to the general procedure as colorless oil (0.35 g, 2.3 mmol, 15% yield after 4 step). **^{1}H NMR (501 MHz, $CDCl_3$)** δ 3.58 (t, *J* = 6.3 Hz, 2H), 1.54 – 1.40 (m, 4H), 1.24 (s, 1H), 0.85 (s, 9H), 0.47 – 0.40 (m, 2H), 0.22 – 0.15 (m, 2H). **^{13}C NMR (126 MHz, $CDCl_3$)** δ 63.9, 32.6, 30.8, 29.7, 27.8, 25.9, 7.9. **HRMS** m/z (ESI): calculated for $C_{10}H_{20}O$. $[M+H]^+$: 157.1587, found 157.1588.

3-(1-phenylcyclopropyl)propan-1-ol (1m)

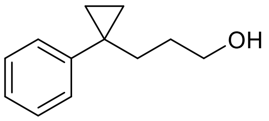

Prepared according to the general procedure as colorless oil (0.67 g, 3.8 mmol, 23% yield after 4 step). **^{1}H NMR (501 MHz, $CDCl_3$)** δ 7.33 – 7.25 (m, 4H), 7.20 – 7.15 (m, 1H), 3.57 (t, *J* = 6.4 Hz, 2H), 1.66 – 1.60 (m, 2H), 1.58 – 1.51 (m, 2H), 1.28 (d, *J* = 13.8 Hz, 1H), 0.85 – 0.79 (m, 2H), 0.71 – 0.64 (m, 2H). **^{13}C NMR (126 MHz, $CDCl_3$)** δ 145.2, 129.1, 128.3, 126.1, 63.0, 36.7, 30.6, 25.5, 13.1. **HRMS** m/z (ESI): calculated for $C_{12}H_{16}O$. $[M+Na]^+$: 199.1093, found 199.1095.

3-(1-benzylcyclopropyl)propan-1-ol (1n)

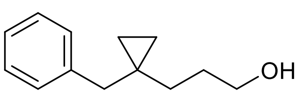

Prepared according to the general procedure as colorless oil (0.53 g, 2.8 mmol, 19% yield after 4 step). **^{1}H NMR (501 MHz, $CDCl_3$)** δ 7.28 (t, *J* = 7.4 Hz, 2H), 7.25 – 7.18 (m, 3H), 3.59 (t, *J* = 6.7 Hz, 2H), 2.61 (s, 2H), 1.70 – 1.63 (m, 2H), 1.27 – 1.15 (m, 3H), 0.49 – 0.43 (m, 2H), 0.37 – 0.31 (m, 2H). **^{13}C NMR (126 MHz, $CDCl_3$)** δ 140.4, 129.4, 128.3, 126.2, 63.3, 41.8, 32.2, 30.1, 20.2, 11.7. **HRMS** m/z (ESI): calculated for $C_{13}H_{18}O$. $[M+Na]^+$: 213.1251, found 213.1250.

3-(1-phenethylcyclopropyl)propan-1-ol (1o)

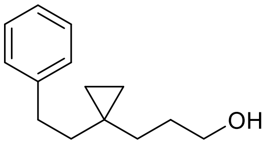

Prepared according to the general procedure as colorless oil (0.61 g, 3.0 mmol, 29% yield after 4 step). **^{1}H NMR (501 MHz, $CDCl_3$)** δ 7.27 (d, *J* = 7.5 Hz, 2H), 7.21 – 7.14 (m, 3H), 3.66 (t, *J* = 6.7 Hz, 2H), 2.70 – 2.62 (m, 2H), 1.73 – 1.51 (m, 5H), 1.42 – 1.36 (m, 2H), 0.29

(s, 4H). **^{13}C NMR (126 MHz, CDCl$_3$)** δ 142.9, 128.4, 125.7, 63.4, 38.3, 33.2, 32.1, 30.0, 27.1, 19.3, 12.3. **HRMS** m/z (ESI): calculated for $C_{14}H_{20}O$. $[M+Na]^+$: 227.1406, found 227.1407.

3-(1-(2,3-dihydro-1H-inden-2-yl)cyclopropyl)propan-1-ol (1p)

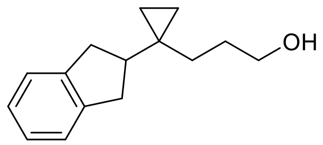

Prepared according to the general procedure as colorless oil (0.74 g, 3.4 mmol, 37% yield after 4 step). **^{1}H NMR (501 MHz, CDCl$_3$)** δ 7.24 – 7.00 (m, 4H), 3.63 (t, J = 6.6 Hz, 2H), 2.93 – 2.81 (m, 2H), 2.78 – 2.67 (m, 1H), 2.56 (dd, J = 15.0, 9.9 Hz, 2H), 1.68 – 1.61 (m, 2H), 1.55 (s, 1H), 1.46 – 1.41 (m, 2H), 0.53 – 0.45 (m, 2H), 0.35 – 0.25 (m, 2H). **^{13}C NMR (126 MHz, CDCl$_3$)** δ 143.3, 126.1, 124.3, 63.3, 43.2, 35.9, 33.3, 29.8, 21.4, 9.4. **HRMS** m/z (ESI): calculated for $C_{15}H_{20}O$. $[M+Na]^+$: 217.1587, found 217.1586.

General Procedure: 1q, 1r

General procedure of substrate synthesis: 1q, 1r

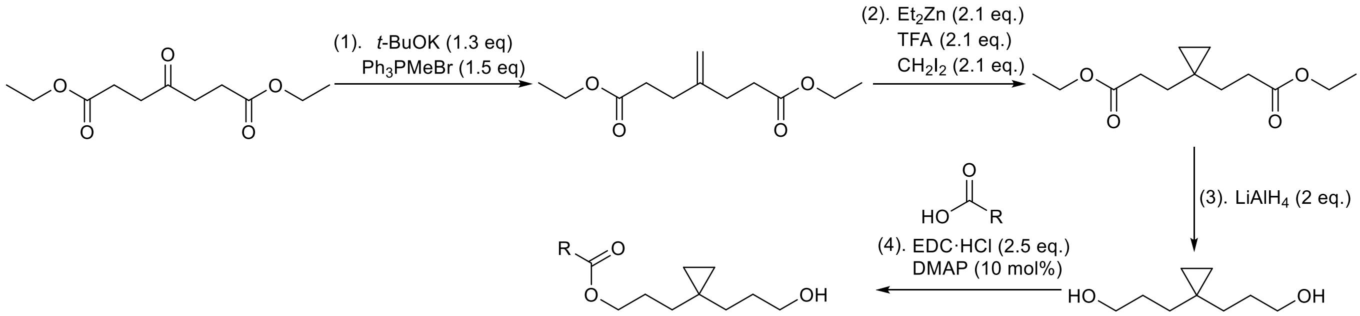

Preparation of cyclopropane alcohol (step (1)-(3)): See general procedure of substrate **1a**–**1p**.

Preparation of functionalized cyclopropane alcohol (step (4)):

In a dry round-bottom flask equipped with a magnetic stir bar, the diol substrate (1.0 equiv) was dissolved in dry dichloromethane (0.4 M) under an inert atmosphere. Carboxylic acid (1.0 equiv) was added, followed by DMAP (0.1 equiv). The reaction mixture was cooled to 0 °C, and 1-Ethyl-3-(3-dimethylaminopropyl)carbodiimide hydrochloride (EDC·HCl) (2.5 equiv) was added.

The reaction was allowed to warm to room temperature and stirred for overnight, during which the progress was monitored by TLC. Upon completion, the reaction was quenched with saturated aqueous NH_4Cl and extracted with dichloromethane, then dried over anhydrous Na_2SO_4, filtered, and concentrated under reduced pressure. The residual crude was purified by silica gel column chromatography (hexane/EtOAc = 10/2.)

3-(1-(3-hydroxypropyl)cyclopropyl)propyl acetate (1q)

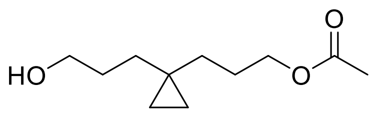

Prepared according to the general procedure as colorless oil (0.09 g, 0.45 mmol, 29% yield after 4 step). **^{1}H NMR (501 MHz, CDCl$_3$)** δ 8.19 (s, 1H), 7.95 (dd, J = 8.2, 1.3 Hz, 1H), 7.76 (d, J = 2.1 Hz, 1H), 7.63 (d, J = 8.2 Hz, 1H), 6.85 – 6.79 (m, 1H), 4.35 (t, J = 6.7 Hz, 2H), 3.65 (t, J = 6.6 Hz, 2H), 1.91 – 1.81 (m, 2H), 1.69 – 1.60 (m, 2H), 1.44 – 1.39 (m, 2H), 1.37 – 1.32 (m, 2H), 0.29 (s, 4H). **^{13}C NMR (126 MHz, CDCl$_3$)** δ 171.4, 64.8, 63.3, 32.2, 32.0, 29.9, 25.9, 21.2, 18.6, 12.2.

3-(1-(3-hydroxypropyl)cyclopropyl)propyl benzofuran-6-carboxylate (1r)

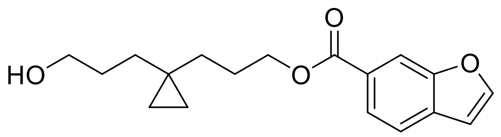

Prepared according to the general procedure as colorless oil (0.18 g, 0.6 mmol, 32% yield after 4 step). **^{1}H NMR (501 MHz, $CDCl_3$)** δ 8.19 (s, 1H), 7.95 (dd, *J* = 8.2, 1.3 Hz, 1H), 7.76 (d, *J* = 2.1 Hz, 1H), 7.63 (d, *J* = 8.2 Hz, 1H), 6.85 – 6.79 (m, 1H), 4.35 (t, *J* = 6.7 Hz, 2H), 3.65 (t, *J* = 6.6 Hz, 2H), 1.91 – 1.81 (m, 2H), 1.69 – 1.60 (m, 2H), 1.44 – 1.39 (m, 2H), 1.37 – 1.32 (m, 2H), 0.29 (s, 4H). **^{13}C NMR (126 MHz, $CDCl_3$)** δ 166.9, 154.6, 148.0, 131.8, 126.8, 124.2, 121.0, 113.2, 107.0, 65.3, 63.3, 32.4, 32.0, 30.0, 26.1, 18.6, 12.3.

3. Procedure for Reaction Development

An oven-dried 1.5 mL vial was charged with catalyst and a magnetic stir bar under an atmosphere of argon, dry solvent toluene was added. The vial was cooled to required reaction temperature. Then **1k** (6.0 µL, 0.04 mmol, 1.0 equiv.) was added and the reaction was stirred for 1 week.

The reaction mixture was quenched with triethylamine (0.04 mmol, 5.6 µL) at reaction temperature, then CH_2Br_2 (internal standard, 2.8 µL, 0.04 mmol, 1.0 equiv.) was added. Then an aliquot of the reaction mixture was taken to determine the yield by crude ^{1}H NMR in $CDCl_3$. The product was purified by prepTLC (Et_2O:Pentane = 1:10) and the er was determined by chiral GC.

Table S1. Results of Initial Screening of Catalysts

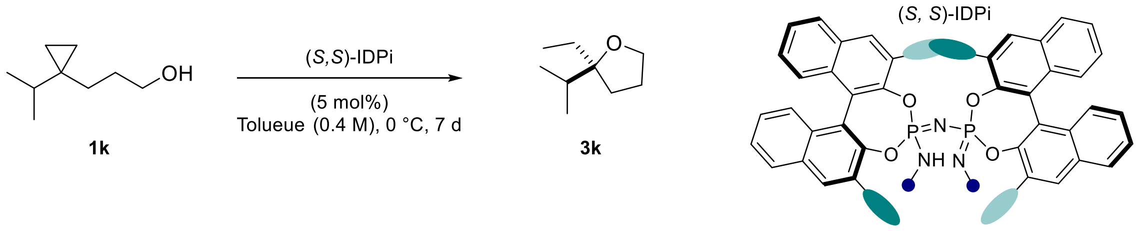

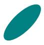		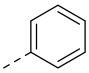	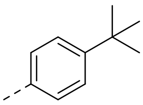	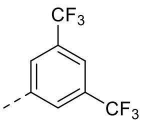	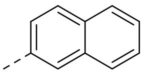	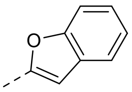	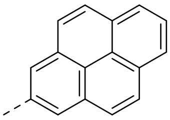
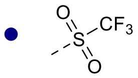	yield:<br>er :	97%<br>52:48	45%<br>62.5:37.5	91%<br>50:50	95%<br>77.5:22.5	92%<br>73:27	87%<br>85:15
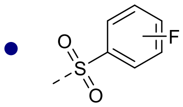	yield:<br>er :	72%<br>61:39	5%<br>82.5:17.5	93%<br>57.5:42.5	80%<br>82:18	97%<br>85.5:14.5	92%<br>95:5

4. Enantioselective Intramolecular Hydroalkoxylation of Cyclopropane

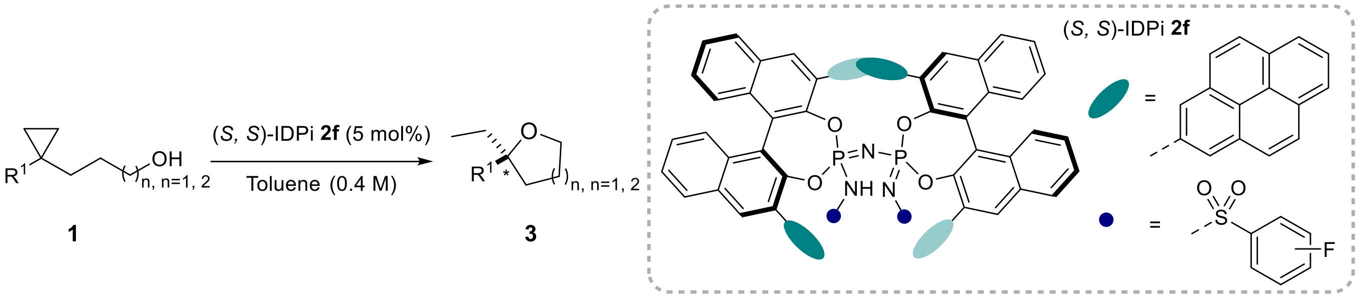

General Procedure for Enantioselective Hydroalkoxylation: A 5 ml vial was charged with IDPi **2f** (10.0 μmol, 5 mol%) and a magnetic stir bar under an atmosphere of argon. Dry toluene (0.4 mL) was added. The vial was cooled to relevant temperature. The substrate **1** (0.2 mmol, 1.0 equiv.) was added dropwise and the reaction was stirred at desired temperature for 7 days (**3a**–**3d**: –10 °C; **3f**, **3g**, **3h**, **3k**, **3n**, **3p**: 0 °C; **3e**, **3i**, **3o**: 25 °C; **3l**: 40 °C; **3m**: 60 °C).

Afterwards, the reaction mixture was treated with triethylamine (0.04 mmol, 5.6 μL, 0.4 equiv.). After the mixture was stirred for 10 min, the solvent of the reaction mixture was directly removed under reduced pressure. The product was purified by column chromatography on silica gel (pentane:diethyl ether = 10:1), then analyzed by chiral HPLC and GC to determine the enantiomeric ratio (er).

Racemate synthesis: The racemic product was synthesized at room temperature for 1 day by using bistriflimide (Tf_2NH, 20 mol%) as the catalyst with the standard purification process.

Note: Based on the volatility of the substrates **3i** and **3l**, it is impossible to completely remove the solvent from the result. The isolated yield is calculated after reducing the solvent mass.

(*S*)-2-cycloheptyl-2-ethyltetrahydrofuran (3a)

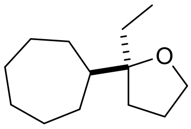

Obtained following the General Procedure, as a colorless oil (28.7 mg, 0.146 mmol, 73%). **^{1}H NMR (501 MHz, $CDCl_3$)** δ 3.81 – 3.64 (m, 2H), 1.78 – 1.29 (m, 17H), 1.10 (m, *J* = 13.2, 9.7, 3.6 Hz, 2H), 0.79 (t, *J* = 7.4 Hz, 3H). **^{13}C NMR (126 MHz, $CDCl_3$)** δ 88.4, 68.0, 46.5, 32.3, 29.9, 28.9, 28.8, 28.5, 28.3, 27.9, 27.8, 27.0, 8.2. **HRMS m/z (ESI):** calculated for $C_{13}H_{24}O$. $[M+Na]^+$: 219.1719, found 219.1719.

GC: 25.0 m Ivadex-1, injection temperature: 100 °C, 0.5 °C/min, 120 °C, iso 5 min, 20°C/min, 220 °C 0.5 bar H_2. t_{R1} = 35.9 min (minor), t_{R2} = 36.4 min (major). **e.r.:** 96.5:3.5. $[\alpha]_D^{25}$ = –4.0 (*c* = 1.9, $CHCl_3$).

(*S*)-2-cyclohexyl-2-ethyltetrahydrofuran (3b)

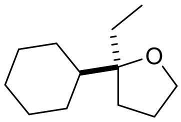

Obtained following the General Procedure, as a white oil (33.5 mg, 0.184 mmol,92%). **^{1}H NMR (501 MHz, $CDCl_3$)** δ 3.87 – 3.73 (m, 2H), 1.73 (d, *J* = 33.9 Hz, 8H), 1.58 – 1.42 (m, 4H), 1.28 – 1.10 (m, 3H), 1.04 – 0.93 (m, 2H), 0.87 (t, *J* = 7.4 Hz, 3H). **^{13}C NMR (126 MHz, $CDCl_3$)** δ 87.1, 68.0, 44.9, 31.8, 29.5, 28.0, 27.3, 27.0, 26.86, 26.88, 26.7, 8.1. **HRMS m/z (ESI):** calculated for $C_{12}H_{22}O$. $[M+H]^+$: 183.1743, found 183.1744.

GC: 25.0 m Ivadex-1, injection temperature: 80 °C, 1 °C/min, 110 °C, iso 5 min, 20 °C/min, 220°C. 0.5 bar H_2. t_{R1} = 22.0 min (minor), t_{R2} = 22.3 min (major). **e.r.:** 96.5:3.5. $[\alpha]_D^{25}$ = –4.7 (c = 1.5, $CHCl_3$).

(*S*)-2-cyclopentyl-2-ethyltetrahydrofuran (3c)

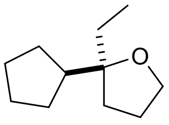

Obtained following the General Procedure, as a white oil (29.9 mg, 0.178 mmol, 89%). **^{1}H NMR (501 MHz, $CDCl_3$)** δ 3.83 (t, J = 6.7 Hz, 2H), 2.14 (m, J = 10.0, 7.6 Hz, 1H), 1.94 – 1.83 (m, 2H), 1.78 – 1.74 (m, 1H), 1.70 – 1.49 (m, 9H), 1.40 – 1.22 (m, 2H), 0.89 (t, J = 7.4 Hz, 3H). **^{13}C NMR (126 MHz, $CDCl_3$)** δ 87.0, 68.2, 46.8, 32.0, 31.6, 27.8, 27.2, 26.8, 25.7, 25.7, 8.6. **HRMS m/z (ESI):** calculated for $C_{11}H_{20}O$. $[M+H]^+$: 169.1587, found 169.1588.

GC: 25.0 m Ivadex-1, injection temperature: 100 °C, 0.5 °C/min, 115 °C, iso 5 min, 20 °C/min, 220 °C. 0.5 bar H_2. t_{R1} = 23.0 min (minor), t_{R2} = 23.3 min (major). **e.r.:** 96:4. $[\alpha]_D^{25}$ = -0.4 (c = 1.4, $CHCl_3$).

(*S*)-2-cyclobutyl-2-ethyltetrahydrofuran (3d)

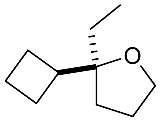

Obtained following the General Procedure, as a white oil (19.1 mg, 0.124 mmol, 62%). **^{1}H NMR (501 MHz, $CDCl_3$)** δ 3.90 – 3.76 (m, 2H), 2.52 (m, J = 9.3, 7.2 Hz, 1H), 2.01 – 1.91 (m, 1H), 1.90 – 1.74 (m, 6H), 1.62 (m, J = 12.3, 8.0, 3.5 Hz, 3H), 1.40 (m, J = 7.5, 1.7 Hz, 2H), 0.82 (t, J = 7.5 Hz, 3H). **^{13}C NMR (126 MHz, $CDCl_3$)** δ 85.9, 68.7, 43.1, 31.4, 30.2, 26.9, 23.4, 23.3, 17.5, 9.1. **HRMS m/z (ESI):** calculated for $C_{10}H_{18}O$. $[M+H]^+$: 155.1430, found 155.1432.

GC: 25.0 m Ivadex-1, injection temperature: 75 °C, 0.5°C/min, 85°C, iso 5 min, 20 °C/min, 220 °C, 0.5 bar H_2. t_{R1} = 18.6 min (minor), t_{R2} = 19.8 min (major). **e.r.:** 94.5:5.5. $[\alpha]_D^{25}$ = +8.6 (c = 1.2, $CHCl_3$).

(*S*)-2-cyclohexyl-2-ethyltetrahydro-2H-pyran (3e)

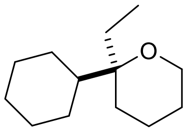

Obtained following the General Procedure, as a white oil (34.2 mg, 0.174 mmol, 87%). **^{1}H NMR (501 MHz, $CDCl_3$)** δ 3.59 (m, J = 11.7, 5.4 Hz, 2H), 1.84 – 1.73 (m, 4H), 1.69 – 1.51 (m, 6H), 1.45 (qd, J = 6.3, 4.7 Hz, 2H), 1.37 (dt, J = 14.8, 7.4 Hz, 1H), 1.30 (td, J = 8.0, 4.1 Hz, 1H), 1.22 (m, J = 12.7, 9.4, 3.3 Hz, 2H), 1.15 – 0.88 (m, 3H), 0.81 (t, J = 7.5 Hz, 3H). **^{13}C NMR (126 MHz, $CDCl_3$)** δ 76.3, 61.0, 42.2, 28.3, 27.3, 27.2, 27.1, 27.0, 26.8, 26.2, 23.2, 19.0, 6.9. **HRMS m/z (ESI):** calculated for $C_{13}H_{24}O$. $[M+NH_4]^+$: 214.2165, found 214.2169.

GC: 25.0 m Ivadex-1, injection temperature: 90 °C, 1 °C/min, 130°C, iso 5 min, 20 °C/min, 220 °C, 0.5 bar H_2. t_{R1} = 32.8 min (minor), t_{R2} = 33.8 min (major). **e.r.:** 13.5:86.5. $[\alpha]_D^{25}$ = –7.3 (c = 1.7, $CHCl_3$).

(*R*)-2-ethyl-2-octyltetrahydrofuran (3f)

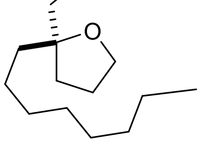

Obtained following the General Procedure, as a white oil (40.8 mg, 0.192 mmol, 96%). **^{1}H NMR (501 MHz, $CDCl_3$)** δ 3.78 (t, J = 6.7 Hz, 2H), 1.90 – 1.82 (m, 2H), 1.65 (dd, J = 8.1, 6.3 Hz, 2H), 1.56 – 1.38 (m, 4H), 1.26 (s, 12H), 0.86 (dt, J = 10.1, 7.1 Hz, 6H). **^{13}C NMR (126 MHz, $CDCl_3$)** δ 85.3, 67.4, 38.2, 34.7, 32.1, 31.0, 30.5, 29.8, 29.5, 26.4, 24.5, 22.8, 14.2, 8.8. **HRMS m/z (ESI):** calculated for $C_{14}H_{28}O$. $[M+H]^+$: 213.2213, found 213.2214.

GC: 25.0 m Ivadex-1, injection temperature: 90 °C, iso 150 min, 20 °C/min, 220 °C, 0.5 bar H_2. t_{R1} = 47.8 min (major), t_{R2} = 49.1 min (minor). **e.r.:** 92:8. $[\alpha]_D^{25}$ = –7.3 (c = 1.7, $CHCl_3$).

(*R*)-2-butyl-2-ethyltetrahydrofuran (3g)

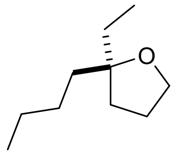

Obtained following the General Procedure, as a white oil (24.1 mg, 0.154 mmol, 77%). **^{1}H NMR (501 MHz, $CDCl_3$)** δ 3.82 – 3.74 (m, 2H), 1.93 – 1.82 (m, 2H), 1.69 – 1.62 (m, 2H), 1.56 – 1.39 (m, 4H), 1.32 – 1.22 (m, 4H), 0.87 (dt, *J* = 21.4, 7.2 Hz, 6H). **^{13}C NMR (126 MHz, $CDCl_3$)** δ 85.3, 67.4, 37.9, 34.7, 31.0, 26.7, 26.4, 23.5, 14.3, 8.8. **HRMS m/z (ESI):** calculated for $C_{10}H_{20}O$. $[M+H]^+$: 157.1587, found 157.1589.

GC: 25.0 m Ivadex-1, injection temperature: 65 °C, iso 32 min, 20 °C/min, 220 °C, 0.5 bar H_2. t_{R1} = 27.2 min (major), t_{R2} = 27.7 min (minor). **e.r.:** 93.5:6.5. $[\alpha]_D^{25}$ = 2.8 (*c* = 1.1, $CHCl_3$).

(*S*)-2-ethyl-2-isobutyltetrahydrofuran (3h)

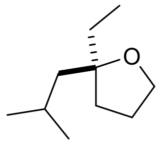

Obtained following the General Procedure, as a white oil (23.0 mg, 0.130 mmol, 65%). **^{1}H NMR (501 MHz, $CDCl_3$)** δ 3.78 (t, *J* = 6.8 Hz, 2H), 1.91 – 1.82 (m, 2H), 1.71 (s, 3H), 1.59 – 1.52 (m, 1H), 1.51 – 1.42 (m, 2H), 1.34 (dd, *J* = 14.2, 6.2 Hz, 1H), 0.93 (m, *J* = 13.7, 6.6 Hz, 6H), 0.86 (t, *J* = 7.5 Hz, 3H). **^{13}C NMR (126 MHz, $CDCl_3$)** δ 85.7, 67.0, 46.6, 35.6, 31.1, 26.3, 24.7, 24.6, 24.6, 8.9. **HRMS m/z (ESI):** calculated for $C_{10}H_{20}O$. $[M+H]^+$: 157.1587, found 157.1588.

GC: 25.0 m Cyclosil B, injection temperature: 60 °C iso 60 min, 20°C/min, 220°C, 0.5 bar H_2. t_{R1} = 53.0 min (major), t_{R2} = 56.5 min (minor). **e.r.:** 94.5:5.5. $[\alpha]_D^{25}$ = –1.2 (*c* = 1.5, $CHCl_3$).

(*S*)-2-ethyl-2-methyltetrahydrofuran (3i)

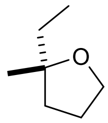

Obtained following the General Procedure, as a white oil (11.6 mg, 0.102 mmol, 51%). **^{1}H NMR (501 MHz, $CDCl_3$)** δ 3.95 – 3.68 (m, 2H), 1.99 – 1.83 (m, 2H), 1.72 (m, *J* = 12.3, 8.3, 7.1 Hz, 1H), 1.65 – 1.59 (m, 1H), 1.56 – 1.48 (m, 2H), 1.16 (s, 3H), 0.90 (t, *J* = 7.5 Hz, 3H). **^{13}C NMR (126 MHz, $CDCl_3$)** δ 67.3, 36.3, 33.8, 29.9, 26.3, 25.3, 9.2. **HRMS m/z (ESI):** calculated for $C_7H_{14}O$. $[M+H]^+$: 115.1117, found 115.1118.

GC: 25.0 m BGB-174, injection temperature: 40 °C iso 18 min, 8°C/min, 240°C, 0.5 bar H_2. t_{R1} = 13.0 min (minor), t_{R2} = 14.5 min (major). **e.r.:** 84:16. $[\alpha]_D^{25}$ = –3.8 (*c* = 0.2, $CHCl_3$).

(*S*)-2-ethyl-2-(pentan-3-yl)tetrahydrofuran (3j)

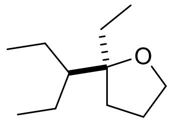

Obtained following the General Procedure, as a white oil (29.3 mg, 0.172 mmol, 86%). **^{1}H NMR (501 MHz, $CDCl_3$)** δ 3.86 – 3.70 (m, 2H), 1.90 – 1.80 (m, 2H), 1.77 – 1.69 (m, 1H), 1.64 – 1.59 (m, 1H), 1.57 – 1.42 (m, 4H), 1.31 (m, *J* = 8.3, 6.9, 3.5 Hz, 1H), 1.24 – 1.10 (m, 2H), 0.98 – 0.82 (m, 9H). **^{13}C NMR (126 MHz, $CDCl_3$)** δ 88.5, 67.8, 49.0, 32.7, 29.3, 27.0, 23.3, 23.0, 14.1, 13.8, 8.2. **HRMS m/z (CI):** calculated for $C_{11}H_{22}O$. $[M+H]^+$: 171.1743, found 171.1744.

GC: 25.0 m Ivadex-1, injection temperature: 80 °C, iso 5 min, 1 °C/min, 95 °C, iso 5 min, 20 °C/min, 220 °C, 0.5 bar H_2. t_{R1} = 13.5 min (major), t_{R2} = 13.9 min (minor). **e.r.:** 95.5:4.5. $[\alpha]_D^{25}$ = –29.3 (*c* = 0.3, $CHCl_3$).

(*S*)-2-ethyl-2-isopropyltetrahydrofuran (3k)

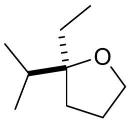

Obtained following the General Procedure, as a white oil (17.4 mg, 0.122 mmol, 61%). **^{1}H NMR (501 MHz, $CDCl_3$)** δ 3.86 – 3.78 (m, 2H), 1.93 – 1.75 (m, 4H), 1.60 – 1.49 (m, 3H), 0.92 – 0.86 (m, 9H). **^{13}C NMR (126 MHz, $CDCl_3$)** δ 87.6, 68.1, 34.2, 31.1, 29.3, 27.0, 17.9, 17.3, 8.1. **HRMS m/z (ESI):** calculated for $C_9H_{18}O$. $[M+H]^+$: 143.1430, found 143.1432.

GC: 25.0 m Ivadex-1, injection temperature: 60 °C, 0.5°C/min, 70°C, iso 5 min, 20 °C/min, 220°C, 0.5 bar H_2. t_{R1} = 13.3 min (minor), t_{R2} = 13.7 min (major). **e.r.:** 95:5. $[\alpha]_D^{25}$ = –13.7 (*c* = 0.7, $CHCl_3$).

(*S*)-2-(tert-butyl)-2-ethyltetrahydrofuran (3l)

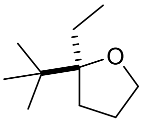

Obtained following the General Procedure, as a white oil (17.2 mg, 0.110 mmol, 55%). **^{1}H NMR (501 MHz, $CDCl_3$)** δ 3.90 (td, *J* = 7.7, 5.5 Hz, 1H), 3.79 (q, *J* = 7.5 Hz, 1H), 1.97 – 1.80 (m, 3H), 1.73 (m, *J* = 14.7, 7.4 Hz, 1H), 1.66 – 1.63 (m, 1H), 1.45 (m, *J* = 14.5, 7.3 Hz, 1H), 0.90 (d, *J* = 14.0 Hz, 12H). **^{13}C NMR (126 MHz, $CDCl_3$)** δ 90.0, 70.2, 38.9, 29.7, 28.4, 27.8, 26.1, 8.9. **HRMS m/z (ESI):** calculated for $C_{10}H_{20}O$. $[M+H]^+$: 157.1587, found 157.1588. $[\alpha]_D^{25}$ = –9.3 (*c* = 0.31, CH_2Cl_2)].

GC: 25.0 m Ivadex-1, injection temperature: 70 °C, 0.5 °C/min, 80 °C, 20 °C/min, 220°C, 0.5 bar H_2. t_{R1} = 14.4 min (minor), t_{R2} = 14.8 min (major). **e.r.:** 87.5:12.5. $[\alpha]_D^{25}$ = –3.6 (*c* = 1.0, $CHCl_3$).

(*S*)-2-ethyl-2-phenyltetrahydrofuran (3m)

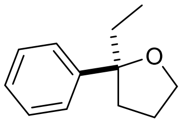

Obtained following the General Procedure, as a white oil (27.5 mg, 0.156 mmol, 78%). **^{1}H NMR (501 MHz, $CDCl_3$)** δ 7.41 – 7.27 (m, 4H), 7.25 – 7.17 (m, 1H), 3.93 (m, *J* = 43.2, 8.0, 6.1 Hz, 2H), 2.18 (m, *J* = 12.6, 8.0, 4.9 Hz, 1H), 2.04 (dt, *J* = 12.1, 8.1 Hz, 1H), 1.93 (m, *J* = 18.0, 8.3, 7.4, 5.4 Hz, 1H), 1.88 – 1.72 (m, 3H), 0.76 (t, *J* = 7.4 Hz, 3H). **^{13}C NMR (126 MHz, $CDCl_3$)** δ 146.7, 128.0, 126.3, 125.5, 87.3, 67.6, 37.9, 35.2, 25.8, 8.9. **HRMS m/z (ESI):** calculated for $C_{12}H_{16}O$. $[M+H]^+$: 177.1274, found 177.1277.

HPLC: Chirapak OJ-3. Heptane/Ethanol = 99.5:0.5, flow rate = 1.0 mL/min, UV-VIS detection at λ = 220 nm, t_{R1} = 4.4 min (major), t_{R2} = 4.8 min (minor). **e.r.:** 80:20. $[\alpha]_D^{25}$ = –4.0 (*c* = 0.2, $CHCl_3$).

(*S*)-2-benzyl-2-ethyltetrahydrofuran (3n)

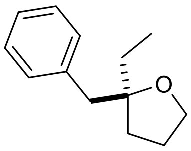

Obtained following the General Procedure, as a white oil (35.8 mg, 0.188 mmol, 94%). **^{1}H NMR (501 MHz, $CDCl_3$)** δ 7.27 – 7.16 (m, 5H), 3.82 – 3.64 (m, 2H), 2.75 (d, *J* = 3.4 Hz, 2H), 1.79 – 1.63 (m, 3H), 1.61 – 1.57 (m, 1H), 1.52 – 1.45 (m, 2H), 0.92 (t, *J* = 7.5 Hz, 3H). **^{13}C NMR (126 MHz, $CDCl_3$)** δ 138.7, 130.7, 128.0, 126.1, 85.6, 67.9, 44.5, 33.7, 32.0, 26.4, 8.8. **HRMS m/z (ESI):** calculated for $C_{13}H_{18}O$. $[M+H]^+$: 191.1430, found 191.1432.

GC: 25.0 m Ivadex-1, injection temperature: 105 °C, iso 50 min, 20°C/min, 220°C, 0.5 bar H_2. t_{R1} = 37.6 min (minor), t_{R2} = 38.3 min (major). **e.r.:** 18:82. $[\alpha]_D^{25}$ = 1.0 (*c* = 0.4, $CHCl_3$).

(*R*)-2-ethyl-2-phenethyltetrahydrofuran (3o)

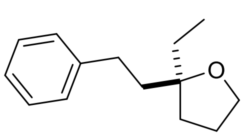

Obtained following the General Procedure, as a white oil (40.2 mg, 0.186 mmol, 93%). **^{1}H NMR (501 MHz, $CDCl_3$)** δ 7.23 – 7.07 (m, 5H), 3.78 (t, *J* = 6.7 Hz, 2H), 2.62 – 2.50 (m, 2H), 1.84 (p, *J* = 7.0 Hz, 2H), 1.75 – 1.62 (m, 4H), 1.57 – 1.46 (m, 2H), 0.85 (t, *J* = 7.5 Hz,

3H). **^{13}C NMR (126 MHz, $CDCl_3$)** δ 142.9, 128.4, 128.3, 125.7, 84.9, 67.5, 40.1, 34.6, 31.0, 30.7, 26.3, 8.8. **HRMS m/z (CI):** calculated for $C_{14}H_{20}O$. $[M+H]^+$: 205.1587, found 205.1585.

HPLC: Chirapak IE-3R. Acetonitrile/Water = 50:50, flow rate = 0.5 mL/min, UV-VIS detection at λ = 220 nm, t_{R1} = 21.7 min (major), t_{R2} = 25.6 min (minor). **e.r.:** 86:14. $[\alpha]_D^{25}$ = 8.7 (c = 0.7, $CHCl_3$).

(*S*)-2-(2,3-dihydro-1H-inden-2-yl)-2-ethyltetrahydrofuran (3p)

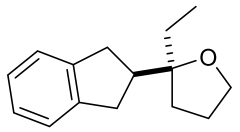

Obtained following the General Procedure, as a white oil (4.6 mg, 0.027 mmol, 27%). **^{1}H NMR (501 MHz, $CDCl_3$)** δ 7.25 – 7.07 (m, 4H), 3.89 (qt, J = 8.4, 4.2 Hz, 2H), 2.99 – 2.79 (m, 5H), 2.00 – 1.83 (m, 3H), 1.79 – 1.72 (m, 1H), 1.60 (m, J = 7.4, 3.7 Hz, 2H), 0.95 (t, J = 7.5 Hz, 3H). **^{13}C NMR (126 MHz, $CDCl_3$)** δ 143.6, 143.3, 126.2, 126.1, 124.5, 124.4, 86.7, 68.4, 46.7, 34.8, 34.3, 32.1, 31.4, 26.9, 8.8. **HRMS m/z (EI):** calculated for $C_{15}H_{20}O$: 216.1509, found 216.1509.

HPLC: Chirapak IE-3R. Acetonitrile/Water = 50:50, flow rate = 0.5 mL/min, UV-VIS detection at λ = 220 nm, t_{R1} = 29.5 min (major), t_{R2} = 39.9 min (minor). **e.r.:** 94:6. $[\alpha]_D^{25}$ = 10.0 (c = 1.4, $CHCl_3$).

(*S*)-3-(2-ethyltetrahydrofuran-2-yl)propyl acetate (3q)

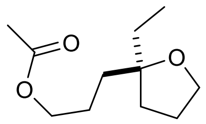

Obtained following the General Procedure, as a white oil (34.9 mg, 0.174 mmol, 87%). **^{1}H NMR (501 MHz, $CDCl_3$)** δ 4.06 (td, J = 6.7, 1.7 Hz, 2H), 3.79 (t, J = 6.7 Hz, 2H), 2.04 (s, 3H), 1.92 – 1.82 (m, 2H), 1.70 – 1.62 (m, 4H), 1.57 – 1.44 (m, 4H), 0.86 (t, J = 7.5 Hz, 3H). ^{13}C NMR (126 **MHz, $CDCl_3$)** δ 171.3, 84.8, 67.6, 65.2, 34.7, 34.3, 31.2, 26.4, 23.7, 21.1, 8.8.

HPLC: Chirapak IG-3R. Acetonitrile/Water = 40:60, flow rate = 1.0 mL/min, UV-VIS detection at λ = 220 nm, t_{R1} = 4.0 min (major), t_{R2} = 4.8 min (minor). **e.r.:** 88:12. $[\alpha]_D^{25}$ = 4.5 (c = 0.3, $CHCl_3$).

(*S*) -3-(2-ethyltetrahydrofuran-2-yl)propyl benzofuran-6-carboxylate (3r)

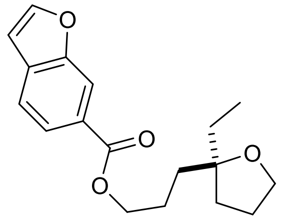

Obtained following the General Procedure, as a white oil (59.3 mg, 0.196 mmol, 98%). **^{1}H NMR (501 MHz, $CDCl_3$)** δ 8.20 (s, 1H), 7.95 (dd, J = 8.1, 1.4 Hz, 1H), 7.76 (d, J = 2.2 Hz, 1H), 7.63 (d, J = 8.1 Hz, 1H), 6.82 (dd, J = 2.2, 1.0 Hz, 1H), 4.35 (t, J = 6.6 Hz, 2H), 3.82 (t, J = 6.7 Hz, 2H), 1.90 (p, J = 7.1 Hz, 2H), 1.86 – 1.76 (m, 2H), 1.75 – 1.67 (m, 2H), 1.66 – 1.50 (m, 4H), 0.90 (t, J = 7.5 Hz, 3H). **^{13}C NMR (126 MHz, $CDCl_3$)** δ 166.9, 154.6, 147.9, 131.8, 126.8, 124.3, 120.9, 113.2, 106.9, 84.9, 67.6, 65.7, 34.7, 34.4, 31.1, 26.4, 24.0, 8.9.

HPLC: Chirapak OJ-3. Heptane/Isopropanol = 95:5, flow rate = 1 mL/min, UV-VIS detection at λ = 254 nm, t_{R1} = 5.4 min (major), t_{R2} = 6.3 min (minor). **e.r.:** 85:15. $[\alpha]_D^{25}$ = 1.9 (c = 0.6, $CHCl_3$).

Synthesis and Characterization of Catalysts lactone 4

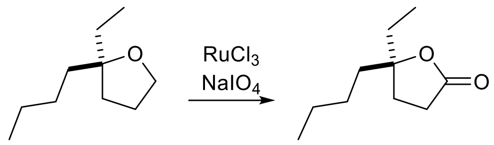

3g 77%, 93.5:6.5 er   **4** 57%, 92.5:7.5 er

To the solution of **3g** (25 mg, 0.16 mol, 1.0 equiv.) in ethylacetate (1.6 mL) was added $NaIO_4$ (273.7 mg 1.28 mmol, 8.0 equiv.) and $RuCl_3$ (11.6 mg, 0.056 mmol, 0.35 equiv.) in water (1.6 mL) at room temperature. The reaction was quenched after 2 hours by the addition of sodium thiosulfate aqueous solution. The organic phase was separated, and the aqueous phase was washed with MTBE twice. The combined organic layer was washed with brine and dried over Na_2SO_4. The crude mixture was purified by silica gel chromatography with 10-30% MTBE in pentane to afford the desired lactone as a colorless oil. (15.6 mg, 57 %).

(*R*)-5-butyl-5-ethyldihydrofuran-2(3H)-one

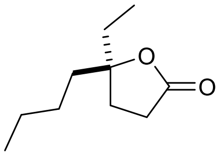

Obtained following the General Procedure, as a white oil (59.3 mg, 0.196 mmol, 98%). **^{1}H NMR (501 MHz, $CDCl_3$)** δ 2.57 (dd, *J* = 9.1, 8.0 Hz, 2H), 2.01 (t, *J* = 8.6 Hz, 2H), 1.73 – 1.59 (m, 4H), 1.38 – 1.28 (m, 4H), 0.92 (q, *J* = 7.6 Hz, 6H). **^{13}C NMR (126 MHz, $CDCl_3$)** δ 177.2, 89.5, 38.1, 31.5, 30.5, 29.3, 25.7, 23.1, 14.1, 8.0.

GC: 25.0 m Cyclosil B, injection temperature: 80°C, 1°C/min, 160°C, iso 5 min, 20°C/min, 220°C, iso 5 min, 0.5 bar H_2. t_{R1} = 66.0 min (major), t_{R2} = 66.7 min (minor). **e.r.:** 92.5:7.5. $[\alpha]_D^{25}$ = 2.8 (*c* = 1.4, $CHCl_3$).

5. Synthesis and Characterization of Catalysts

General procedure for cross-coupling reactions and subsequent MOM-deprotections and iDPi synthesis:

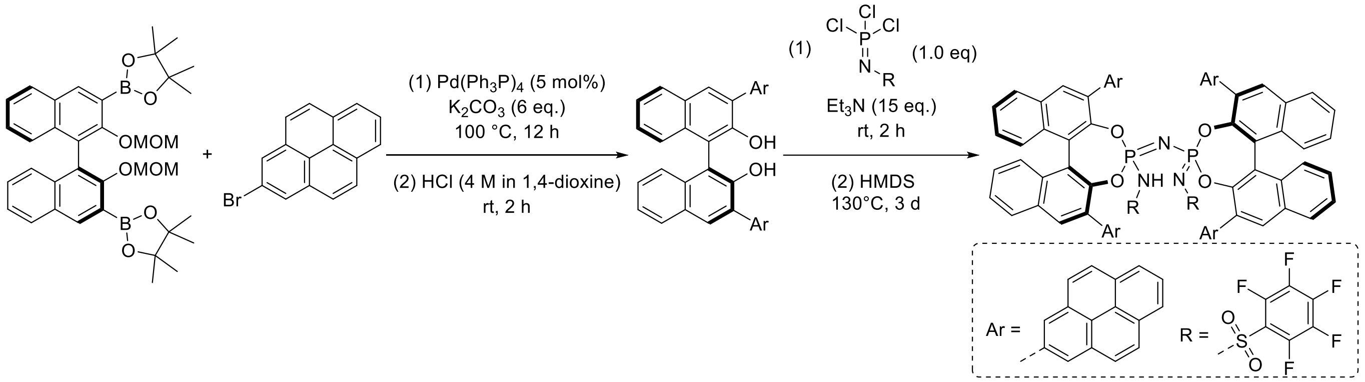

Diol synthesis: To a two-neck round-bottom flask with a condenser was added (S)-2,2'-(2,2'-bis(methoxymethoxy)-[1,1'-binaphthalene]-3,3'-diyl)bis(4,4,5,5-tetramethyl-1,3,2-dioxaborolane) (500 mg, 1.0 eq.), 2-bromopyrene, tetrakis(triphenylphosphine)palladium (46.1 mg, 5 mol%) and K_2CO_3 (667 mg, 6 eq.) under argon atmosphere. Degassed toluene (6 mL), ethanol (4 mL) and water (2 mL) were sequentially added. The mixture was then heated to 100 °C and stirred at that temperature overnight. After cooling the reaction mixture to rt, the organic layer was separated and the aqueous phase was extracted with EtOAc (3x10 mL). The organic phase was combined and filtered through a thin layer of silica gel using a Büchner funnel and the silica gel layer was washed with some extra EtOAc.

The solvent was removed under reduced pressure and the crude MOM-protected diol was obtained. Subsequently, the crude product was dissolved in a small amount of DCM (2 mL). A solution of HCl (4 M in 1,4-dioxane, 6 mL) was added at rt and the mixture was stirred at rt. for 2 h. The solvent was removed under reduced pressure and the crude was purified by column chromatography to afford the corresponding diol (Pentane:EtOAc = 6:1).

IDPi synthesis: In a flame-dried flask under Ar, 3,3'-di(pyren-2-yl)-[1,1'-binaphthalene]-2,2'-diol (200 mg, 1.0 eq.) and ((perfluorophenyl)sulfonyl)phosphorimidoyl trichloride (116 mg, 1.0 eq.) were dissolved in toluene (1 mL), then Et_3N (460 mg, 15.0 eq.) was added and the yellow suspension was stirred at r.t for 3 hours. A freshly titrated solution of HMDS (24.5 mg, 0.50 eq.) was added to the reaction mixture, which was stirred at r.t. for 10 min, then heated to 130 °C for 3 days. The reaction mixture was cooled to room temperature, diluted with CH_2Cl_2 (10 mL), washed with 1.0 M HCl (10 mL) and brine (10 mL), dried (Na_2SO_4), filtered (filter paper) and concentrated under reduced pressure. Purification by column chromatography (Pentane:EtOA:DCM = 6:2:2) afforded a yellow solid, which was subjected to acidification. The solid was dissolved in CH_2Cl_2 (2.5 mL) and vigourously stirred with 6.0 M HCl (2.5 mL) at r.t. for 15 min, diluted with CH_2Cl_2 (10 mL) and washed with 6.0 M HCl (2x10 mL). The combined organic layers were then dried under reduced pressure with toluene (2x5 mL) to afford IDPi as a white solid.

(*S*)-3,3'-di(pyren-2-yl)-[1,1'-binaphthalene]-2,2'-diol

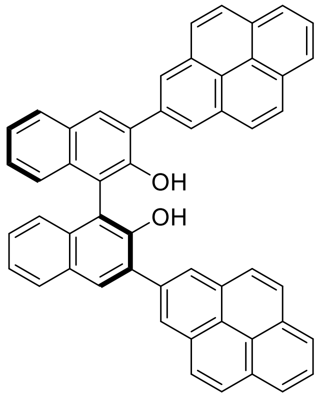

^{1}H NMR (501 MHz, $CDCl_3$) δ 8.57 (s, 4H), 8.28 (s, 2H), 8.22 (d, *J* = 7.7 Hz, 4H), 8.17 – 8.10 (m, 8H), 8.03 (t, *J* = 7.7 Hz, 4H), 7.48 – 7.39 (m, 6H), 5.63 (s, 2H). **^{13}C NMR (126 MHz, $CDCl_3$)** δ 150.51, 135.2, 133.4, 132.5, 131.5, 131.4, 131.1, 129.8, 128.8, 128.0, 127.7, 127.6, 126.2, 125.3, 124.7, 124.6, 124.6, 124.2, 113.0.

(*S*)- imidodiphosphorimidates (2f)

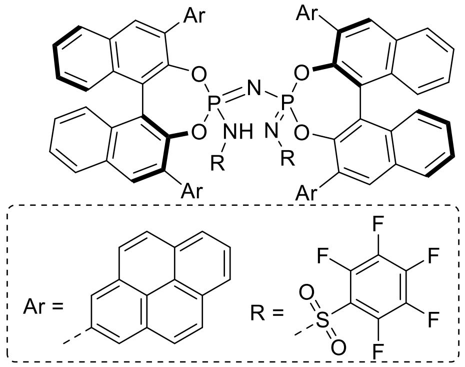

^{1}H NMR (501 MHz, $CDCl_3$) δ 8.28 (s, 2H), 8.18 (dd, *J* = 14.7, 8.4 Hz, 4H), 8.10 – 8.05 (m, 2H), 7.98 (s, 4H), 7.93 (t, *J* = 7.6 Hz, 6H), 7.87 – 7.75 (m, 16H), 7.71 (m, *J* = 8.1, 6.3, 1.6 Hz, 2H), 7.62 (dd, *J* = 8.4, 2.8 Hz, 6H), 7.54 – 7.46 (m, 4H), 7.20 (s, 2H), 7.13 (s, 4H), 6.83 (d, *J* = 9.1 Hz, 4H). **^{13}C NMR (126 MHz, $CDCl_3$)** δ 145.0, 143.6, 134.4, 133.6, 133.5, 132.8, 132.5, 132.4, 132.21, 132.1, 132.0, 131.9, 131.2, 131.1, 131.1, 130.2, 130.1, 128.9, 127.9, 127.8, 127.5, 127.3, 127.2, 127.1, 126.8, 126.6, 126.5, 126.3, 126.1, 125.7, 125.3, 125.0, 124.5, 124.3, 123.8, 123.7, 123.0, 122.4, 53.6. **^{19}F NMR (471 MHz, $CDCl_3$)** δ –136.9, –146.4, –159.8. **^{31}P NMR (203 MHz, $CDCl_3$)** δ –16.0. **HRMS m/z (ESI):** calculated for $C_{128}H_{52}F_{10}N_1O_1P_2S_2$ [M–H]$^-$: 1934.2811, found 1934.2818.

6. Mechanistic Study

6.1 Deuteration NMR Study

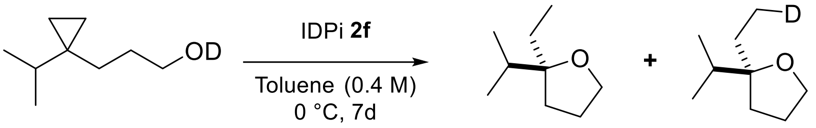

1k' **3k** **3k'**

The product **3k'** is the only deuterated product, based on proton background in reaction mixture, this reaction still obtained 70% non-deuterated product **3k**.

Integral ratio from ^{1}H line shape simulation: Non-deuterated product **3k**: 65 %, deuterated product **3k'**: 35 %.

Integral ratio from ^{13}C: Non-deuterated product **3k**: ~70 %, deuterated product **3k'**: ~30 %.

Atom	δ (ppm)	J	HSQC	mb-HMQC	COSY	NOESY
C1	68.24		1	2, 3a, 3b		
H1	3.797	m	1	2, 3, 4	2, 3a, 3b	8, 9
C2	27.09		2	1, 3a, 3b		
H2	1.836	m (o.l.)	2	1	1, 3a, 3b	
C3	31.26		3a, 3b	1, 5, 7		
H3a	1.763	m (o.l.)	3	1, 2, 4, 5, 7	1, 2, 3b	8, 9
H3b	1.543	m	3	1, 2, 5	1, 2, 3a	8, 9
C4	87.72			1, 3a, 5, 6, 7, 8, 9		
C5	29.37		5	3a, 3b, 6, 7		
H5	1.497	t 7.4(6), 0.8(?)	5	3, 4, 6, 7	6	
C6	7.92	t 19.2(D6)	6	5		
H6	0.850	t 7.4(5), t 1.9(D6)	6	4, 5	5	
D6	0.818	s				
C7	34.35		7	3a, 5, 8, 9		
H7	1.860	m (o.l.)	7	3, 4, 5, 8, 9	8, 9	
C8	18.03		8	7, 9		
H8	0.867	d 6.9(7)	8	4, 7, 9	7	1, 3a, 3b
C9	17.45		9	7, 8		
H9	0.875	d 6.9(7)	9	4, 7, 8	7	1, 3a, 3b

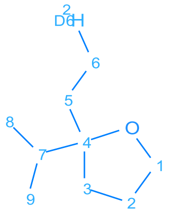

Atom	δ (ppm)	J	HSQC	mb-HMQC	COSY	NOESY
C11	68.24		11	12, 13a, 13b		
H11	3.797	m	11	12, 13, 14	12, 13a, 13b	18, 19
C12	27.09		12	11, 13a, 13b		
H12	1.836	m (o.l.)	12	11	11, 13a, 13b	
C13	31.26		13a, 13b	11, 15, 17		
H13a	1.763	m (o.l.)	13	11, 12, 14, 15, 17	11, 12, 13b	18, 19
H13b	1.543	m	13	11, 12, 15	11, 12, 13a	18, 19
C14	87.72			11, 13a, 15, 16, 17, 18, 19		
C15	29.45		15	13a, 13b, 16, 17		
H15	1.506	q 7.4(16), d 0.7(?)	15	13, 14, 16, 17	16	
C16	8.20		16	15		
H16	0.867	t 7.4(15)	16	14, 15	15	
C17	34.34		17	13a, 15, 18, 19		
H17	1.860	m (o.l.)	17	13, 14, 15, 18, 19	18, 19	
C18	18.03		18	17, 19		
H18	0.867	d 6.9(17)	18	14, 17, 19	17	11, 13a, 13b
C19	17.45		19	17, 18		
H19	0.875	d 6.9(17)	19	14, 17, 18	17	11, 13a, 13b

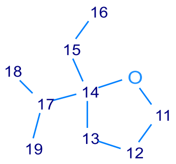

Figure S1: ^{2}H NMR (600 MHz) list of **3k** and **3k'**, with the respective integrals used to estimate the deuterium incorporation.

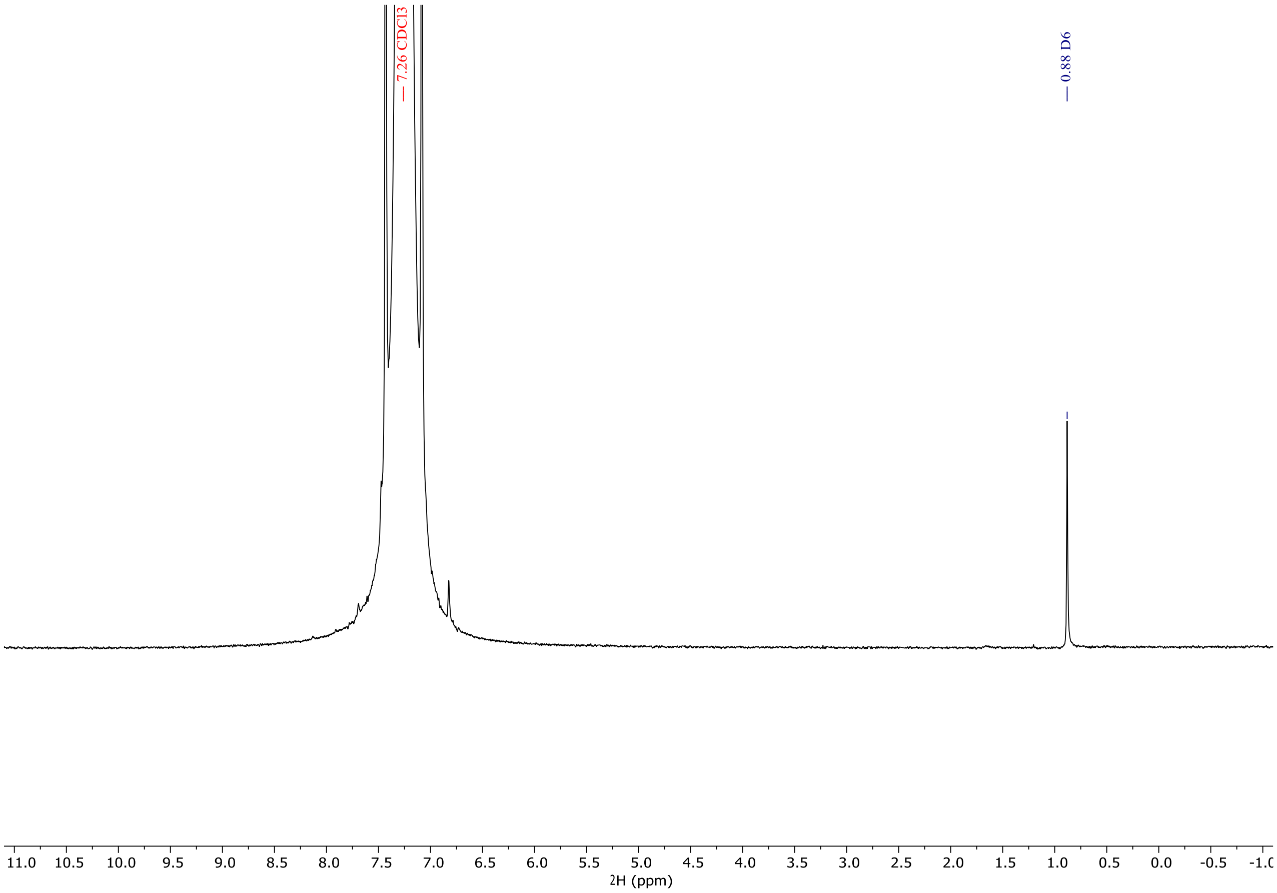

Figure S2: ^{2}H NMR (600 MHz) spectra of **3k** and **3k'** mixture.

Name: M1

From: 0.830 ppm

To: 0.887 ppm

Residual Error:1.63e+09

#	ppm	Hz	Height	Width	L/G	Shape	Area	Total Area %
1	0.881	528.82	2245672.86	0.81	1.17	Generalized Lorentzian	26319295.72	16.35
2	0.879	527.57	1092925.61	0.59	0.24	Generalized Lorentzian	10678869.07	6.63
3	0.8726	523.73	2286755.34	0.8	1.6	Generalized Lorentzian	24721136.41	15.36
4	0.8696	521.96	2234678.5	0.84	1.41	Generalized Lorentzian	25907125.18	16.09
5	0.8666	520.14	2300774.44	0.55	0.6	Generalized Lorentzian	20109035.31	12.49
6	0.8651	519.28	80000	1	0.4	Lorentzian-Gaussian	1105349	0.69
7	0.862	517.39	80000.94	1.02	0.4	Lorentzian-Gaussian	1127469.23	0.7
8	0.8611	516.85	2300774.44	0.8	0.78	Lorentzian-Gaussian	29427553.73	18.28
9	0.8586	515.38	80000	0.92	0.4	Lorentzian-Gaussian	1016921.08	0.63
10	0.8542	512.7	1022970.83	0.6	0.7	Generalized Lorentzian	9555076.59	5.94
11	0.8528	511.88	159426.93	0.98	0.34	Generalized Lorentzian	2565686.69	1.59
12	0.8496	509.93	180466.16	0.88	0.43	Generalized Lorentzian	2558405.21	1.59
13	0.8463	507.95	154144.27	0.88	0.36	Generalized Lorentzian	2206953.17	1.37
14	0.8405	504.47	75537.34	1.09	0.32	Generalized Lorentzian	1350048.34	0.84
15	0.8372	502.48	80299.29	0.98	0.37	Generalized Lorentzian	1278196.6	0.79
16	0.8339	500.52	71888.35	0.91	0.4	Generalized Lorentzian	1060624.34	0.66

Determined ratio of peak integrals: H16: 74%, H6: 26 %

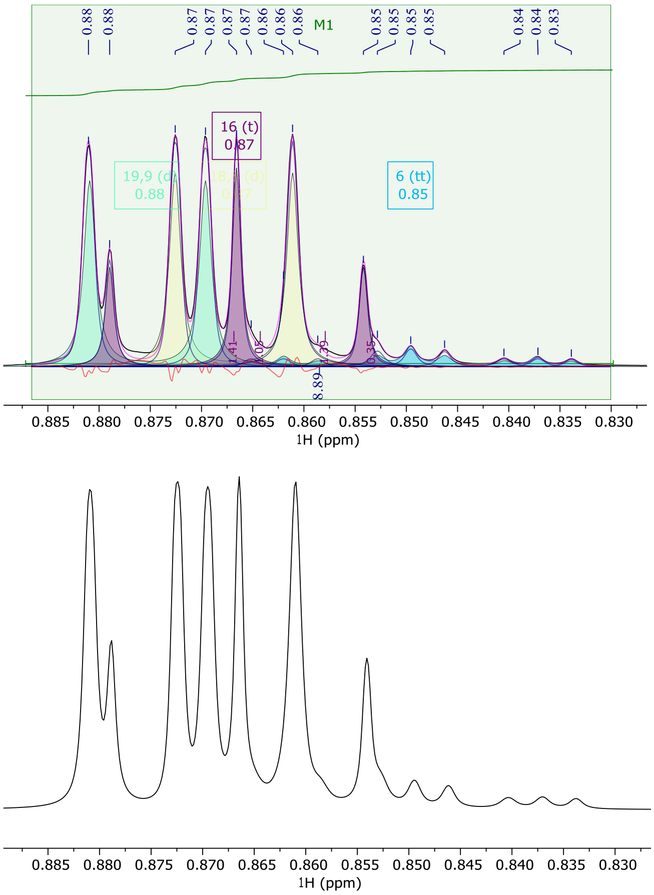

Figure S3: Characteristic peak of ^{1}H NMR (600 MHz) spectra of **3k** and **3k'** mixture.

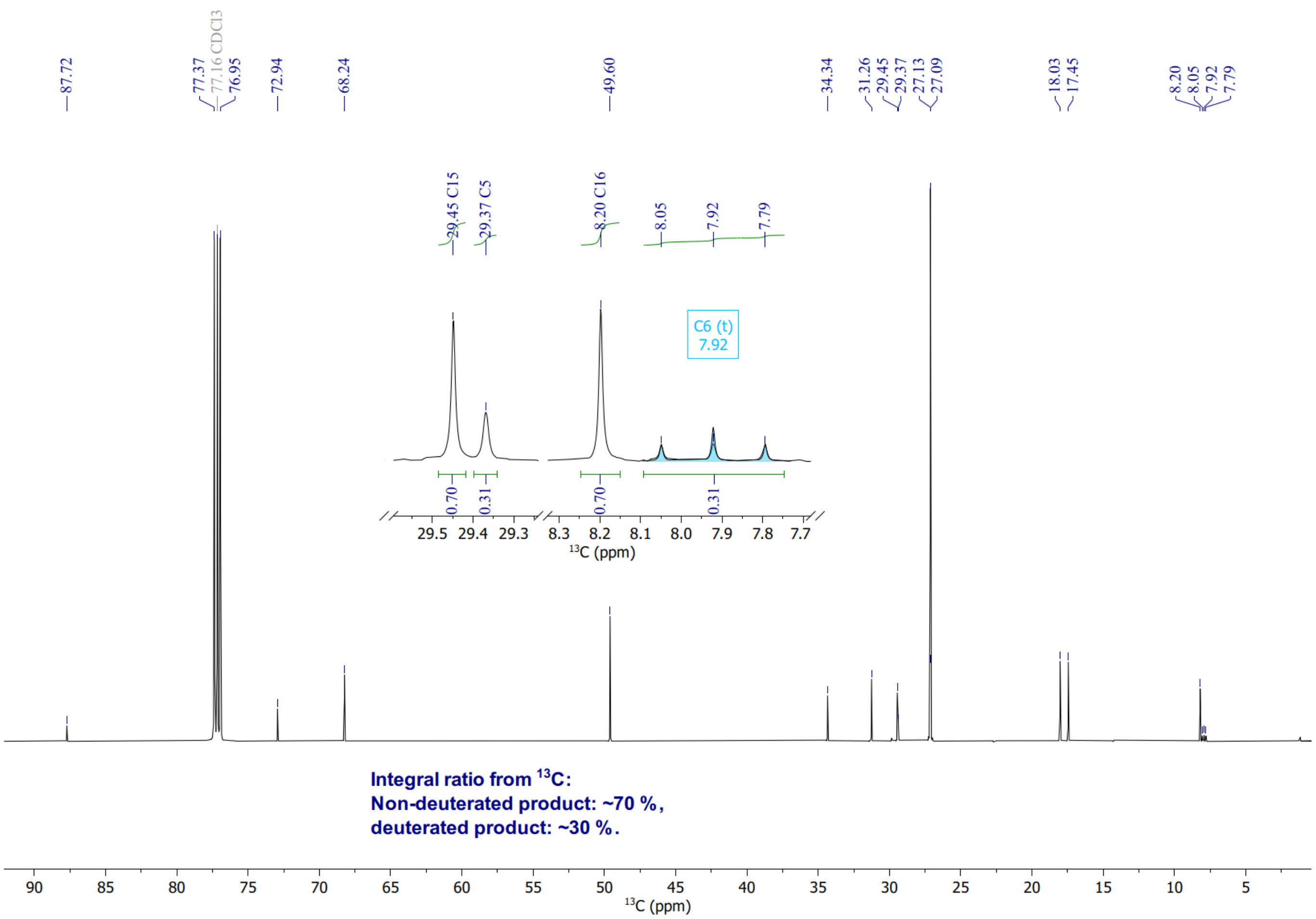

Figure S4: Characteristic peak of ^{13}C NMR (600 MHz) spectra of **3k** and **3k'** mixture.

6.2 Reaction Monitoring

The NMR tube was charged with IDPi **2f** (10.0 µmol, 5 mol%). Dry toluene-d$_8$ (0.4 mL) was added. Then the substrate **1k** (0.2 mmol, 1.0 equiv.) was added dropwise and the reaction was set up at room temperature for ^{1}H NMR measurement.

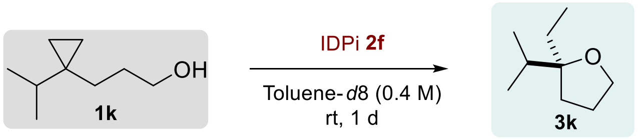

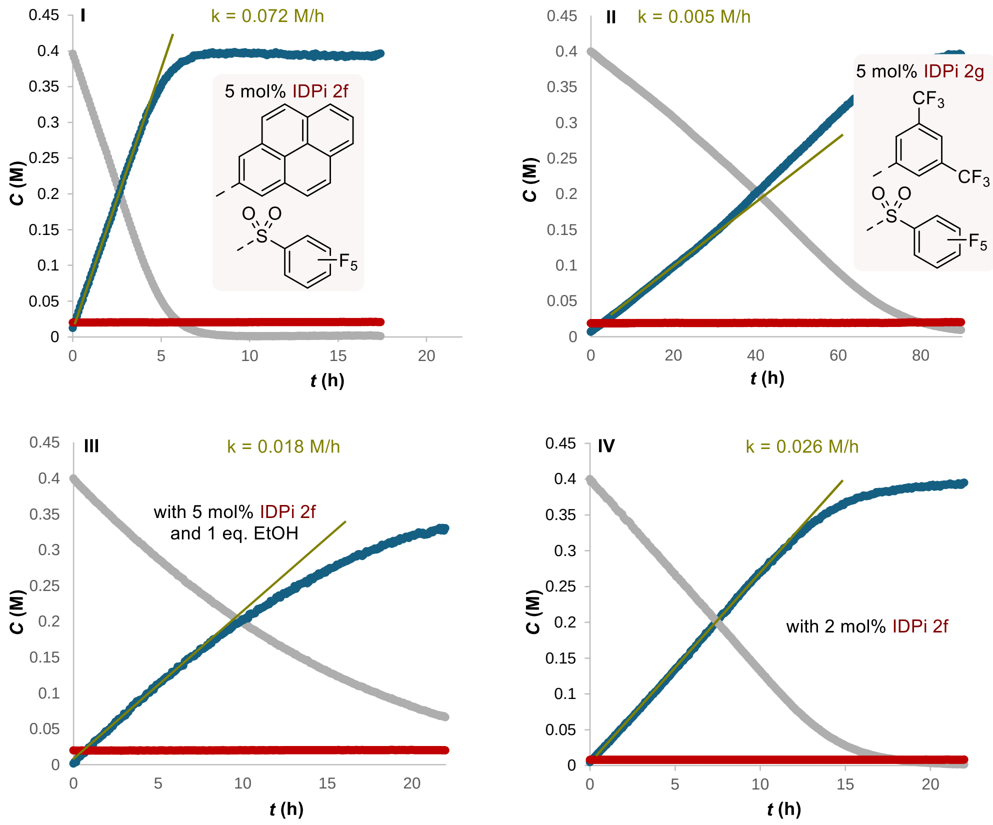

Figure S5. Reaction profile monitored by ^{1}H NMR spectroscopy.

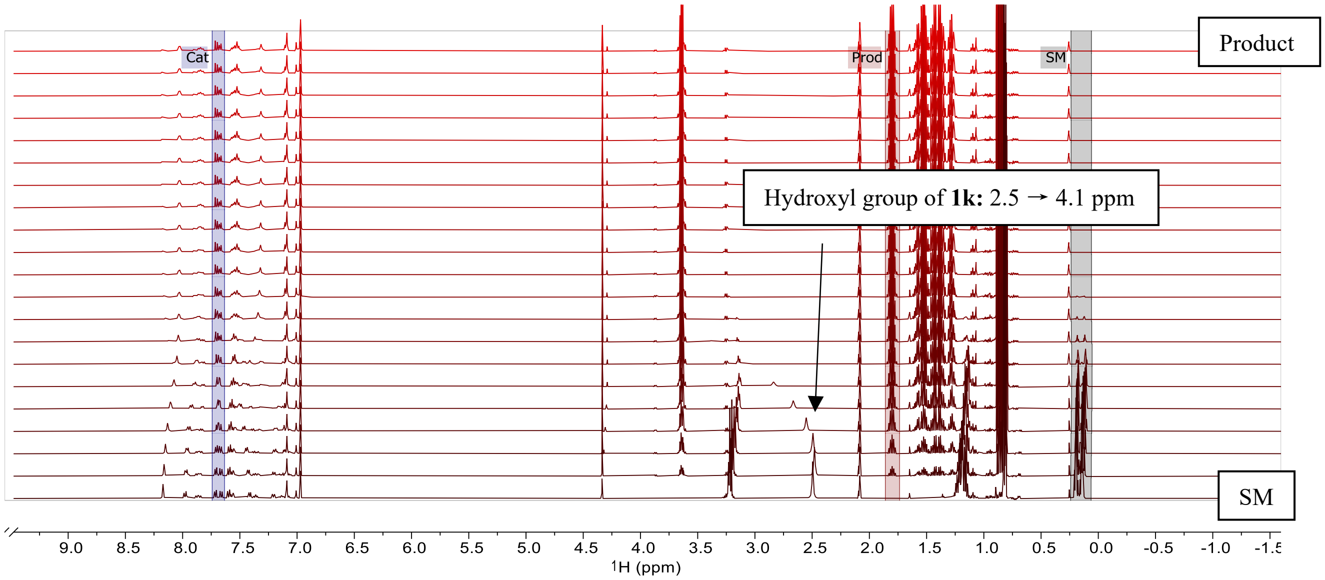

Figure S6. Stacked quantitative ^{1}H NMR spectra of reaction monitoring of **Figure S5I** which showing integration signals of interest.

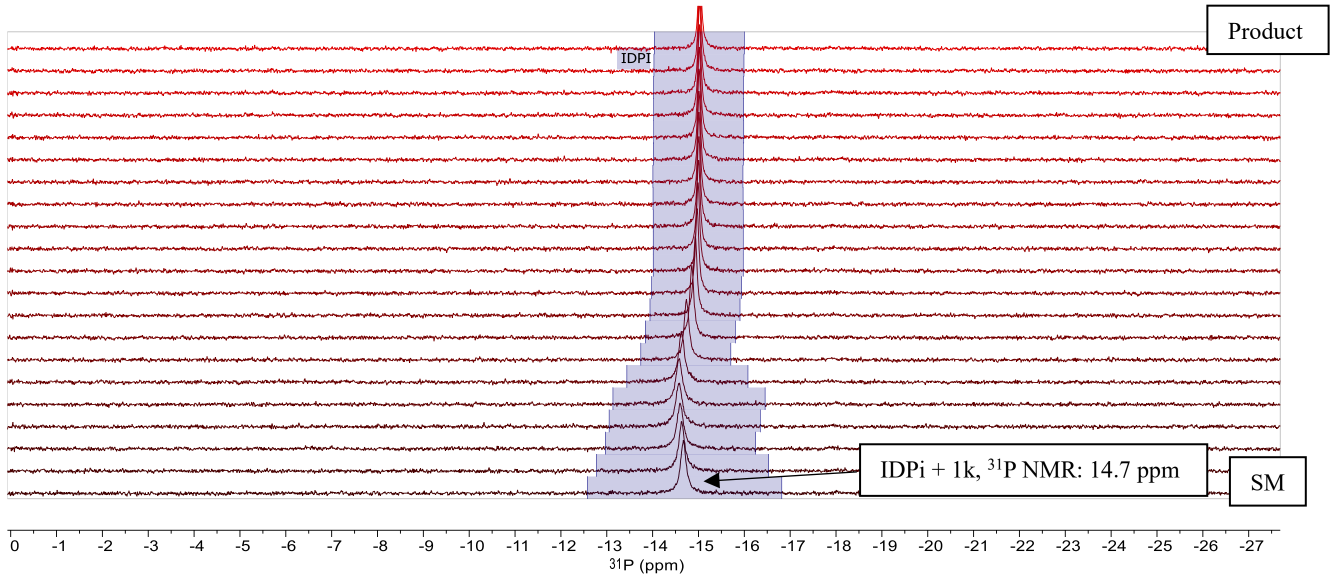

Figure S7. Stacked quantitative ^{31}P NMR spectra of reaction monitoring of **Figure S5I** which showing integration signals of interest.

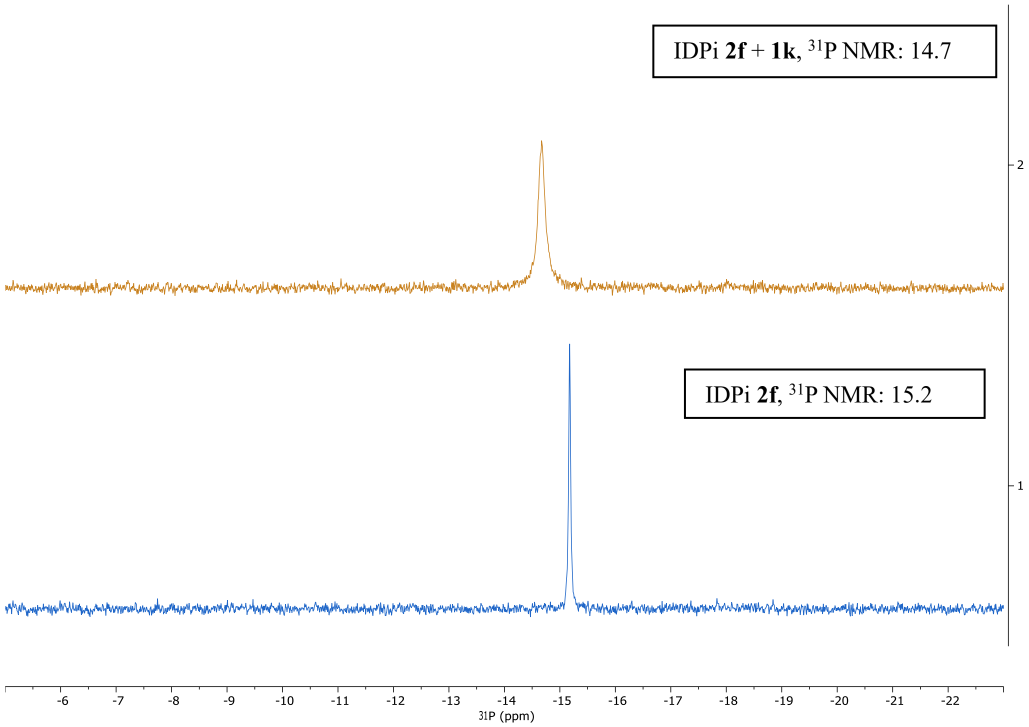

Figure S8. ^{31}P NMR spectra of IDPi **2f** and IDPi **2f** with substrate **1k** in toluene-d_8.

6.3 MS detection

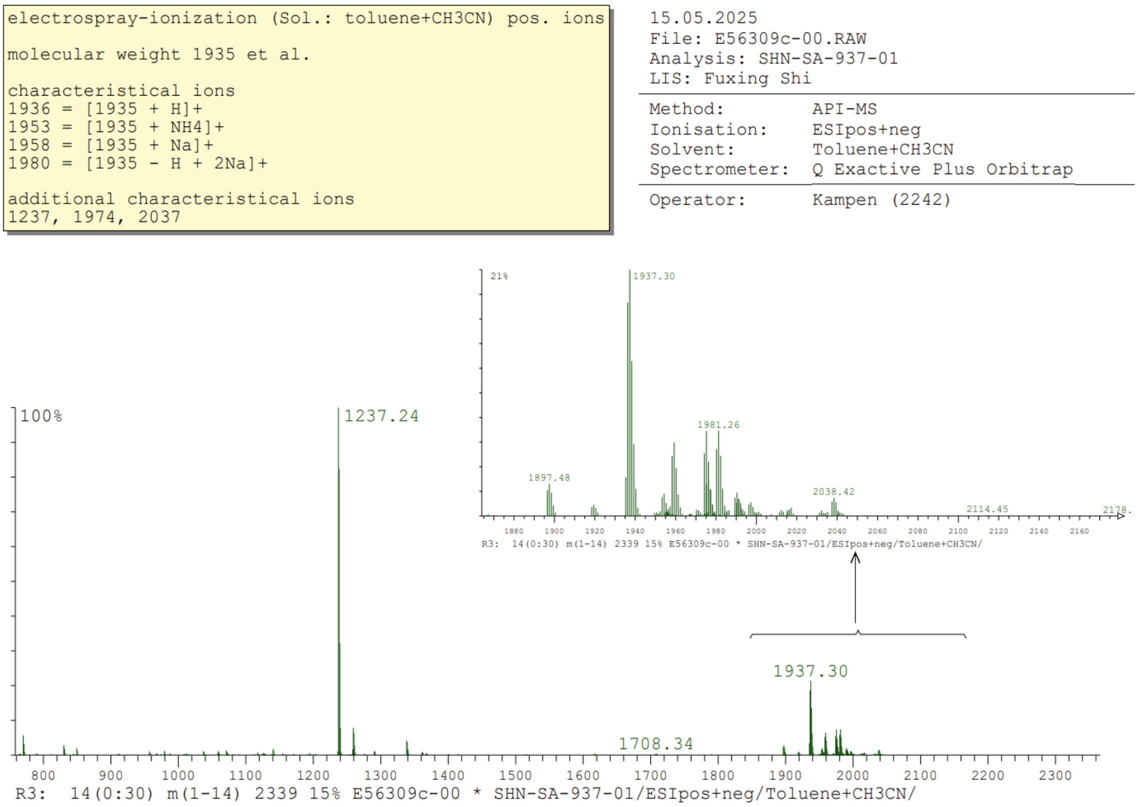

Figure S9. MS spectra of reaction monitoring signals of interest in toluene and CH_3CN solvent, there is no covalent bonding intermediate (MS ≈ 2079.02) detected.

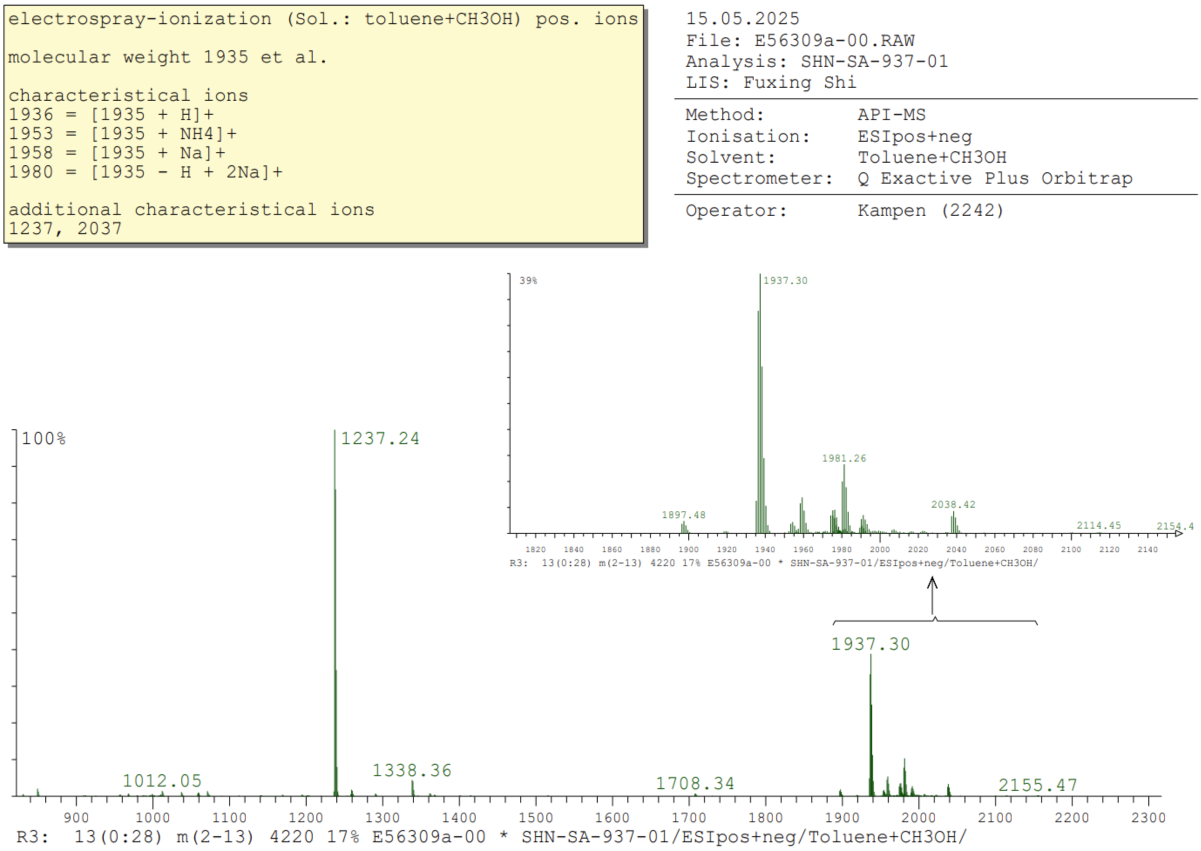

Figure S10. MS spectra of reaction monitoring signals of interest in toluene and CH_3OH solvent, there is no covalent bonding intermediate (MS ≈ 2079.02) detected.

6.4 Procedure of etherification of the corresponding alcohol and ester substrate

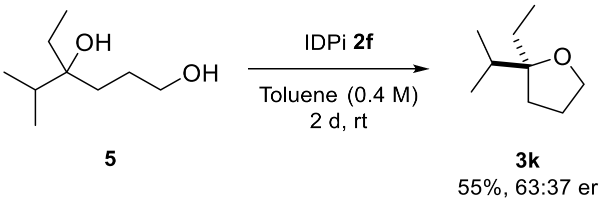

A 5 ml vial was charged with IDPi **2f** (10.0 μmol, 5 mol%) and a magnetic stir bar under an atmosphere of argon. Dry toluene (0.4 mL) was added. The vial was cooled to relevant temperature. The substrate **5** (0.2 mmol, 1.0 equiv.) was added dropwise and the reaction was stirred for 2 days at room temperature.

Afterwards, the reaction mixture was treated with triethylamine (0.04 mmol, 5.6 µL, 0.4 equiv.). After the mixture was stirred for 10 min, the solvent of the reaction mixture was directly removed under reduced pressure. The product was purified by column chromatography on silica gel (pentane: Et_2O = 10:1), then analyzed by chiral HPLC and GC to determine the enantiomeric ratio (er).

2-cyclopropylheptan-2-yl acetate (6)

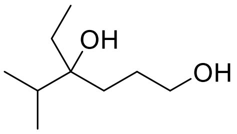

^{1}H NMR (501 MHz, CDCl$_3$) δ 3.66 (td, *J* = 6.1, 3.0 Hz, 2H), 1.84 – 1.48 (m, 9H), 0.93 – 0.84 (m, 9H). **^{13}C NMR (126 MHz, CDCl$_3$)** δ 75.8, 63.6, 34.0, 32.2, 28.3, 26.5, 16.8, 16.7, 7.6.

7. GC and HPLC Traces of the Products

7.1. GC trace

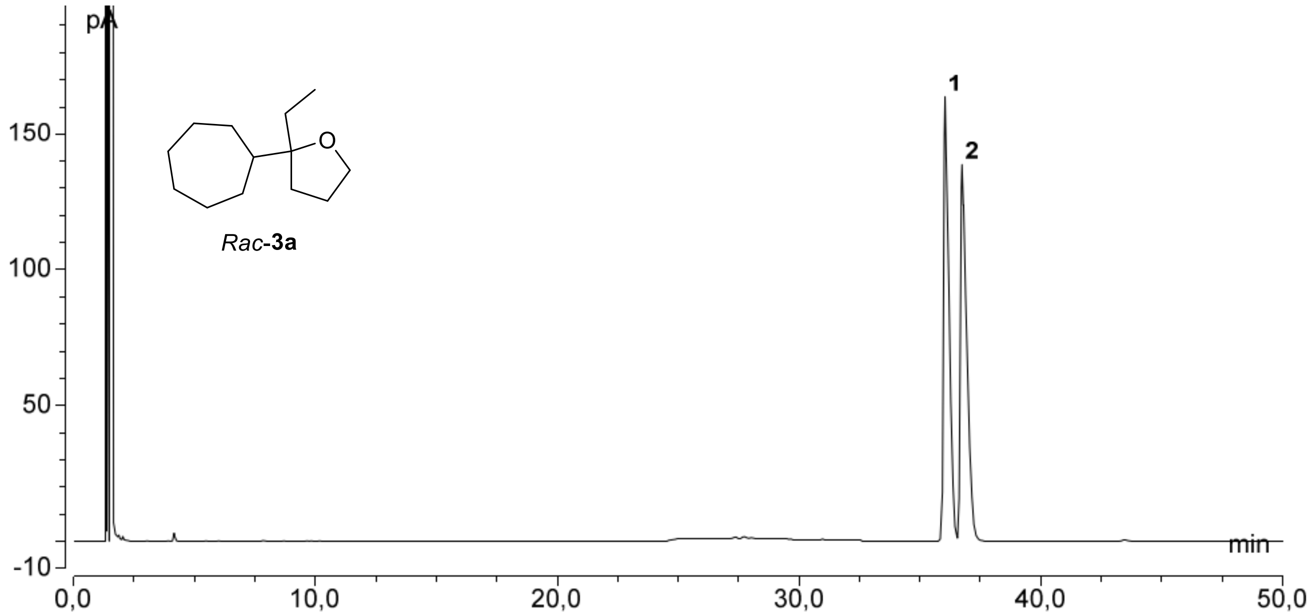

Peak#	Ret. Time (min)	Area%
1	36.04	49.90
2	36.75	50.10
Total		100.00

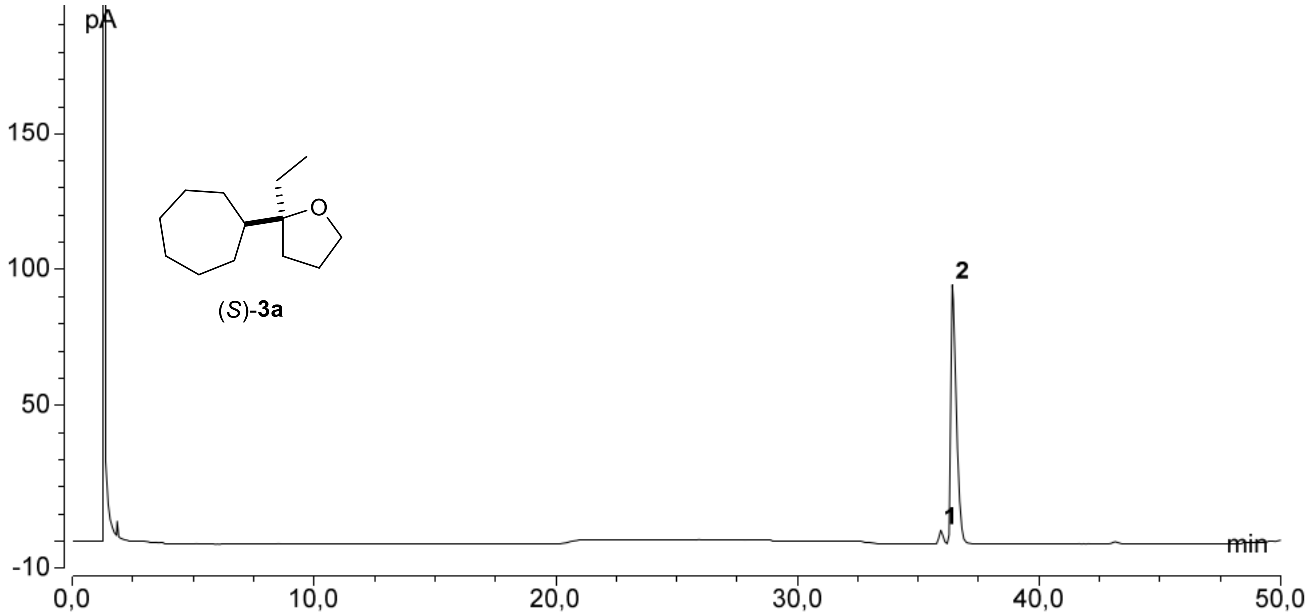

Peak#	Ret. Time (min)	Area%
1	35.93	3.80
2	36.41	96.20
Total		100.00

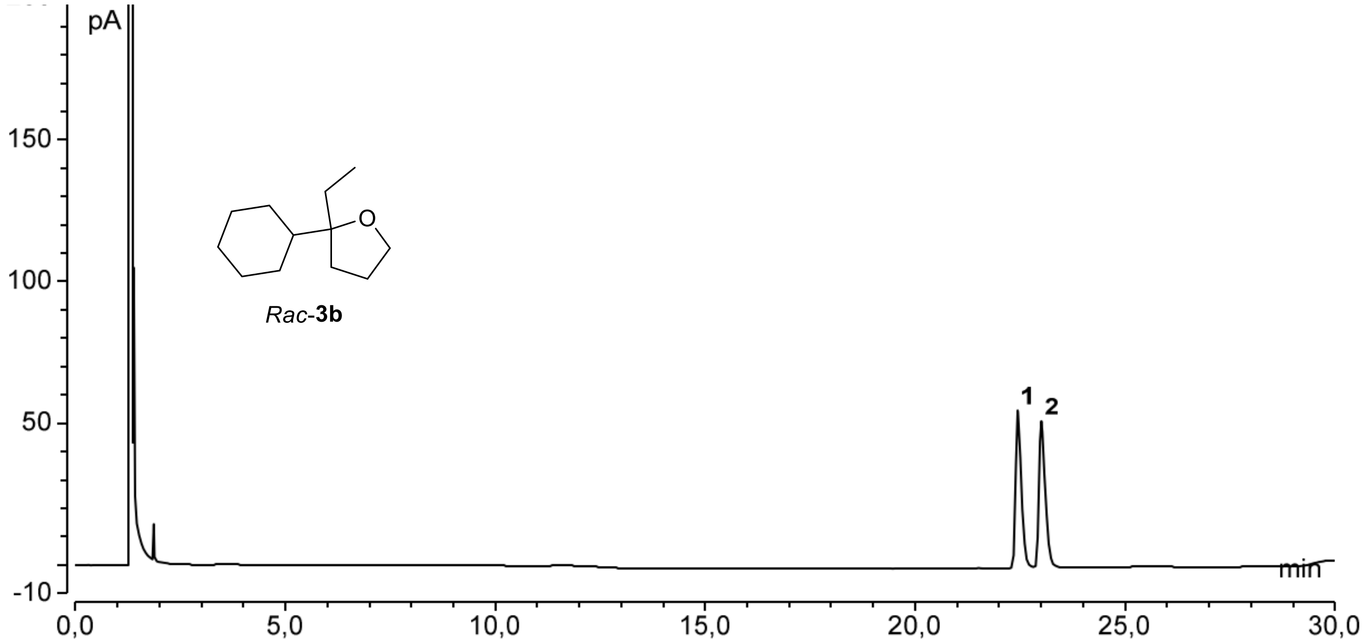

Peak#	Ret. Time (min)	Area%
1	22.44	49.94
2	23.00	50.06
Total		100.00

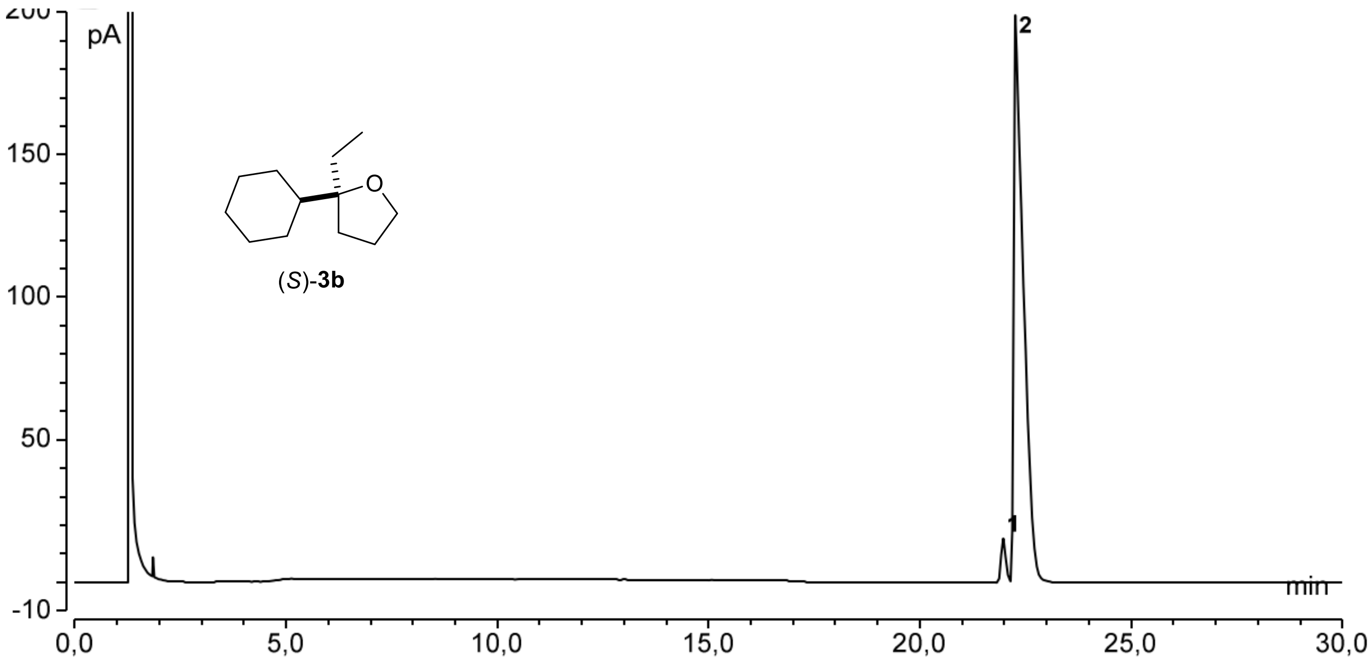

Peak#	Ret. Time (min)	Area%
1	21.97	3.47
2	22.26	96.53
Total		100.00

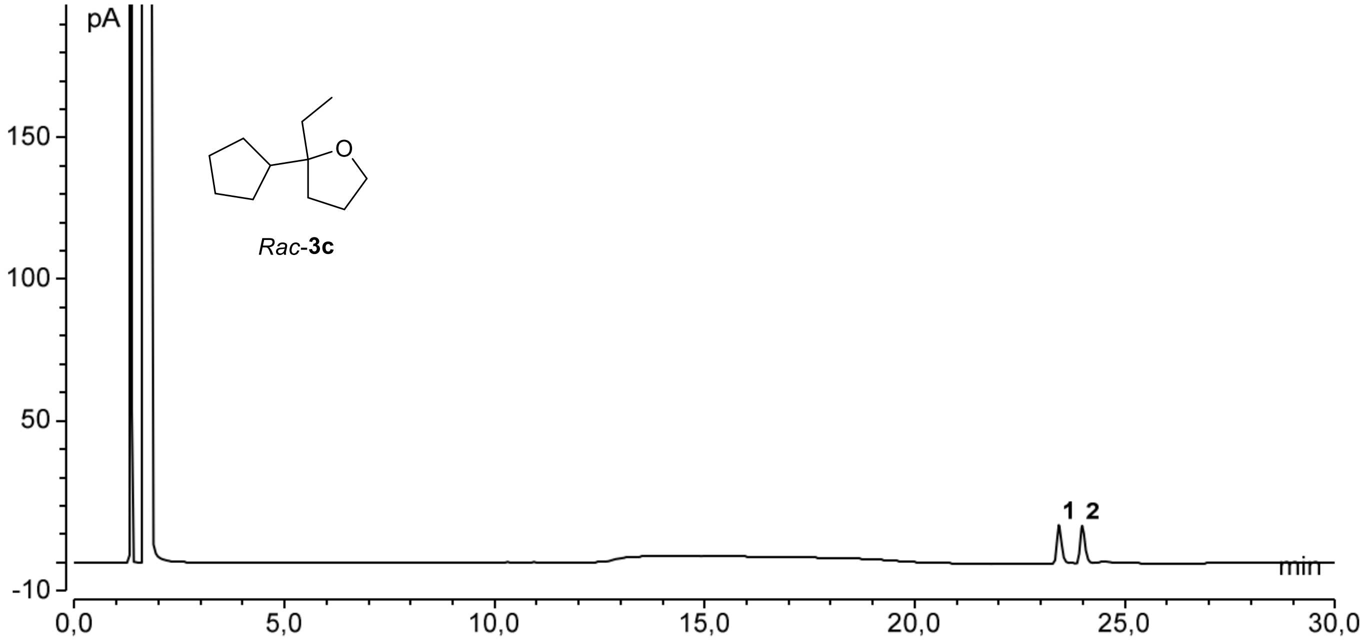

Peak#	Ret. Time (min)	Area%
1	23.43	50.12
2	23.99	49.88
Total		100.00

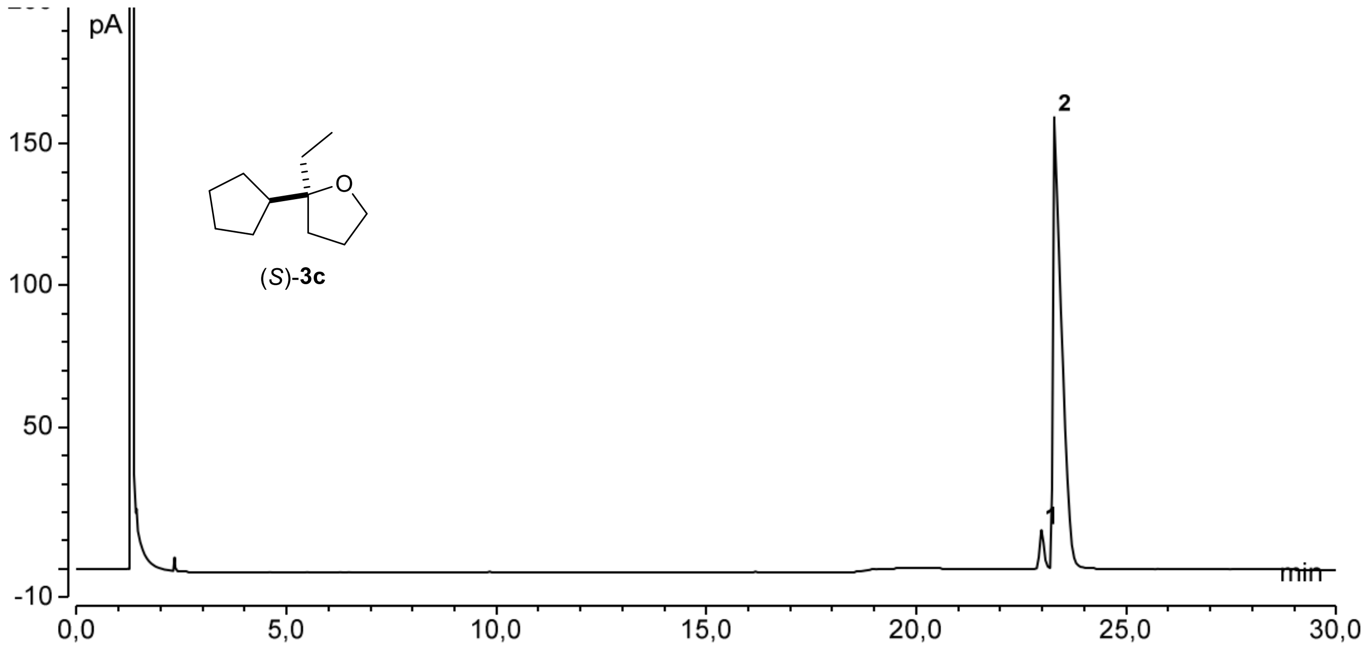

Peak#	Ret. Time (min)	Area%
1	22.98	3.91
2	23.28	96.09
Total		100.00

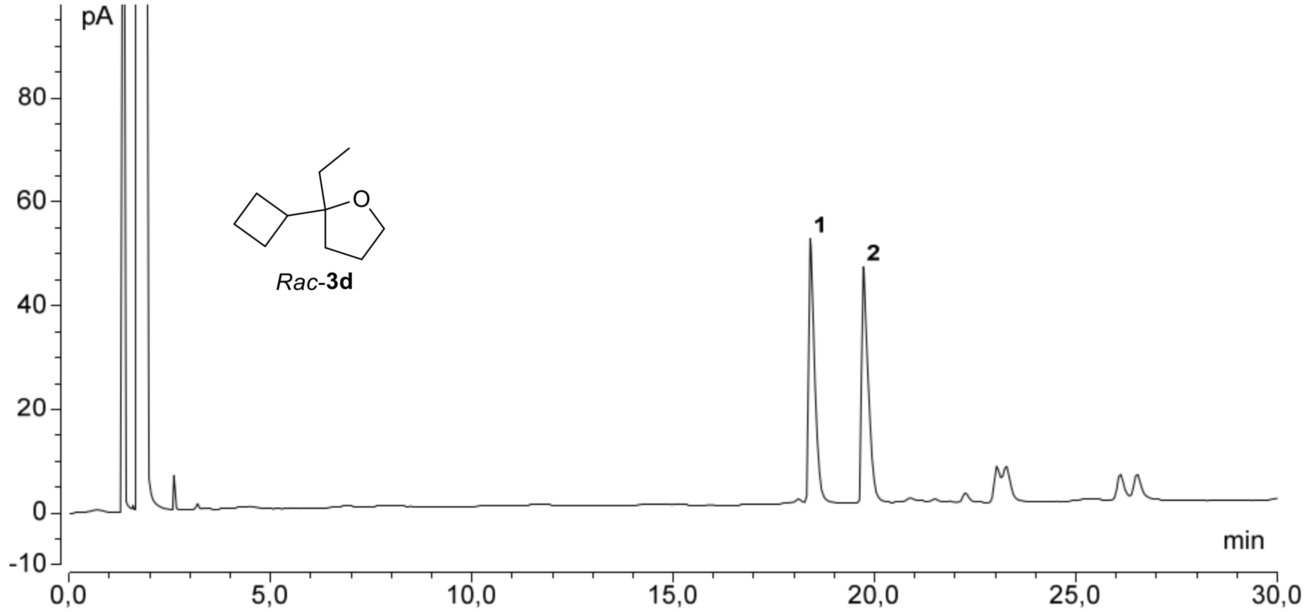

Peak#	Ret. Time (min)	Area%
1	18.40	49.97
2	19.72	50.03
Total		100.00

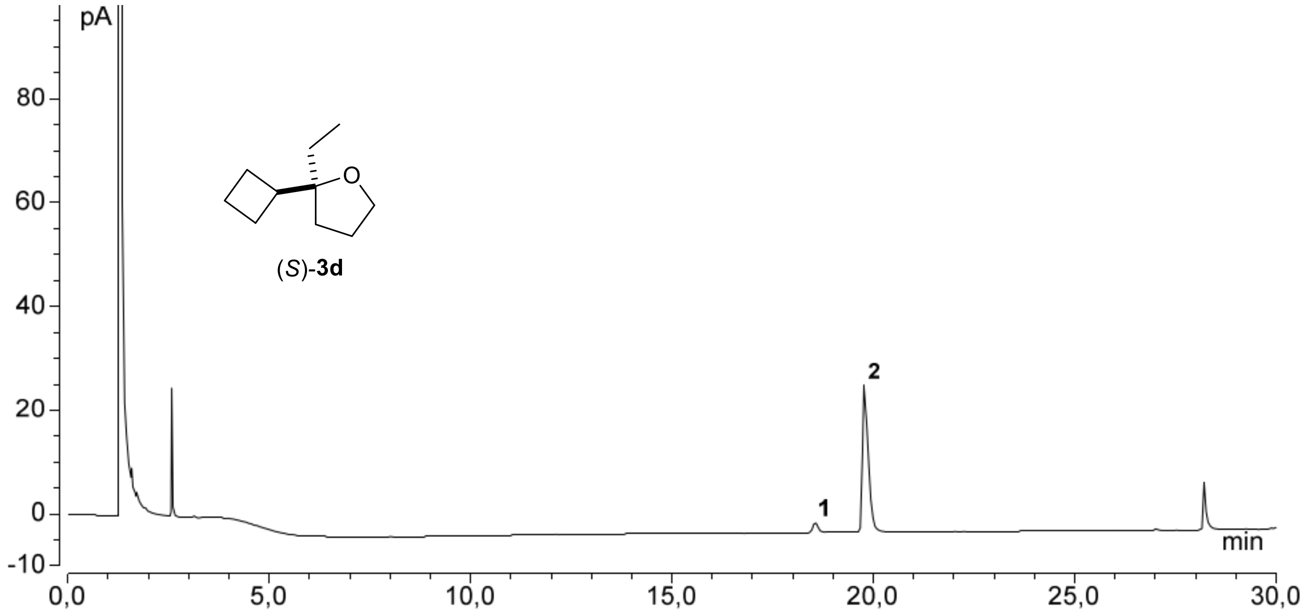

Peak#	Ret. Time (min)	Area%
1	18.55	5.69
2	19.78	94.31
Total		100.00

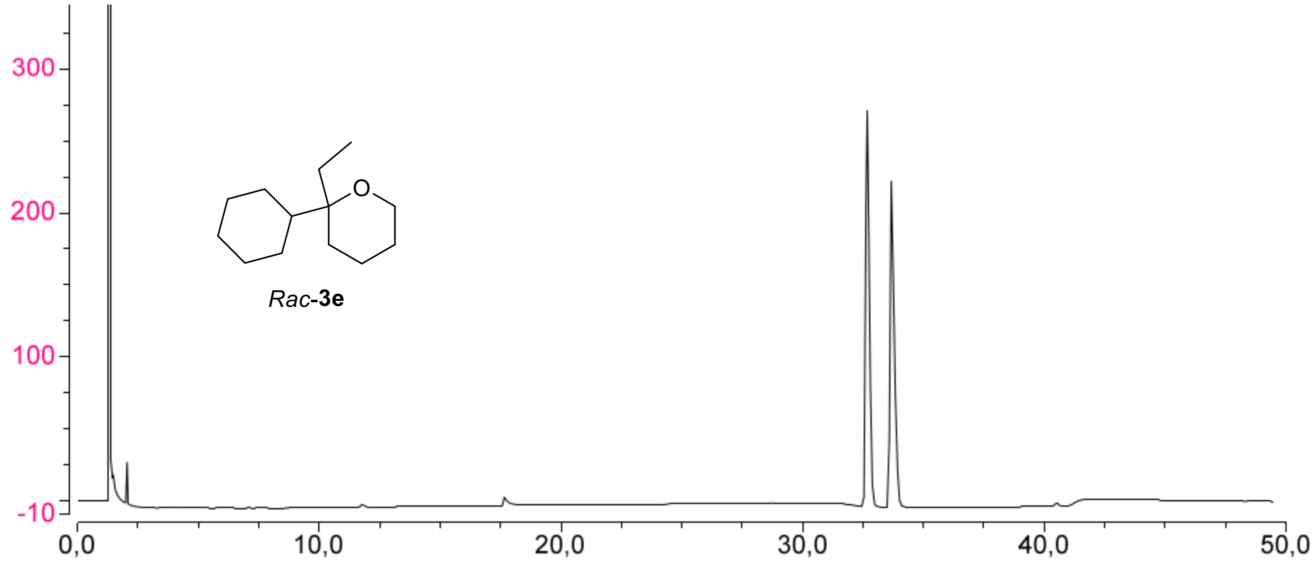

Peak#	Ret. Time (min)	Area%
1	32.67	49.96
2	33.67	50.04
Total		100.00

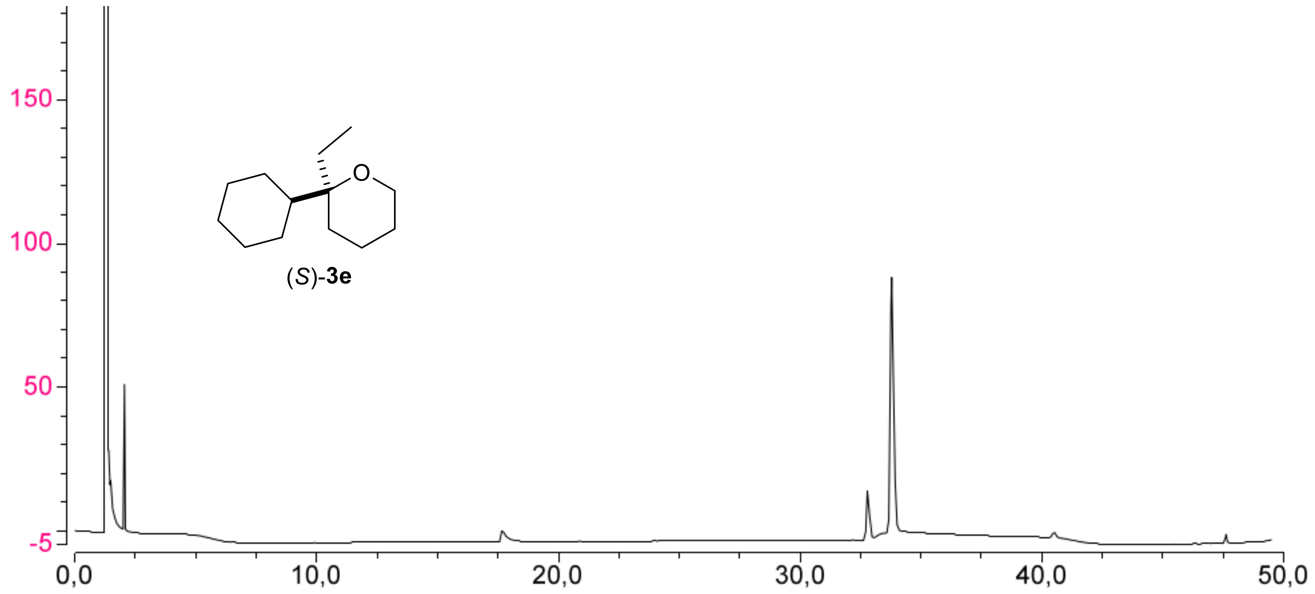

Peak#	Ret. Time (min)	Area%
1	32.81	13.58
2	33.79	86.42
Total		100.00

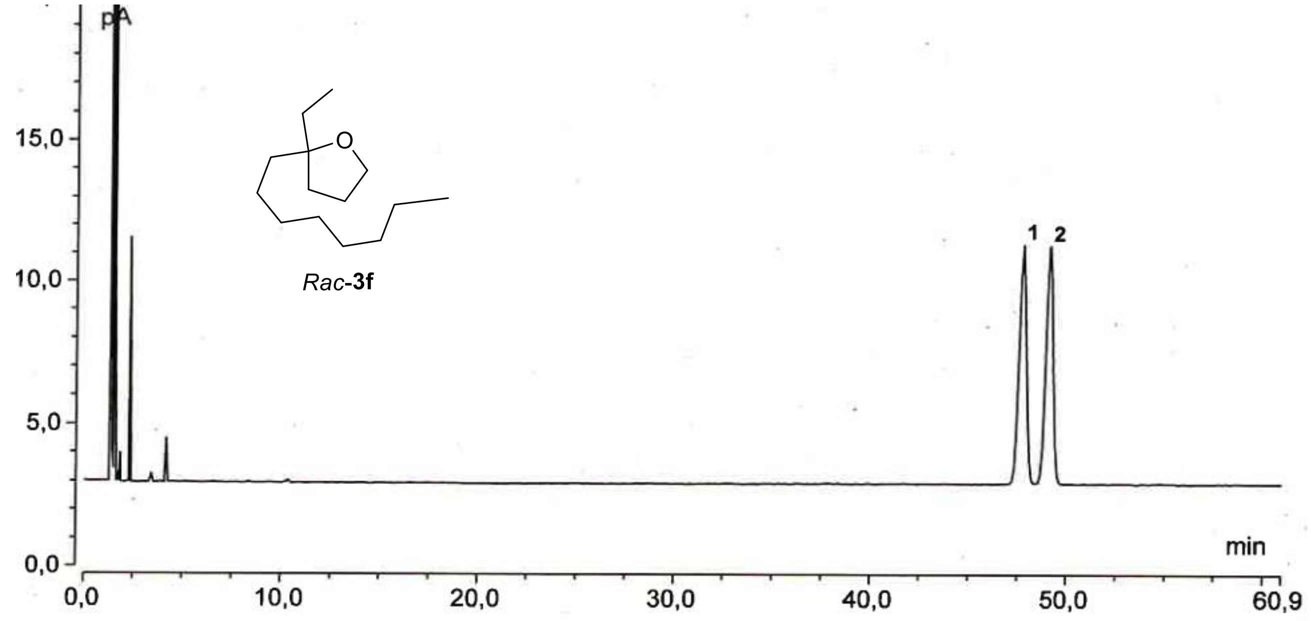

Peak#	Ret. Time (min)	Area%
1	47.74	50.00
2	49.10	50.00
Total		100.00

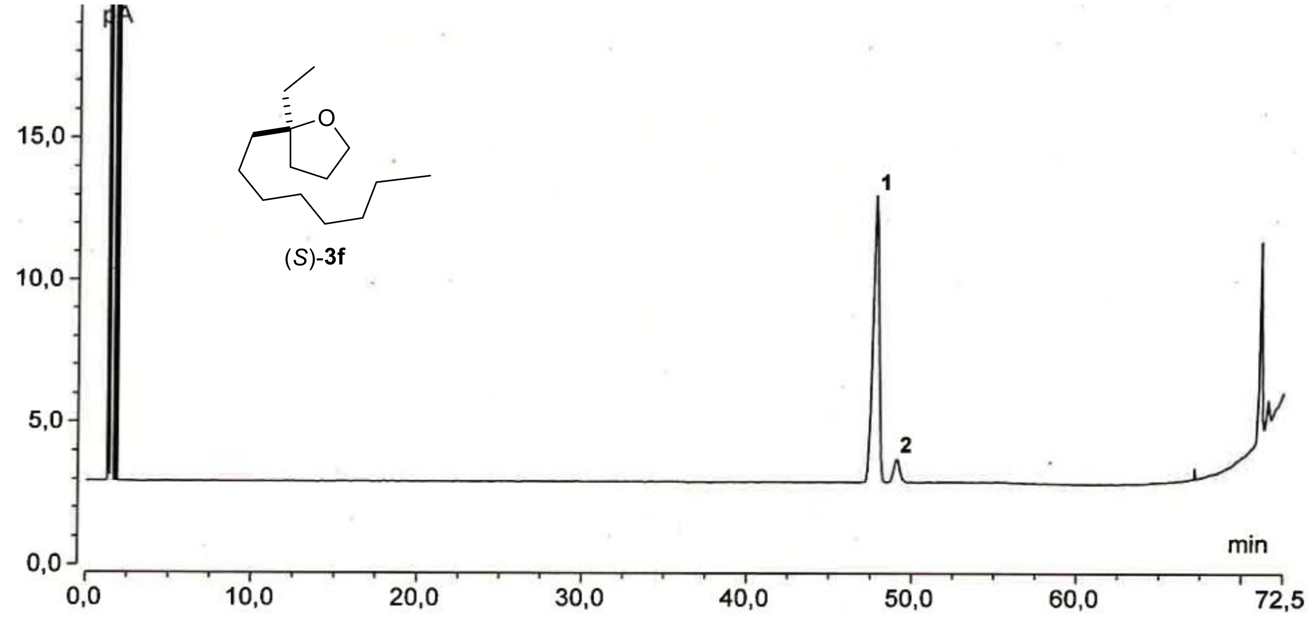

Peak#	Ret. Time (min)	Area%
1	47.76	92.01
2	49.06	7.99
Total		100.00

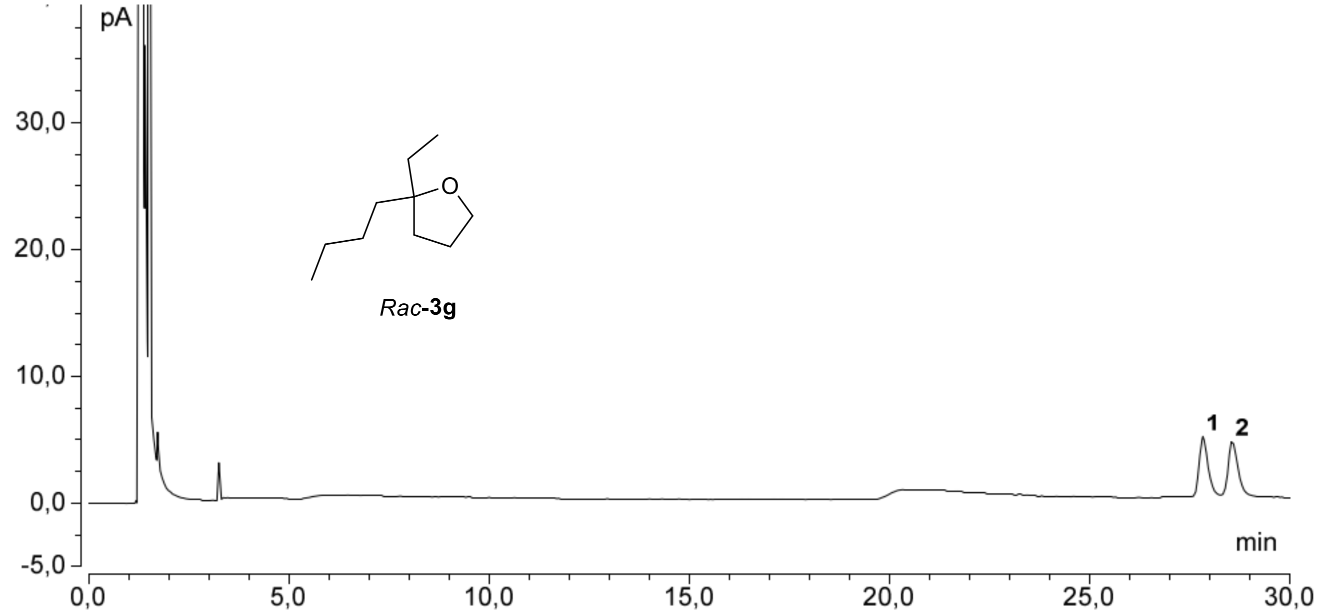

Peak#	Ret. Time (min)	Area%
1	27.83	50.01
2	28.56	49.98
Total		100.00

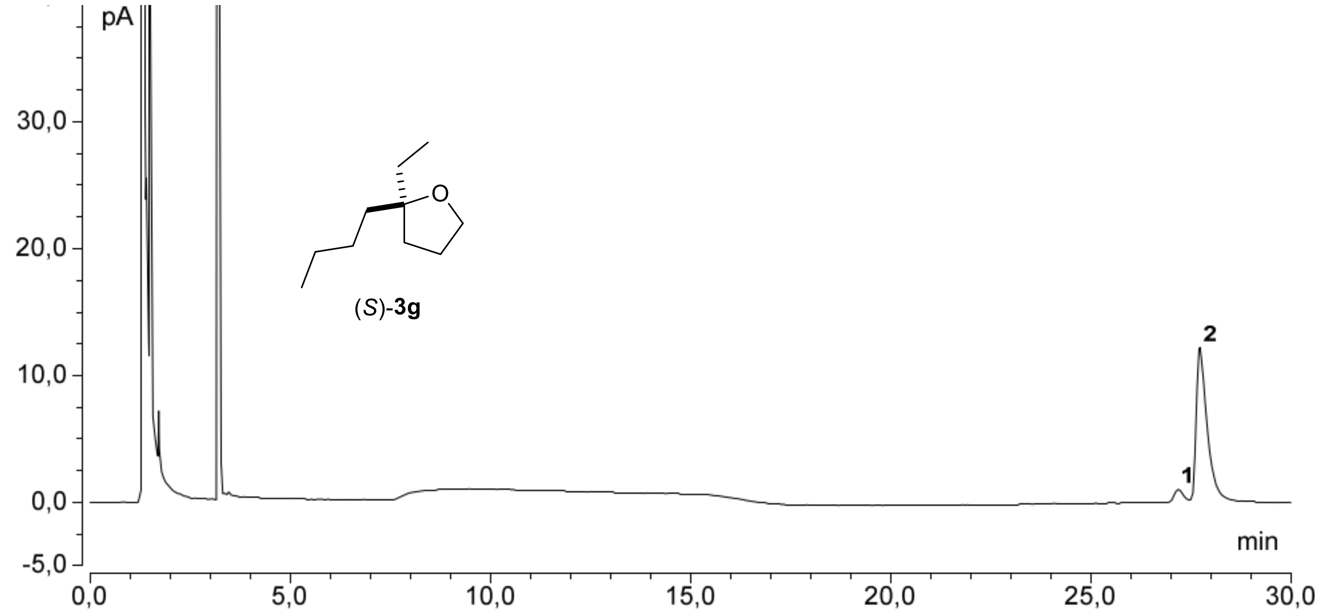

Peak#	Ret. Time (min)	Area%
1	27.19	6.64
2	27.72	93.36
Total		100.00

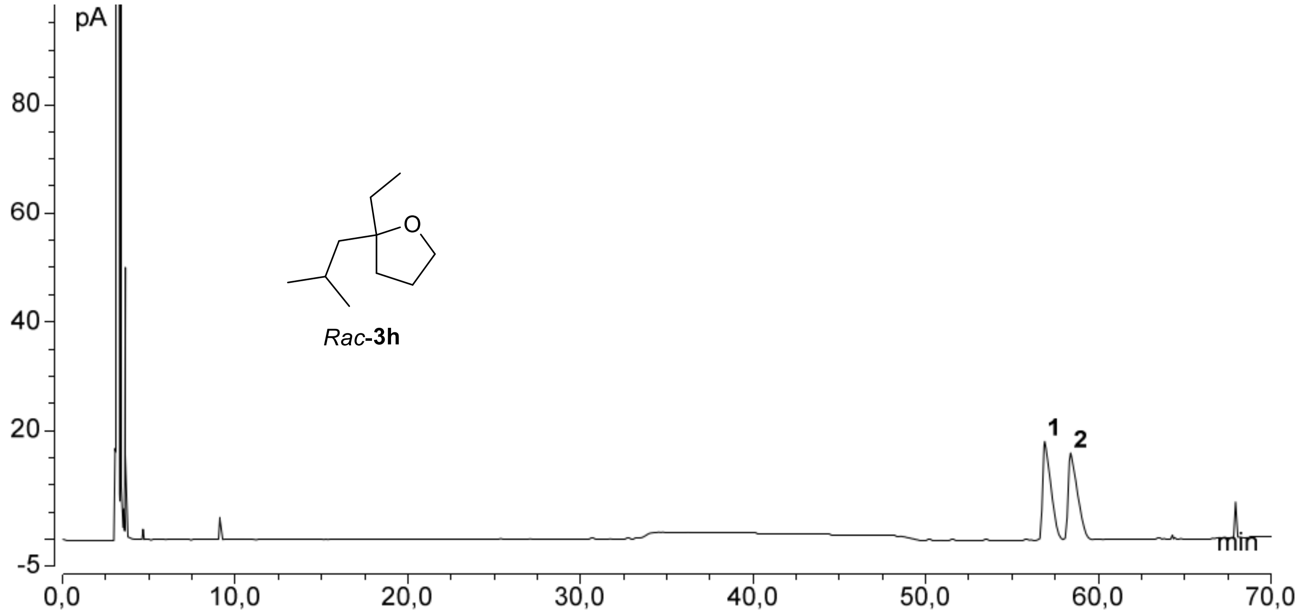

Peak#	Ret. Time (min)	Area%
1	56.89	49.97
2	58.39	50.03
Total		100.00

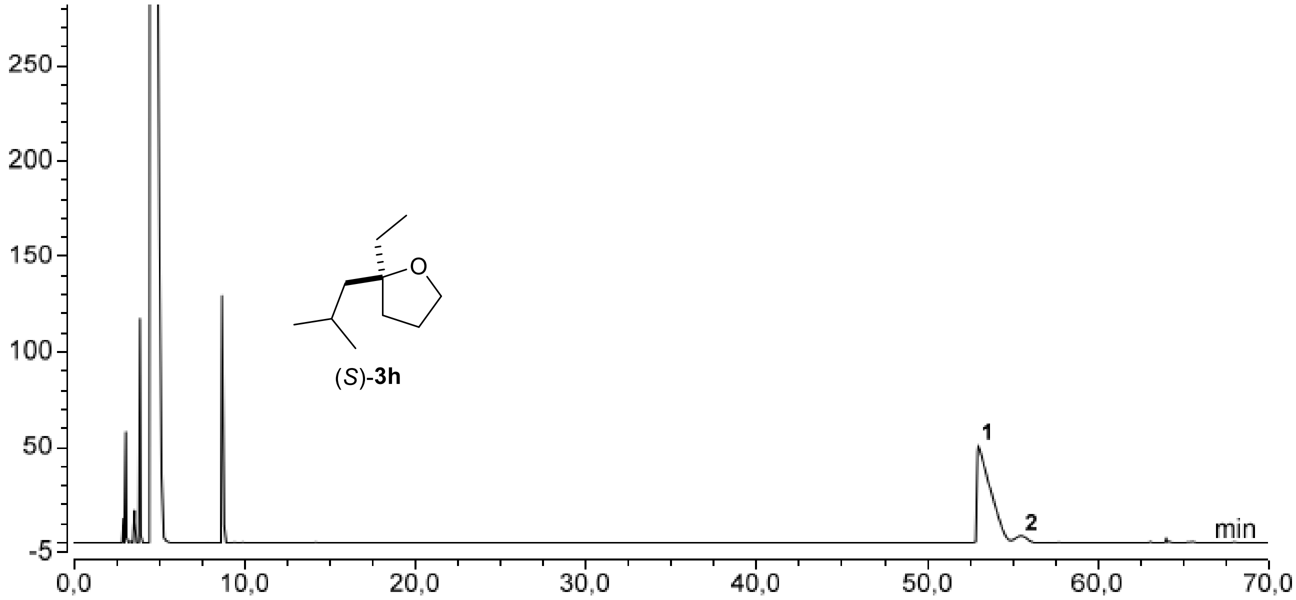

Peak#	Ret. Time (min)	Area%
1	52.99	94.27
2	56.51	5.73
Total		100.00

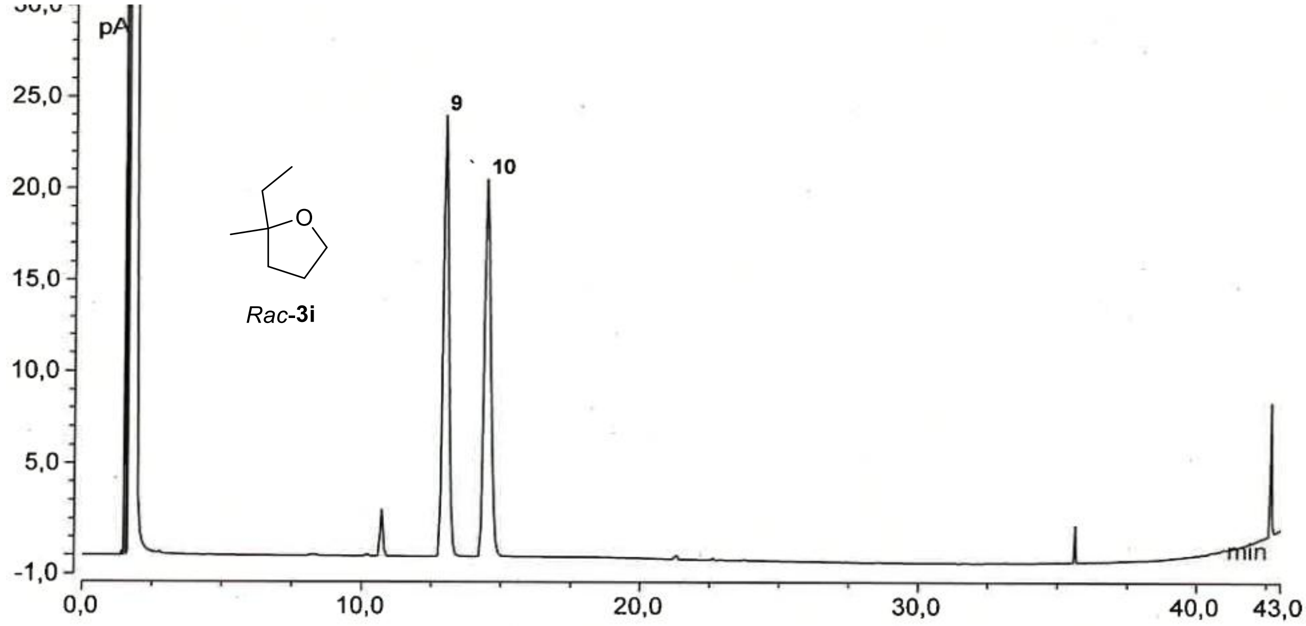

Peak#	Ret. Time (min)	Area%
1	12.93	49.98
2	14.42	50.02
Total		100.00

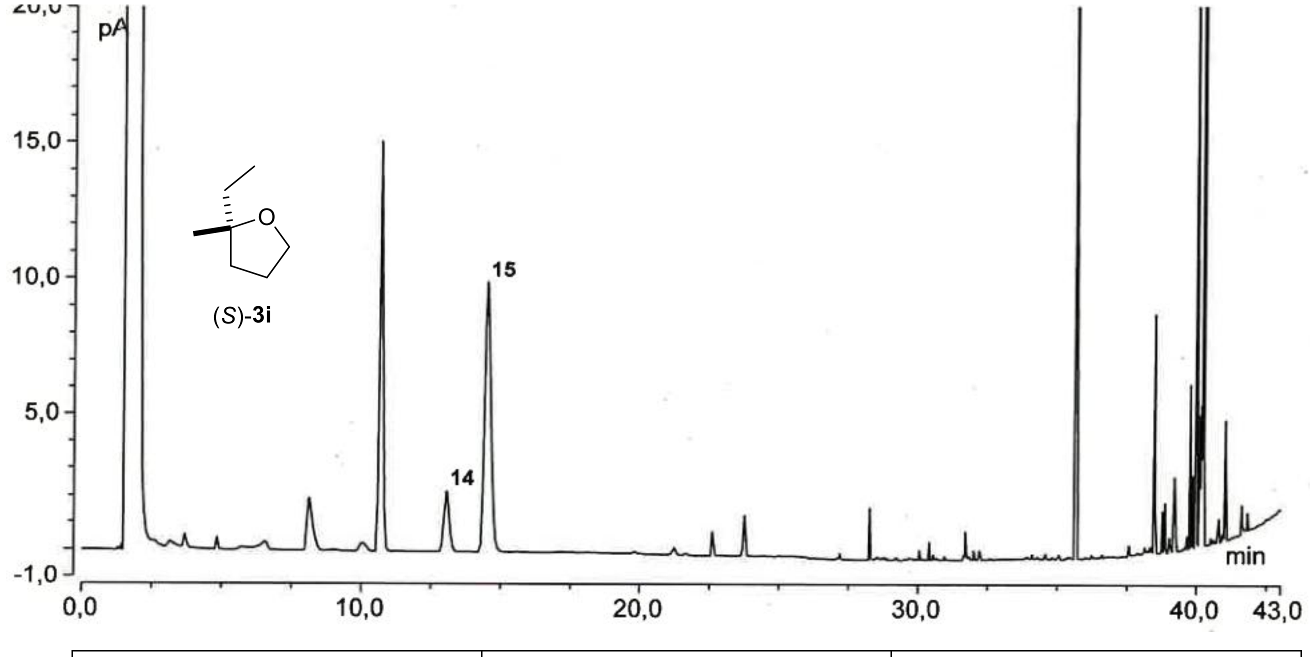

Peak#	Ret. Time (min)	Area%
1	13.04	16.07
2	14.47	83.93
Total		100.00

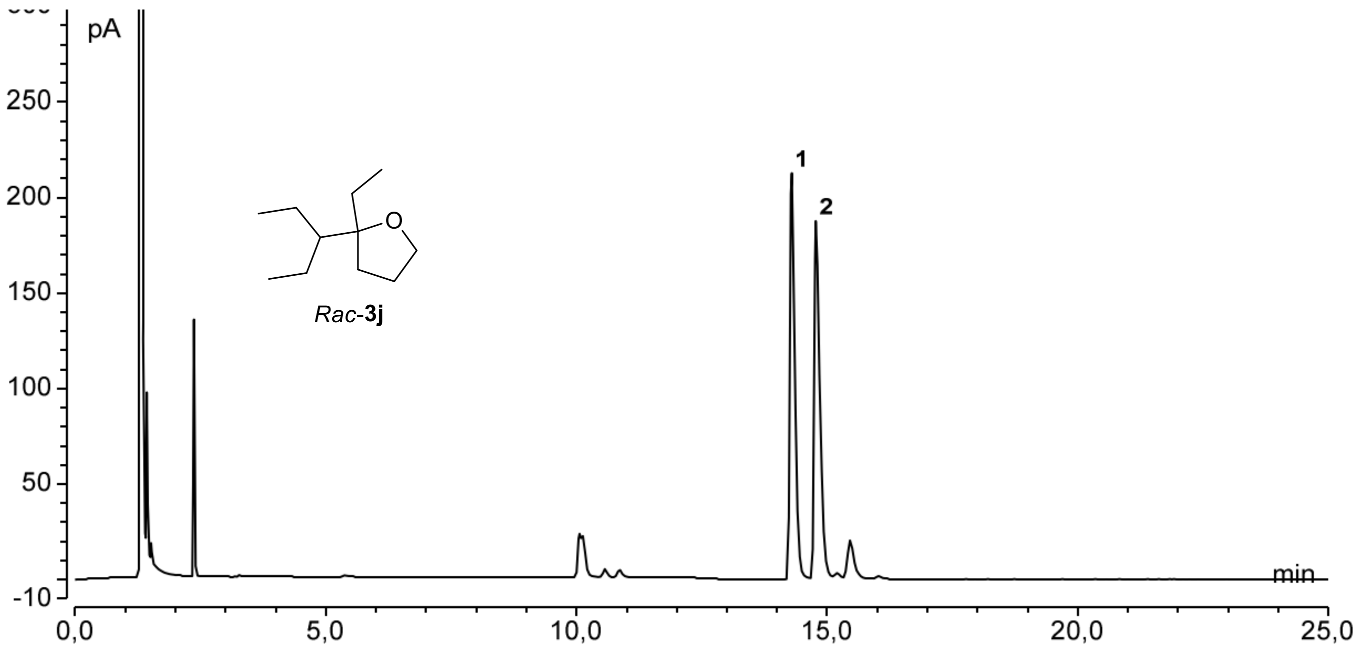

Peak#	Ret. Time (min)	Area%
1	14.29	50.09
2	14.78	49.91
Total		100.00

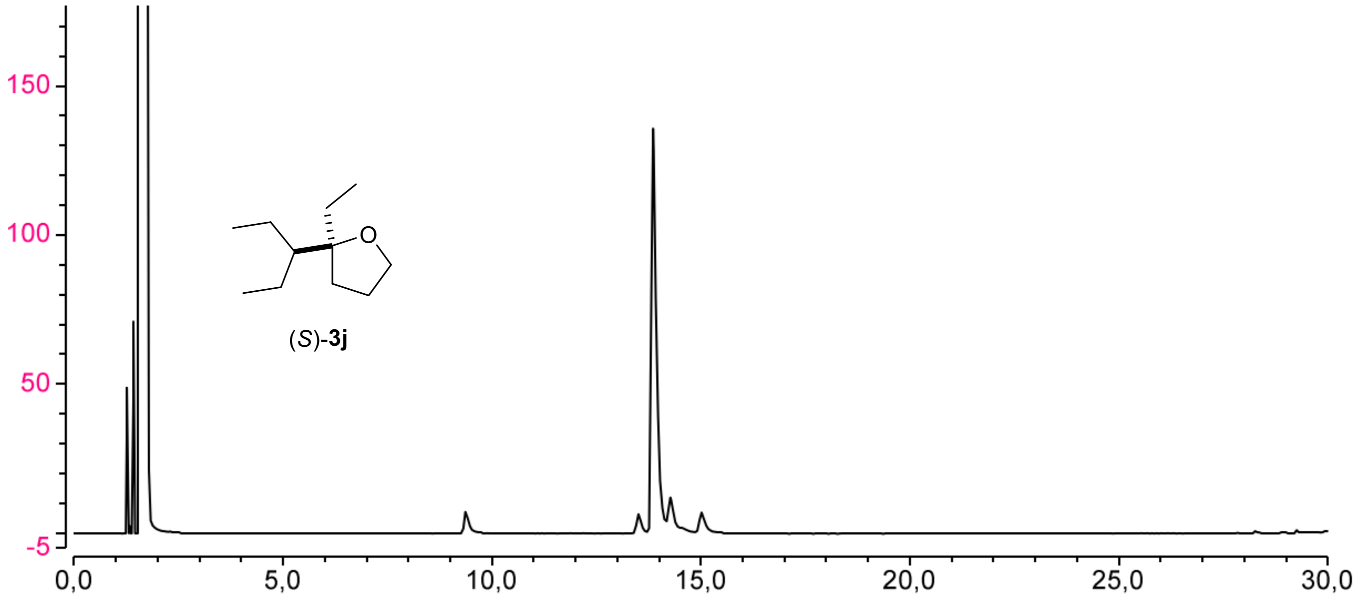

Peak#	Ret. Time (min)	Area%
1	13.50	4.57
2	13.85	95.43
Total		100.00

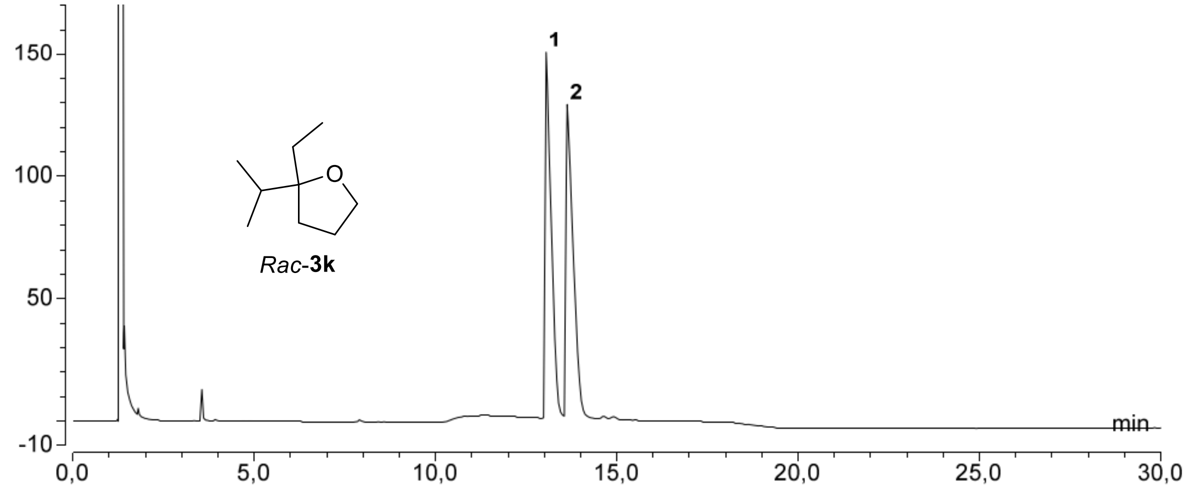

Peak#	Ret. Time (min)	Area%
1	13.05	50.06
2	13.63	49.94
Total		100.00

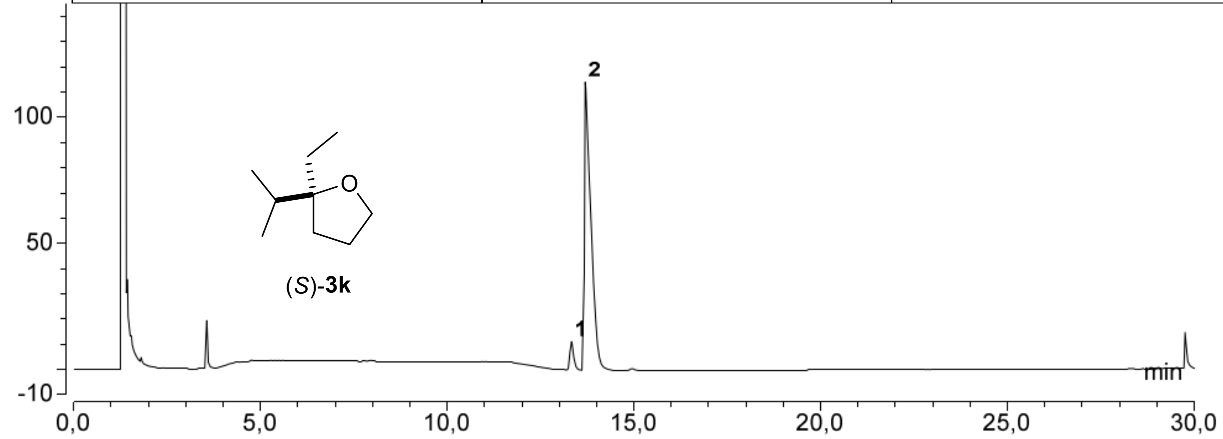

Peak#	Ret. Time (min)	Area%
1	13.33	4.94
2	13.70	95.06
Total		100.00

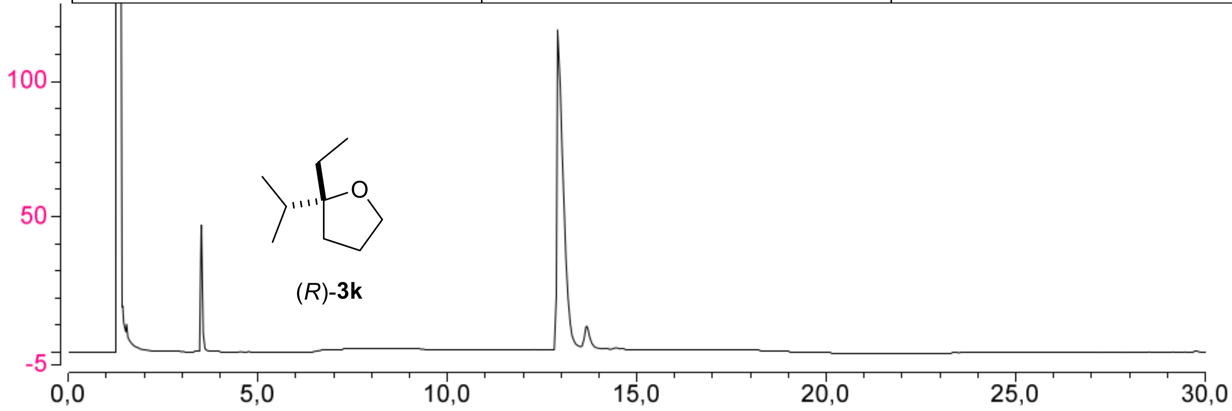

Peak#	Ret. Time (min)	Area%
1	12.92	94.50
2	13.68	5.50
Total		100.00

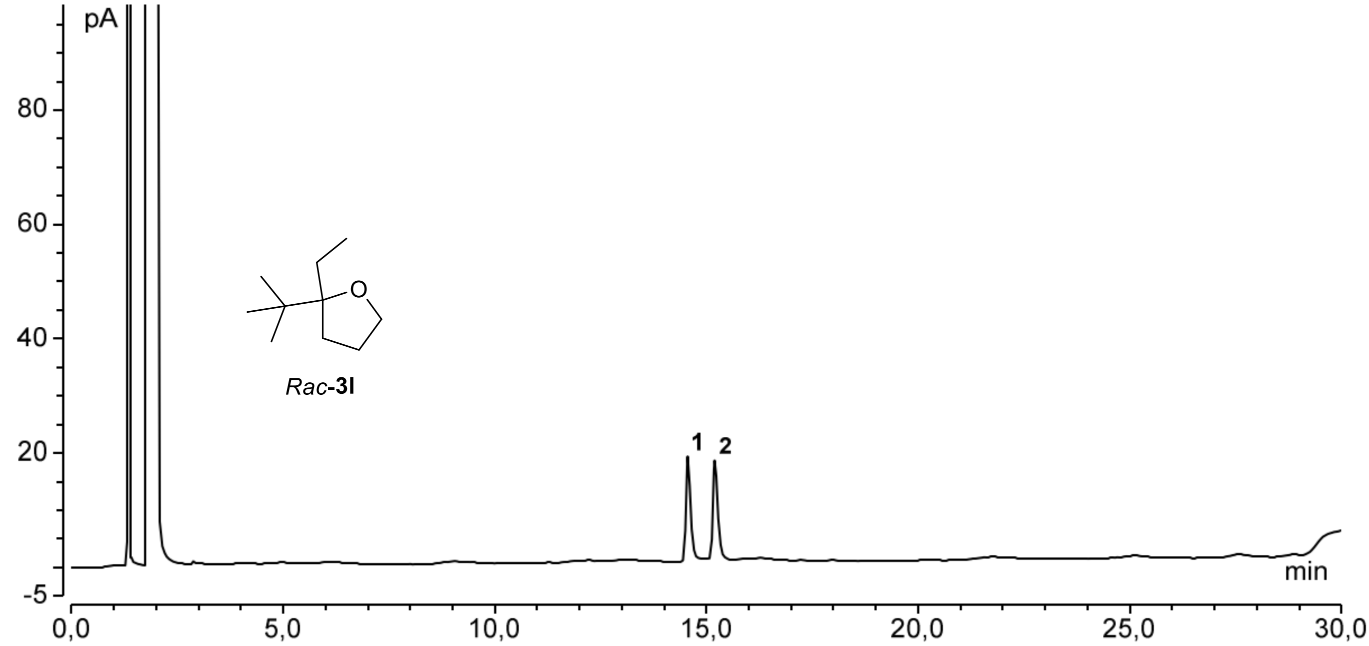

Peak#	Ret. Time (min)	Area%
1	14.56	49.92
2	15.19	50.08
Total		100.00

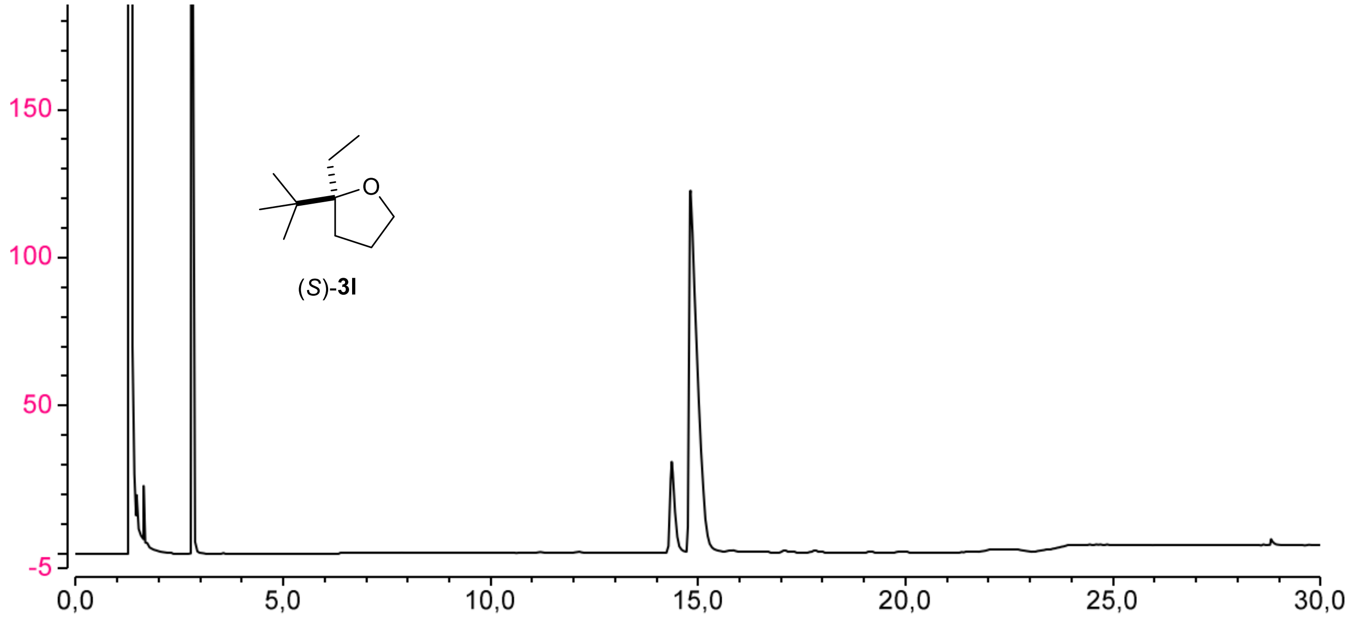

Peak#	Ret. Time (min)	Area%
1	14.36	12.38
2	14.82	87.62
Total		100.00

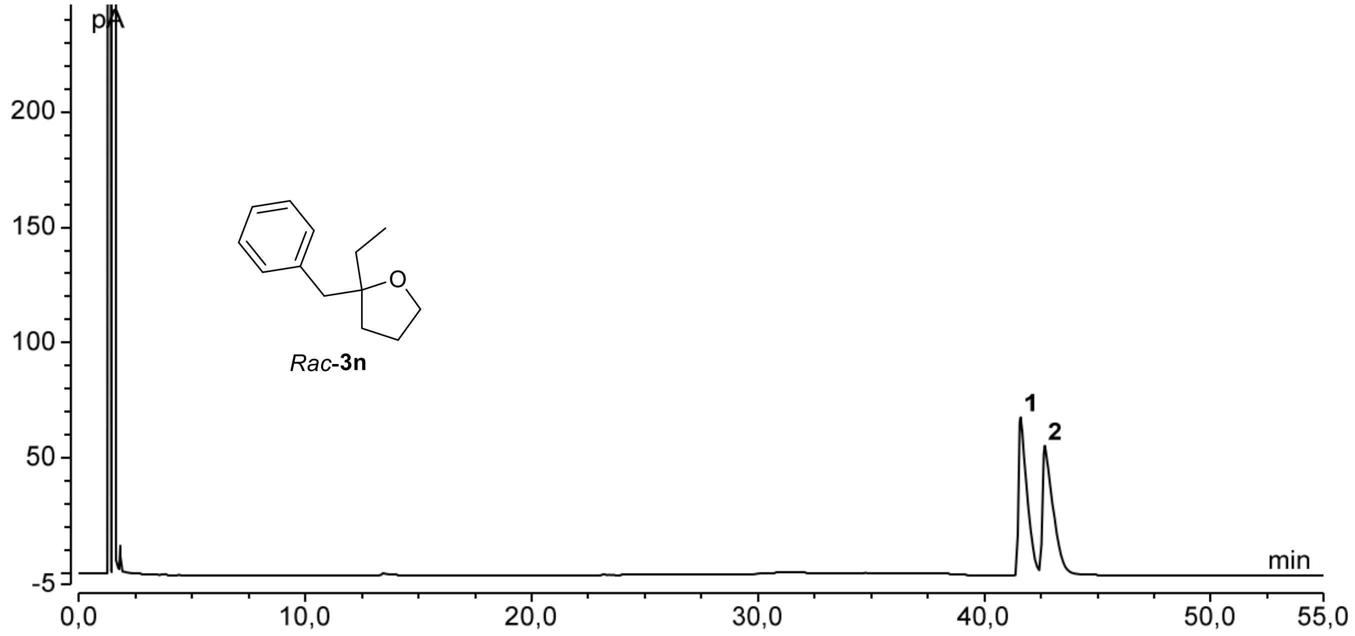

Peak#	Ret. Time (min)	Area%
1	41.61	49.94
2	42.67	50.06
Total		100.00

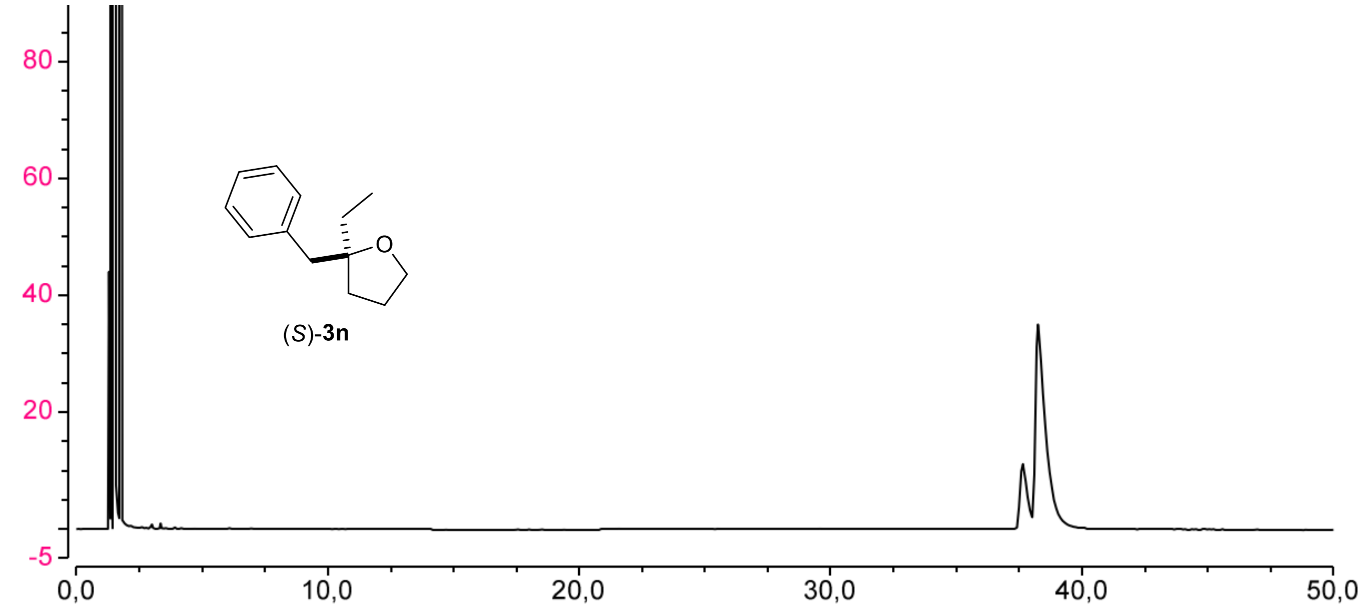

Peak#	Ret. Time (min)	Area%
1	37.65	18.10
2	38.25	81.90
Total		100.00

7.2. HPLC trace

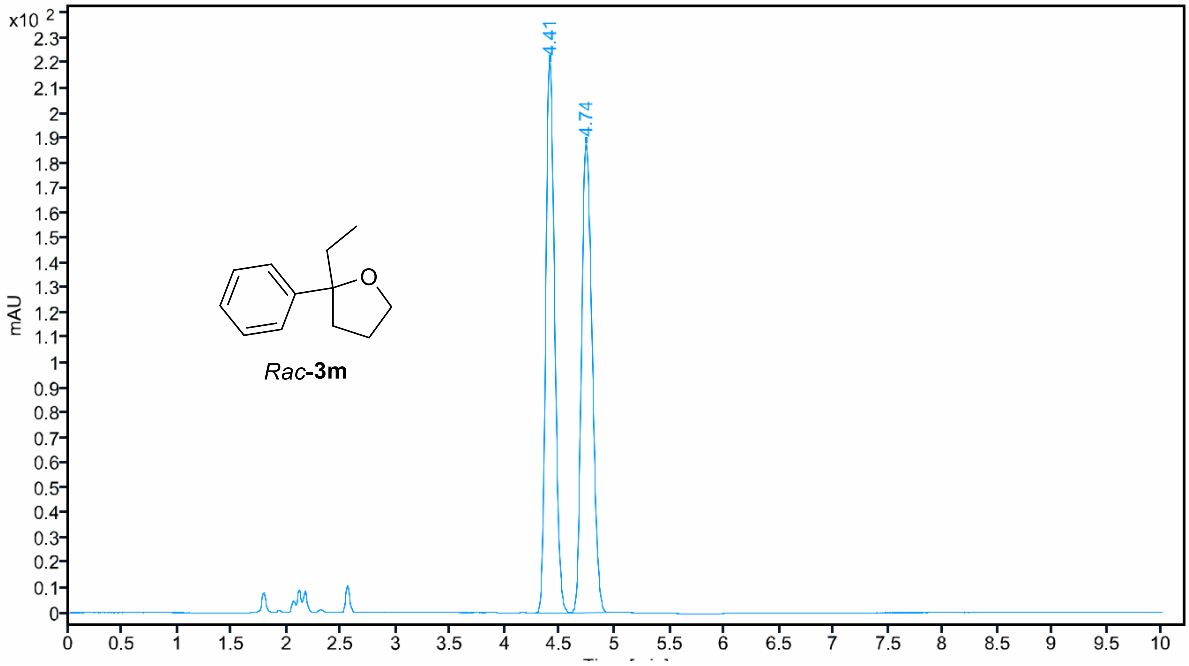

Peak#	Ret. Time (min)	Area%
1	4.41	49.71
2	4.75	50.29
Total		100.00

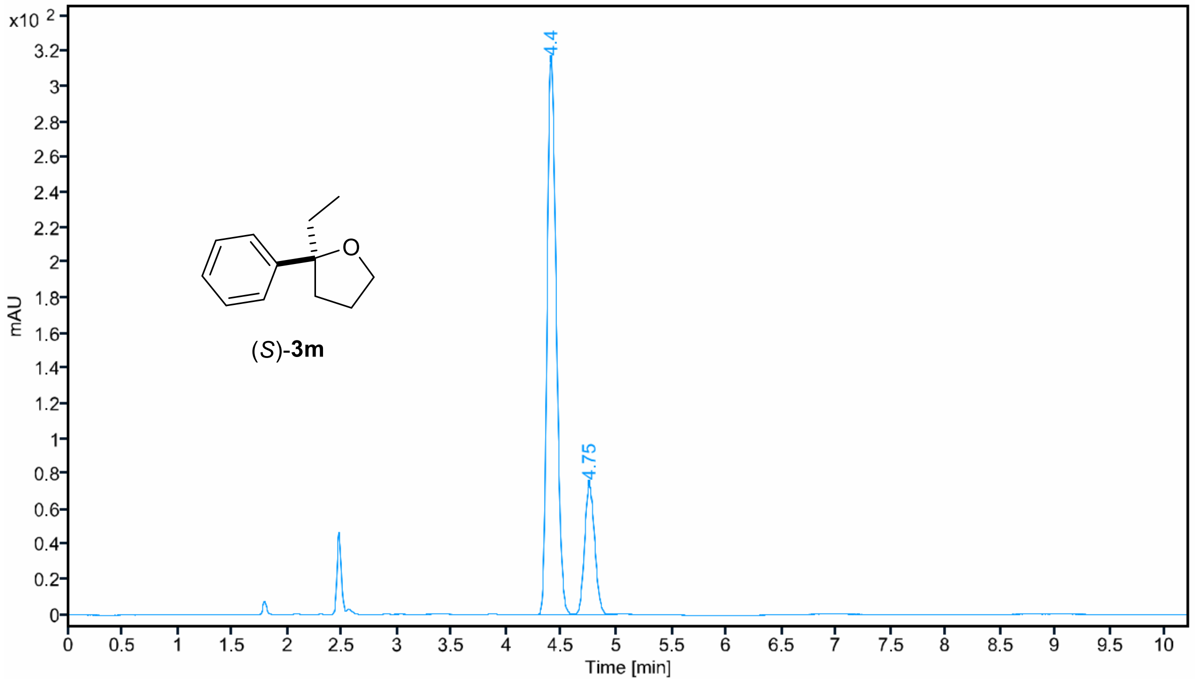

Peak#	Ret. Time (min)	Area%
1	4.40	79.57
2	4.75	20.43
Total		100.00

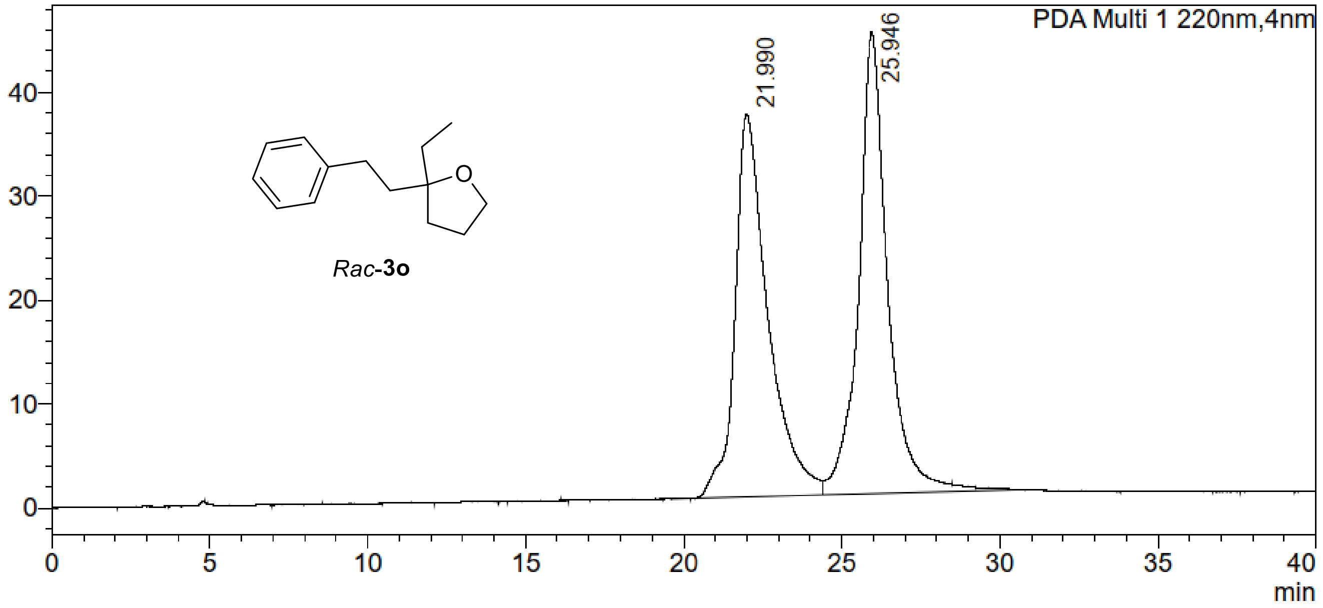

Peak#	Ret. Time (min)	Area%
1	21.99	48.54
2	25.95	51.46
Total		100.00

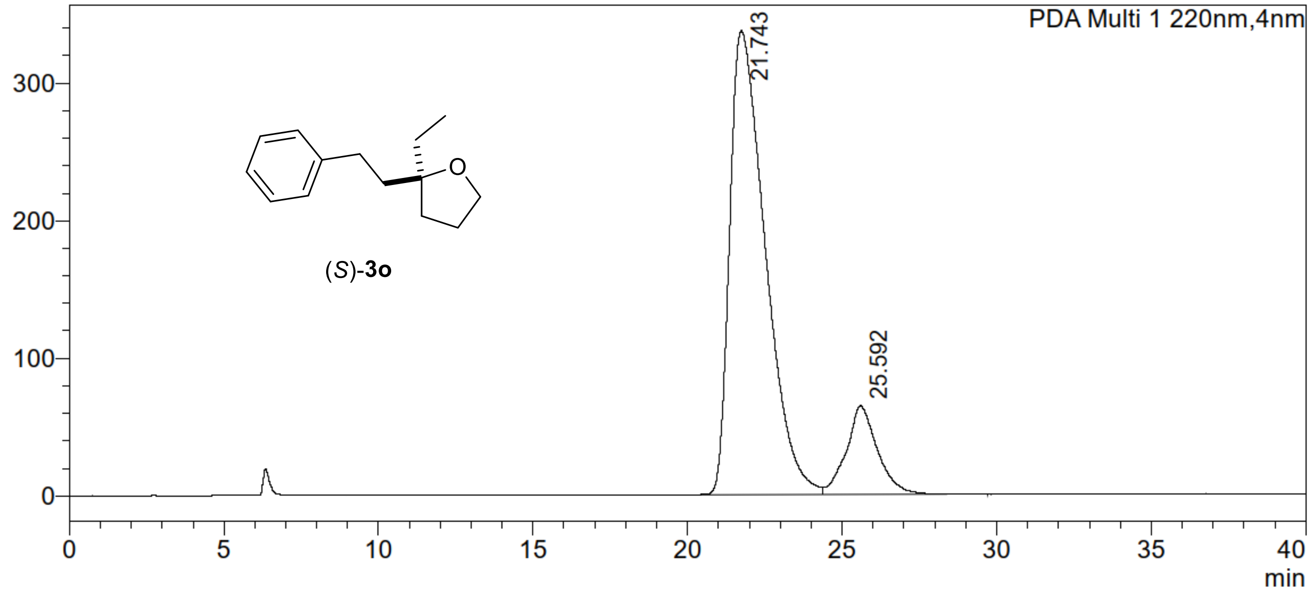

Peak#	Ret. Time (min)	Area%
1	21.74	85.71
2	25.59	14.29
Total		100.00

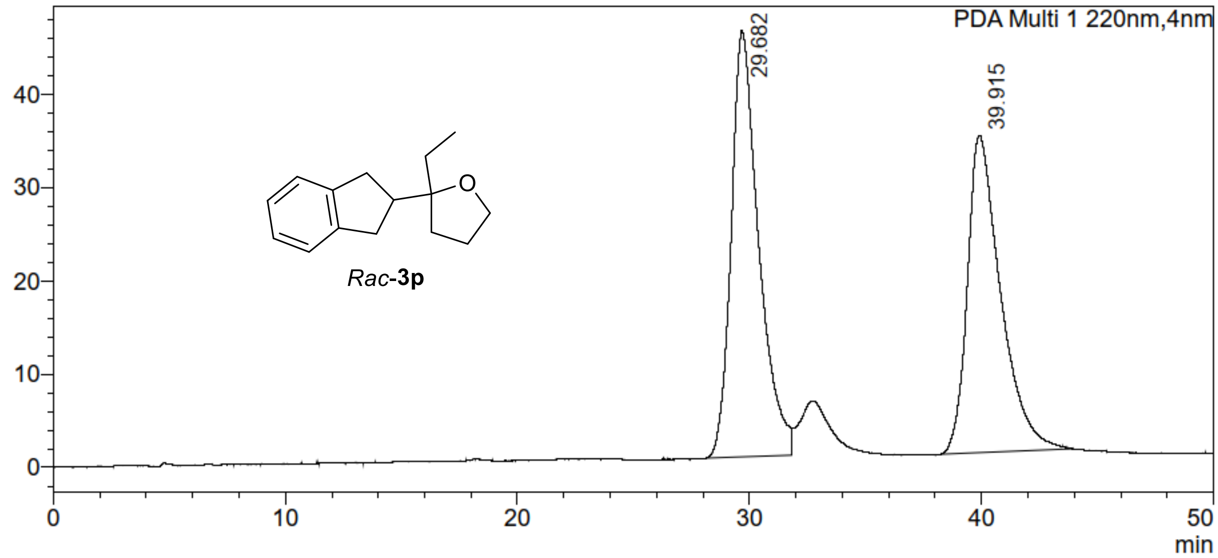

Peak#	Ret. Time (min)	Area%
1	29.68	52.56
2	39.92	47.44
Total		100.00

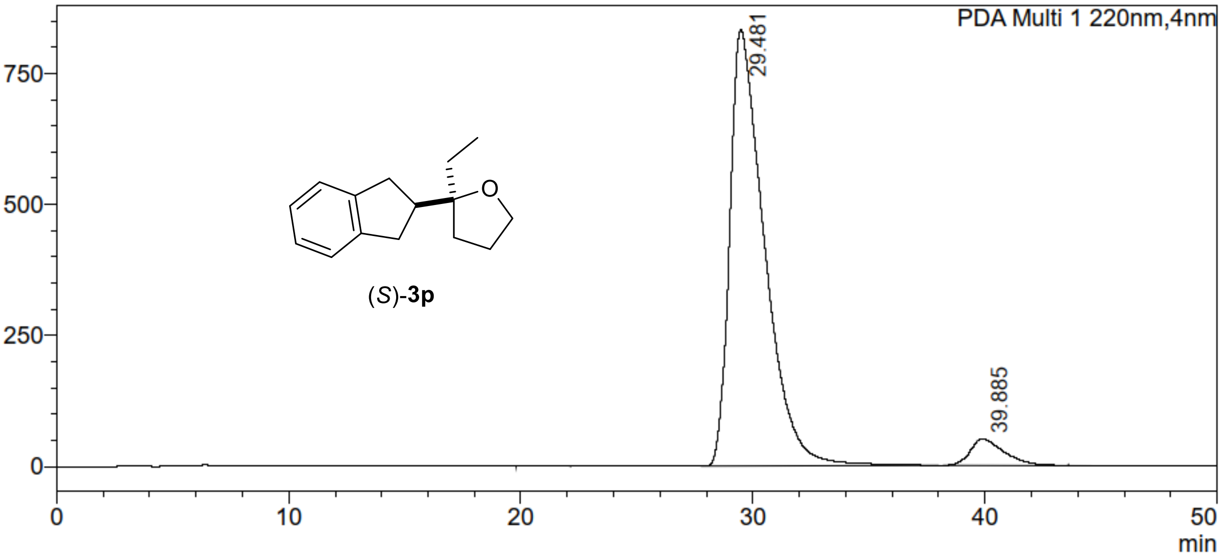

Peak#	Ret. Time (min)	Area%
1	29.48	94.23
2	39.89	5.77
Total		100.00

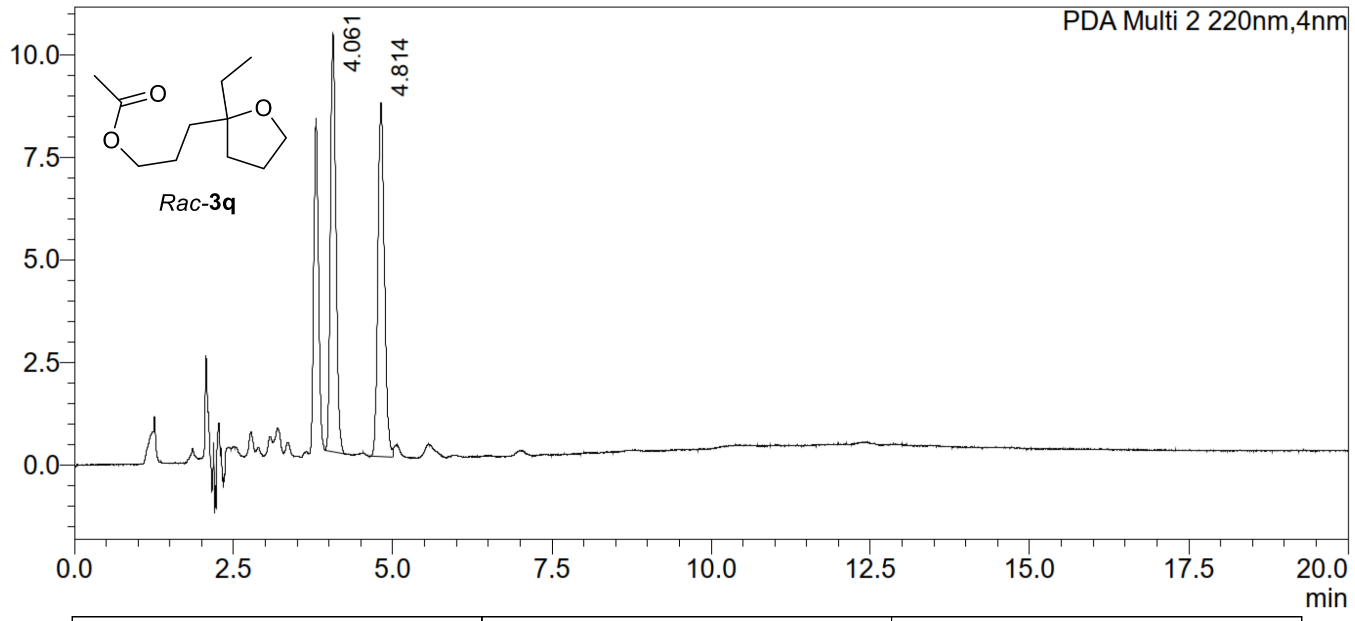

Peak#	Ret. Time (min)	Area%
1	4.06	50.67
2	4.81	49.33
Total		100.00

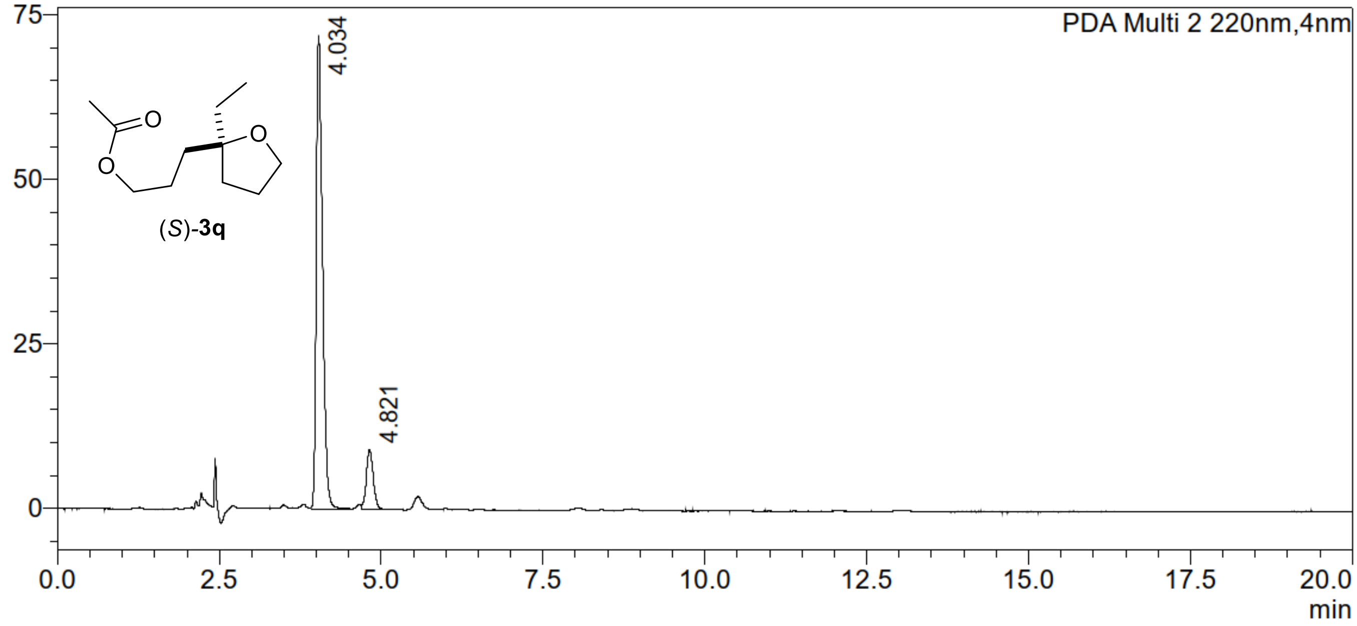

Peak#	Ret. Time (min)	Area%
1	4.03	87.88
2	4.82	12.12
Total		100.00

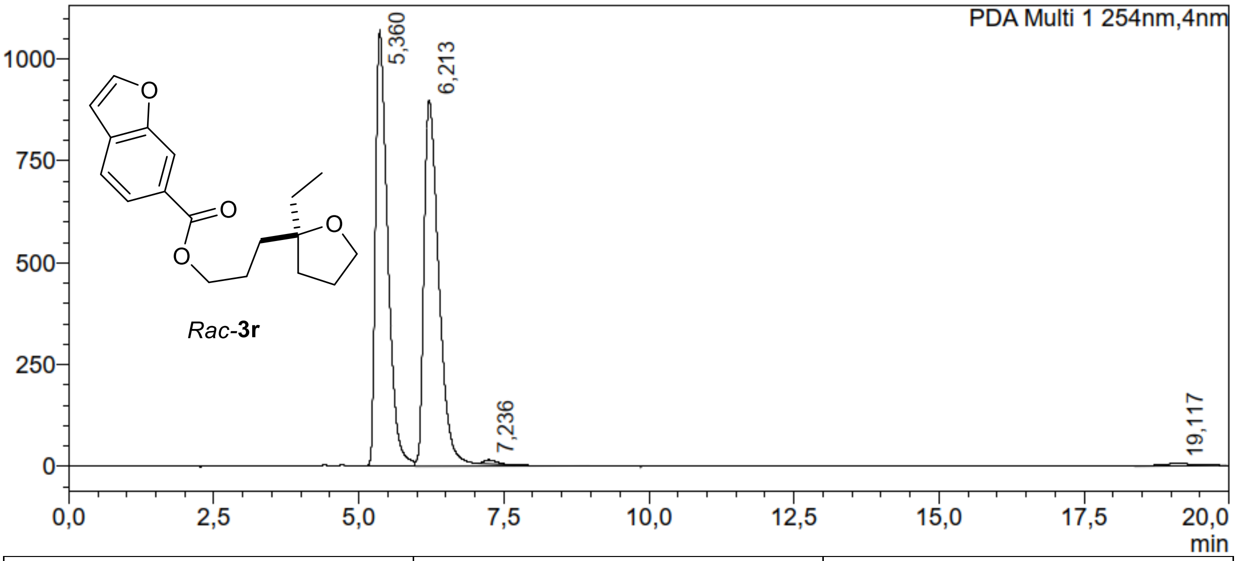

Peak#	Ret. Time (min)	Area%
1	5.41	48.97
2	6.30	51.03
Total		100.00

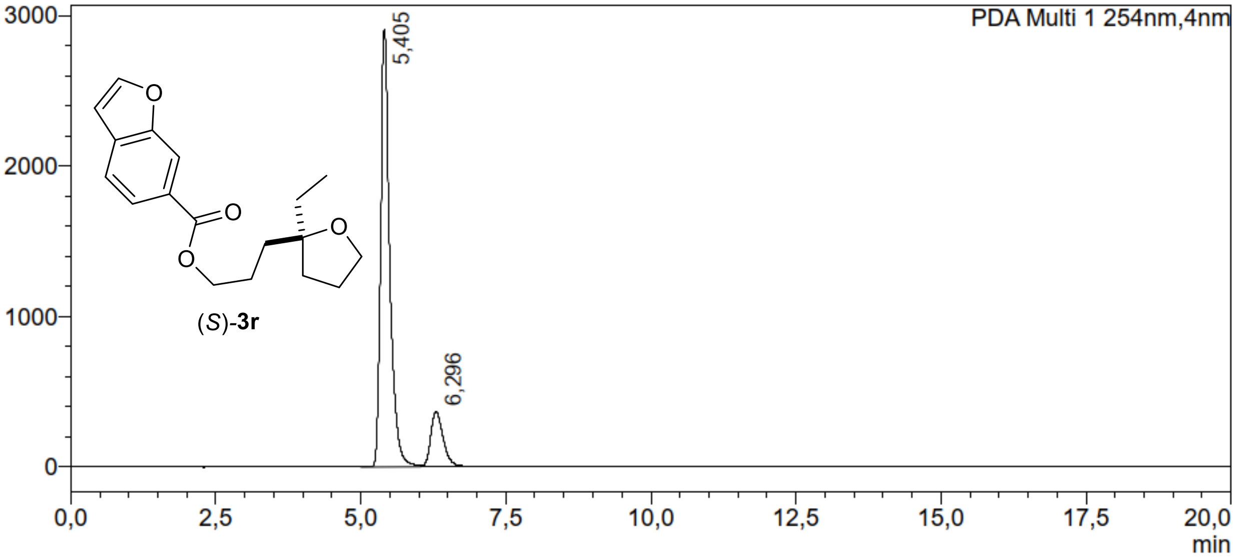

Peak#	Ret. Time (min)	Area%
1	5.36	84.66%
2	6.21	15.34%
Total		100.00

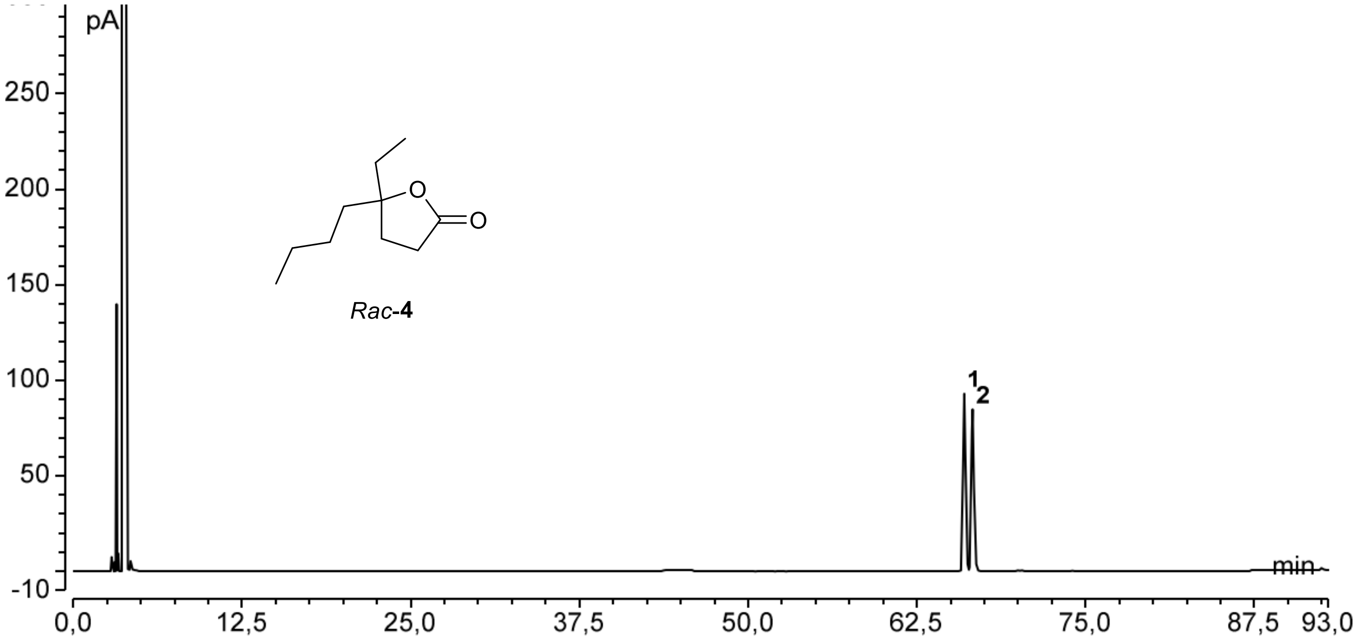

Peak#	Ret. Time (min)	Area%
1	66.02	49.75
2	66.63	50.25
Total		100.00

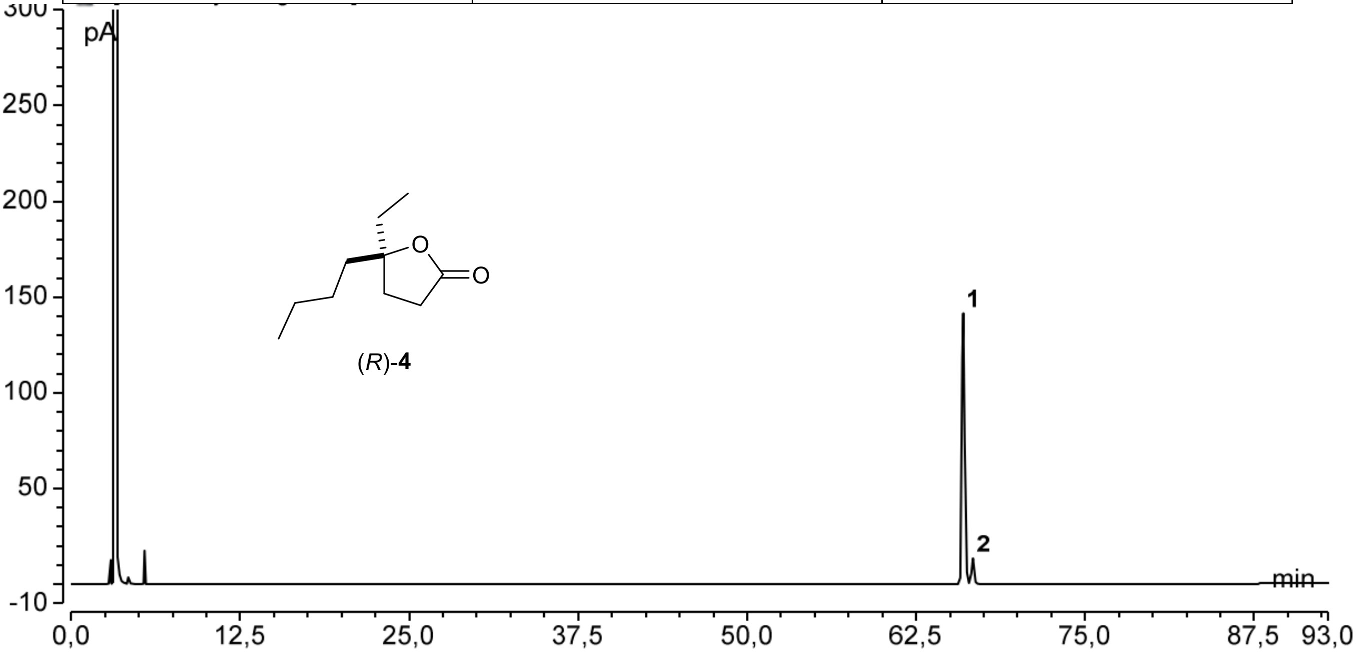

Peak#	Ret. Time (min)	Area%
1	65.99	92.36
2	66.70	7.64
Total		100.00

8. ^{1}H, ^{13}C, ^{31}P and ^{19}F NMR spectra of substrates and products

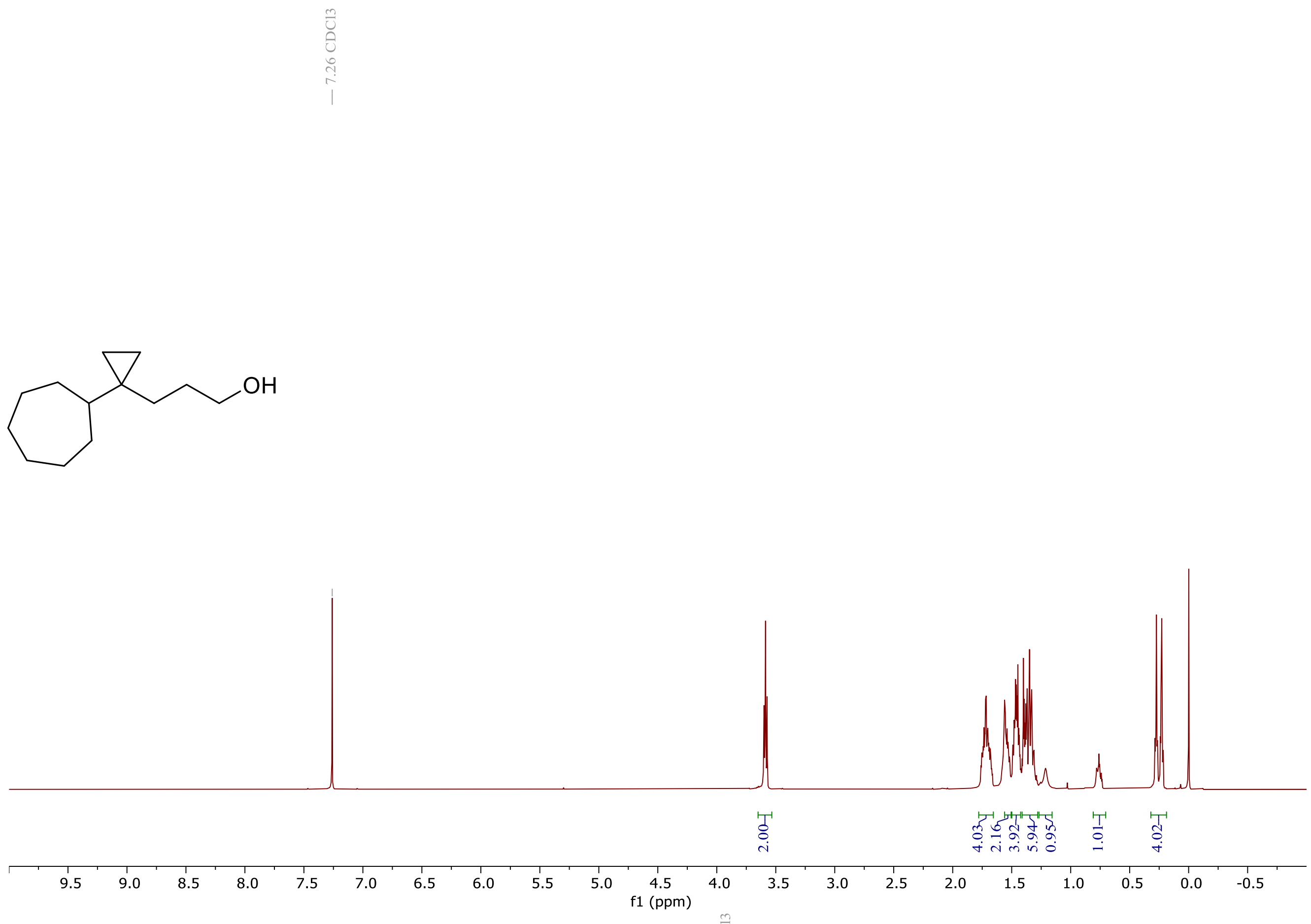

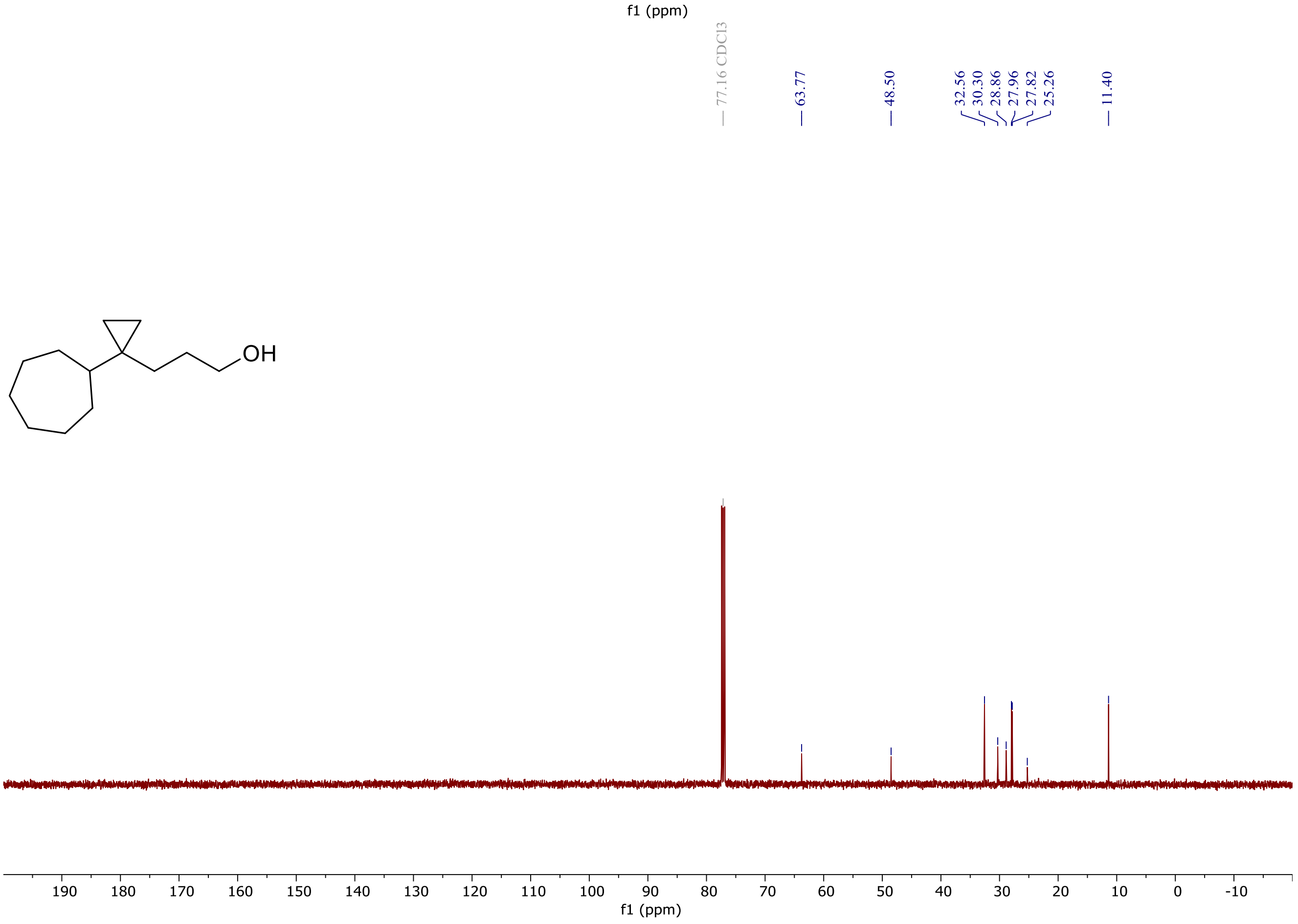

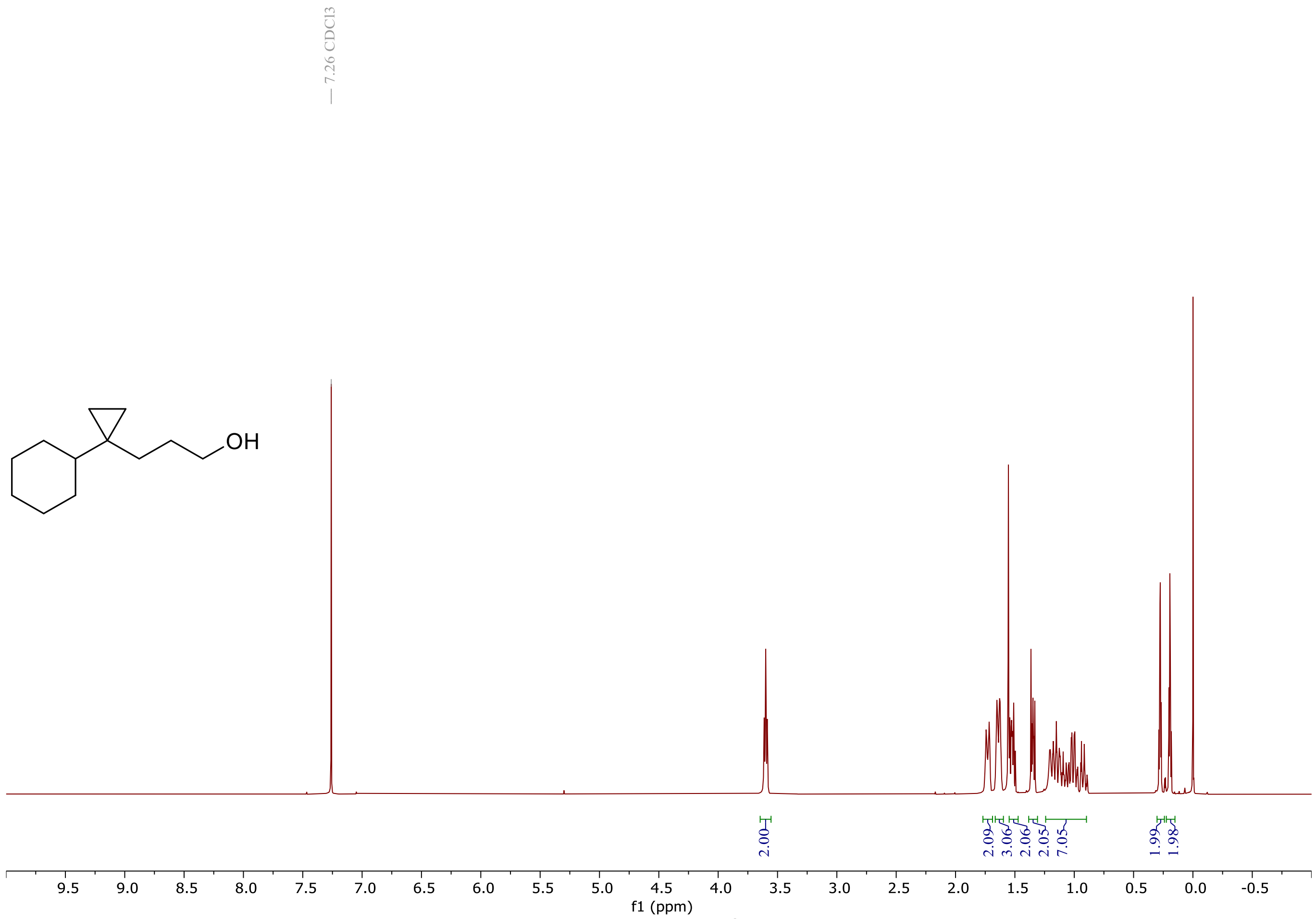
OH
7.26 CDCl3
2.00
2.09
3.06
2.06
2.05
7.05
1.99
1.98
f1 (ppm)

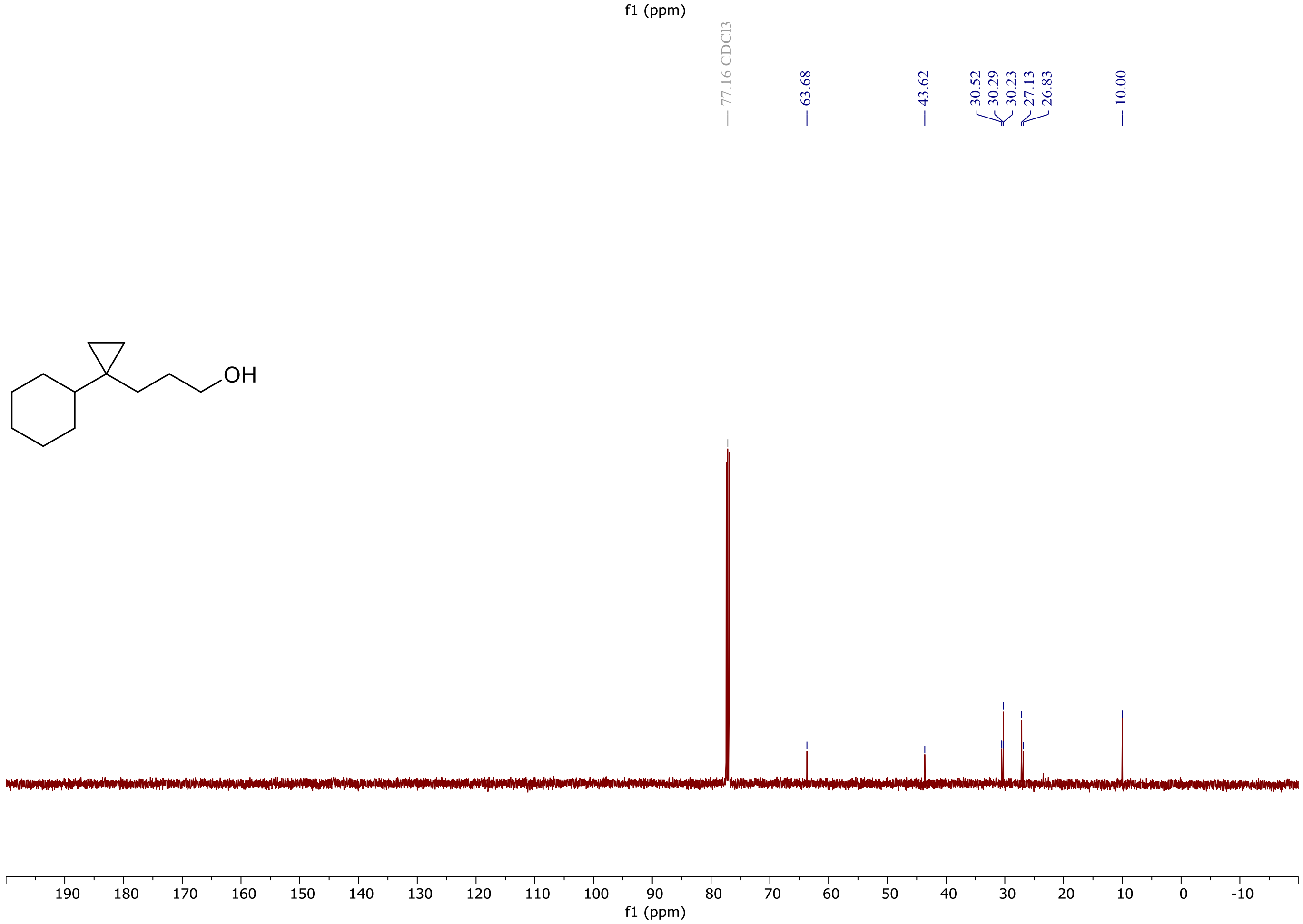
OH
77.16 CDCl3
63.68
43.62
30.52
30.29
30.23
27.13
26.83
10.00
f1 (ppm)

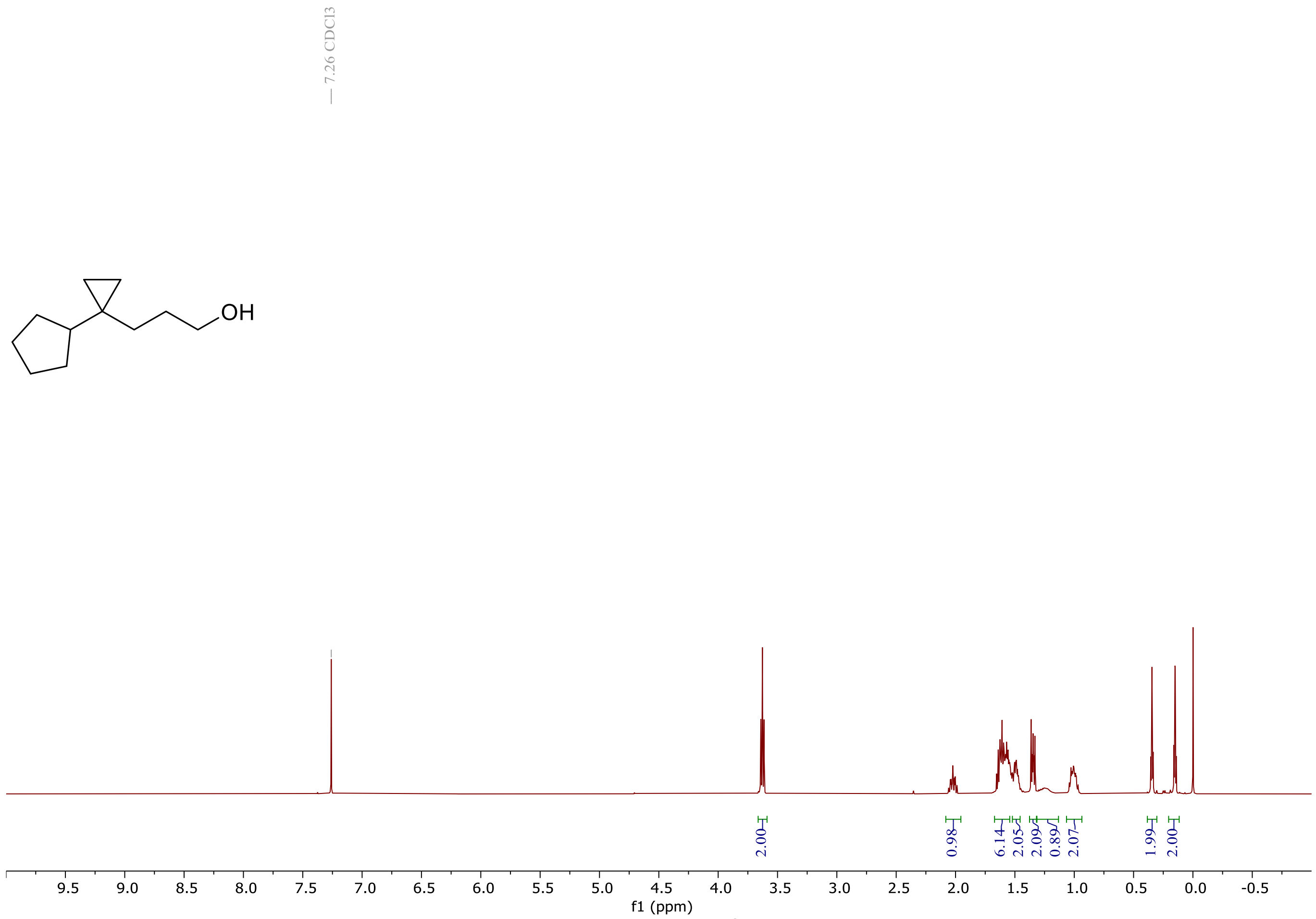
7.26 CDCl3
OH
2.00
0.98
6.14
2.05
2.09
0.89
2.07
1.99
2.00
9.5 9.0 8.5 8.0 7.5 7.0 6.5 6.0 5.5 5.0 4.5 4.0 3.5 3.0 2.5 2.0 1.5 1.0 0.5 0.0 -0.5
f1 (ppm)

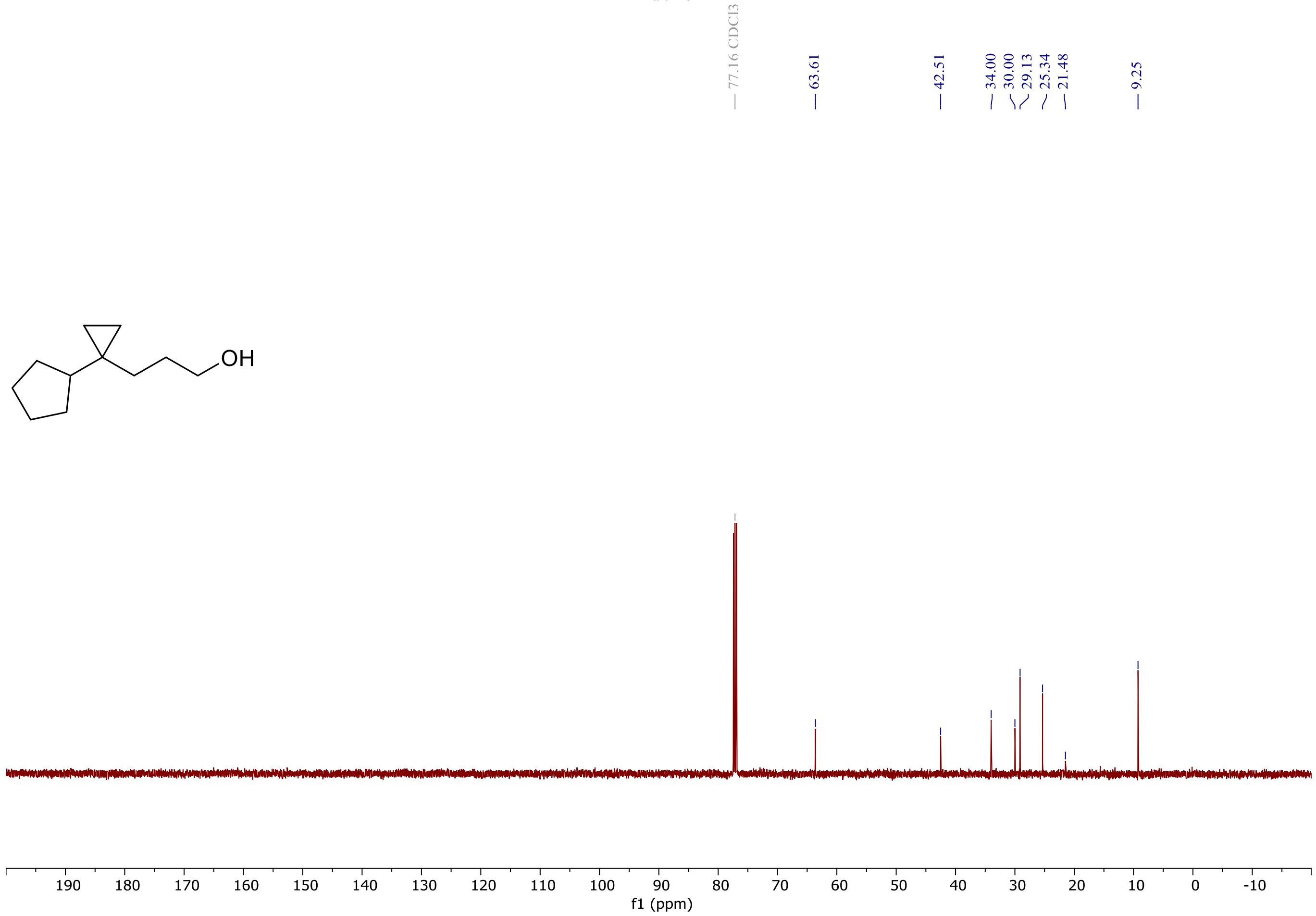
77.16 CDCl3
63.61
42.51
34.00
30.00
29.13
25.34
21.48
9.25
OH
190 180 170 160 150 140 130 120 110 100 90 80 70 60 50 40 30 20 10 0 -10
f1 (ppm)

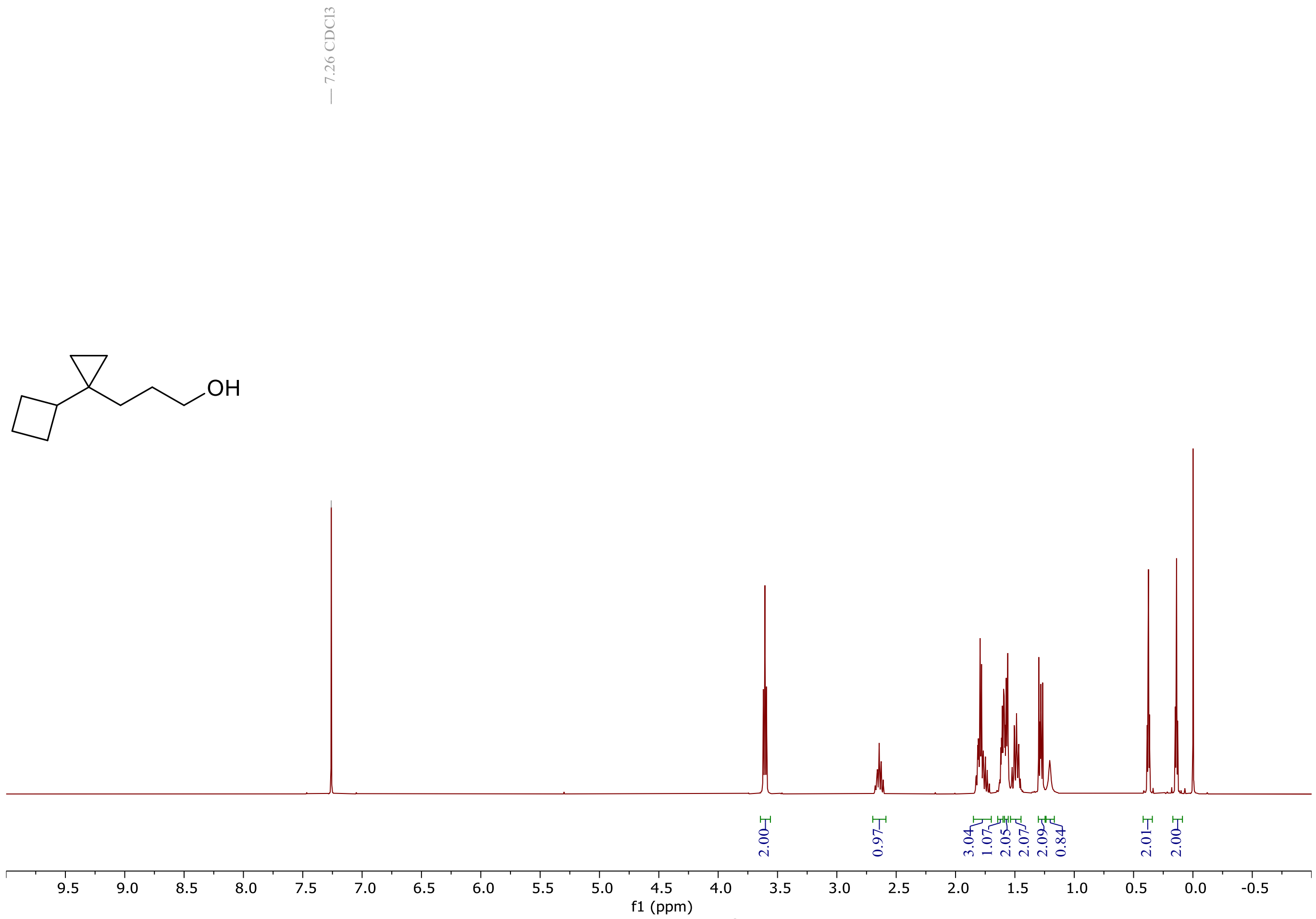

— 7.26 CDCl3
OH
2.00
0.97
3.04
1.07
2.05
2.07
2.09
0.84
2.01
2.00
9.5 9.0 8.5 8.0 7.5 7.0 6.5 6.0 5.5 5.0 4.5 4.0 3.5 3.0 2.5 2.0 1.5 1.0 0.5 0.0 -0.5
f1 (ppm)

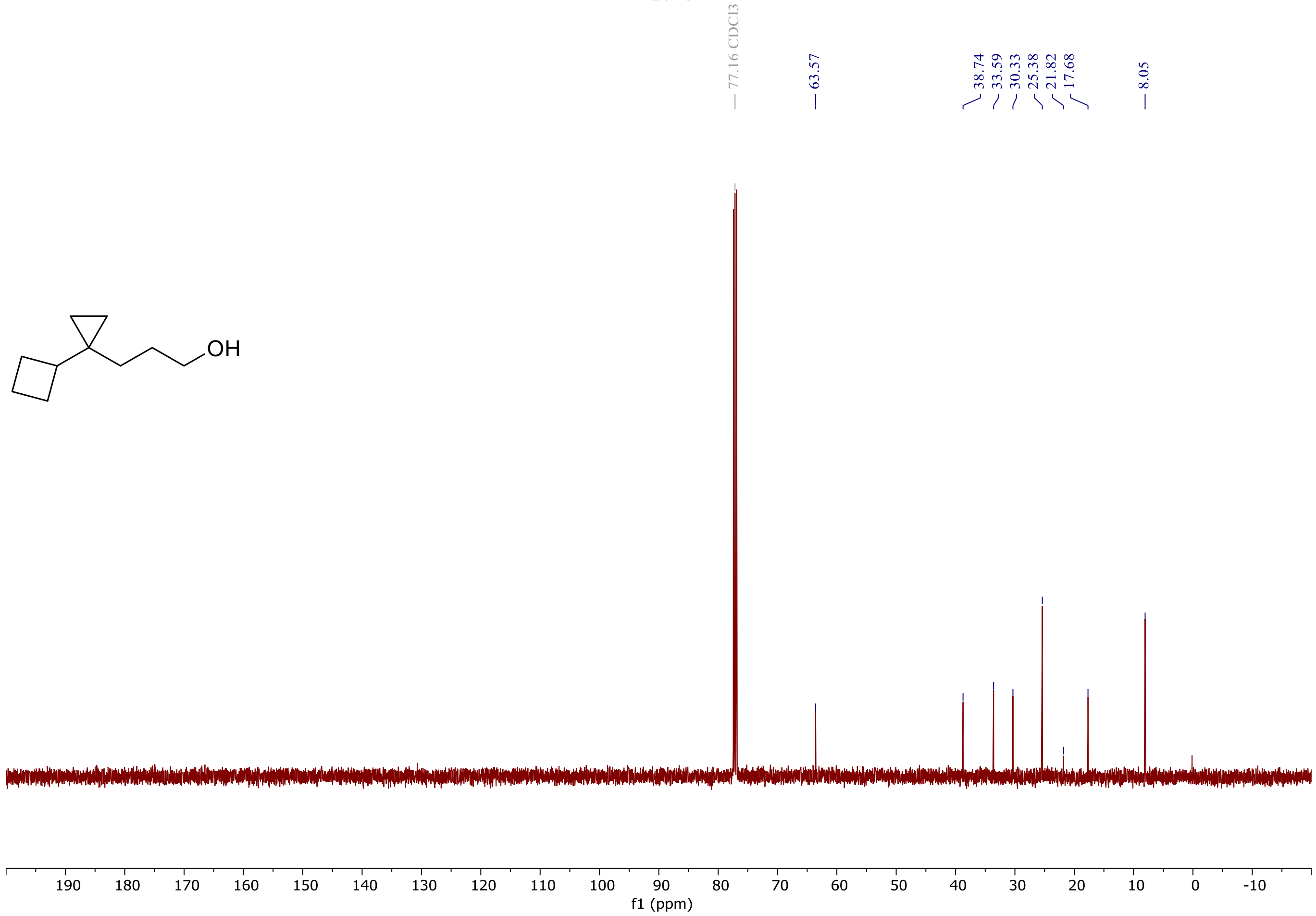

— 77.16 CDCl3
— 63.57
38.74
33.59
30.33
25.38
21.82
17.68
— 8.05
OH
190 180 170 160 150 140 130 120 110 100 90 80 70 60 50 40 30 20 10 0 -10
f1 (ppm)

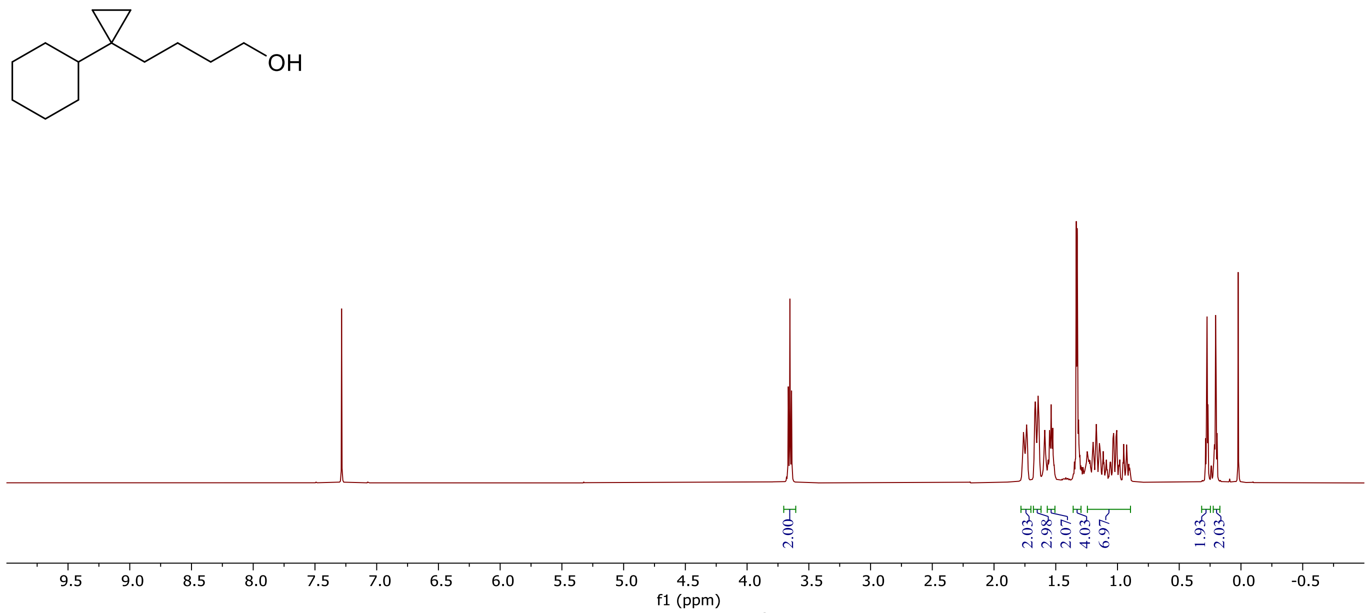
OH
2.00
2.03
2.98
2.07
4.03
6.97
1.93
2.03
f1 (ppm)

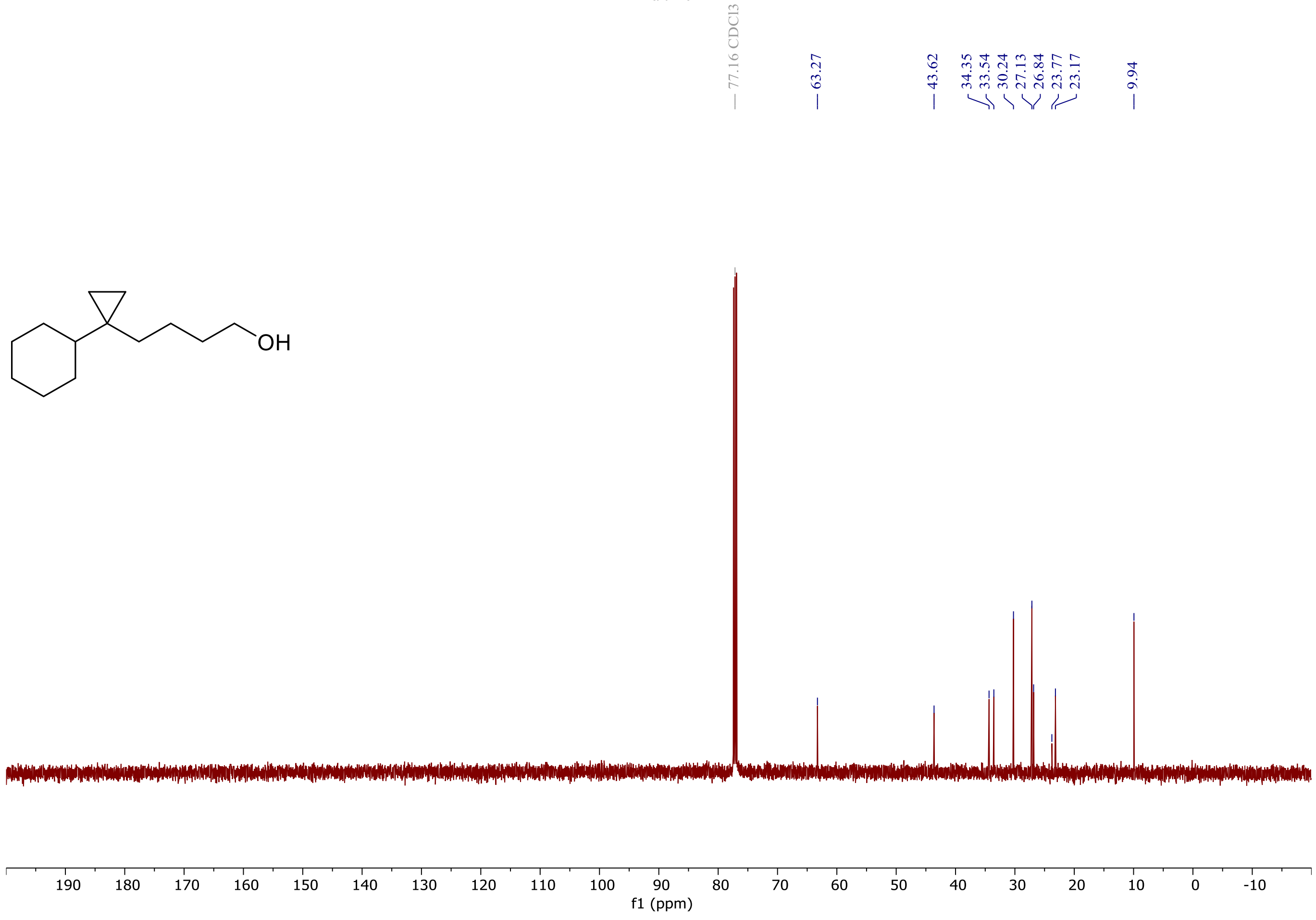
77.16 CDCl3
63.27
43.62
34.35
33.54
30.24
27.13
26.84
23.77
23.17
9.94
OH
f1 (ppm)

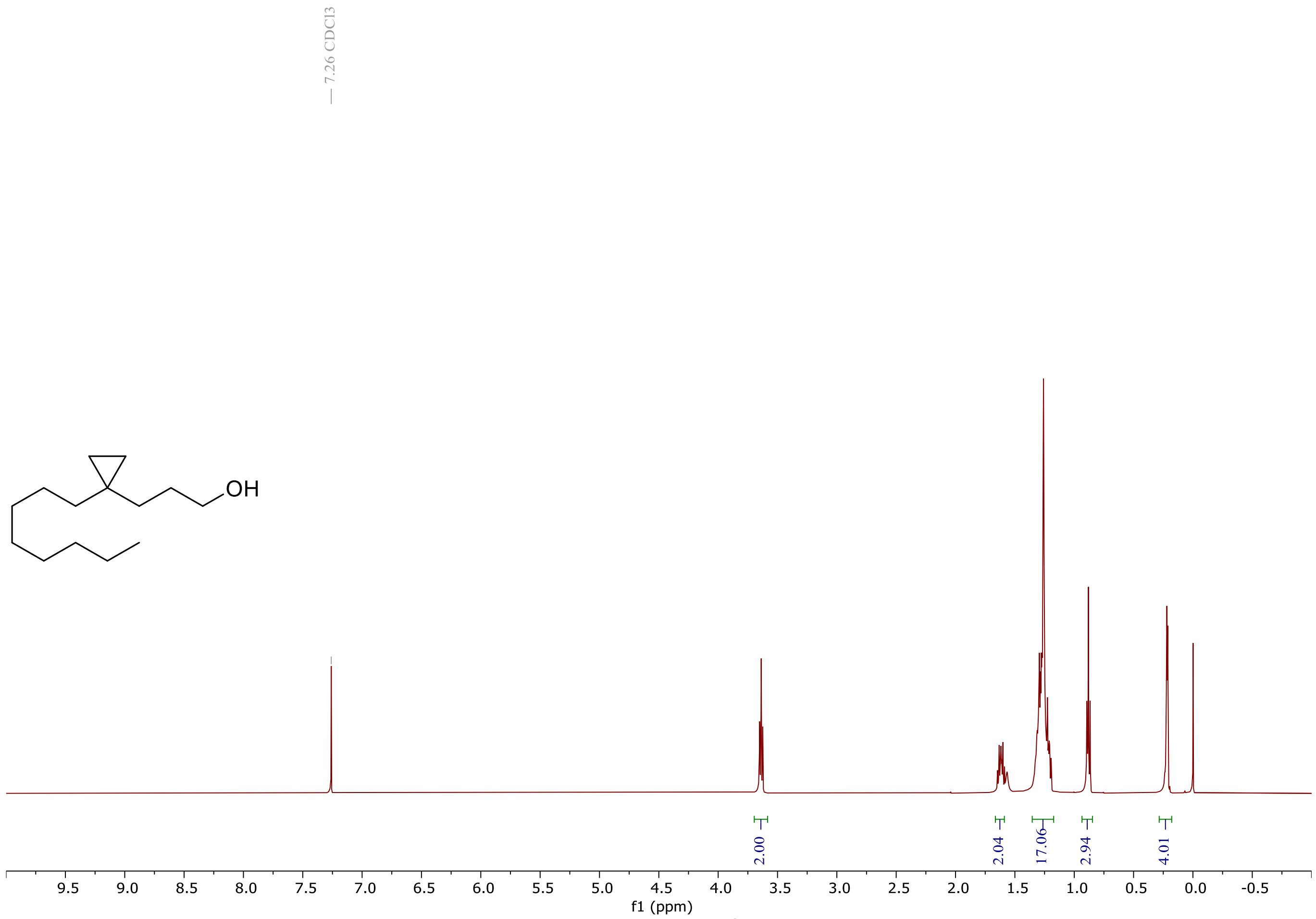
7.26 CDCl3
OH
2.00
2.04
17.06
2.94
4.01
9.5 9.0 8.5 8.0 7.5 7.0 6.5 6.0 5.5 5.0 4.5 4.0 3.5 3.0 2.5 2.0 1.5 1.0 0.5 0.0 -0.5
f1 (ppm)

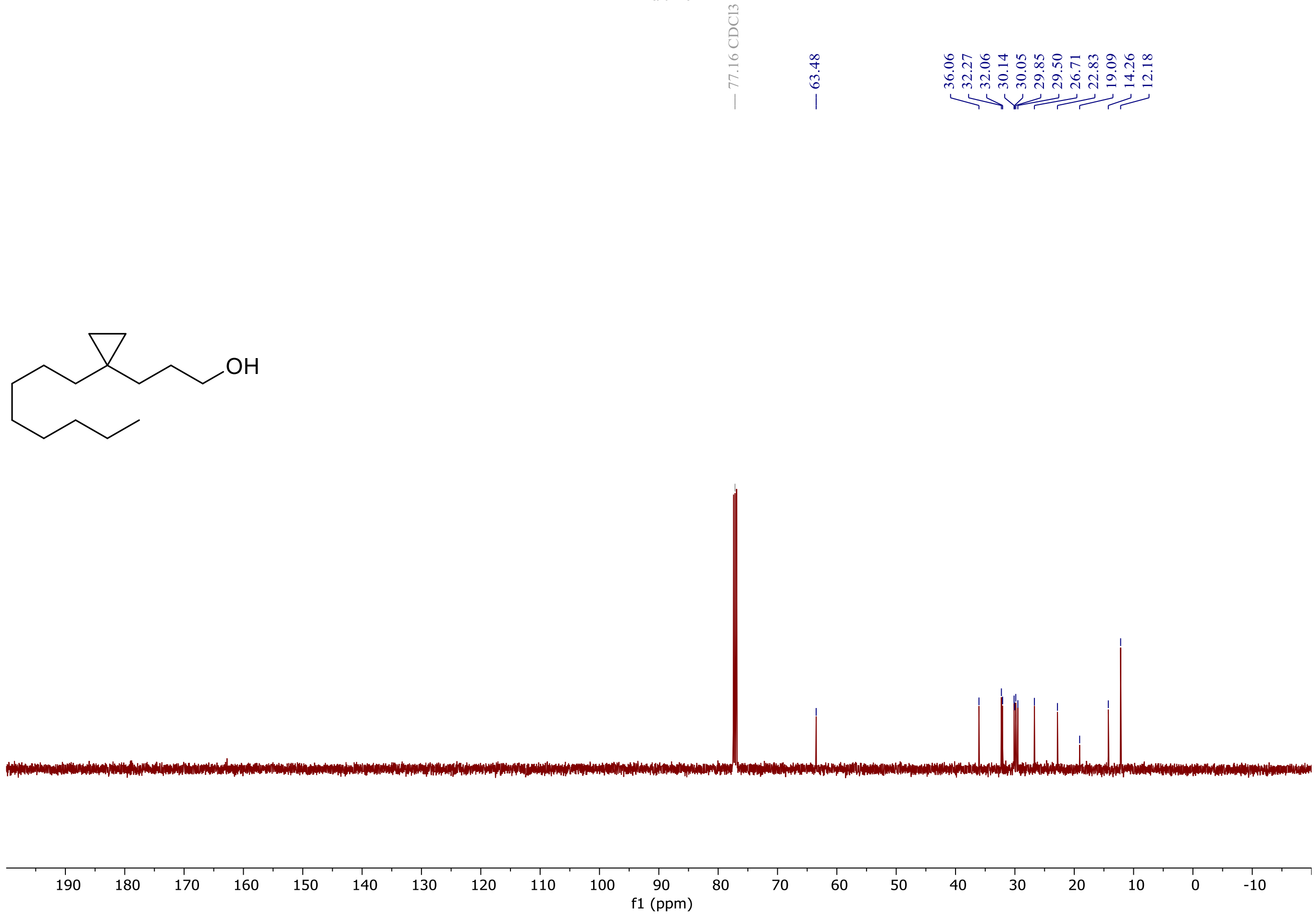
77.16 CDCl3
63.48
36.06
32.27
32.06
30.14
30.05
29.85
29.50
26.71
22.83
19.09
14.26
12.18
OH
190 180 170 160 150 140 130 120 110 100 90 80 70 60 50 40 30 20 10 0 -10
f1 (ppm)

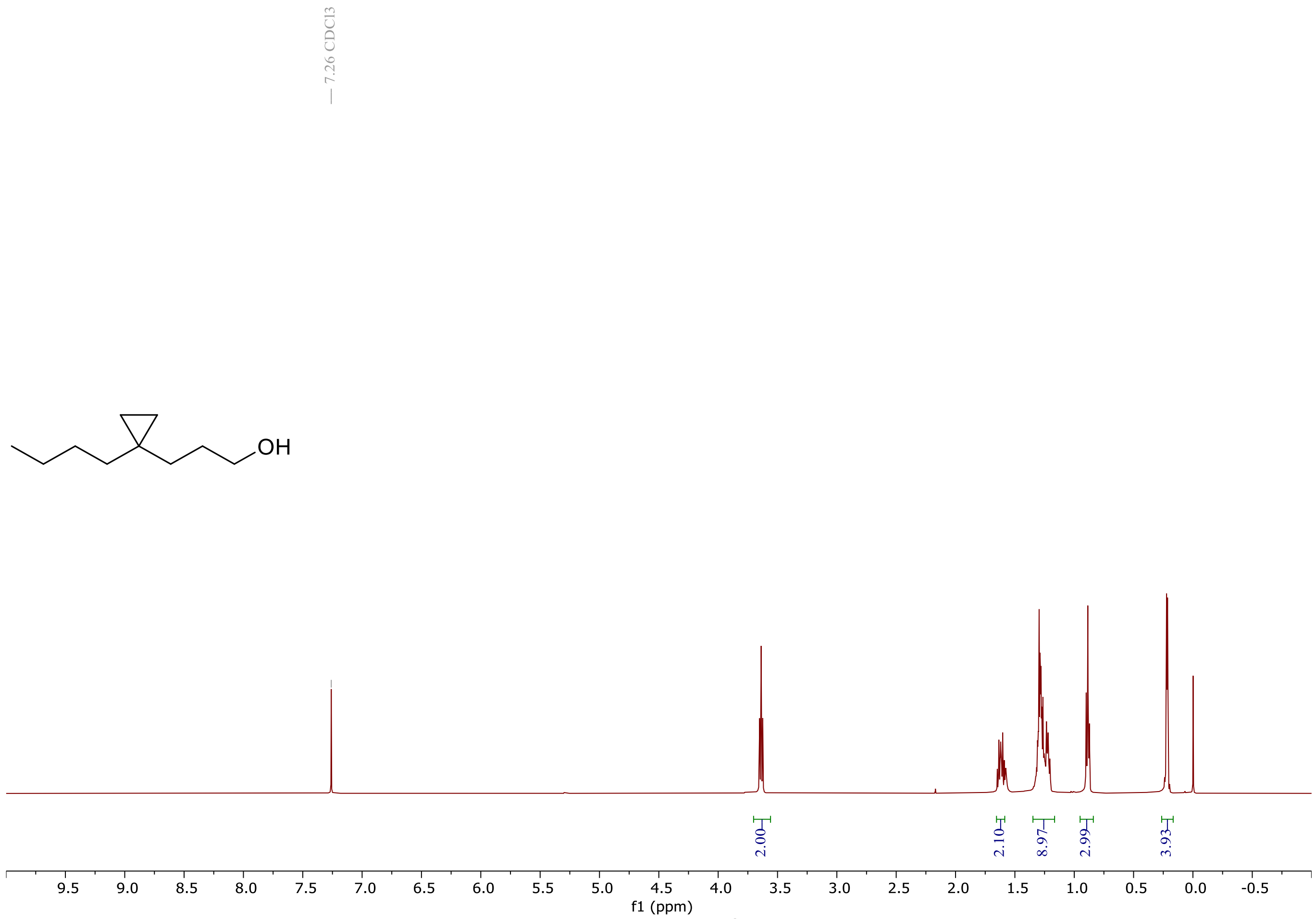
7.26 CDCl3
OH
2.00
2.10
8.97
2.99
3.93
f1 (ppm)

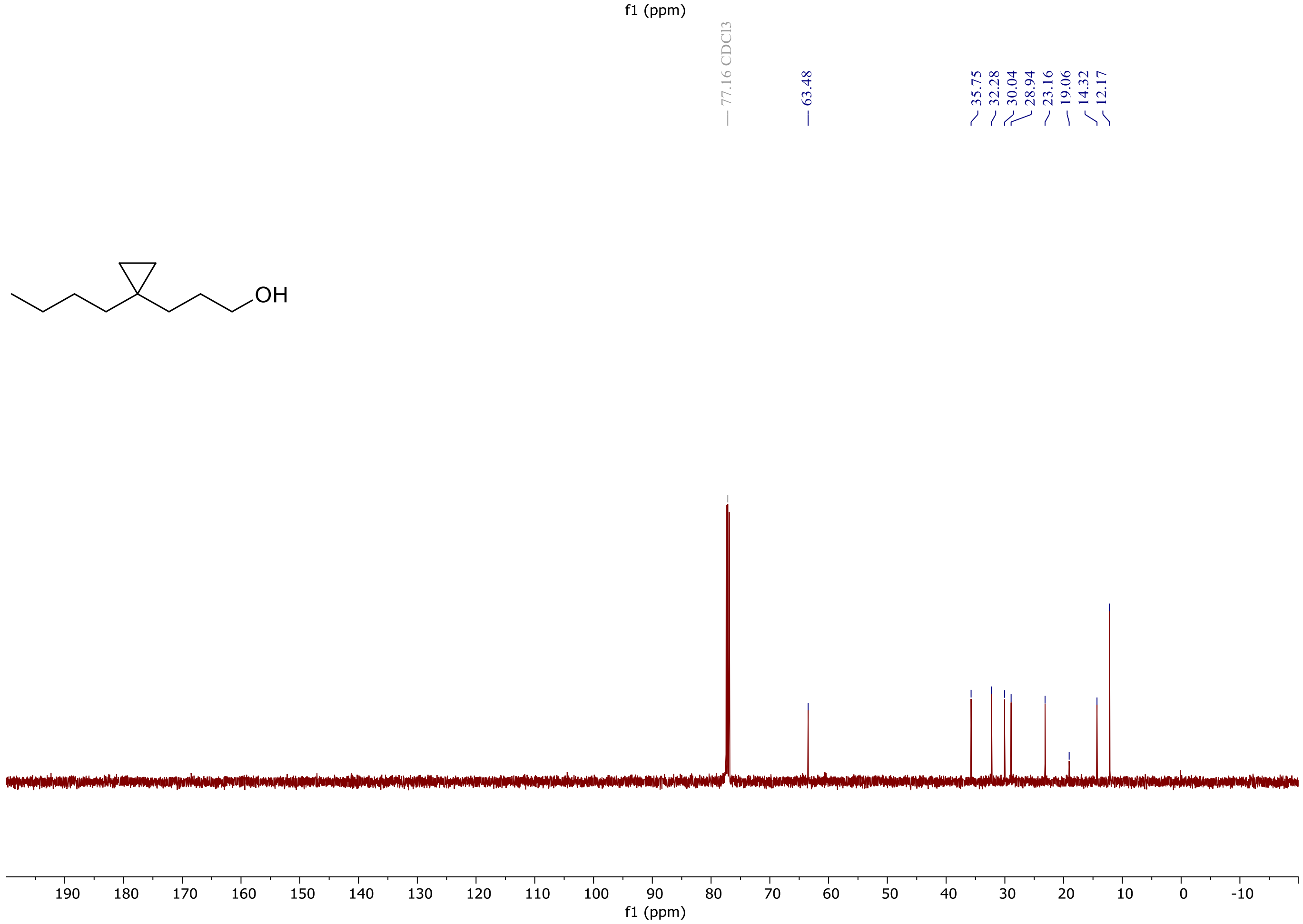
77.16 CDCl3
63.48
35.75
32.28
30.04
28.94
23.16
19.06
14.32
12.17
OH
f1 (ppm)

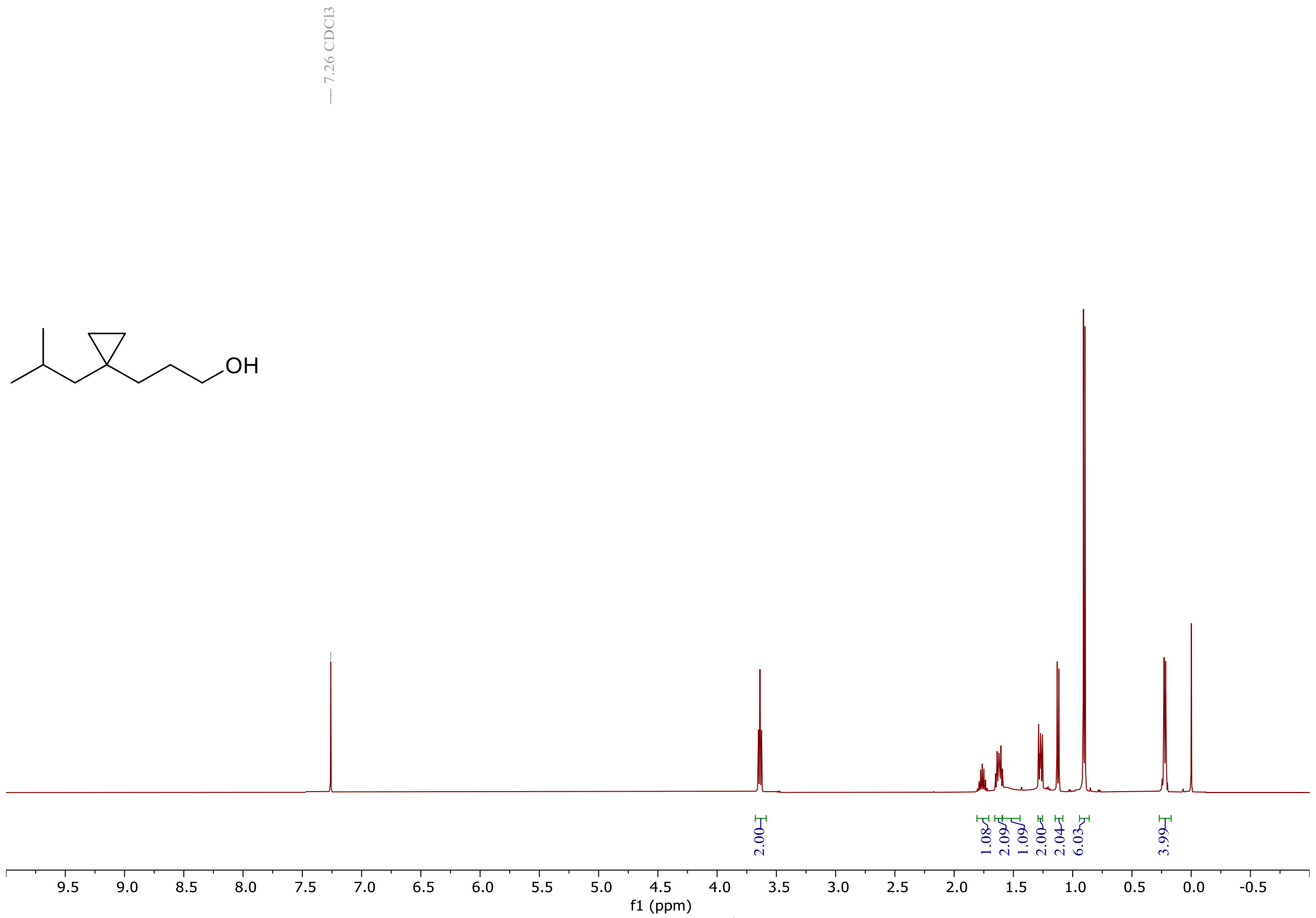
7.26 CDCl3
OH
2.00
1.08
2.09
1.09
2.00
2.04
6.03
3.99
9.5 9.0 8.5 8.0 7.5 7.0 6.5 6.0 5.5 5.0 4.5 4.0 3.5 3.0 2.5 2.0 1.5 1.0 0.5 0.0 -0.5
f1 (ppm)

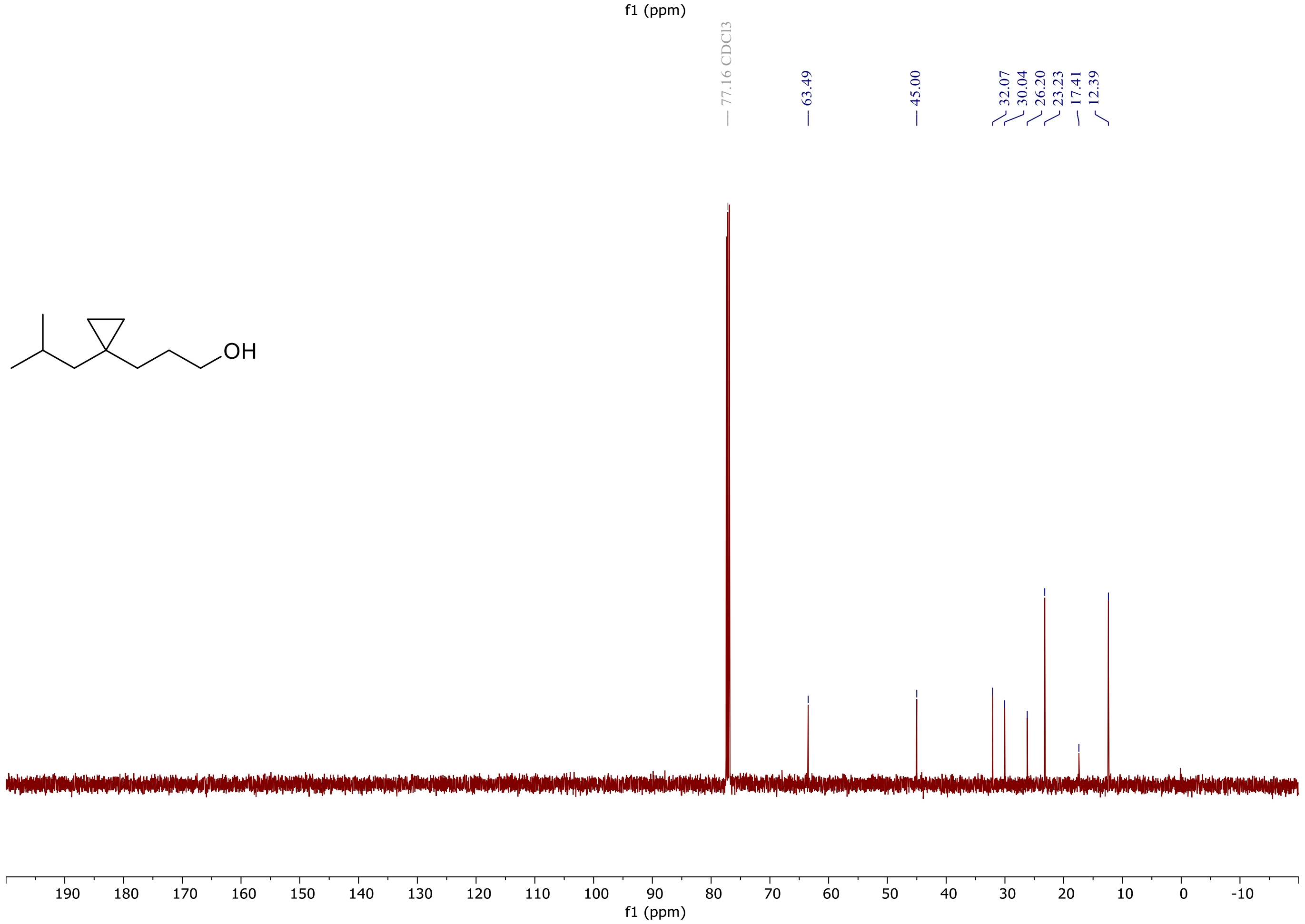
77.16 CDCl3
63.49
45.00
32.07
30.04
26.20
23.23
17.41
12.39
OH
190 180 170 160 150 140 130 120 110 100 90 80 70 60 50 40 30 20 10 0 -10
f1 (ppm)

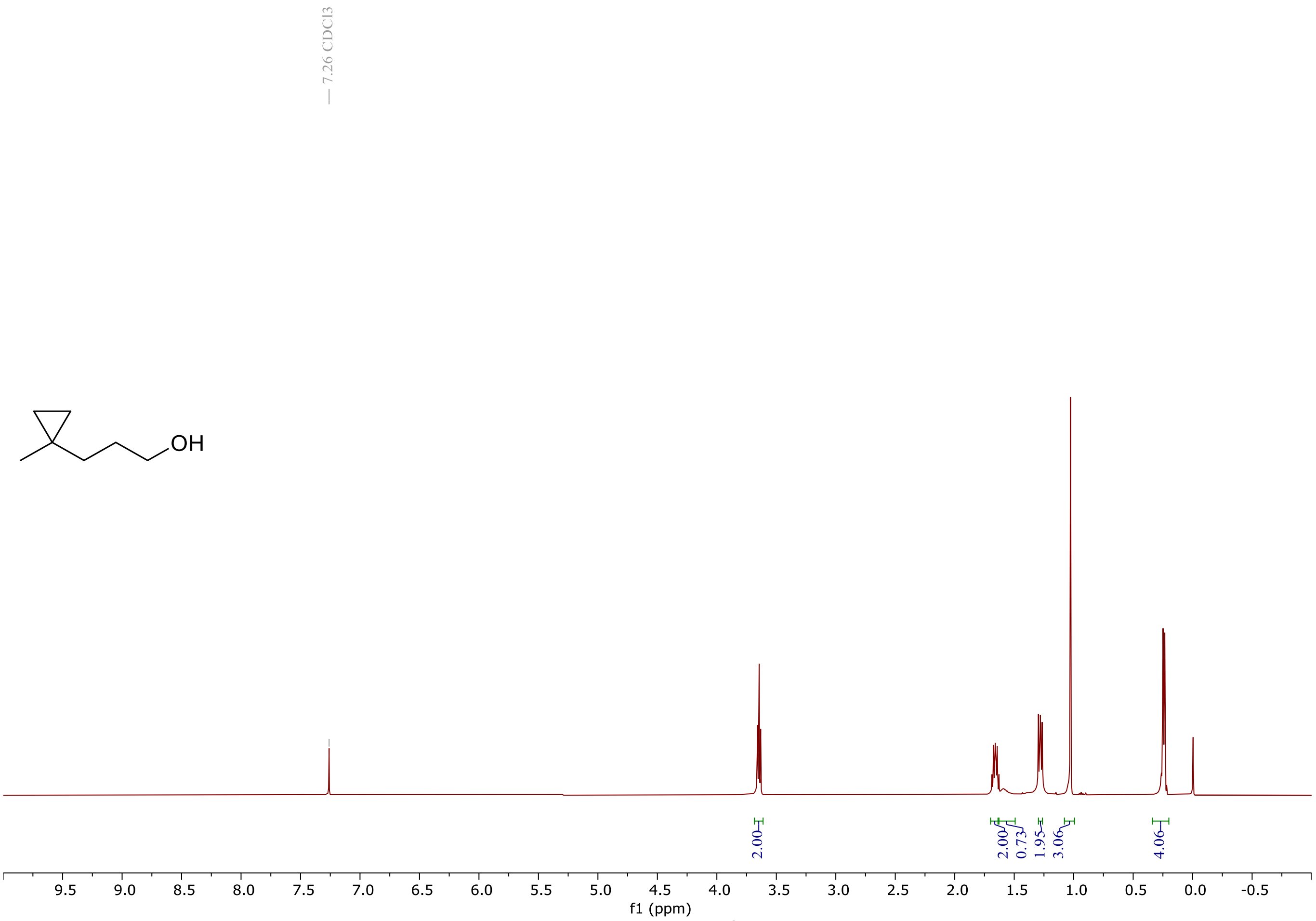
7.26 CDCl3
OH
2.00
2.00
0.73
1.95
3.06
4.06
9.5
9.0
8.5
8.0
7.5
7.0
6.5
6.0
5.5
5.0
4.5
4.0
3.5
3.0
2.5
2.0
1.5
1.0
0.5
0.0
-0.5
f1 (ppm)

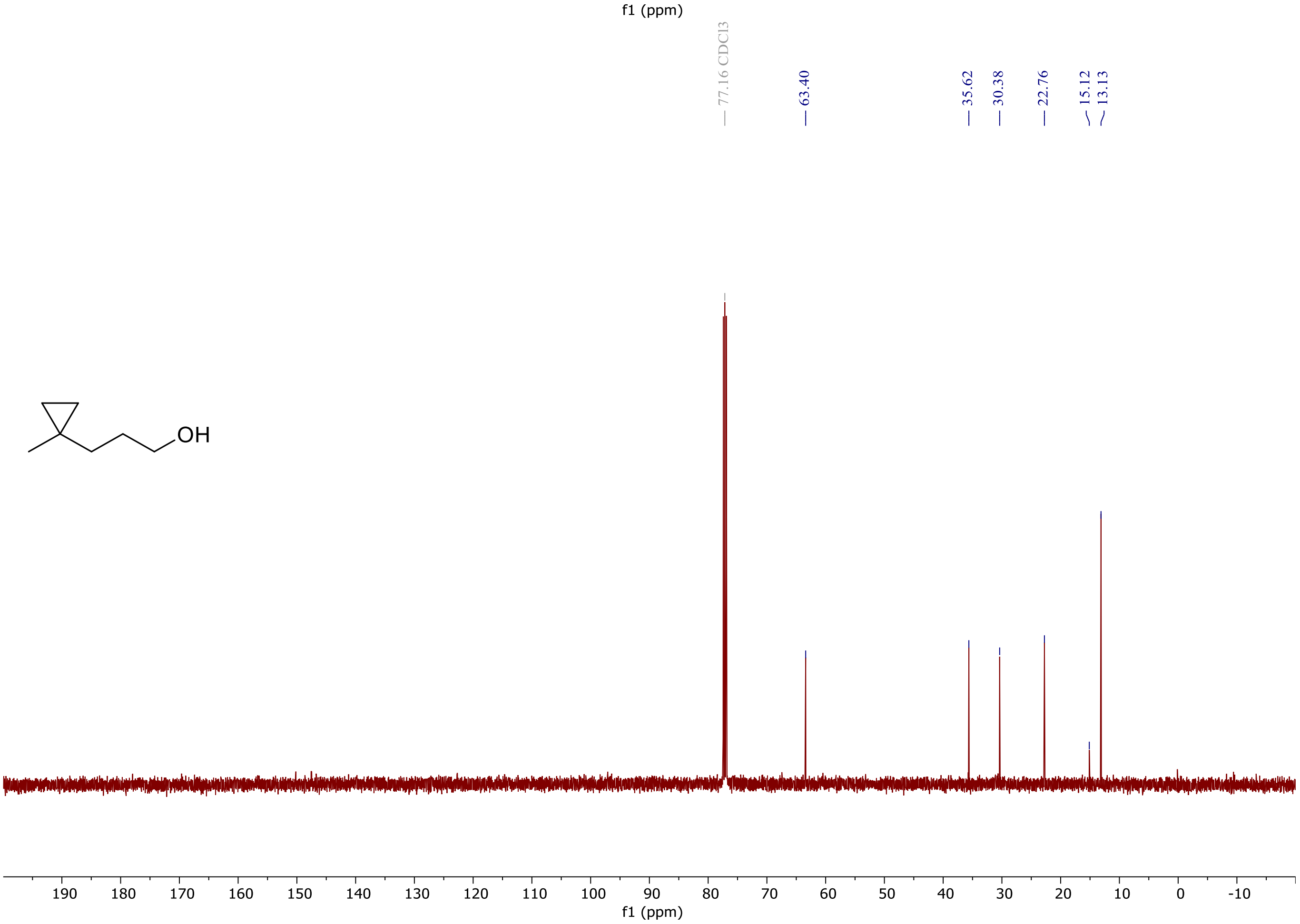
77.16 CDCl3
63.40
35.62
30.38
22.76
15.12
13.13
OH
190
180
170
160
150
140
130
120
110
100
90
80
70
60
50
40
30
20
10
0
-10
f1 (ppm)

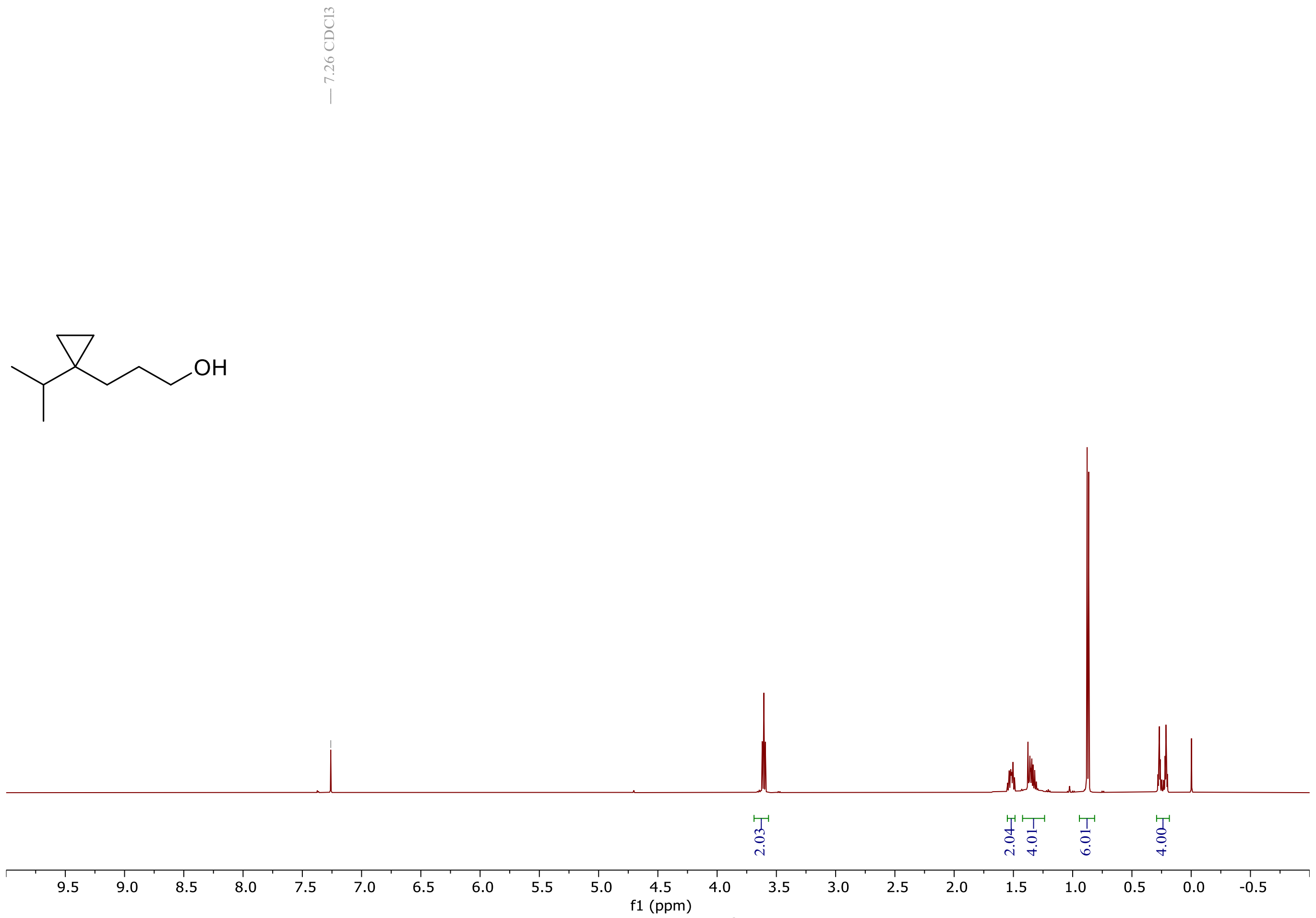
7.26 CDCl3
OH
2.03
2.04
4.01
6.01
4.00
9.5 9.0 8.5 8.0 7.5 7.0 6.5 6.0 5.5 5.0 4.5 4.0 3.5 3.0 2.5 2.0 1.5 1.0 0.5 0.0 -0.5
f1 (ppm)

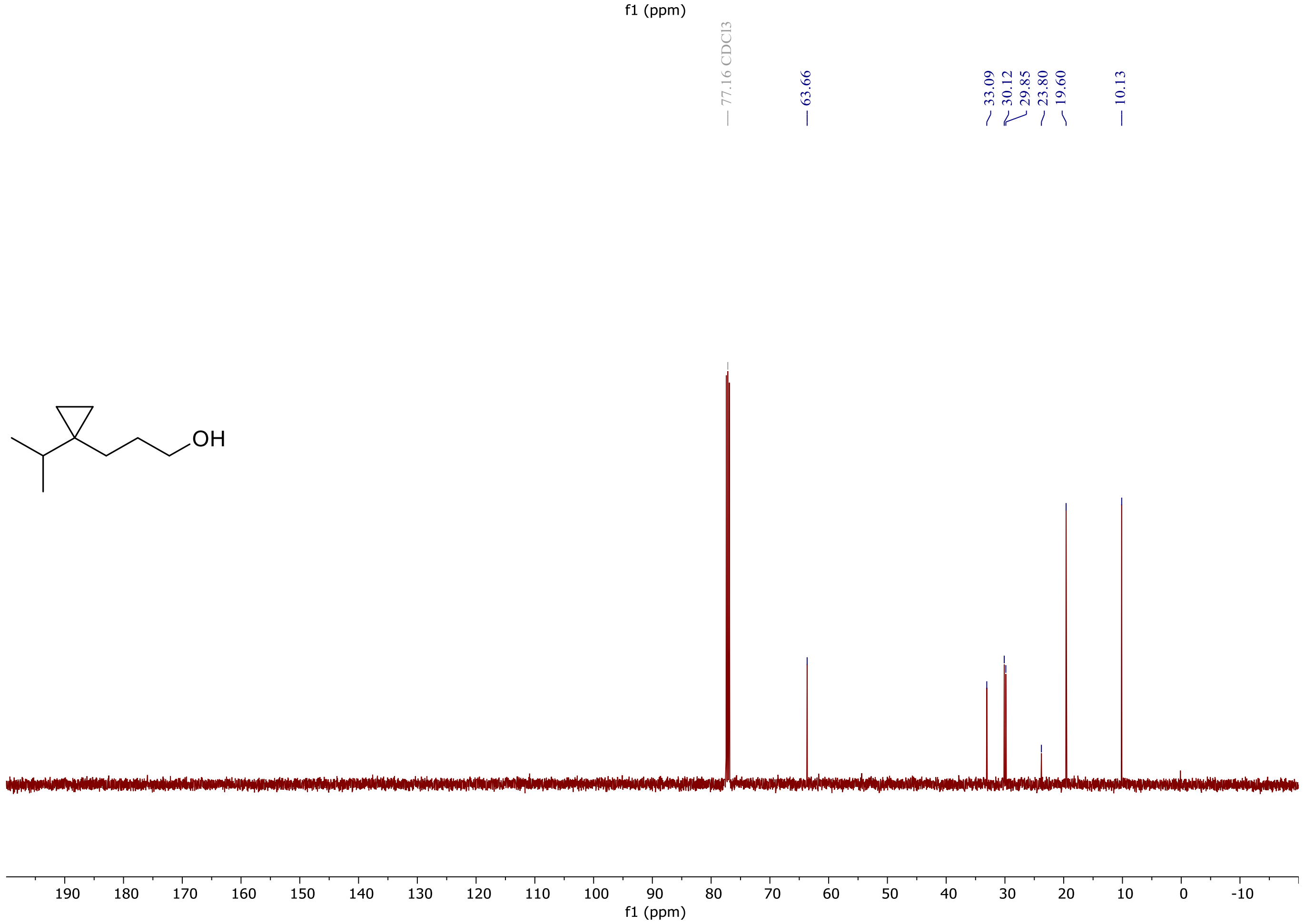
77.16 CDCl3
63.66
33.09
30.12
29.85
23.80
19.60
10.13
OH
190 180 170 160 150 140 130 120 110 100 90 80 70 60 50 40 30 20 10 0 -10
f1 (ppm)

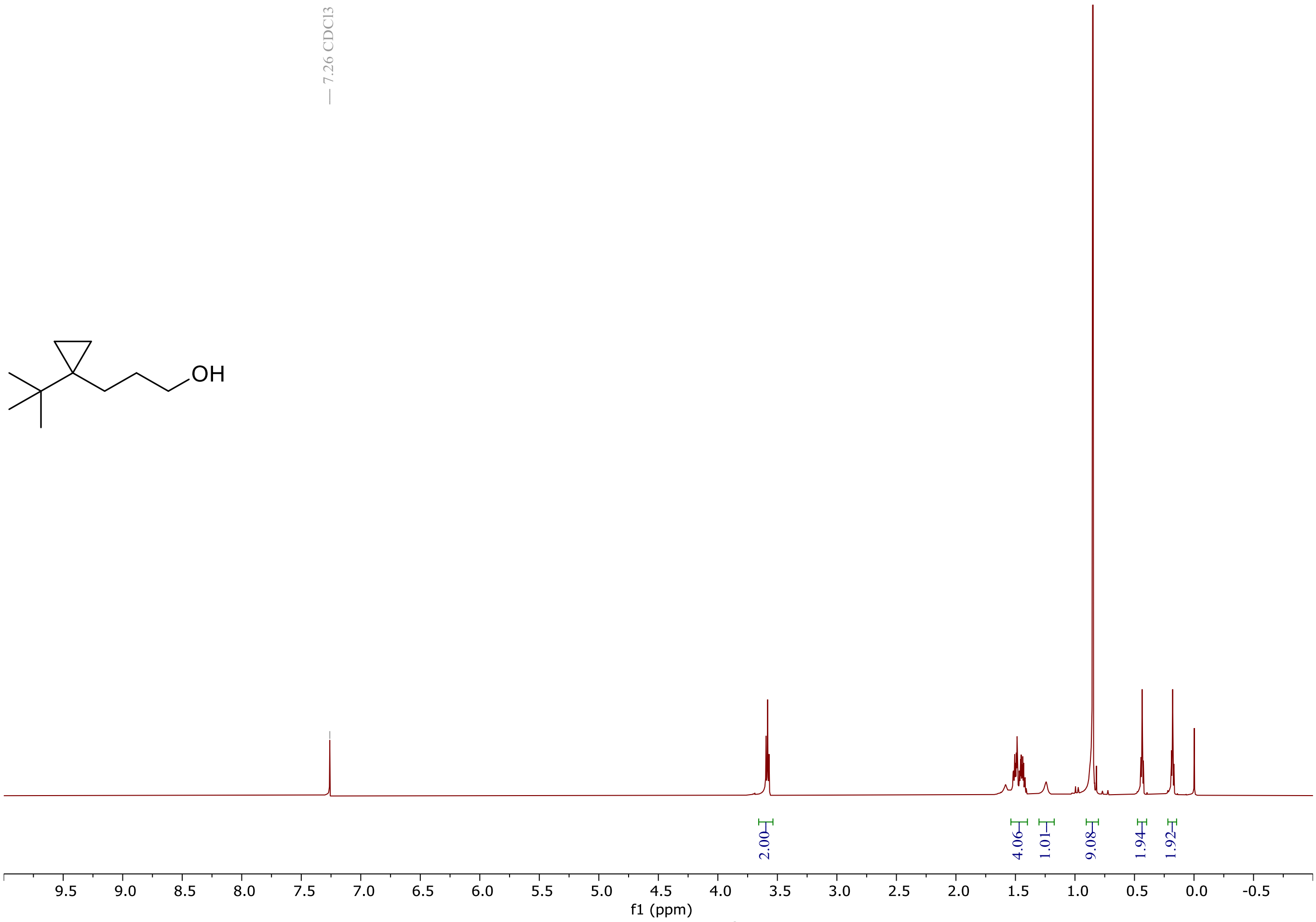
7.26 CDCl3
OH
2.00
4.06
1.01
9.08
1.94
1.92
f1 (ppm)

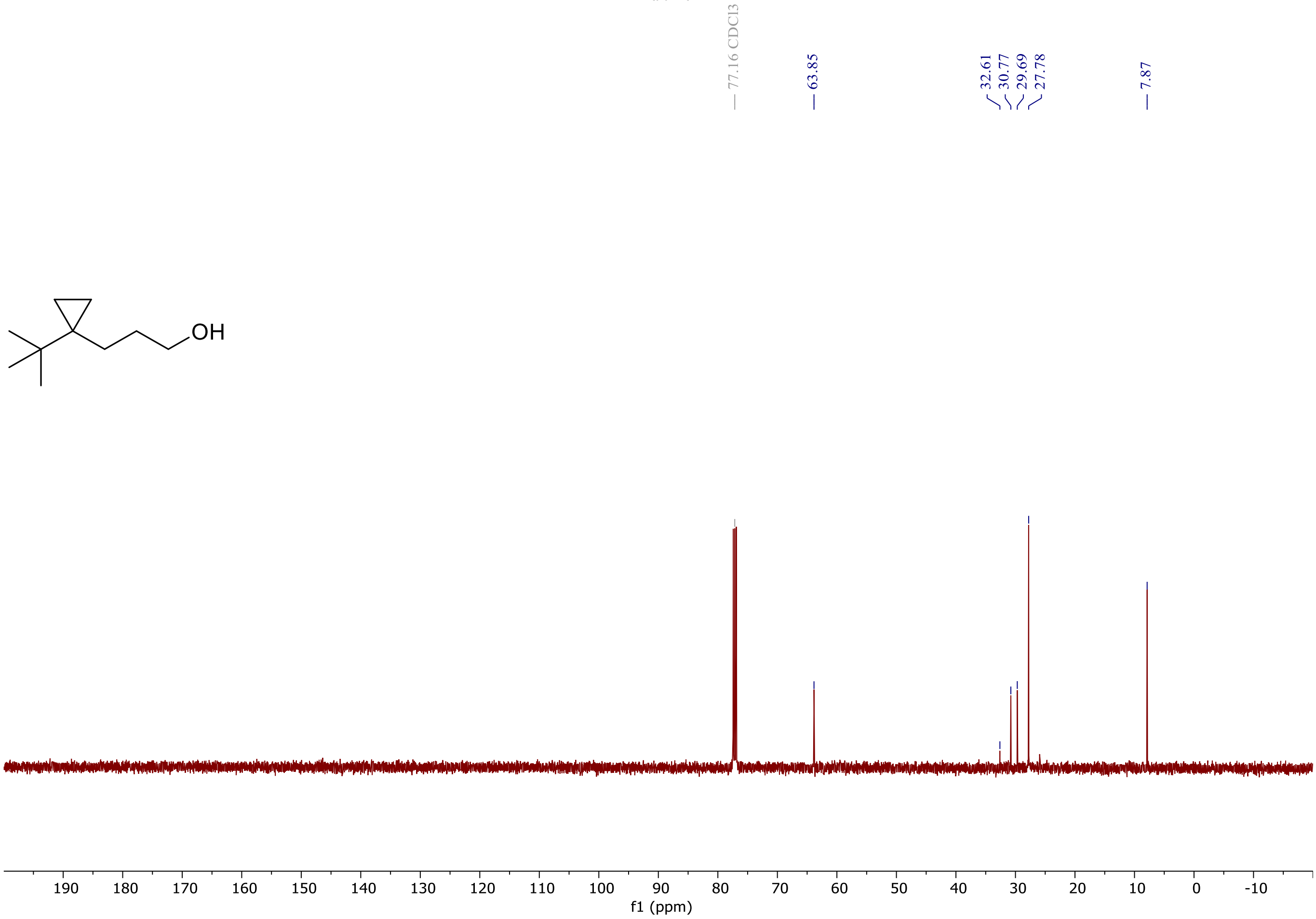
77.16 CDCl3
63.85
32.61
30.77
29.69
27.78
7.87
OH
f1 (ppm)

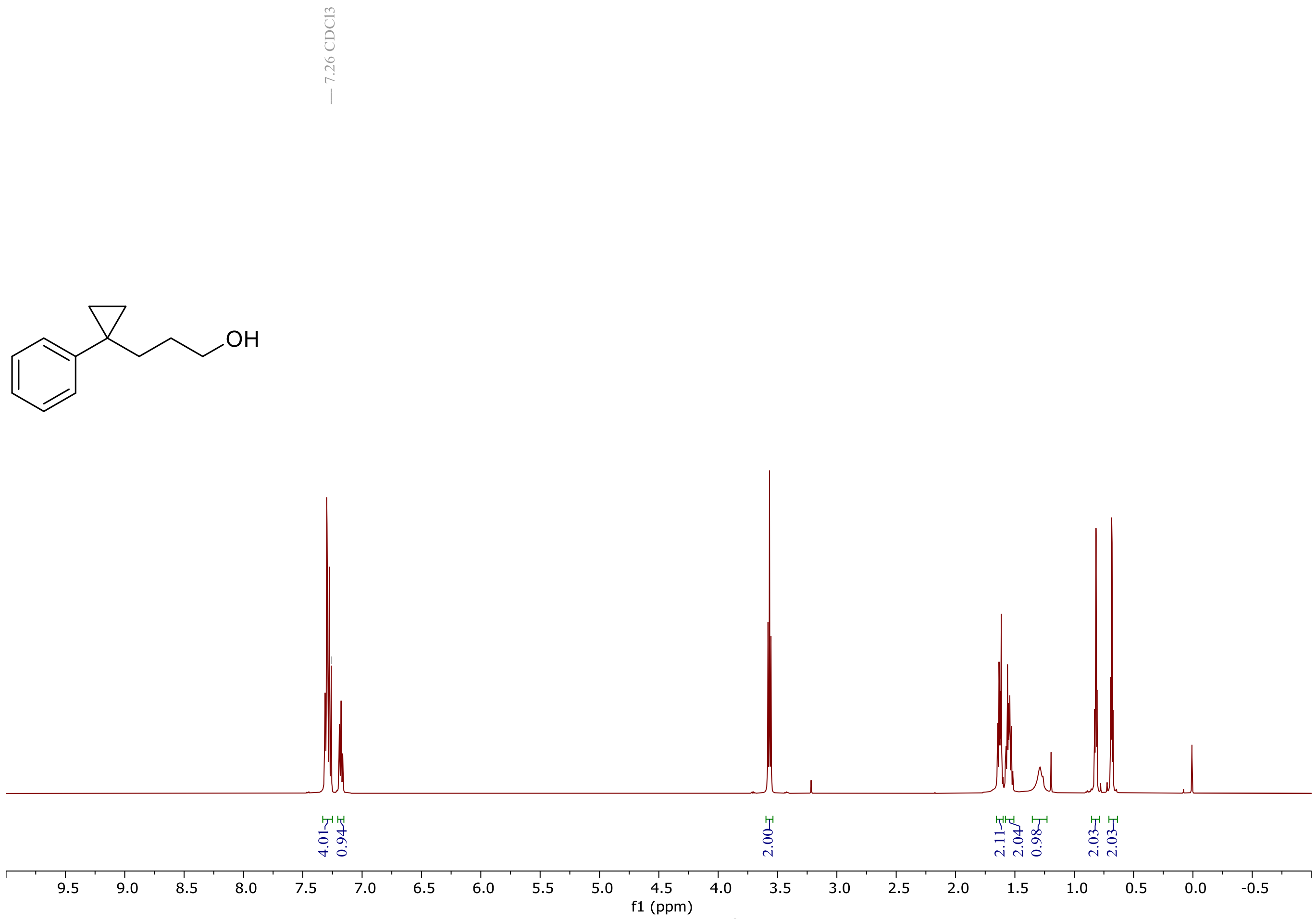
7.26 CDCl3
OH
4.01
0.94
2.00
2.11
2.04
0.98
2.03
2.03
9.5 9.0 8.5 8.0 7.5 7.0 6.5 6.0 5.5 5.0 4.5 4.0 3.5 3.0 2.5 2.0 1.5 1.0 0.5 0.0 -0.5
f1 (ppm)

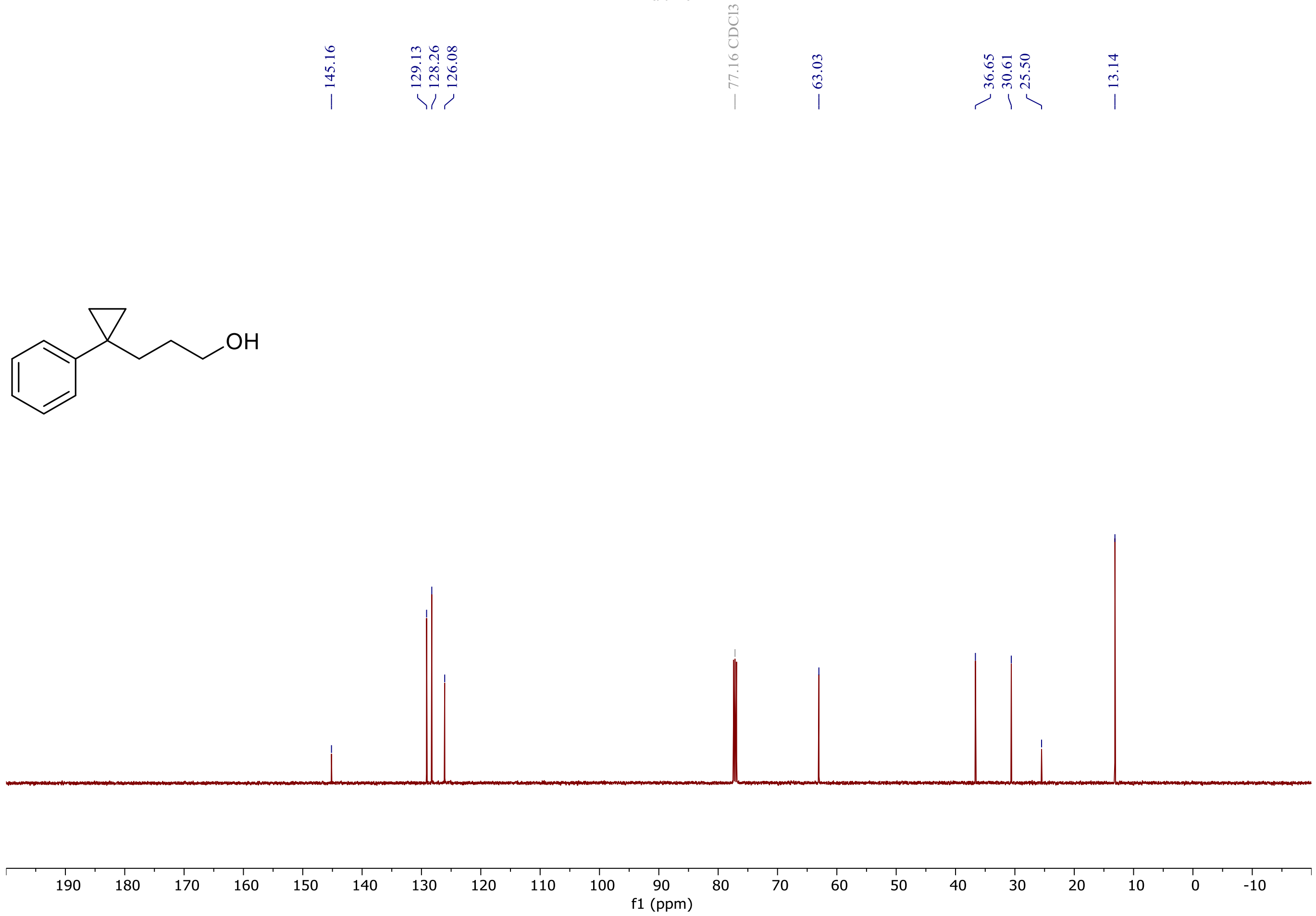
145.16
129.13
128.26
126.08
77.16 CDCl3
63.03
36.65
30.61
25.50
13.14
OH
190 180 170 160 150 140 130 120 110 100 90 80 70 60 50 40 30 20 10 0 -10
f1 (ppm)

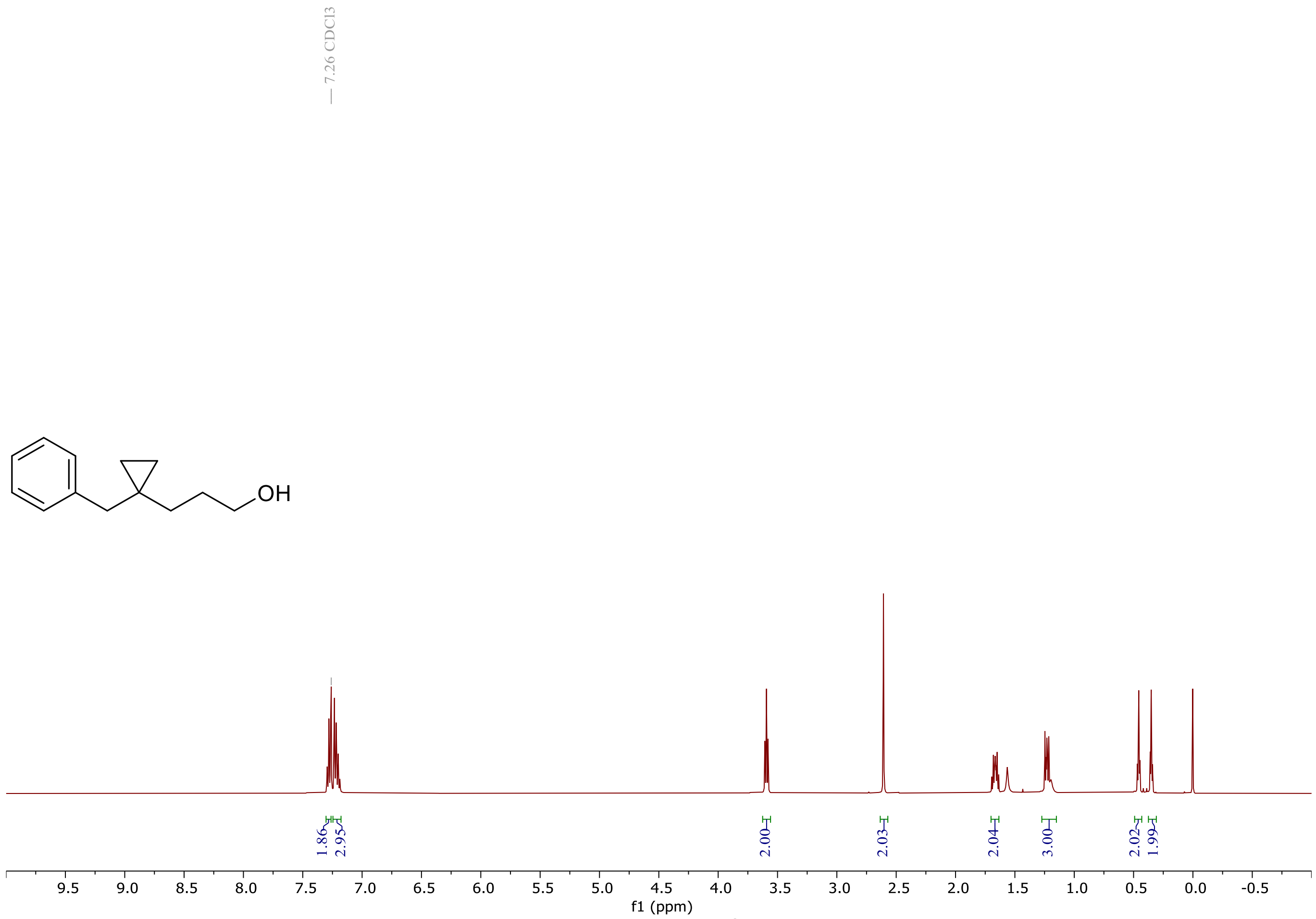
7.26 CDCl3
OH
1.86
2.95
2.00
2.03
2.04
3.00
2.02
1.99
9.5 9.0 8.5 8.0 7.5 7.0 6.5 6.0 5.5 5.0 4.5 4.0 3.5 3.0 2.5 2.0 1.5 1.0 0.5 0.0 -0.5
f1 (ppm)

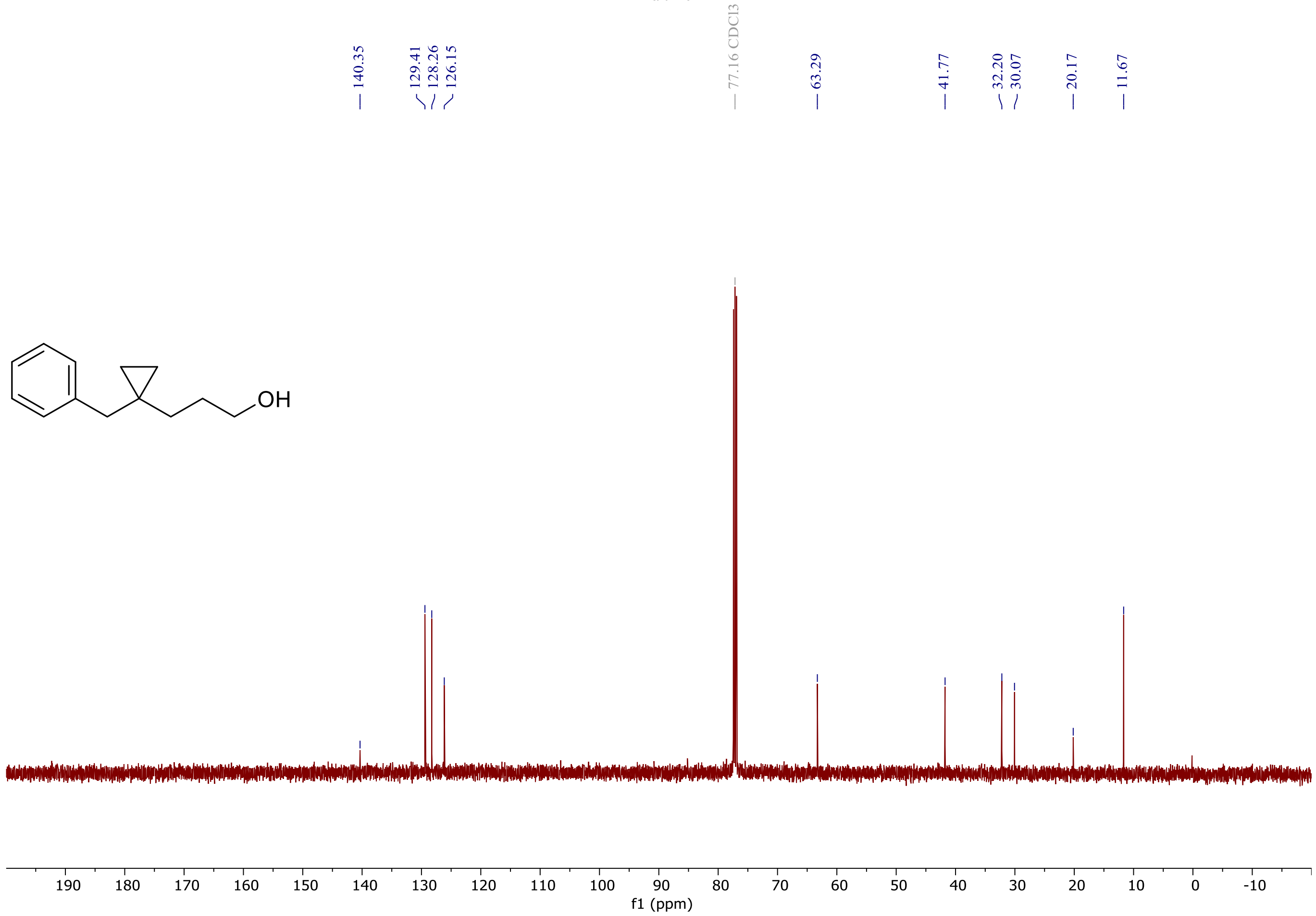
140.35
129.41
128.26
126.15
77.16 CDCl3
63.29
41.77
32.20
30.07
20.17
11.67
OH
190 180 170 160 150 140 130 120 110 100 90 80 70 60 50 40 30 20 10 0 -10
f1 (ppm)

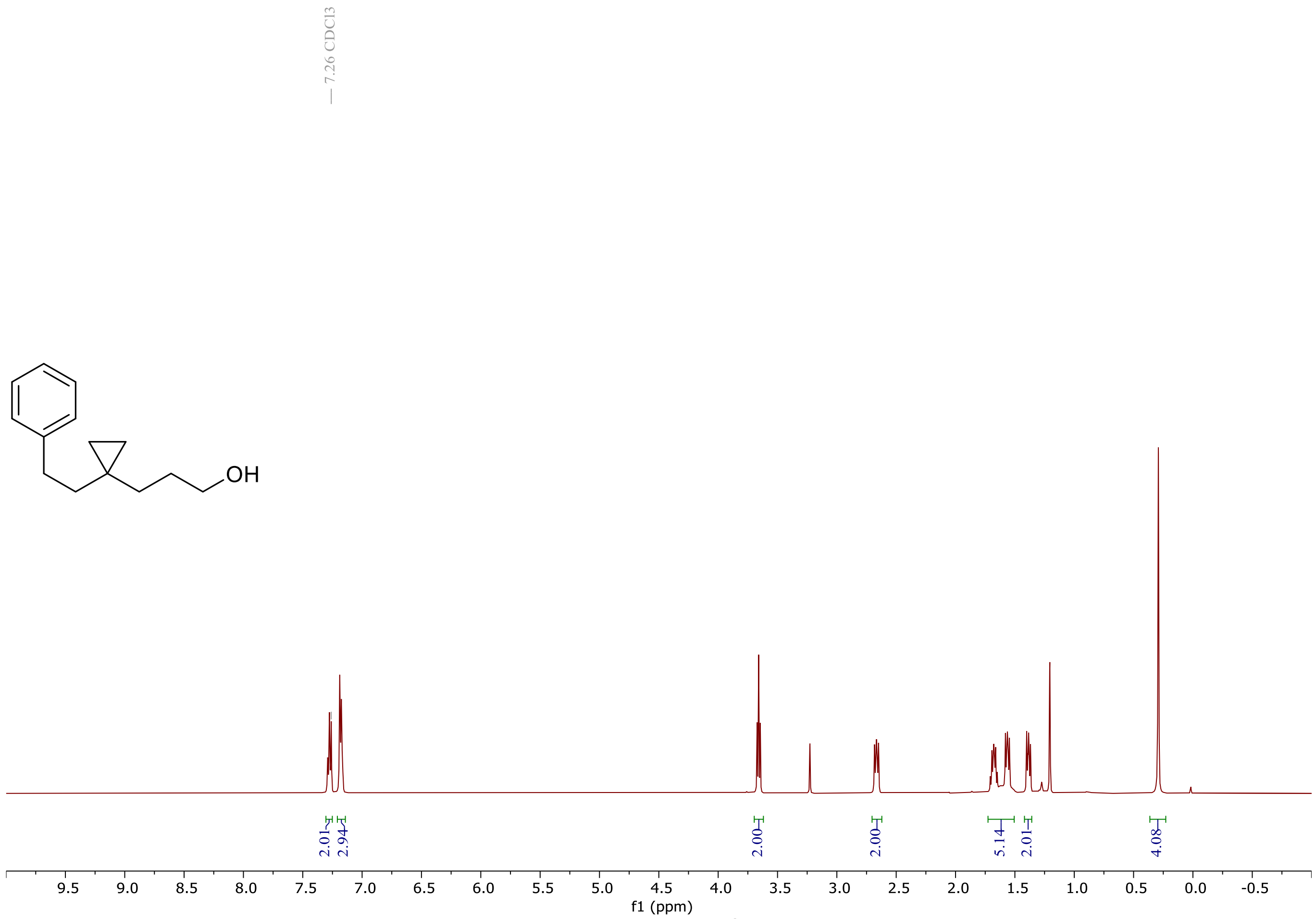
7.26 CDCl3
OH
2.01
2.94
2.00
2.00
5.14
2.01
4.08
9.5 9.0 8.5 8.0 7.5 7.0 6.5 6.0 5.5 5.0 4.5 4.0 3.5 3.0 2.5 2.0 1.5 1.0 0.5 0.0 -0.5
f1 (ppm)

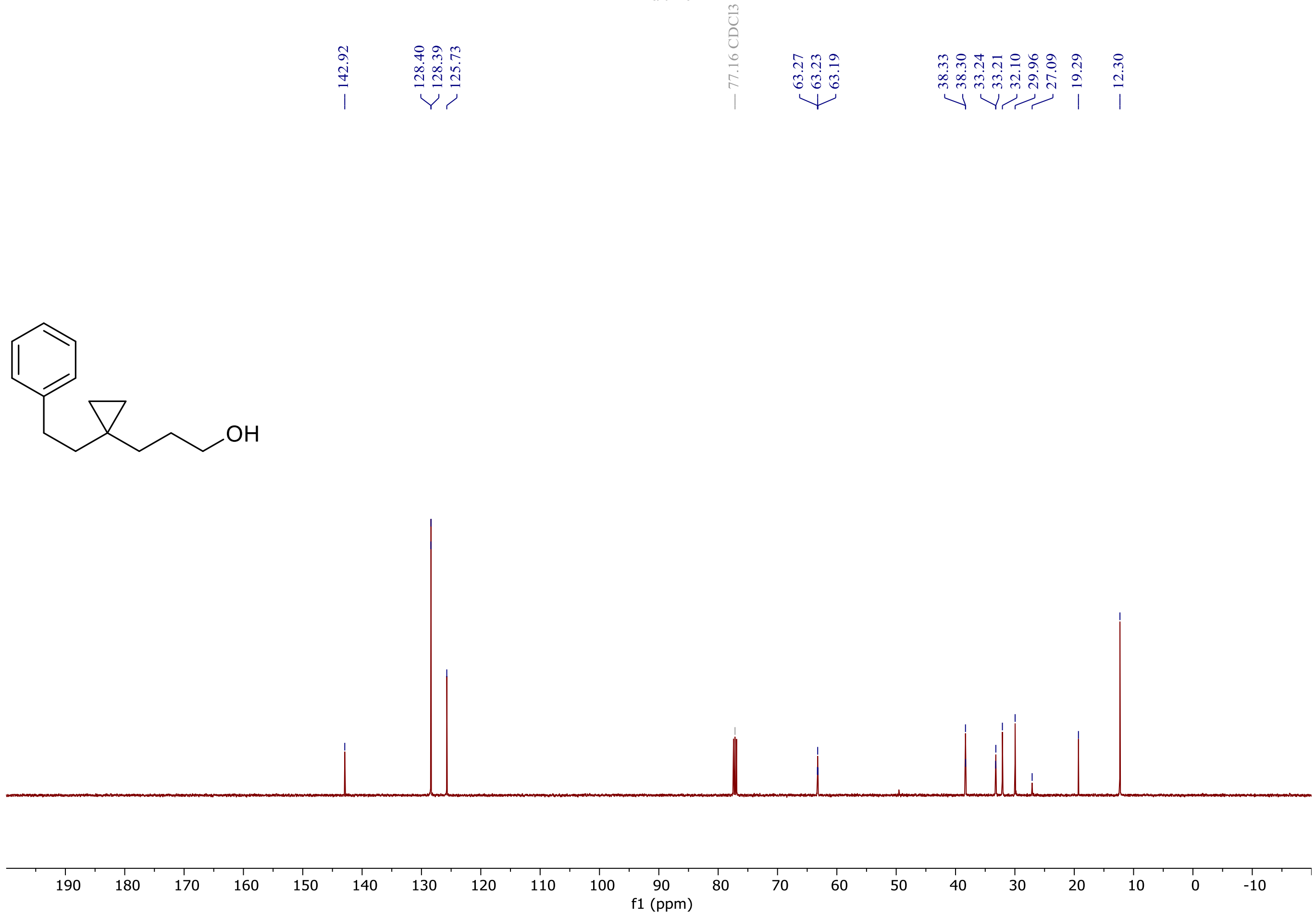
142.92
128.40
128.39
125.73
77.16 CDCl3
63.27
63.23
63.19
38.33
38.30
33.24
33.21
32.10
29.96
27.09
19.29
12.30
OH
190 180 170 160 150 140 130 120 110 100 90 80 70 60 50 40 30 20 10 0 -10
f1 (ppm)

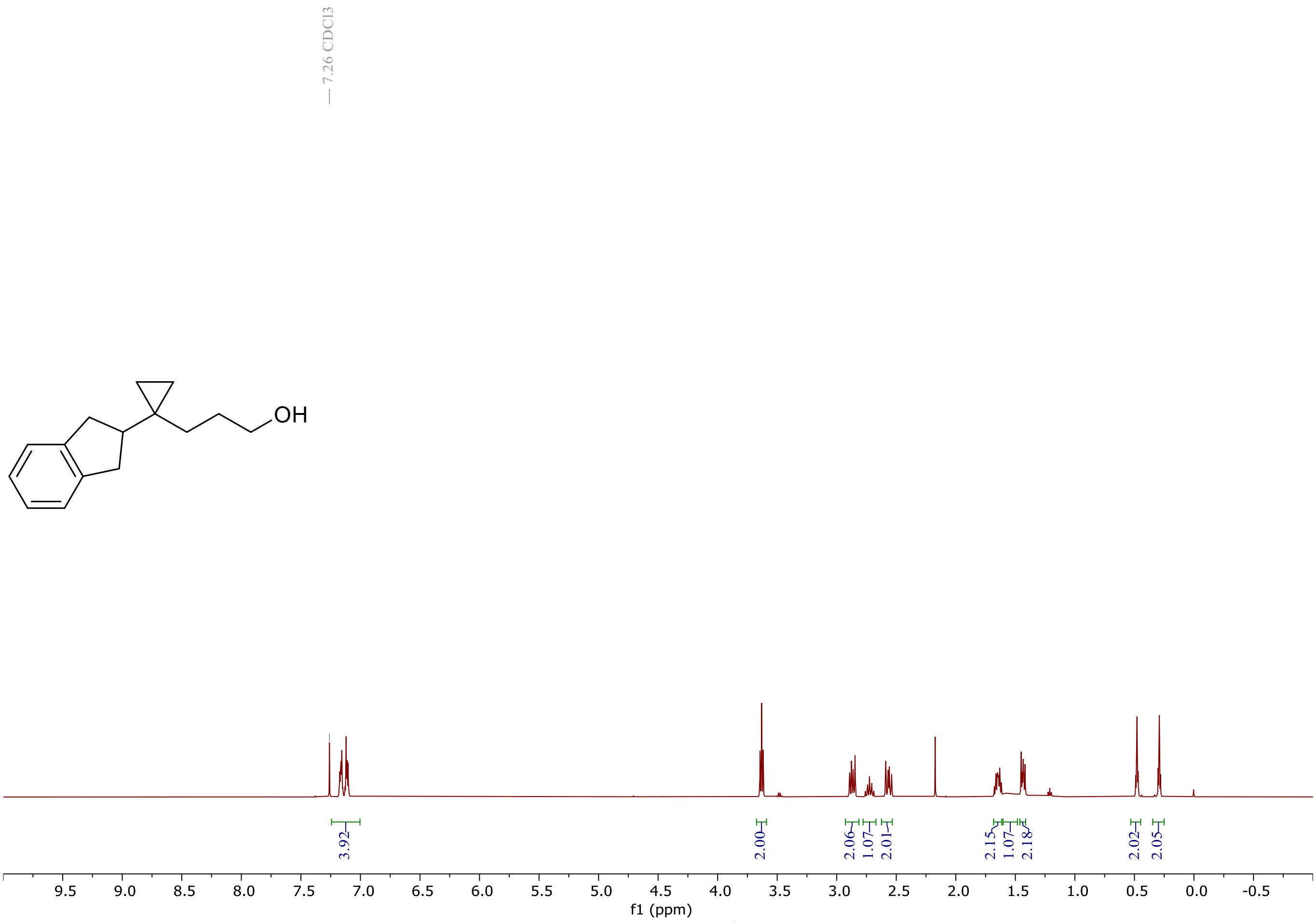

7.26 CDCl3
OH
3.92
2.00
2.06
1.07
2.01
2.15
1.07
2.18
2.02
2.05
9.5 9.0 8.5 8.0 7.5 7.0 6.5 6.0 5.5 5.0 4.5 4.0 3.5 3.0 2.5 2.0 1.5 1.0 0.5 0.0 -0.5
f1 (ppm)

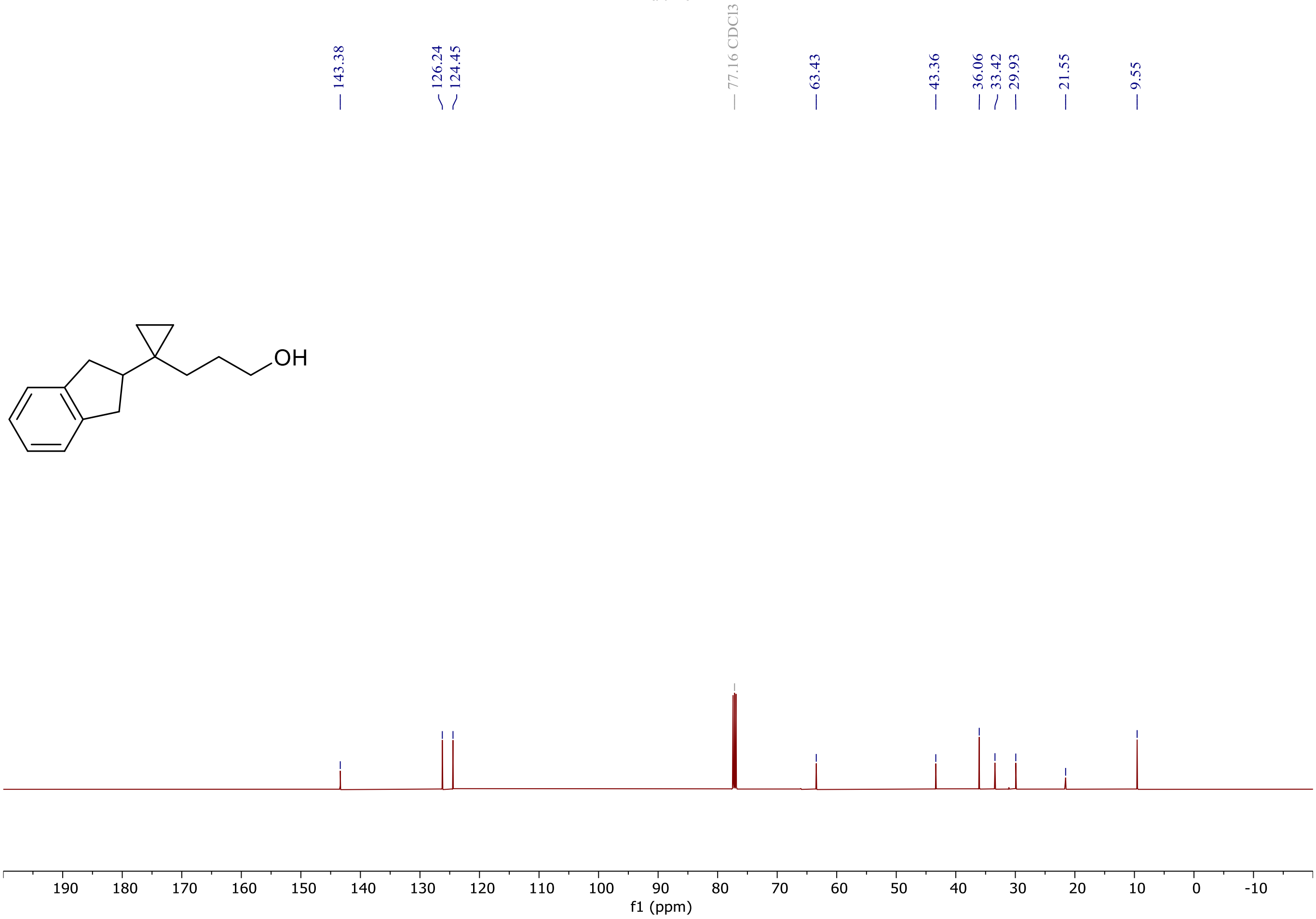

143.38
126.24
124.45
77.16 CDCl3
63.43
43.36
36.06
33.42
29.93
21.55
9.55
OH
190 180 170 160 150 140 130 120 110 100 90 80 70 60 50 40 30 20 10 0 -10
f1 (ppm)

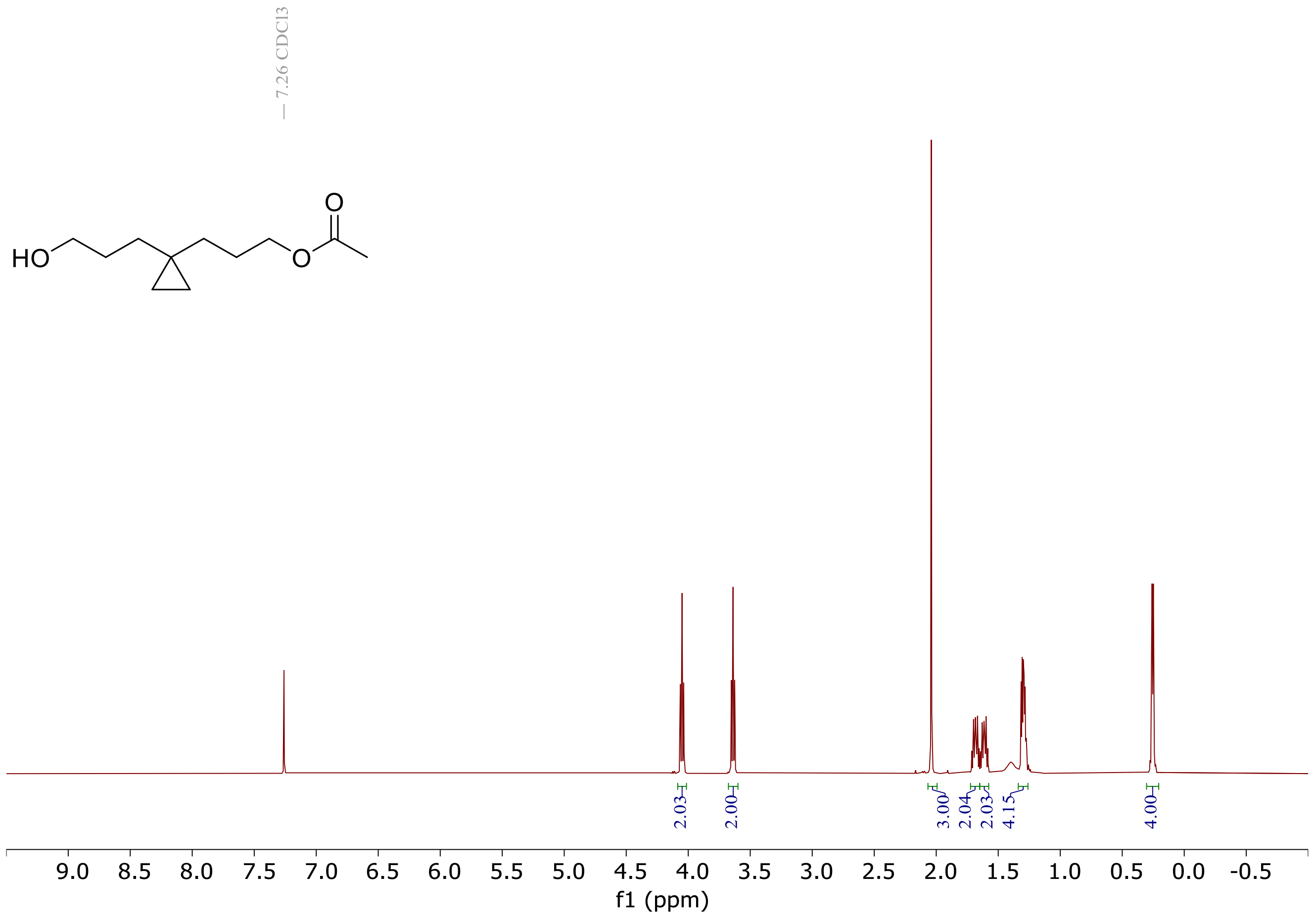
7.26 CDCl3
HO
O
O
2.03
2.00
3.00
2.04
2.03
4.15
4.00
9.0 8.5 8.0 7.5 7.0 6.5 6.0 5.5 5.0 4.5 4.0 3.5 3.0 2.5 2.0 1.5 1.0 0.5 0.0 -0.5
f1 (ppm)

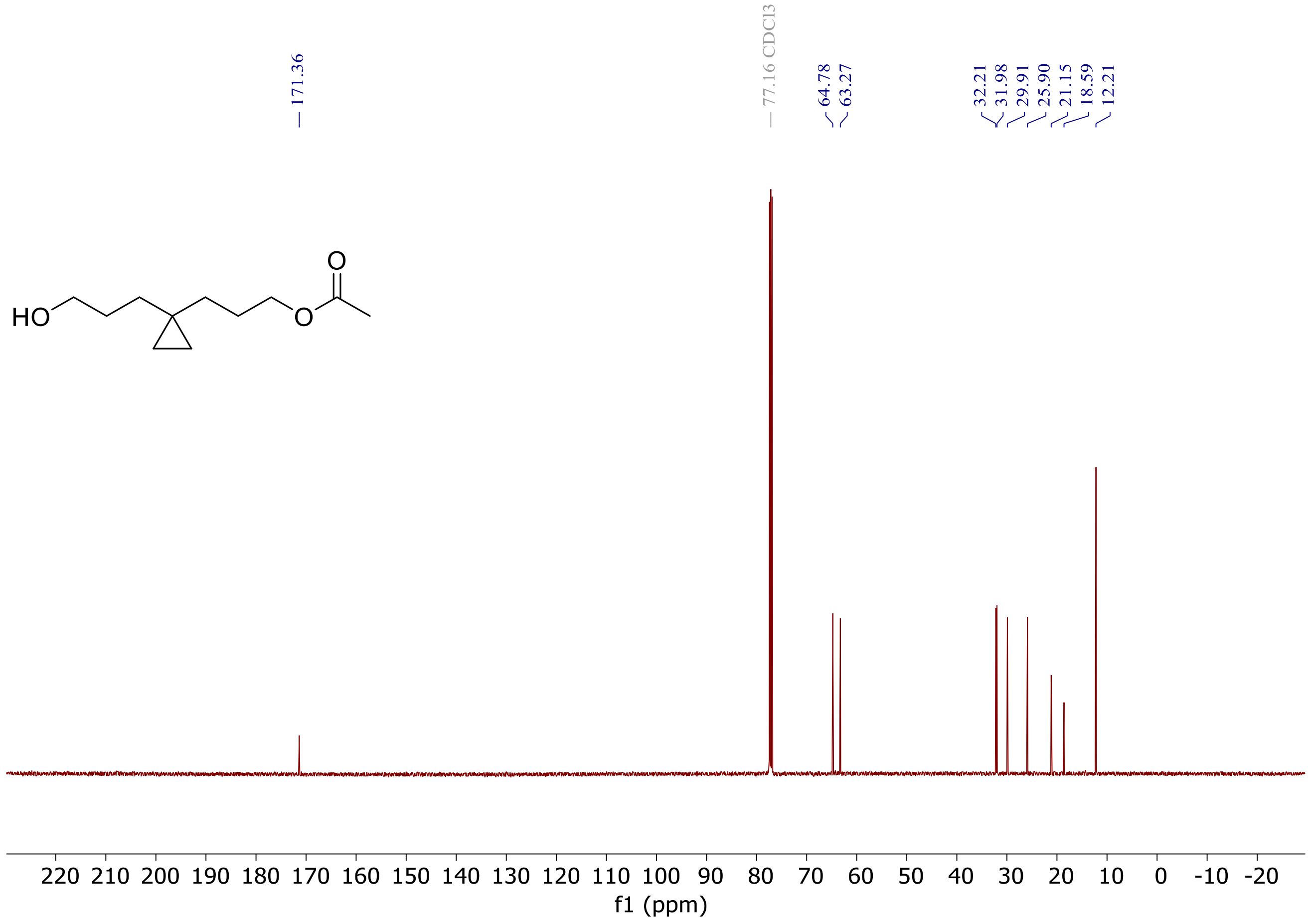
171.36
77.16 CDCl3
64.78
63.27
32.21
31.98
29.91
25.90
21.15
18.59
12.21
HO
O
O
220 210 200 190 180 170 160 150 140 130 120 110 100 90 80 70 60 50 40 30 20 10 0 -10 -20
f1 (ppm)

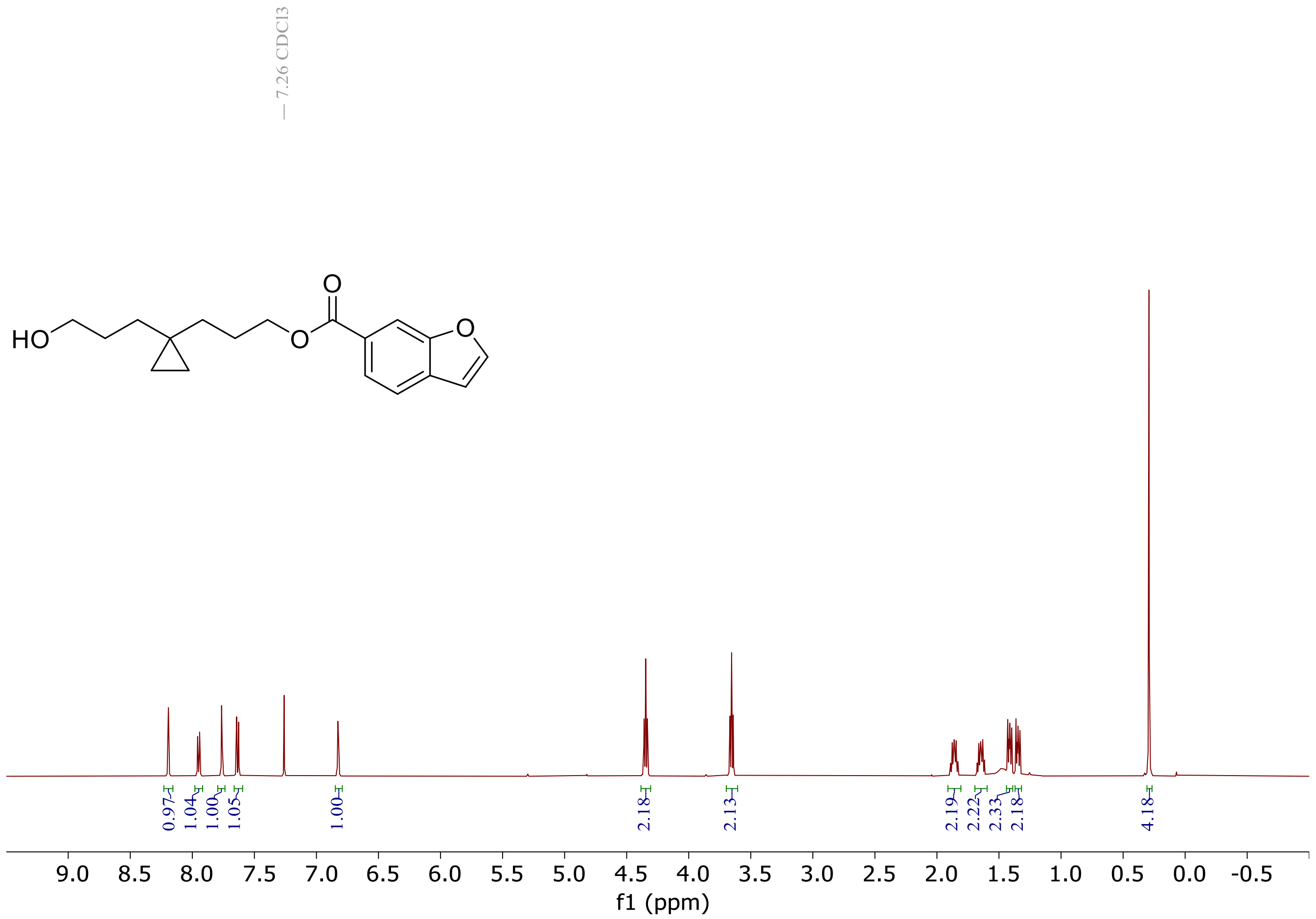
7.26 CDCl3
HO
O
O
O
0.97
1.04
1.00
1.05
1.00
2.18
2.13
2.19
2.22
2.33
2.18
4.18
9.0 8.5 8.0 7.5 7.0 6.5 6.0 5.5 5.0 4.5 4.0 3.5 3.0 2.5 2.0 1.5 1.0 0.5 0.0 -0.5
f1 (ppm)

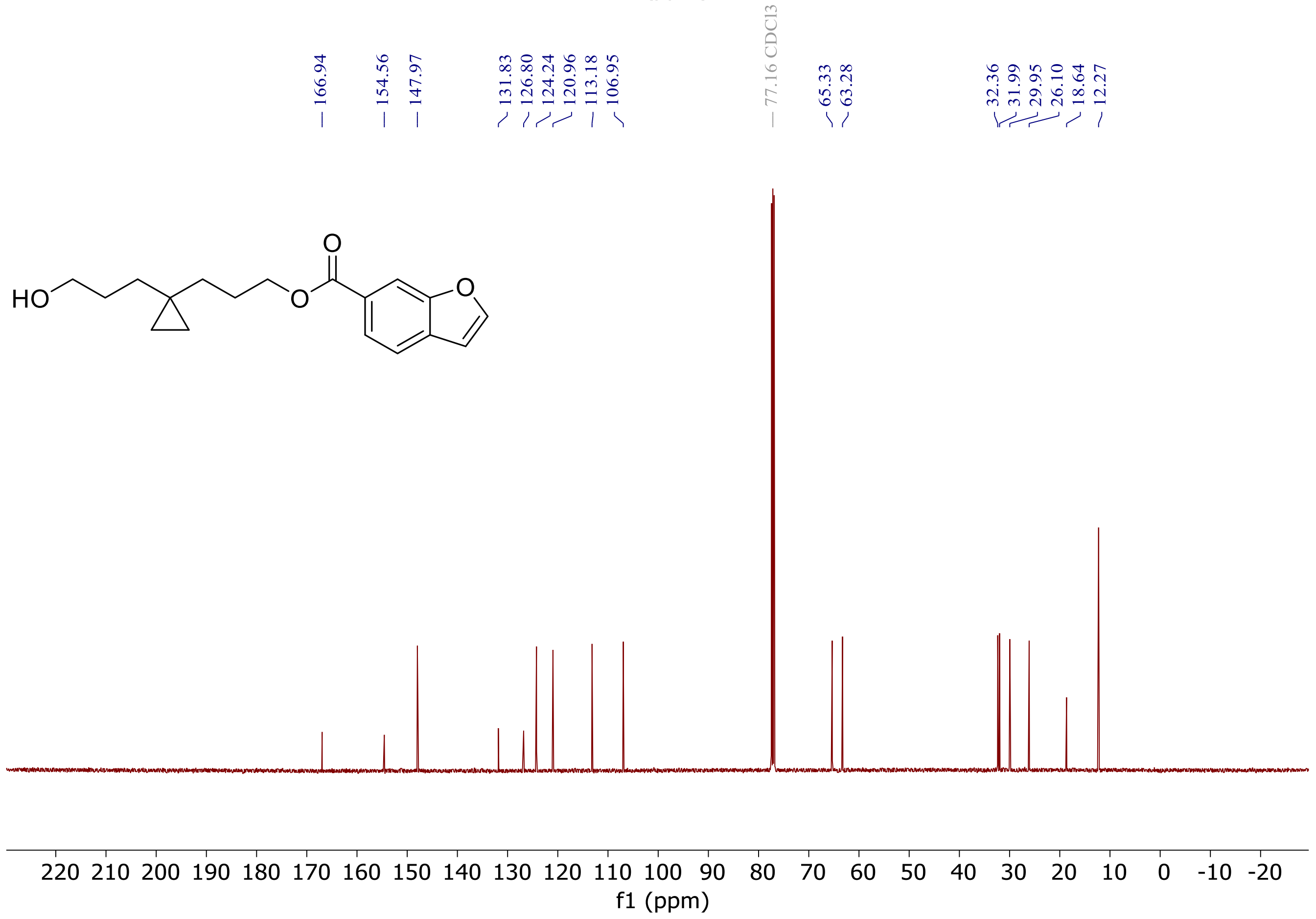
166.94
154.56
147.97
131.83
126.80
124.24
120.96
113.18
106.95
77.16 CDCl3
65.33
63.28
32.36
31.99
29.95
26.10
18.64
12.27
HO
O
O
O
220 210 200 190 180 170 160 150 140 130 120 110 100 90 80 70 60 50 40 30 20 10 0 -10 -20
f1 (ppm)

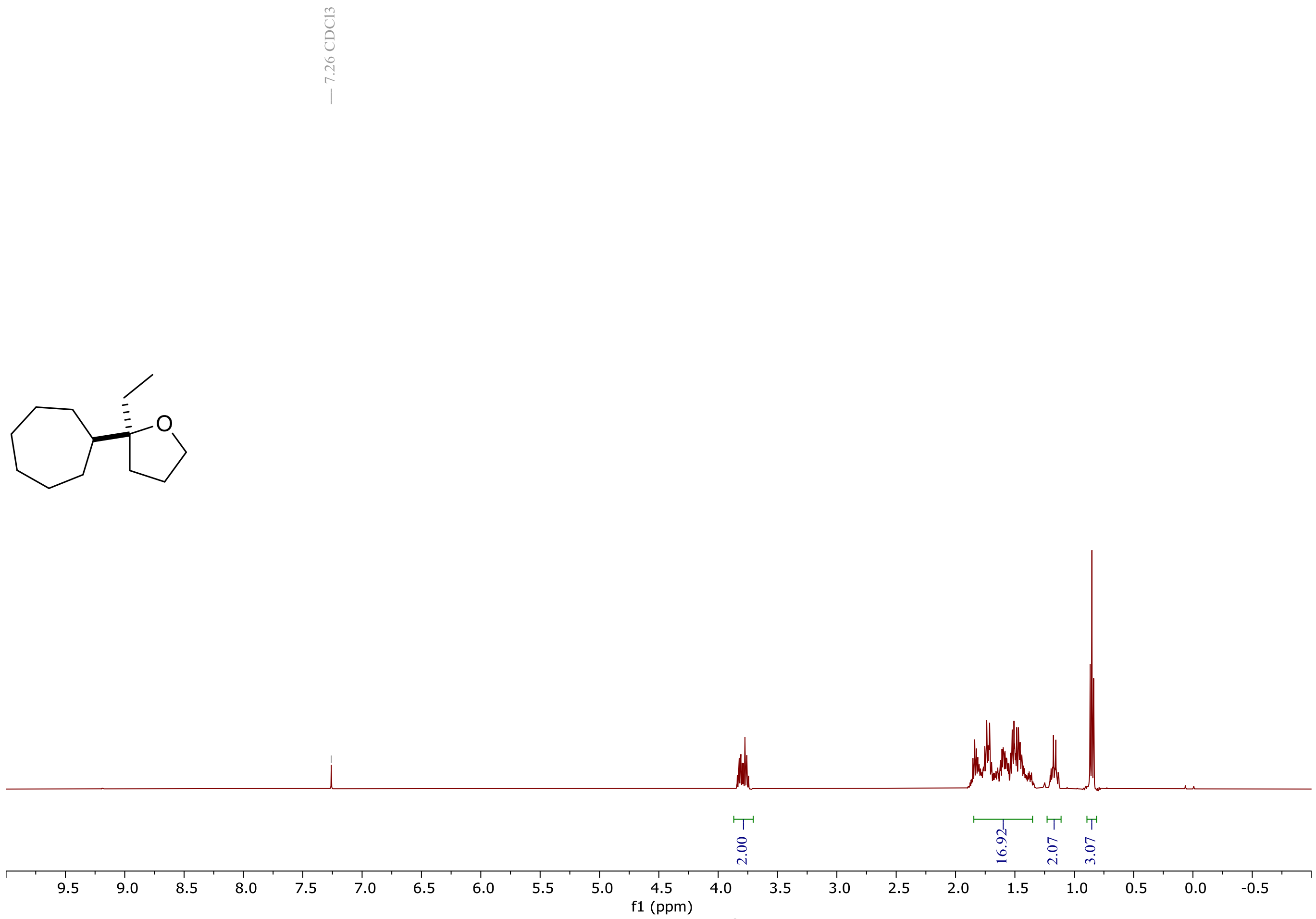

7.26 CDCl3
O
2.00
16.92
2.07
3.07
9.5 9.0 8.5 8.0 7.5 7.0 6.5 6.0 5.5 5.0 4.5 4.0 3.5 3.0 2.5 2.0 1.5 1.0 0.5 0.0 -0.5
f1 (ppm)

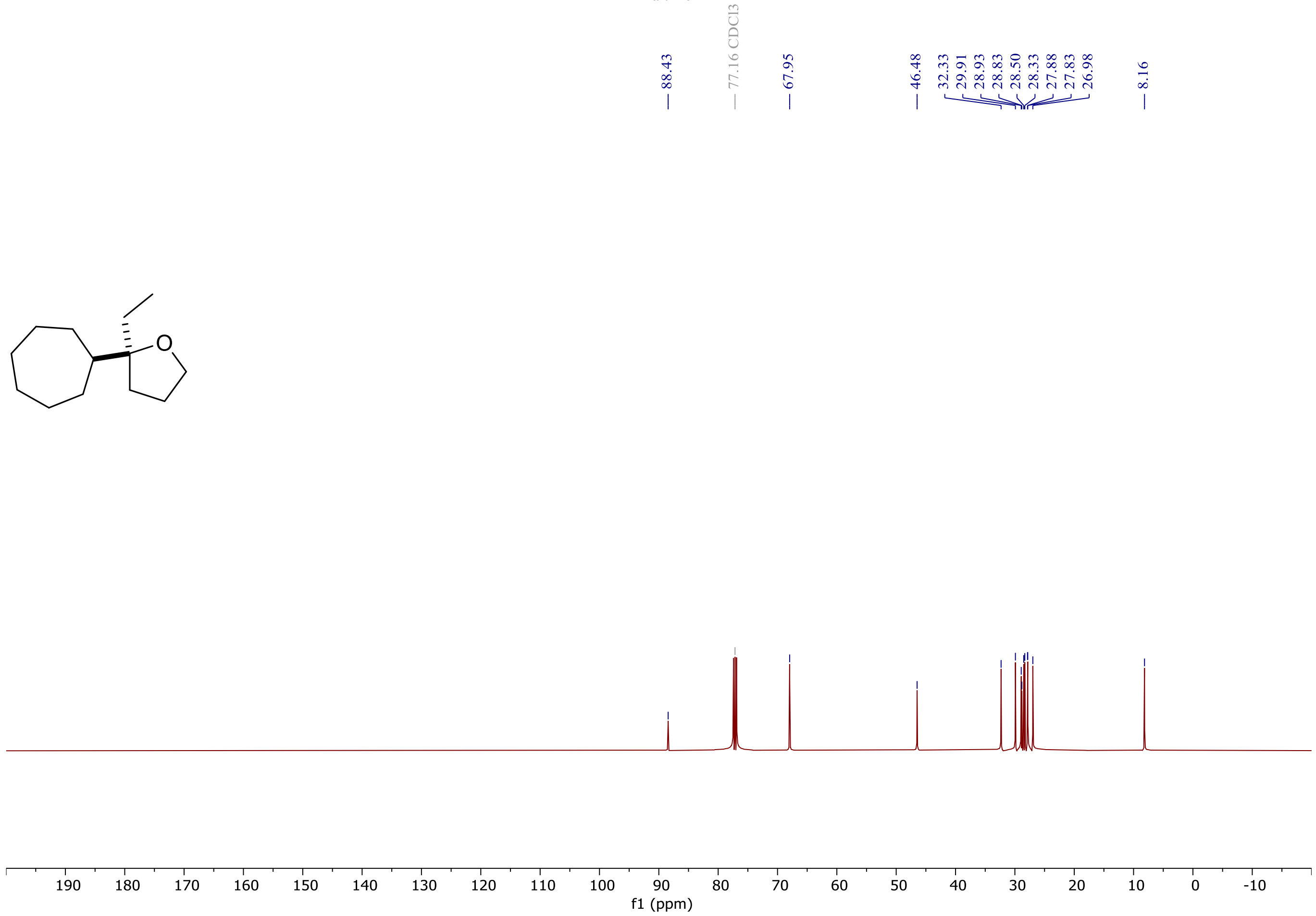

88.43
77.16 CDCl3
67.95
46.48
32.33
29.91
28.93
28.83
28.50
28.33
27.88
27.83
26.98
8.16
O
190 180 170 160 150 140 130 120 110 100 90 80 70 60 50 40 30 20 10 0 -10
f1 (ppm)

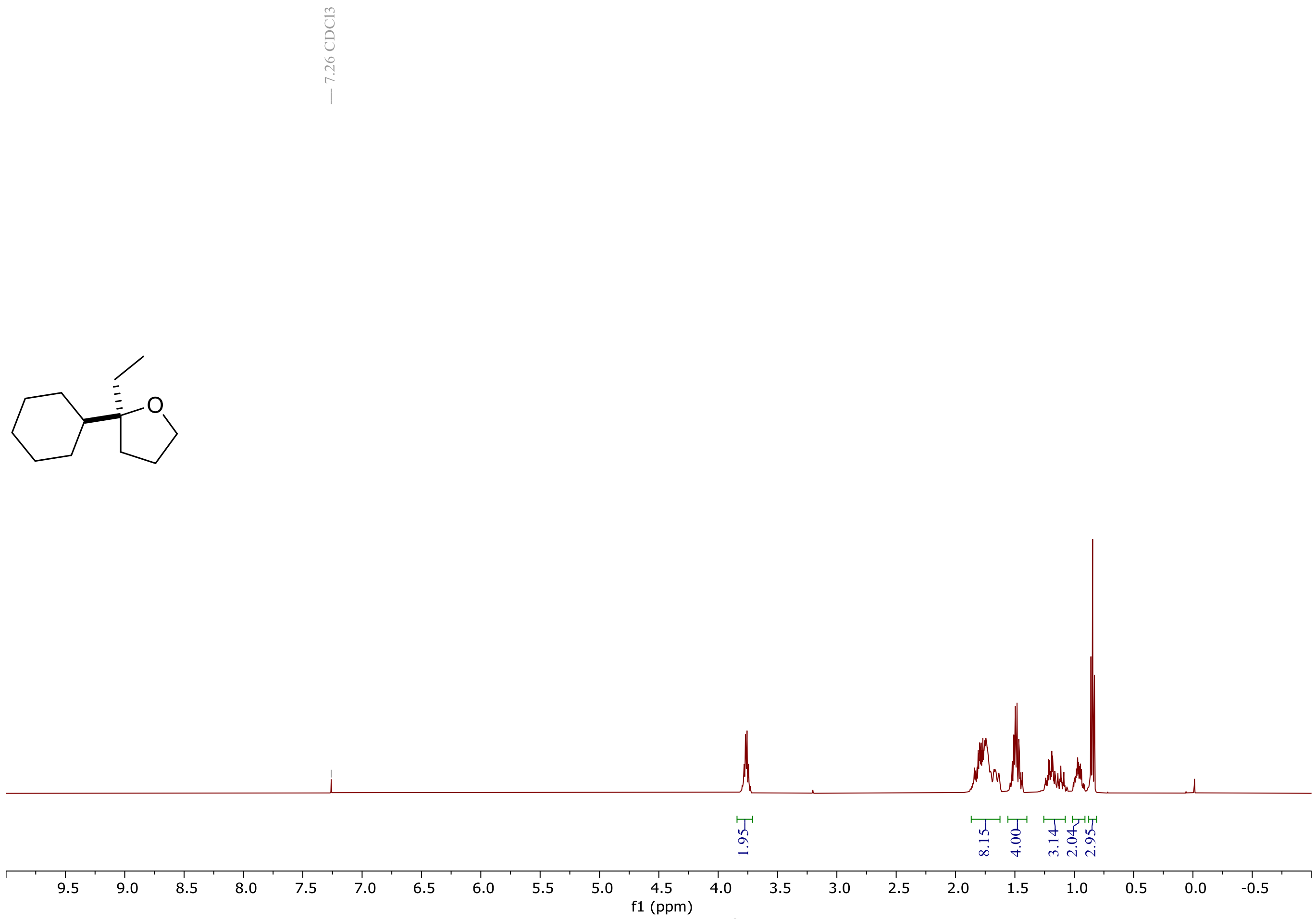
7.26 CDCl3
1.95
8.15
4.00
3.14
2.04
2.95
9.5 9.0 8.5 8.0 7.5 7.0 6.5 6.0 5.5 5.0 4.5 4.0 3.5 3.0 2.5 2.0 1.5 1.0 0.5 0.0 -0.5
f1 (ppm)

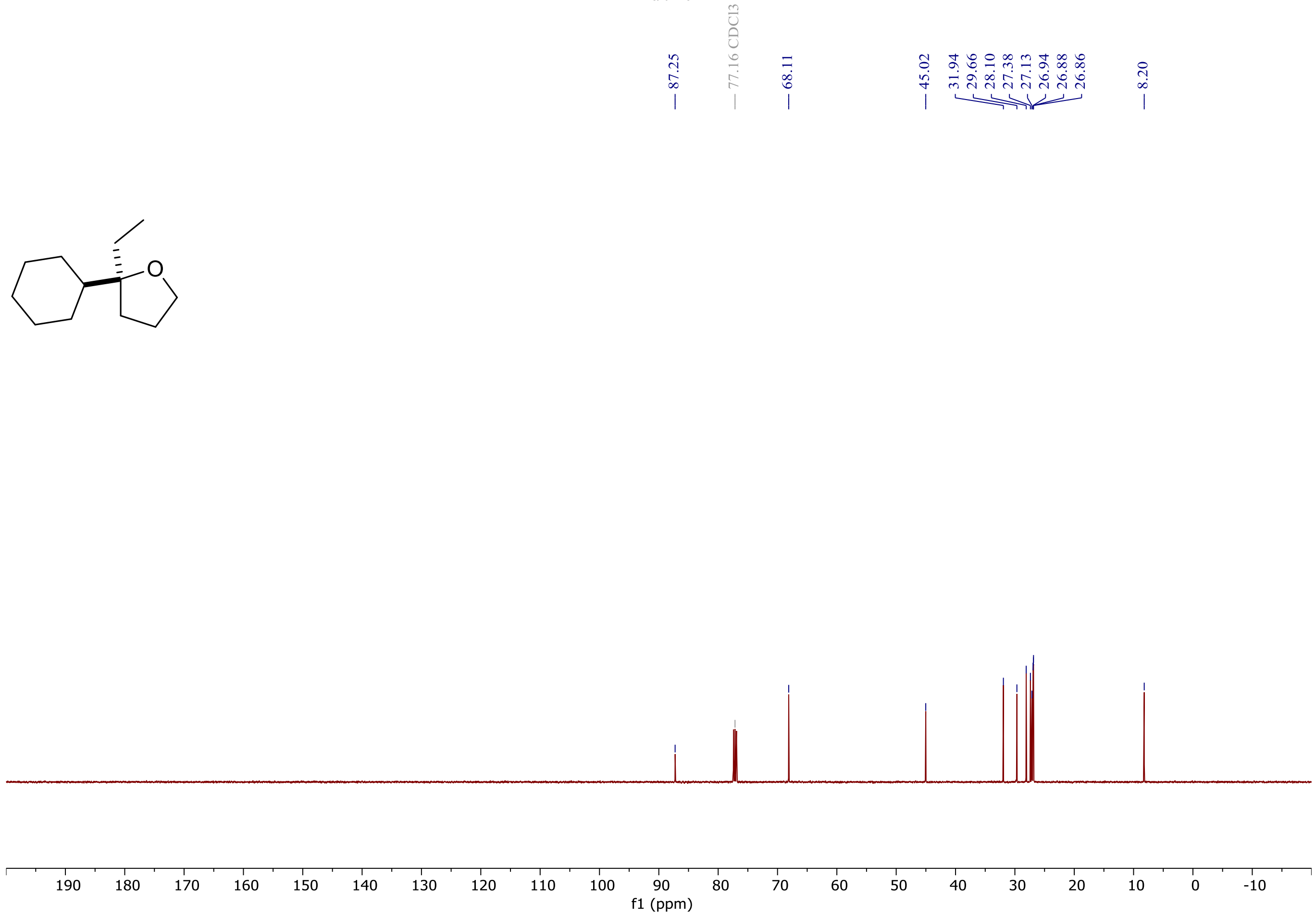
87.25
77.16 CDCl3
68.11
45.02
31.94
29.66
28.10
27.38
27.13
26.94
26.88
26.86
8.20
190 180 170 160 150 140 130 120 110 100 90 80 70 60 50 40 30 20 10 0 -10
f1 (ppm)

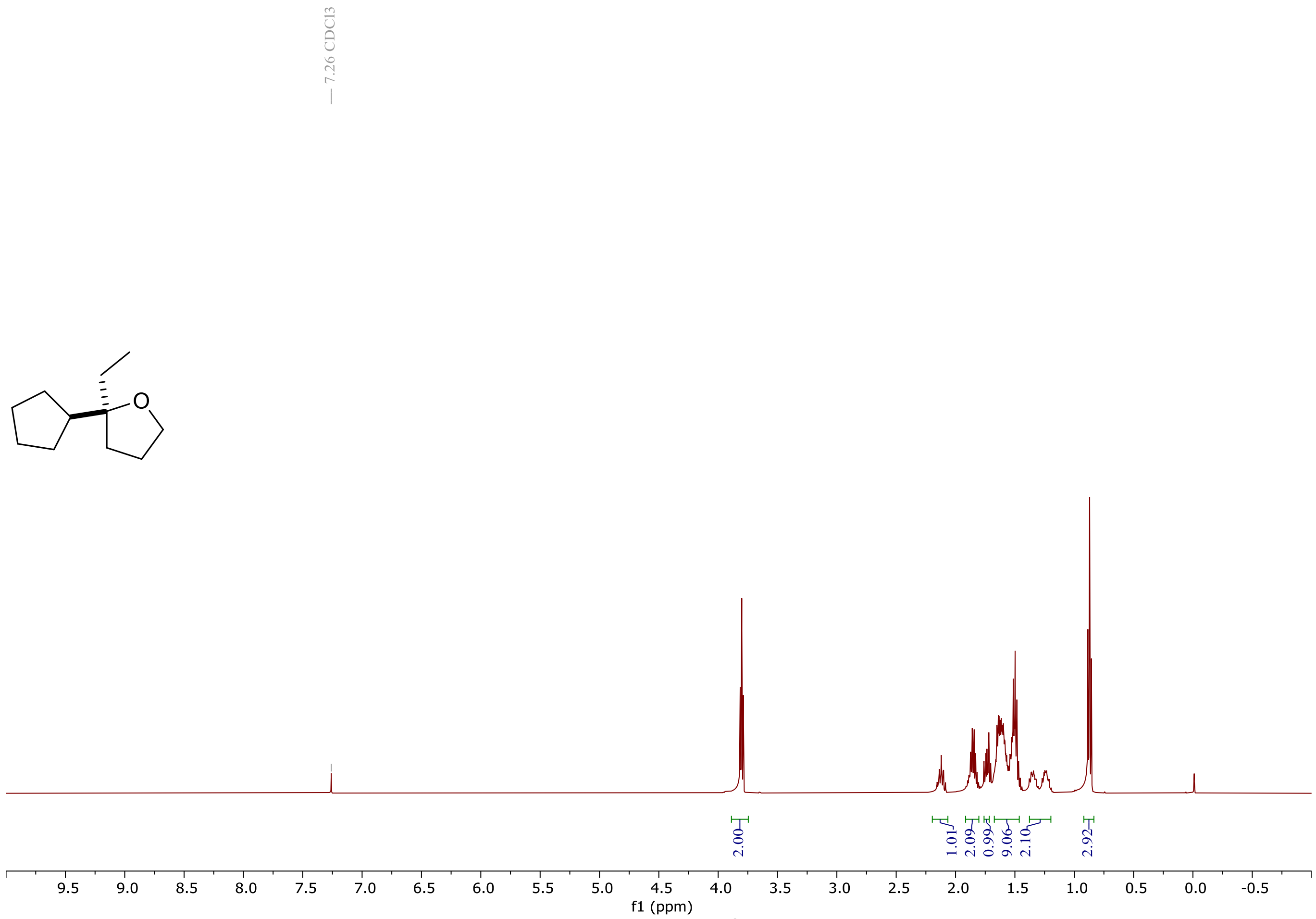

7.26 CDCl3
O
2.00
1.01
2.09
0.99
9.06
2.10
2.92
9.5 9.0 8.5 8.0 7.5 7.0 6.5 6.0 5.5 5.0 4.5 4.0 3.5 3.0 2.5 2.0 1.5 1.0 0.5 0.0 -0.5
f1 (ppm)

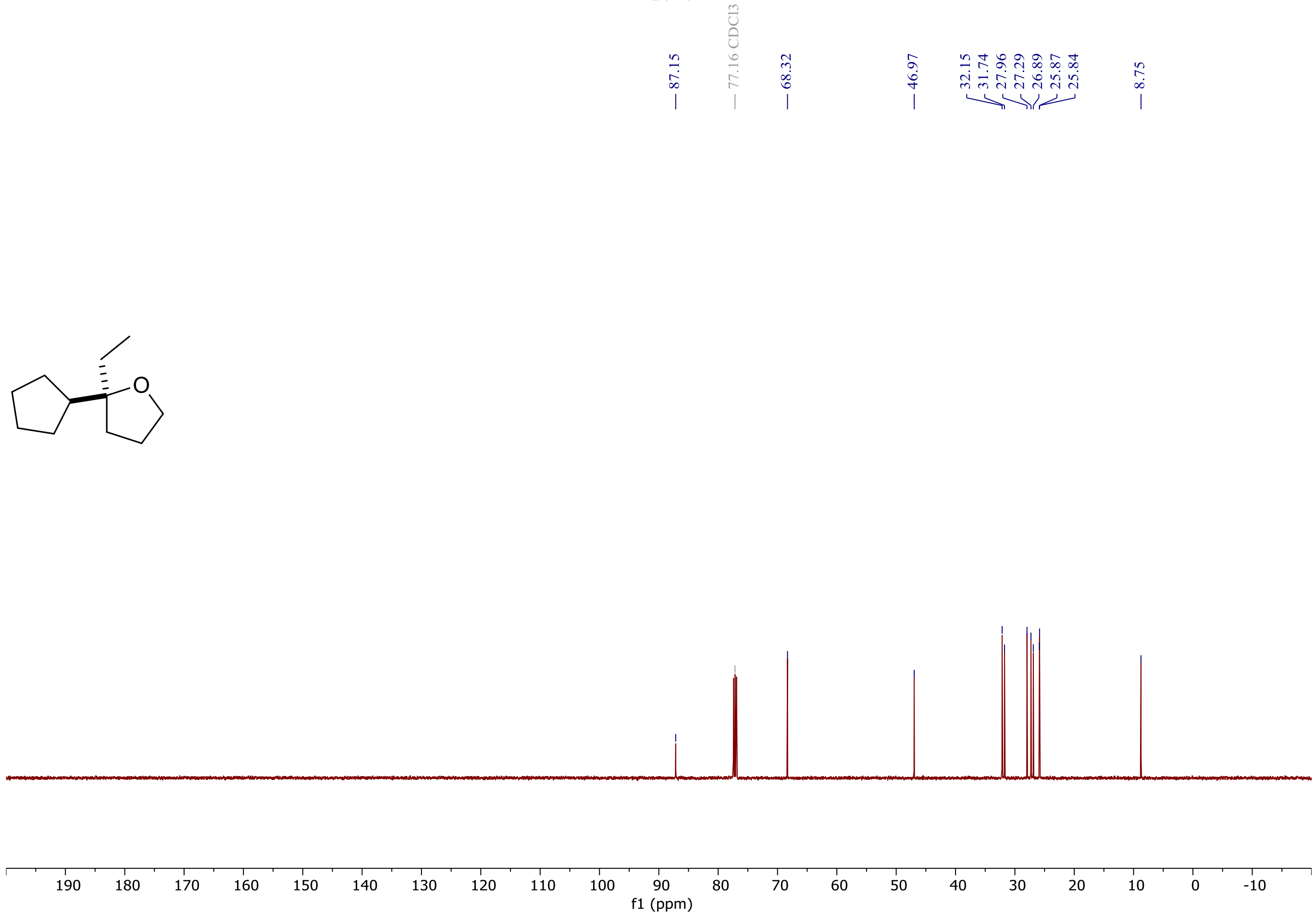

87.15
77.16 CDCl3
68.32
46.97
32.15
31.74
27.96
27.29
26.89
25.87
25.84
8.75
O
190 180 170 160 150 140 130 120 110 100 90 80 70 60 50 40 30 20 10 0 -10
f1 (ppm)

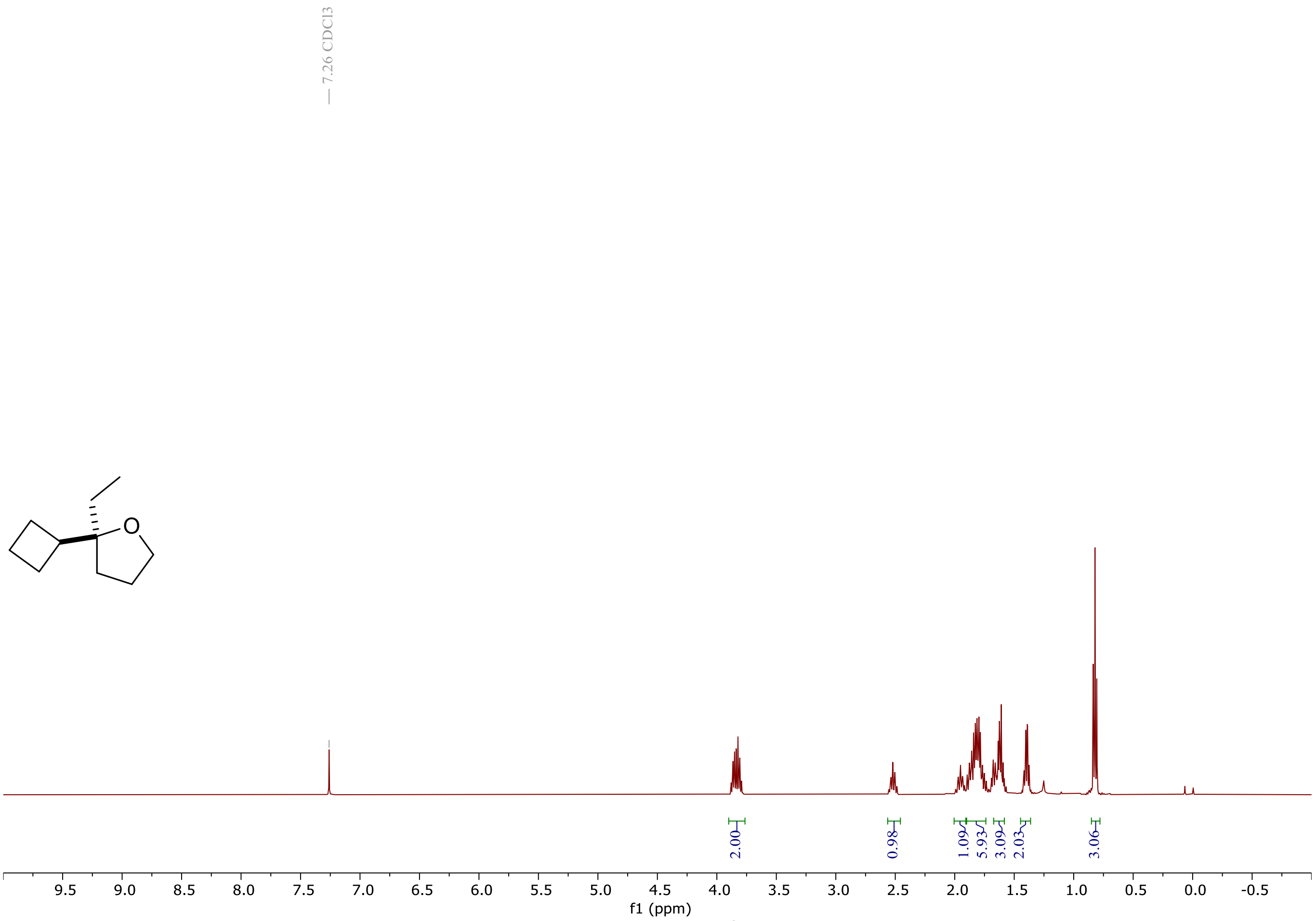

7.26 CDCl3
O
2.00
0.98
1.09
5.93
3.09
2.03
3.06
9.5 9.0 8.5 8.0 7.5 7.0 6.5 6.0 5.5 5.0 4.5 4.0 3.5 3.0 2.5 2.0 1.5 1.0 0.5 0.0 -0.5
f1 (ppm)

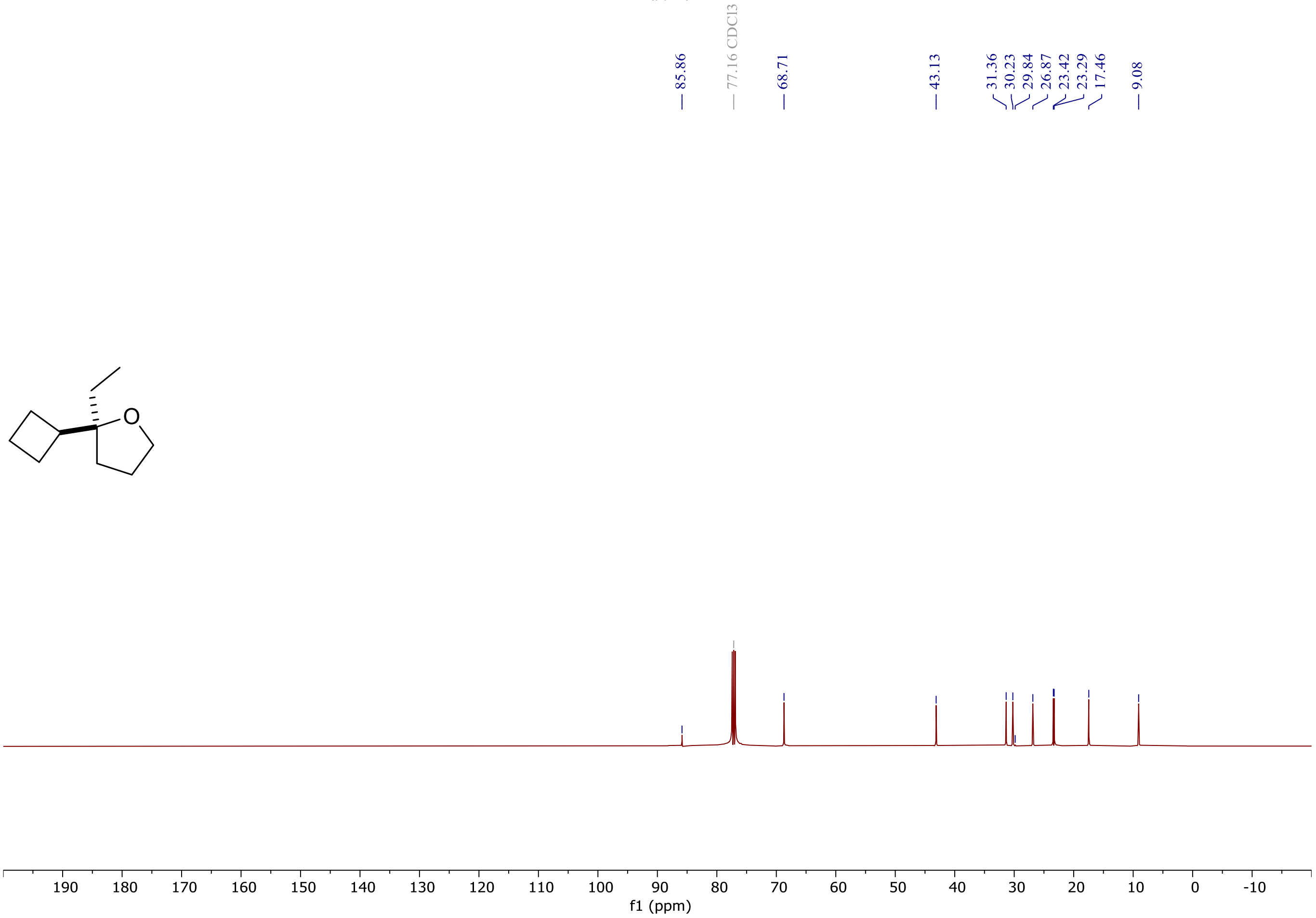

85.86
77.16 CDCl3
68.71
43.13
31.36
30.23
29.84
26.87
23.42
23.29
17.46
9.08
O
190 180 170 160 150 140 130 120 110 100 90 80 70 60 50 40 30 20 10 0 -10
f1 (ppm)

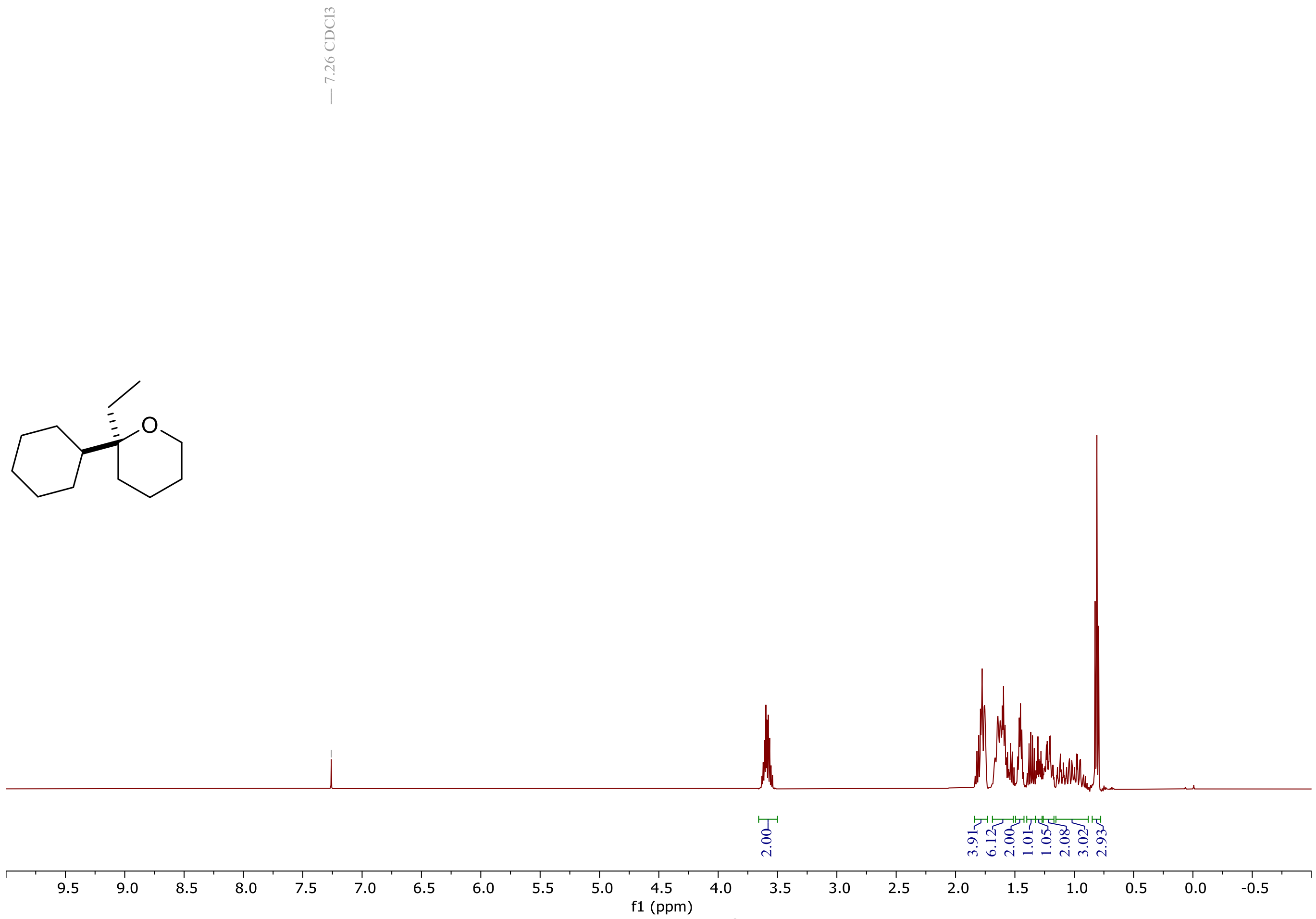
7.26 CDCl3
O
2.00
3.91
6.12
2.00
1.01
1.05
2.08
3.02
2.93
9.5 9.0 8.5 8.0 7.5 7.0 6.5 6.0 5.5 5.0 4.5 4.0 3.5 3.0 2.5 2.0 1.5 1.0 0.5 0.0 -0.5
f1 (ppm)

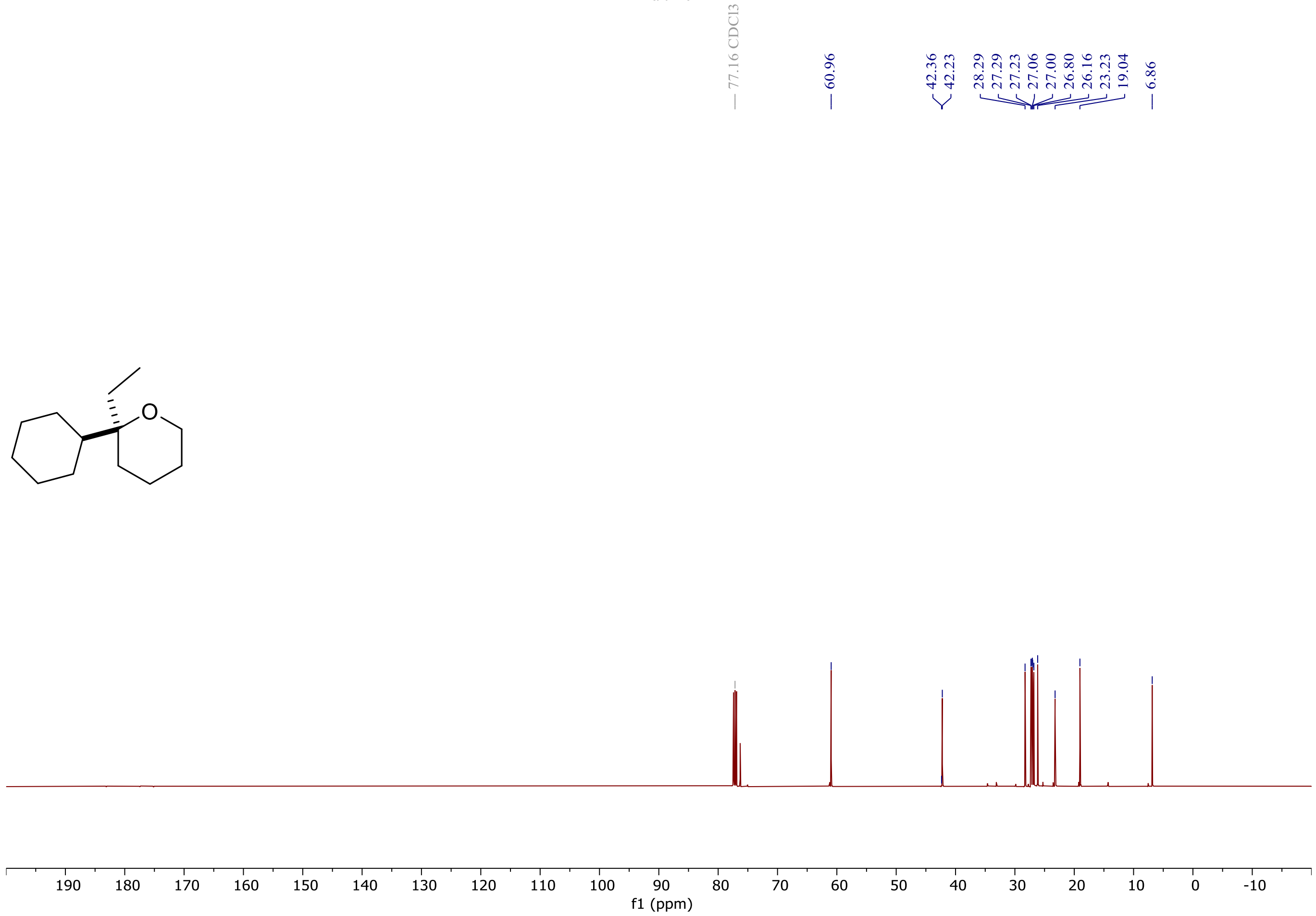
77.16 CDCl3
60.96
42.36
42.23
28.29
27.29
27.23
27.06
27.00
26.80
26.16
23.23
19.04
6.86
O
190 180 170 160 150 140 130 120 110 100 90 80 70 60 50 40 30 20 10 0 -10
f1 (ppm)

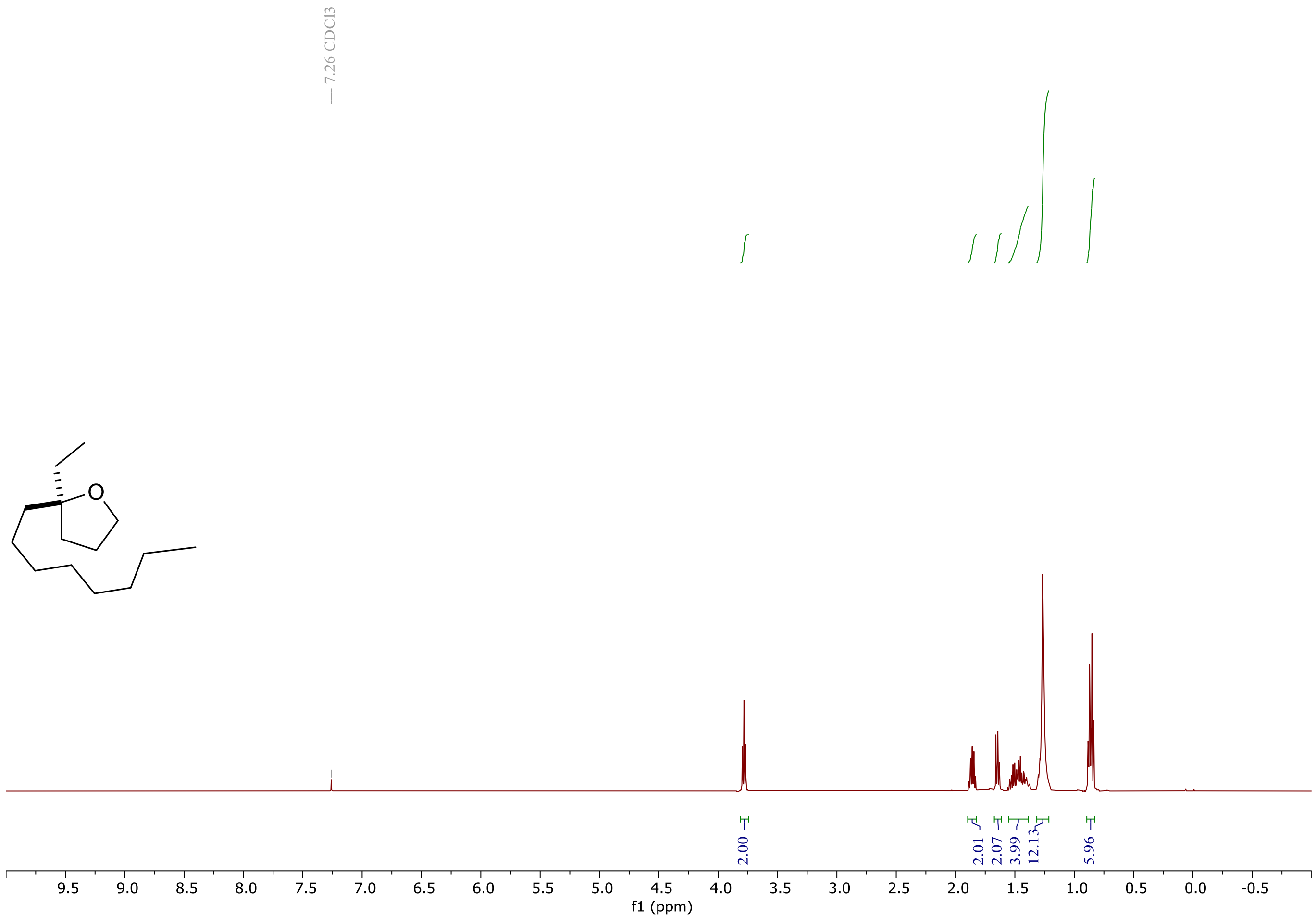
7.26 CDCl3
O
2.00
2.01
2.07
3.99
12.13
5.96
9.5 9.0 8.5 8.0 7.5 7.0 6.5 6.0 5.5 5.0 4.5 4.0 3.5 3.0 2.5 2.0 1.5 1.0 0.5 0.0 -0.5
f1 (ppm)

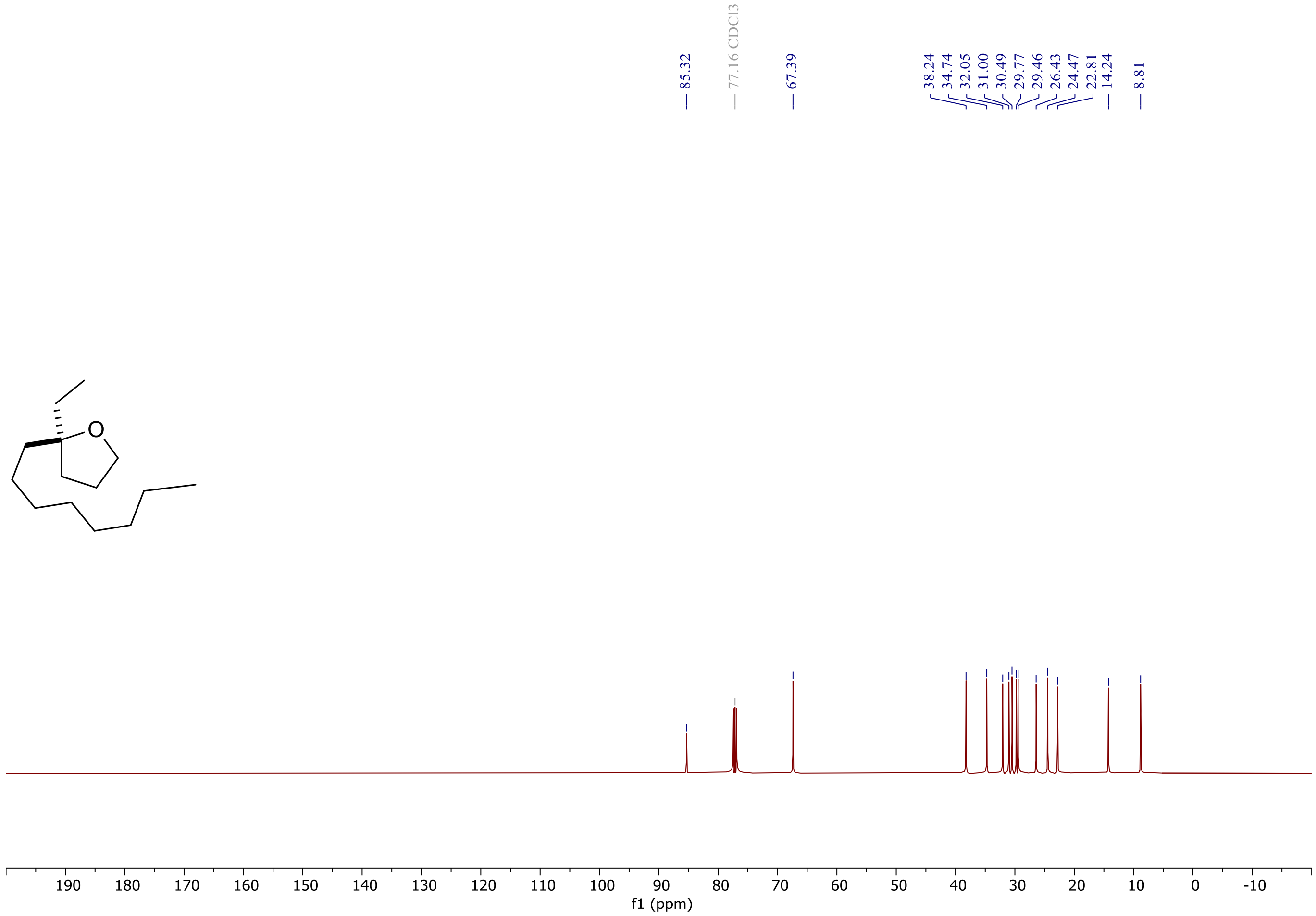
85.32
77.16 CDCl3
67.39
38.24
34.74
32.05
31.00
30.49
29.77
29.46
26.43
24.47
22.81
14.24
8.81
O
190 180 170 160 150 140 130 120 110 100 90 80 70 60 50 40 30 20 10 0 -10
f1 (ppm)

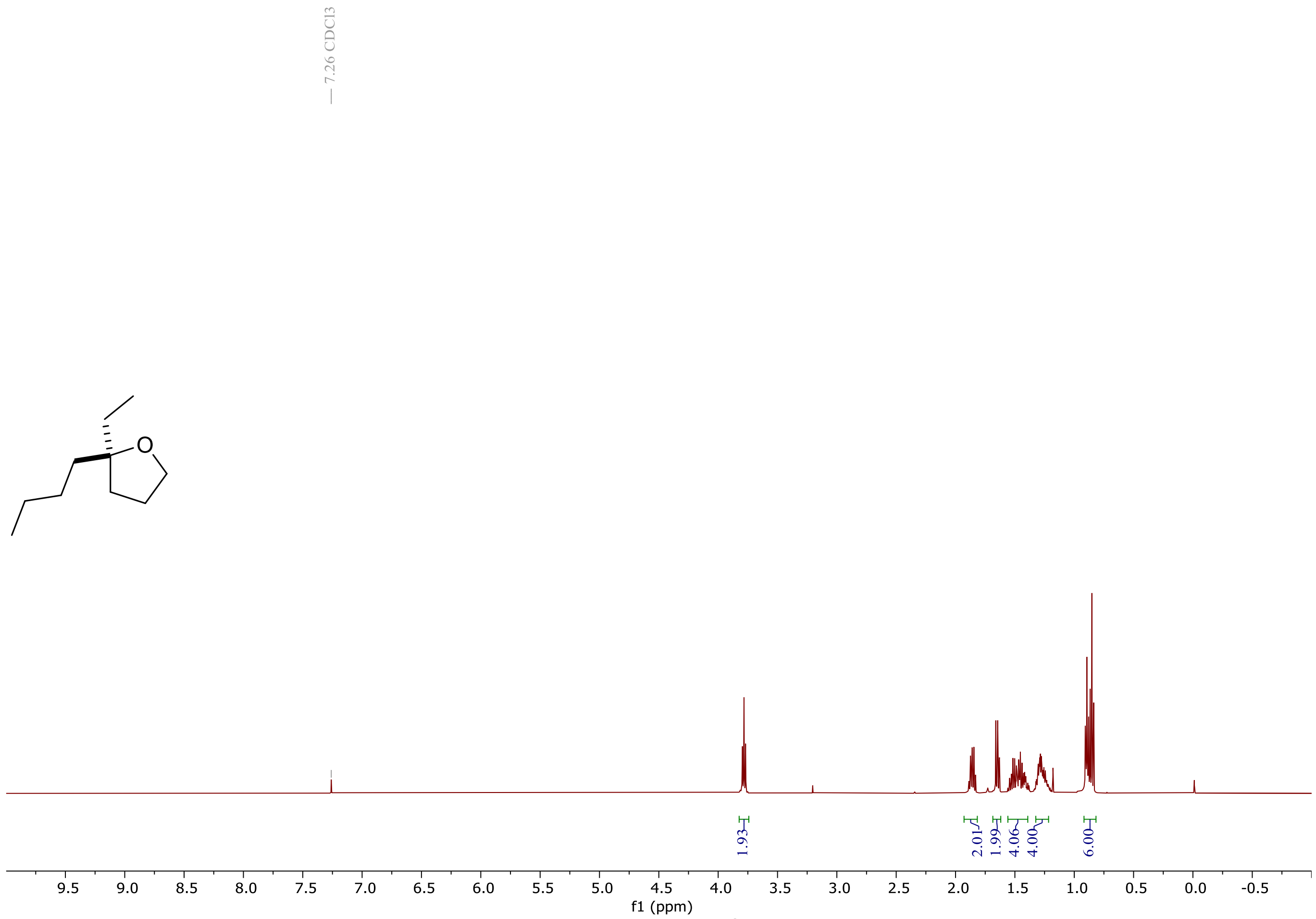

7.26 CDCl3
O
1.93
2.01
1.99
4.06
4.00
6.00
9.5
9.0
8.5
8.0
7.5
7.0
6.5
6.0
5.5
5.0
4.5
4.0
3.5
3.0
2.5
2.0
1.5
1.0
0.5
0.0
-0.5
f1 (ppm)

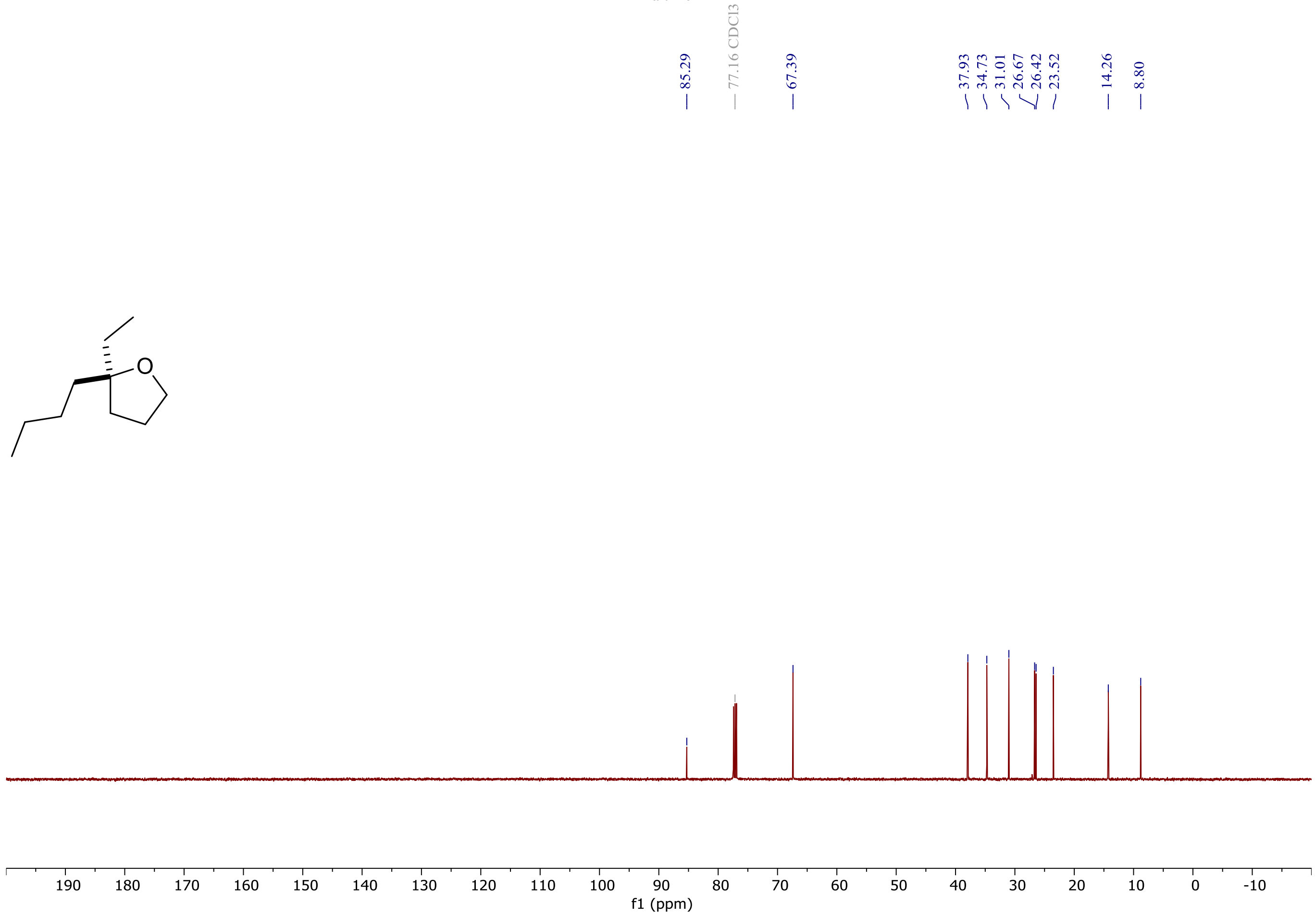

85.29
77.16 CDCl3
67.39
37.93
34.73
31.01
26.67
26.42
23.52
14.26
8.80
O
190
180
170
160
150
140
130
120
110
100
90
80
70
60
50
40
30
20
10
0
-10
f1 (ppm)

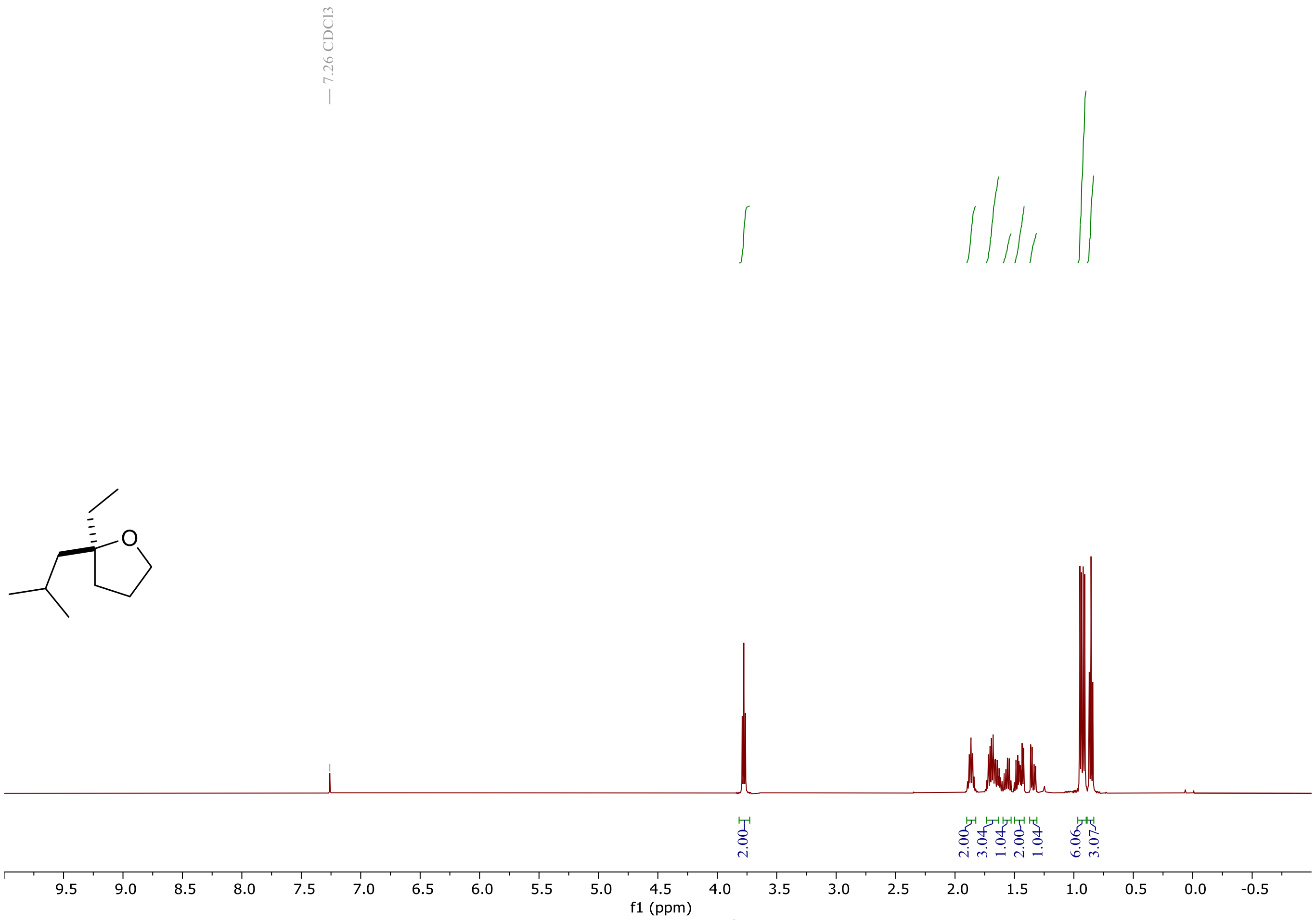
7.26 CDCl3
2.00
2.00
3.04
1.04
2.00
1.04
6.06
3.07
f1 (ppm)

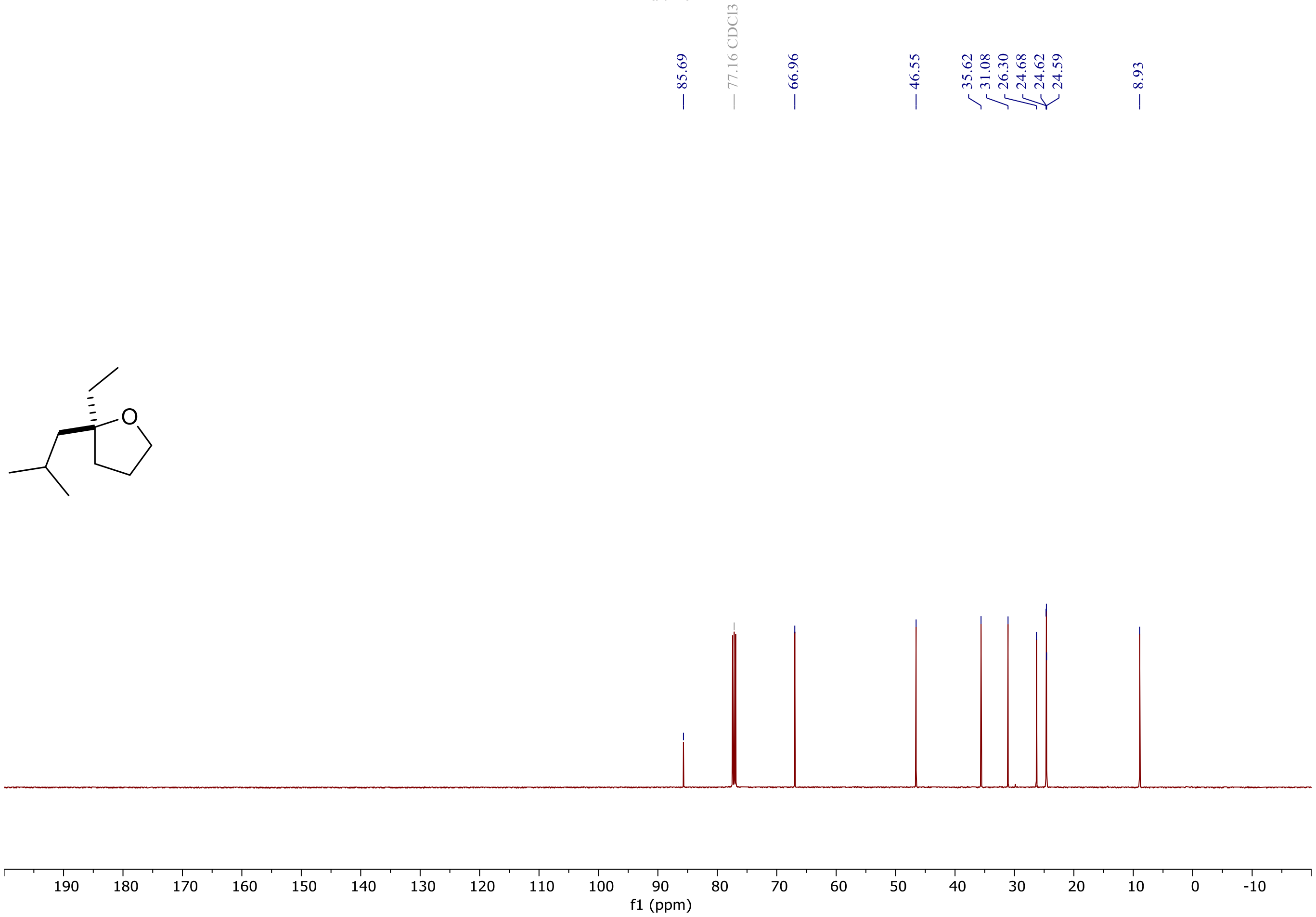
85.69
77.16 CDCl3
66.96
46.55
35.62
31.08
26.30
24.68
24.62
24.59
8.93
f1 (ppm)

Based on the volatility of the substrates **3i**, it is impossible to completely eliminate the DCM and MTBE solvent from the result.

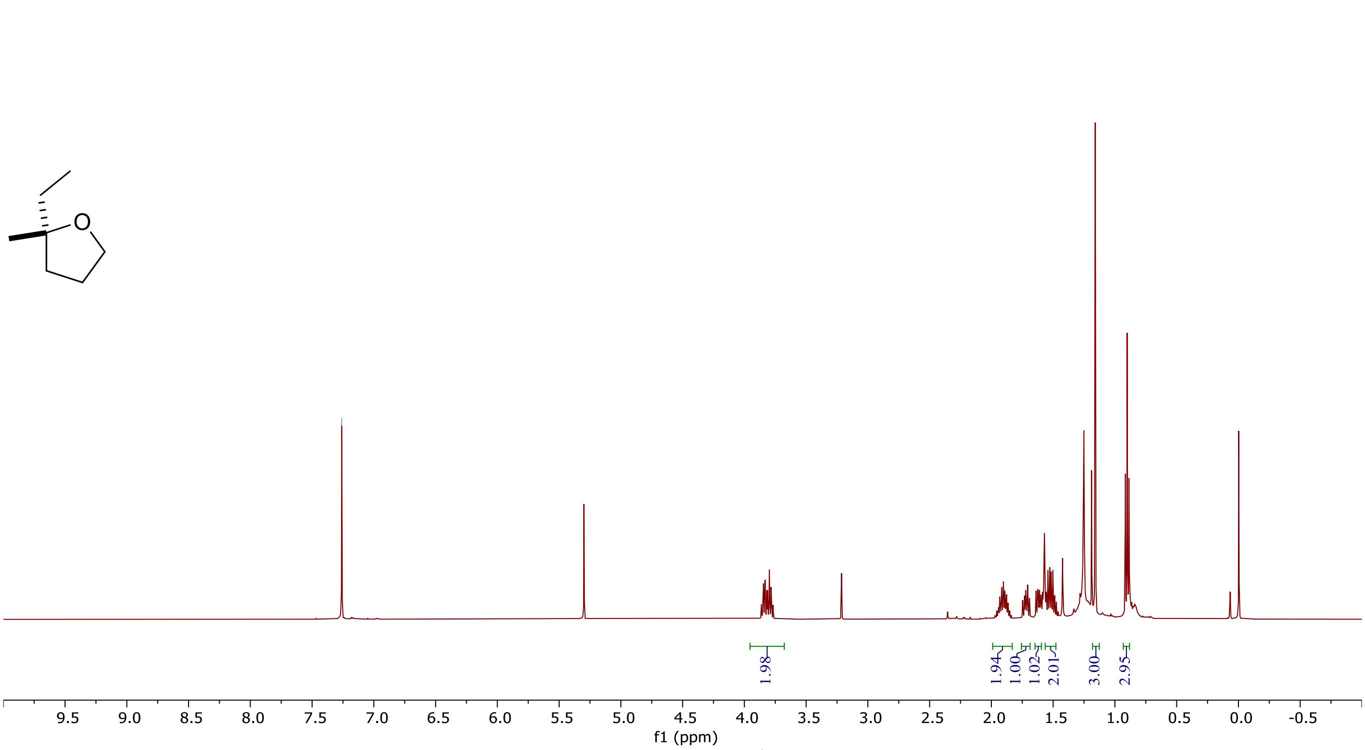

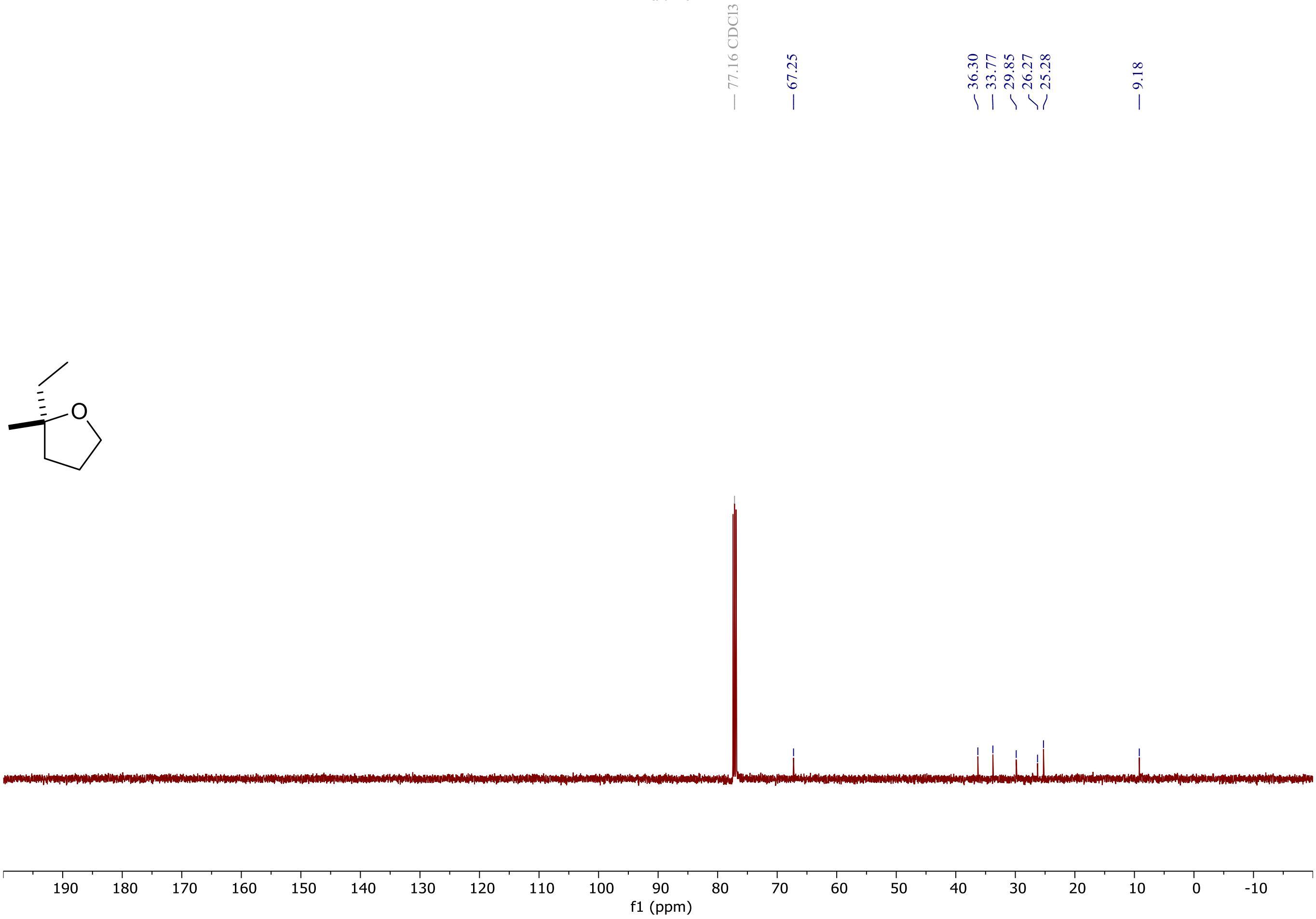

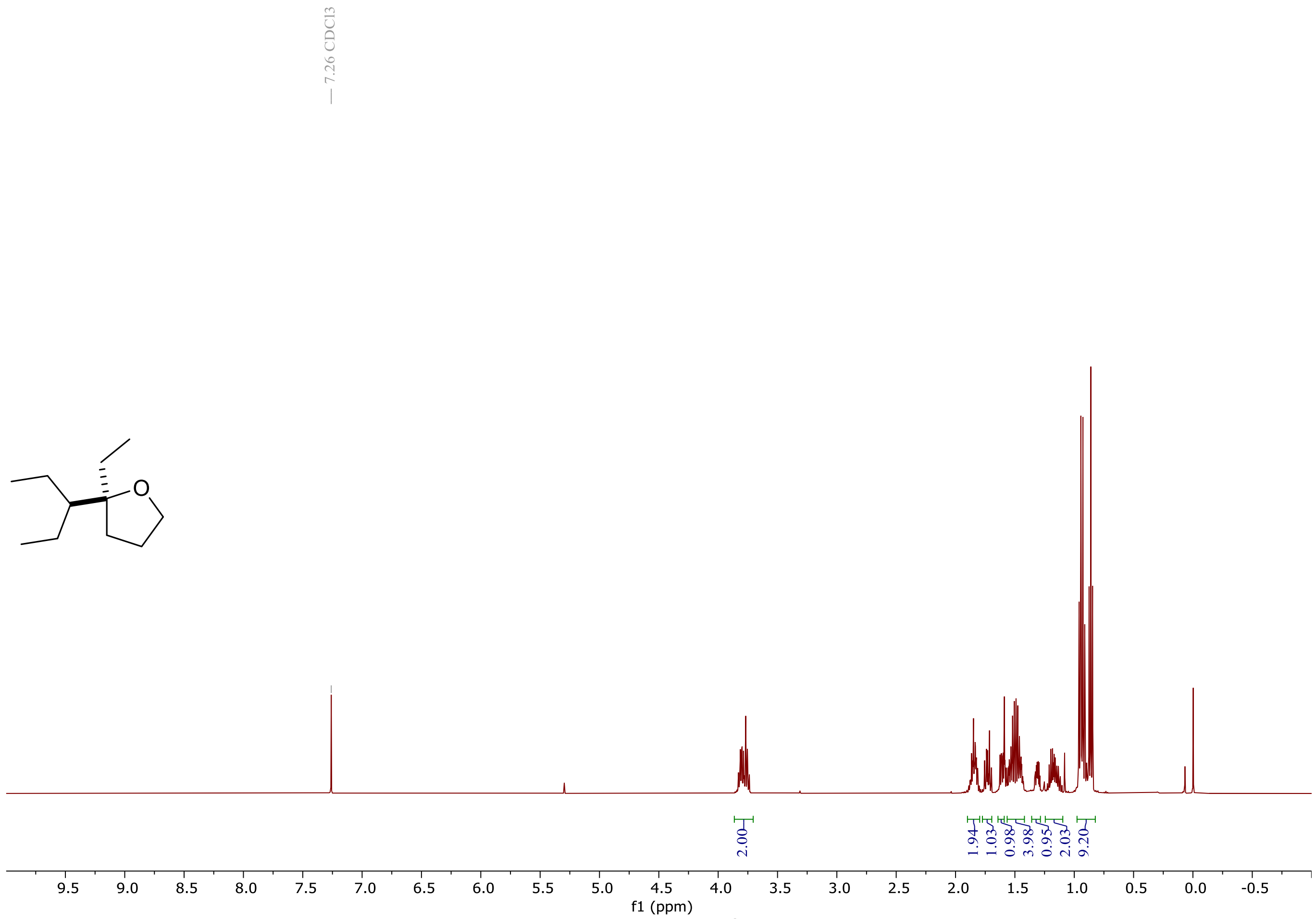
7.26 CDCl3
2.00
1.94
1.03
0.98
3.98
0.95
2.03
9.20
9.5 9.0 8.5 8.0 7.5 7.0 6.5 6.0 5.5 5.0 4.5 4.0 3.5 3.0 2.5 2.0 1.5 1.0 0.5 0.0 -0.5
f1 (ppm)
O

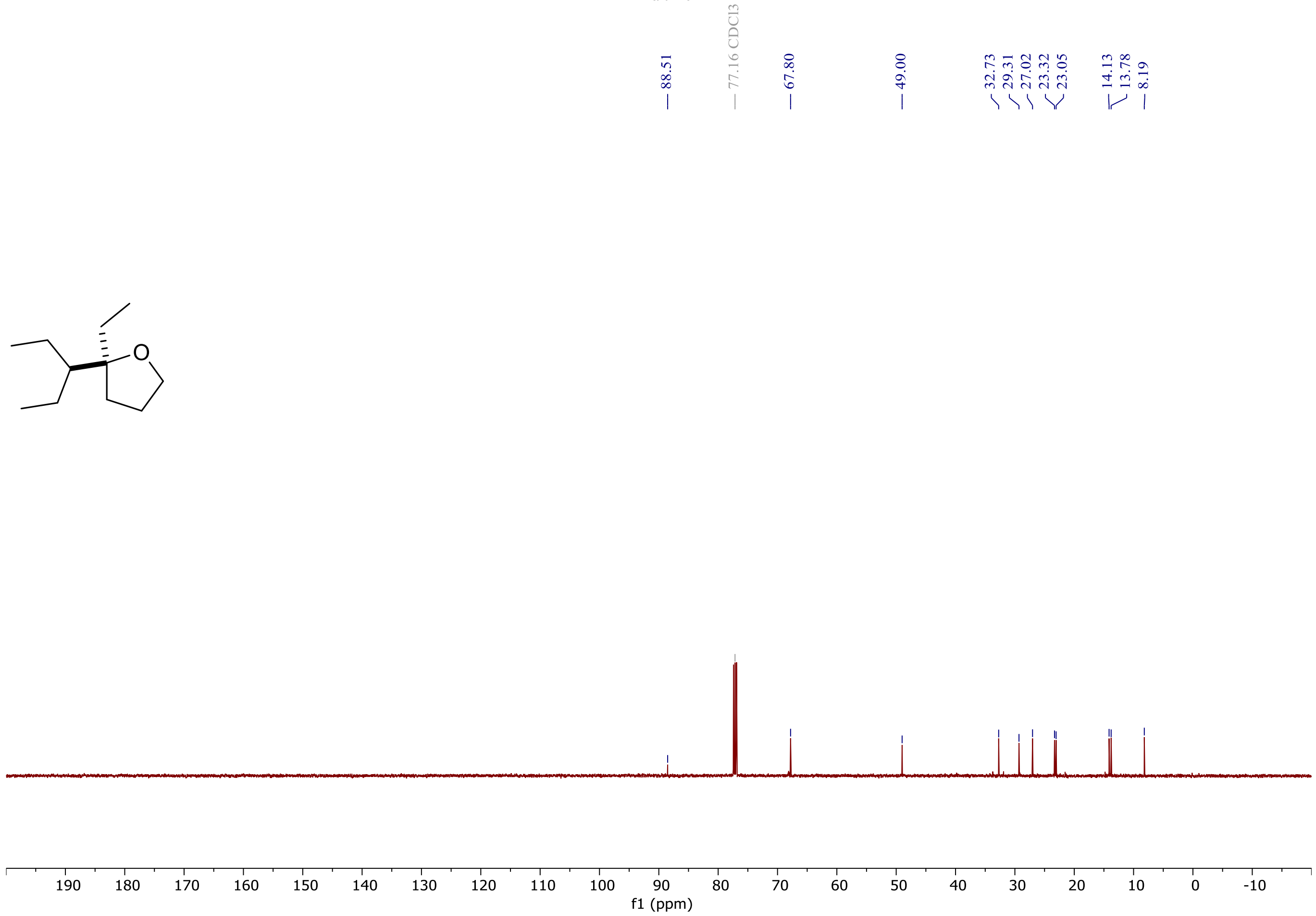
88.51
77.16 CDCl3
67.80
49.00
32.73
29.31
27.02
23.32
23.05
14.13
13.78
8.19
190 180 170 160 150 140 130 120 110 100 90 80 70 60 50 40 30 20 10 0 -10
f1 (ppm)
O

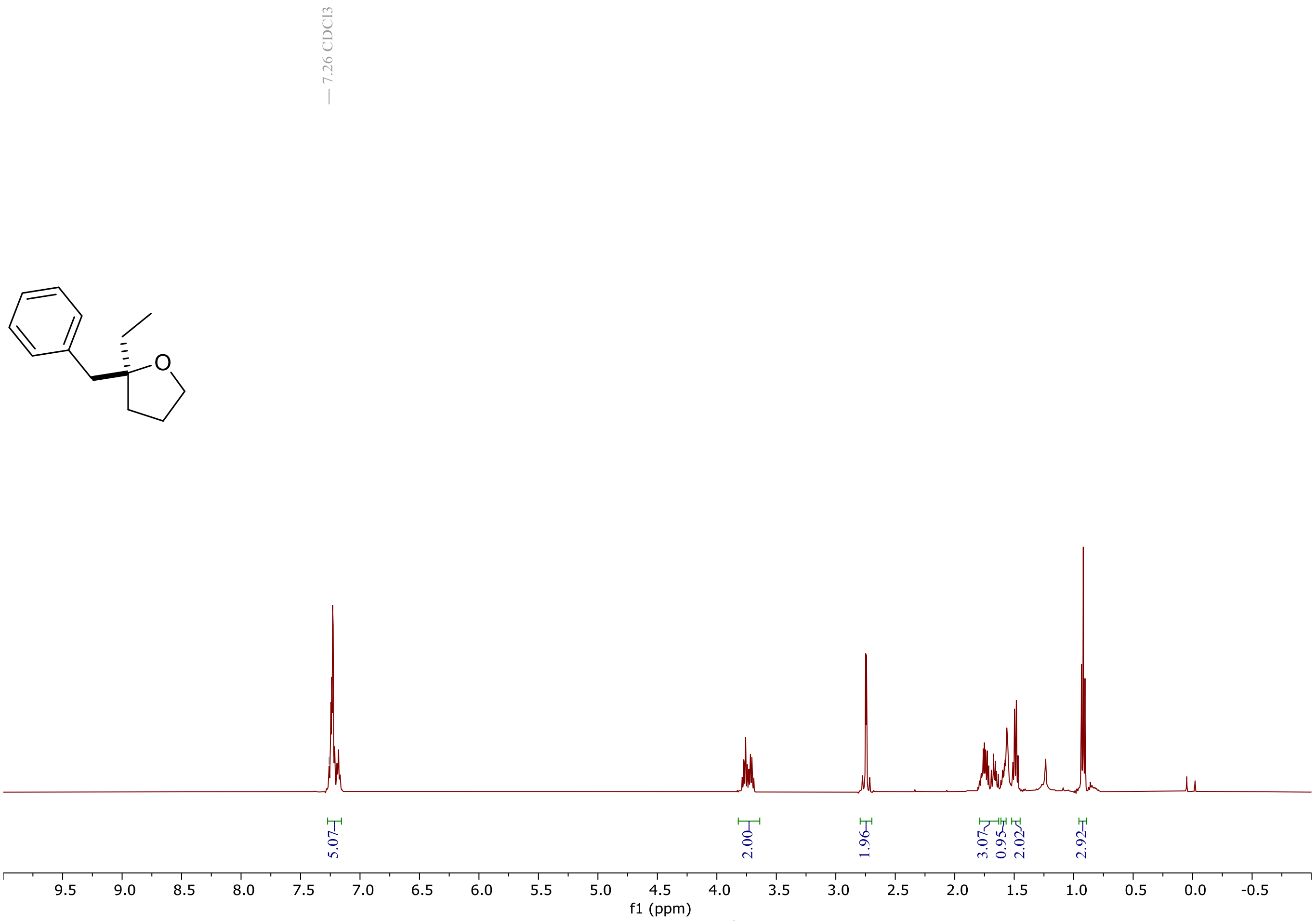
7.26 CDCl3
O
5.07
2.00
1.96
3.07
0.95
2.02
2.92
9.5 9.0 8.5 8.0 7.5 7.0 6.5 6.0 5.5 5.0 4.5 4.0 3.5 3.0 2.5 2.0 1.5 1.0 0.5 0.0 -0.5
f1 (ppm)

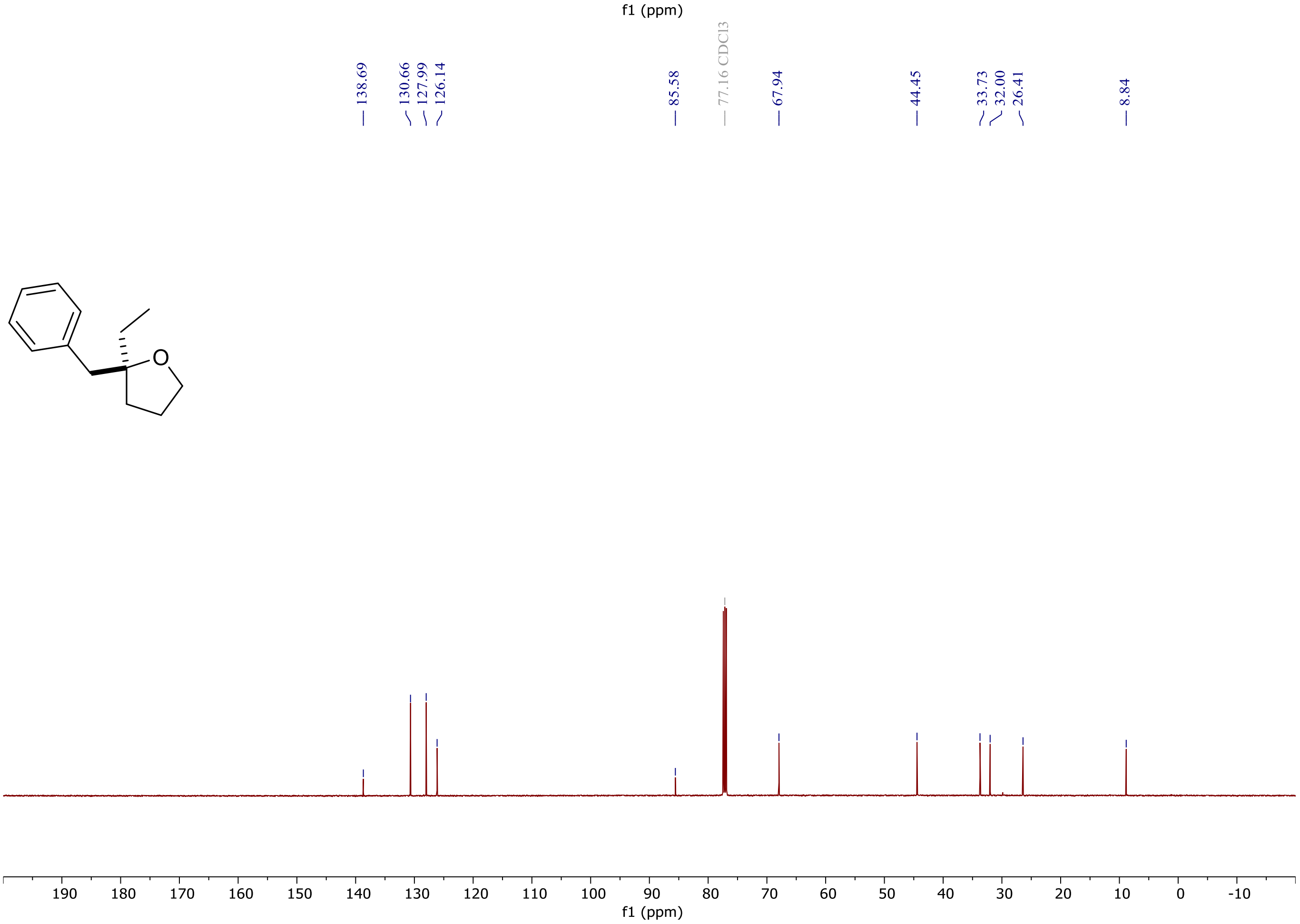
138.69
130.66
127.99
126.14
85.58
77.16 CDCl3
67.94
44.45
33.73
32.00
26.41
8.84
O
190 180 170 160 150 140 130 120 110 100 90 80 70 60 50 40 30 20 10 0 -10
f1 (ppm)

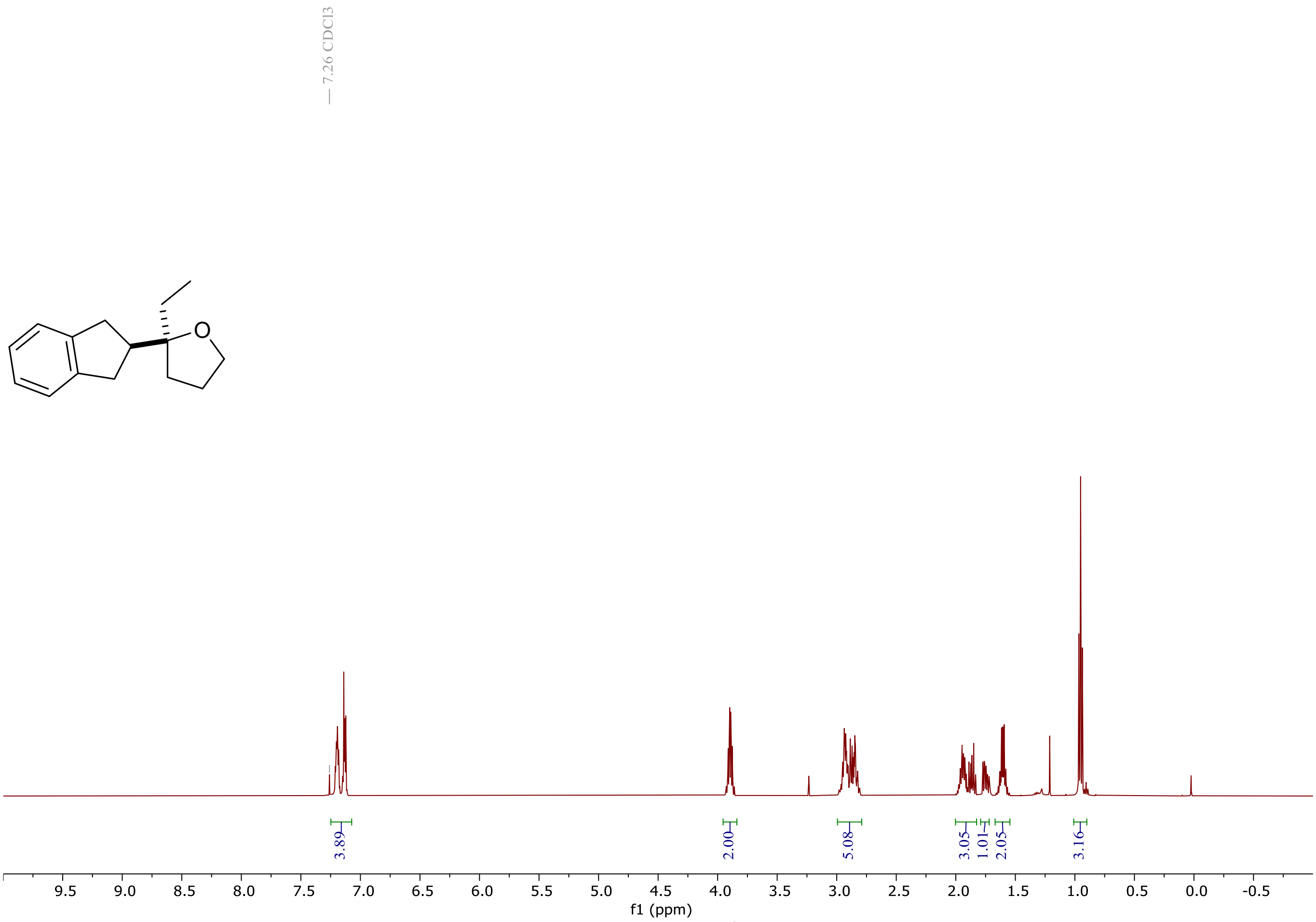
7.26 CDCl3
O
3.89
2.00
5.08
3.05
1.01
2.05
3.16
9.5 9.0 8.5 8.0 7.5 7.0 6.5 6.0 5.5 5.0 4.5 4.0 3.5 3.0 2.5 2.0 1.5 1.0 0.5 0.0 -0.5
f1 (ppm)

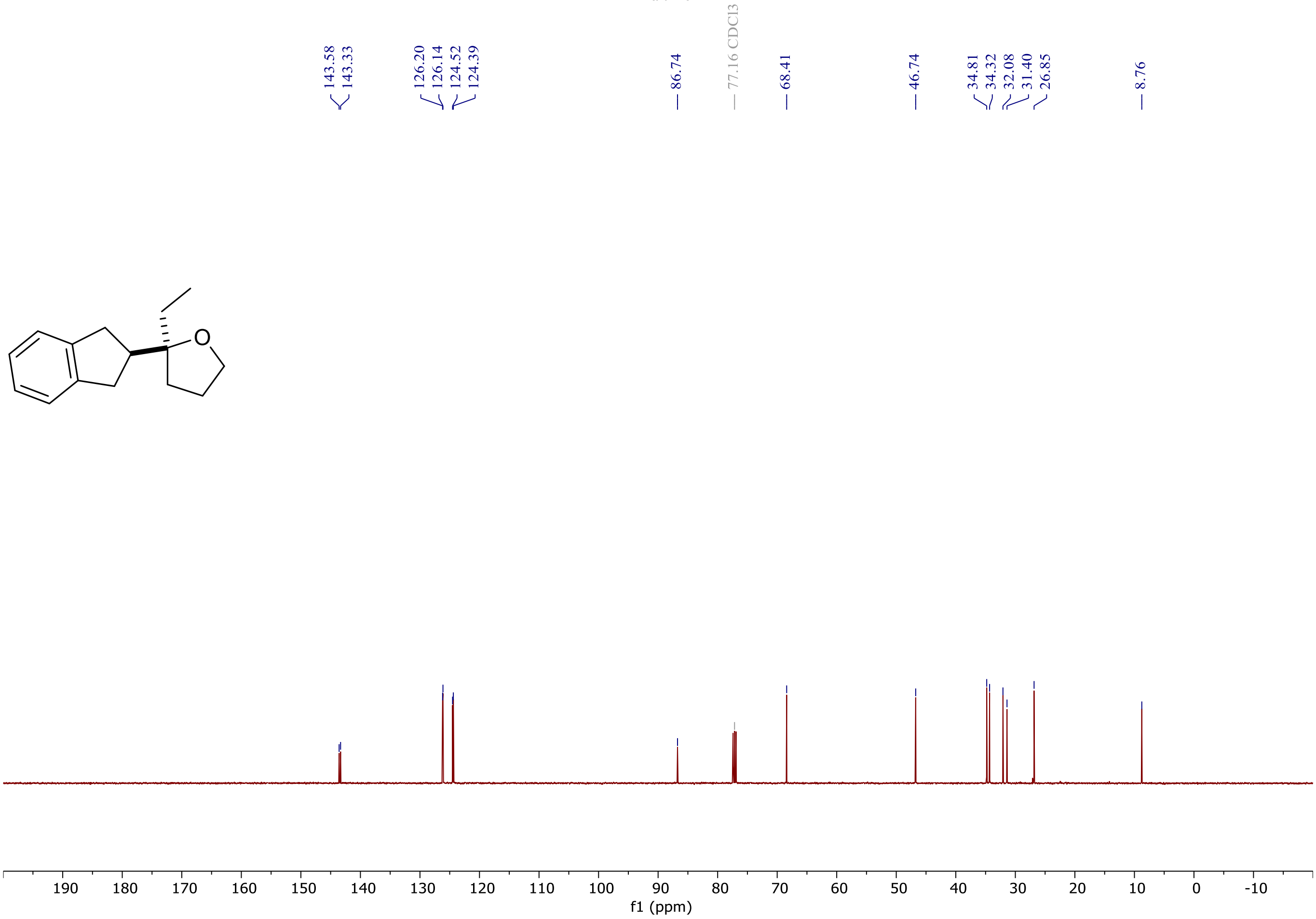
143.58
143.33
126.20
126.14
124.52
124.39
86.74
77.16 CDCl3
68.41
46.74
34.81
34.32
32.08
31.40
26.85
8.76
O
190 180 170 160 150 140 130 120 110 100 90 80 70 60 50 40 30 20 10 0 -10
f1 (ppm)

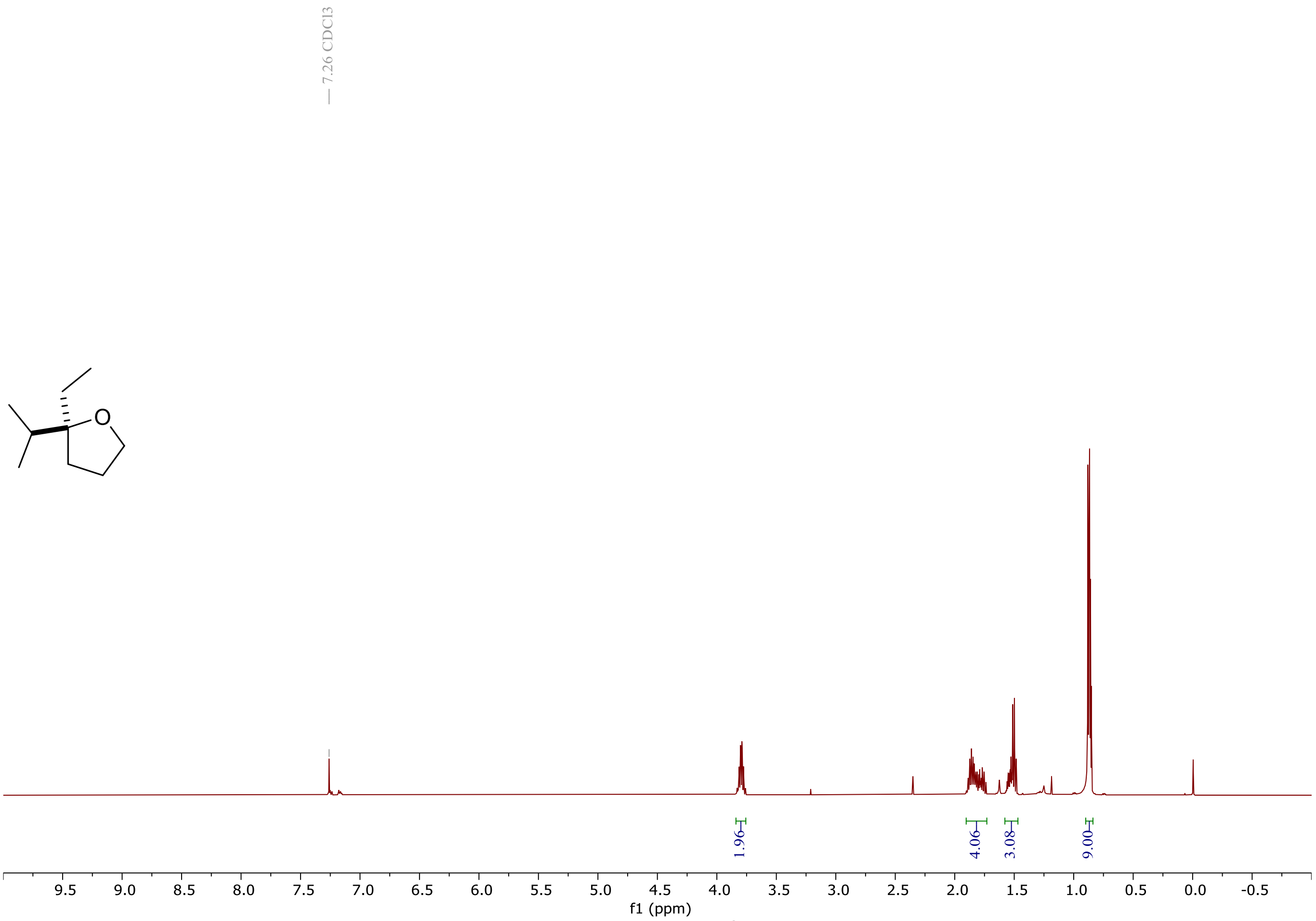

7.26 CDCl3
1.96
4.06
3.08
9.00
9.5 9.0 8.5 8.0 7.5 7.0 6.5 6.0 5.5 5.0 4.5 4.0 3.5 3.0 2.5 2.0 1.5 1.0 0.5 0.0 -0.5
f1 (ppm)

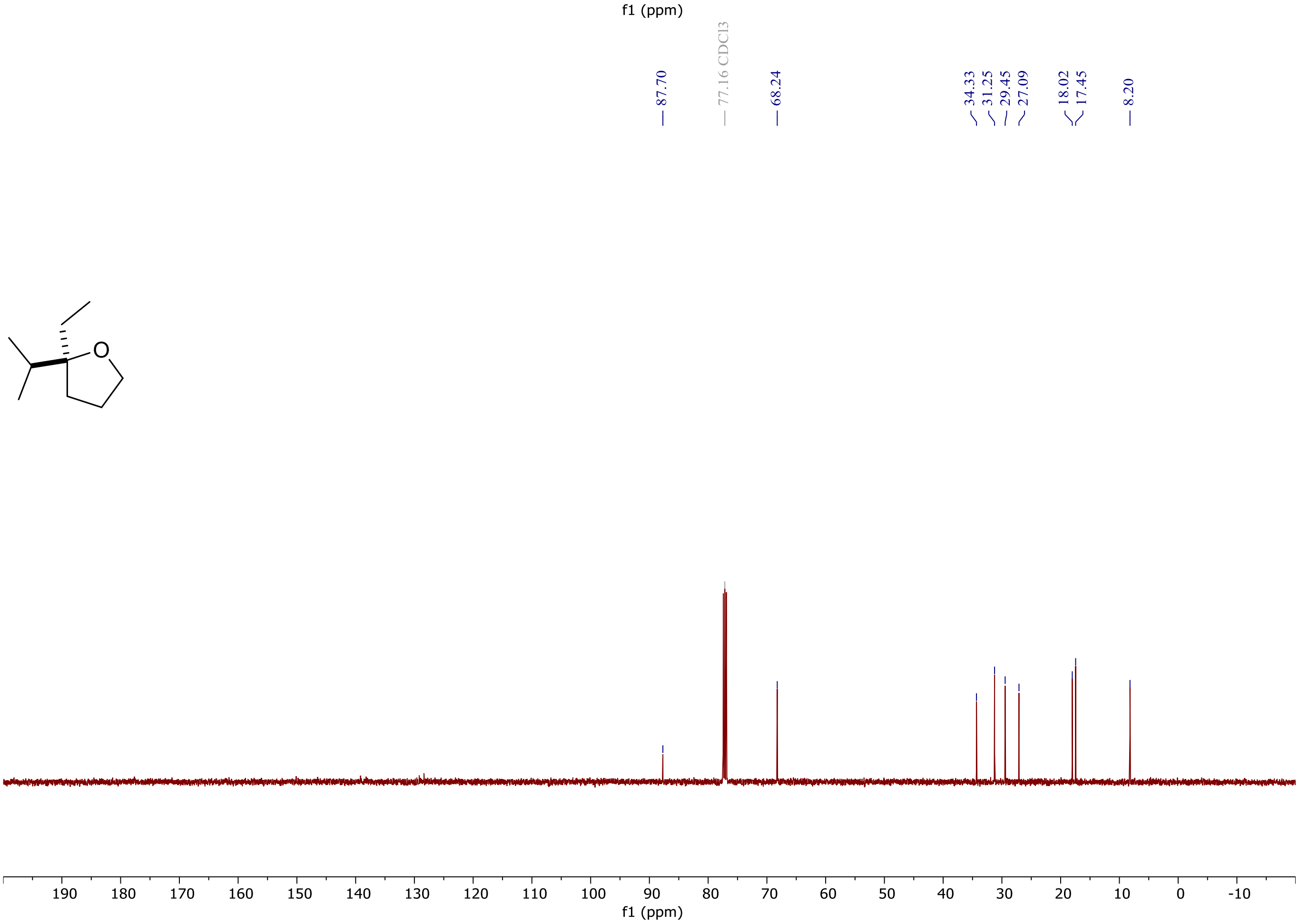

87.70
77.16 CDCl3
68.24
34.33
31.25
29.45
27.09
18.02
17.45
8.20
190 180 170 160 150 140 130 120 110 100 90 80 70 60 50 40 30 20 10 0 -10
f1 (ppm)

Based on the volatility of the substrates **3l**, it is impossible to completely remove the Et_2O solvent from the result.

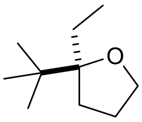

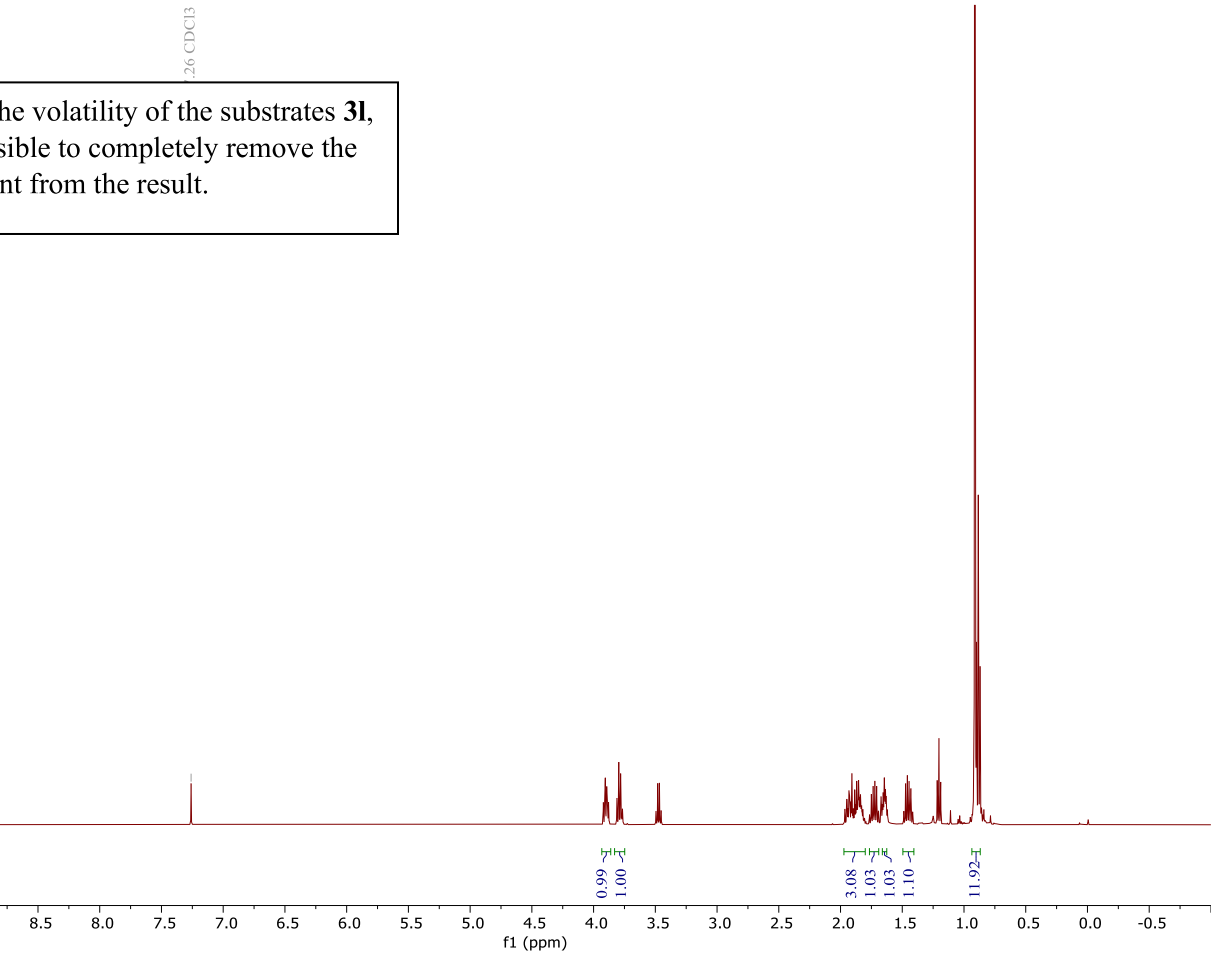

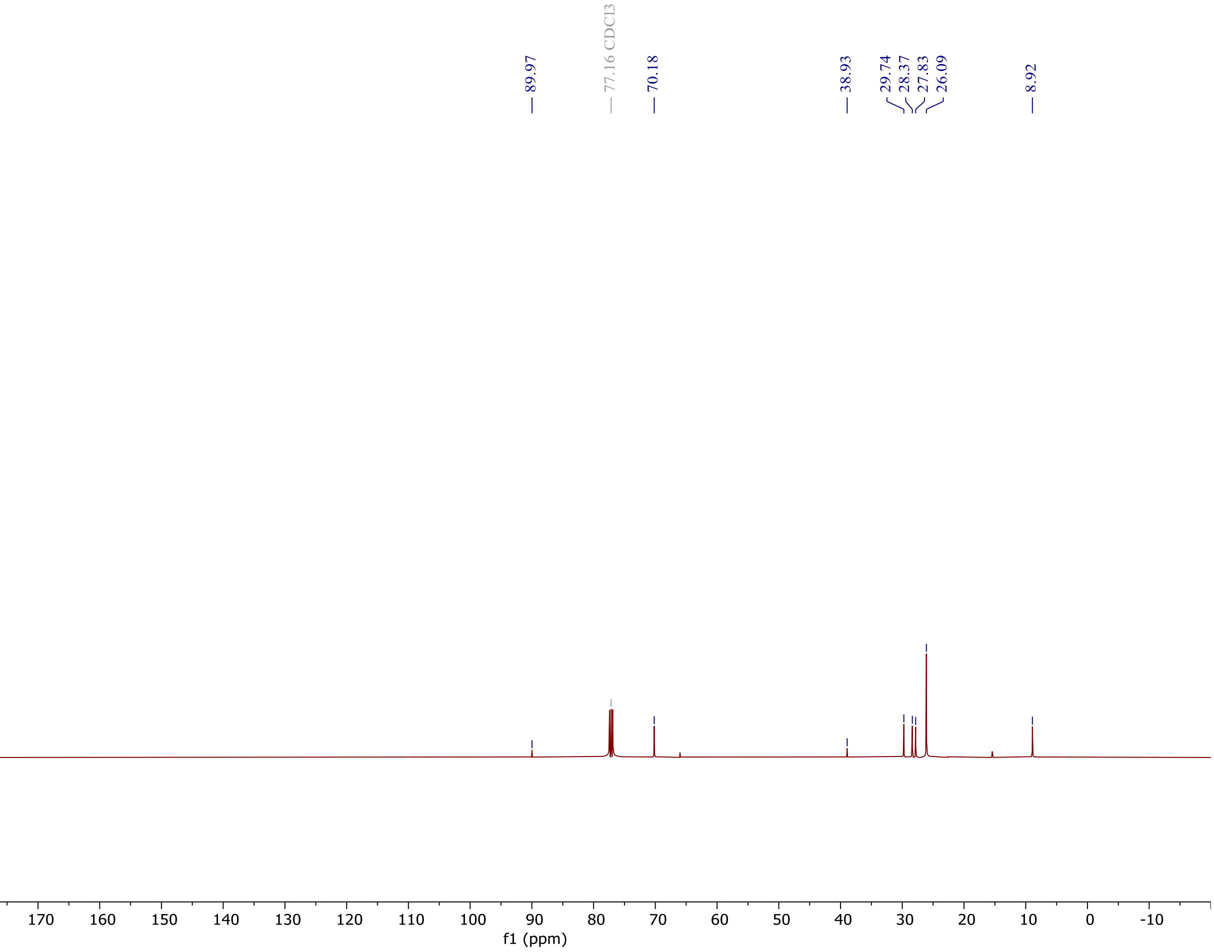

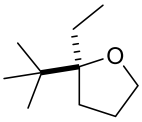

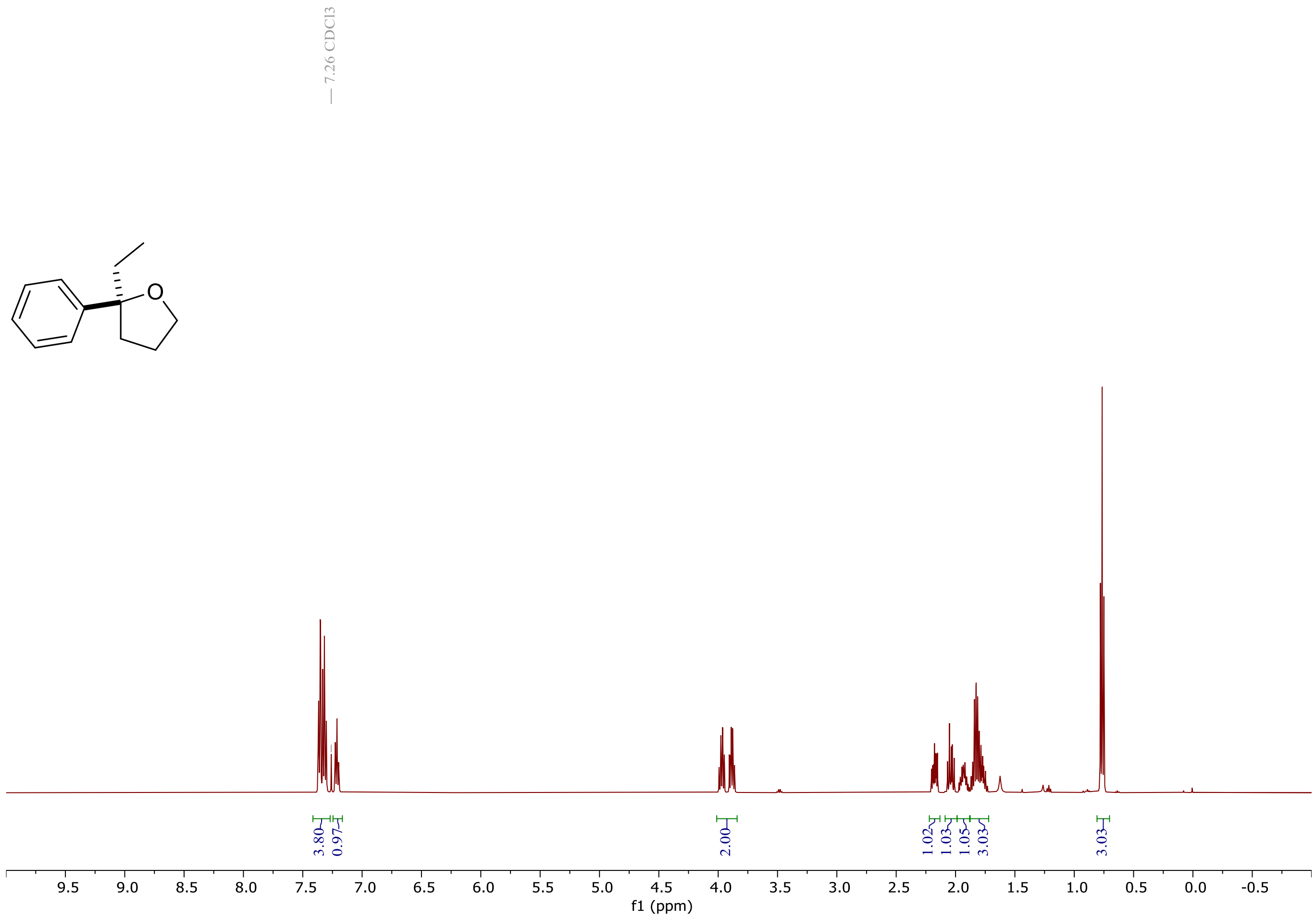

7.26 CDCl3
O
3.80
0.97
2.00
1.02
1.03
1.05
3.03
3.03
9.5 9.0 8.5 8.0 7.5 7.0 6.5 6.0 5.5 5.0 4.5 4.0 3.5 3.0 2.5 2.0 1.5 1.0 0.5 0.0 -0.5
f1 (ppm)

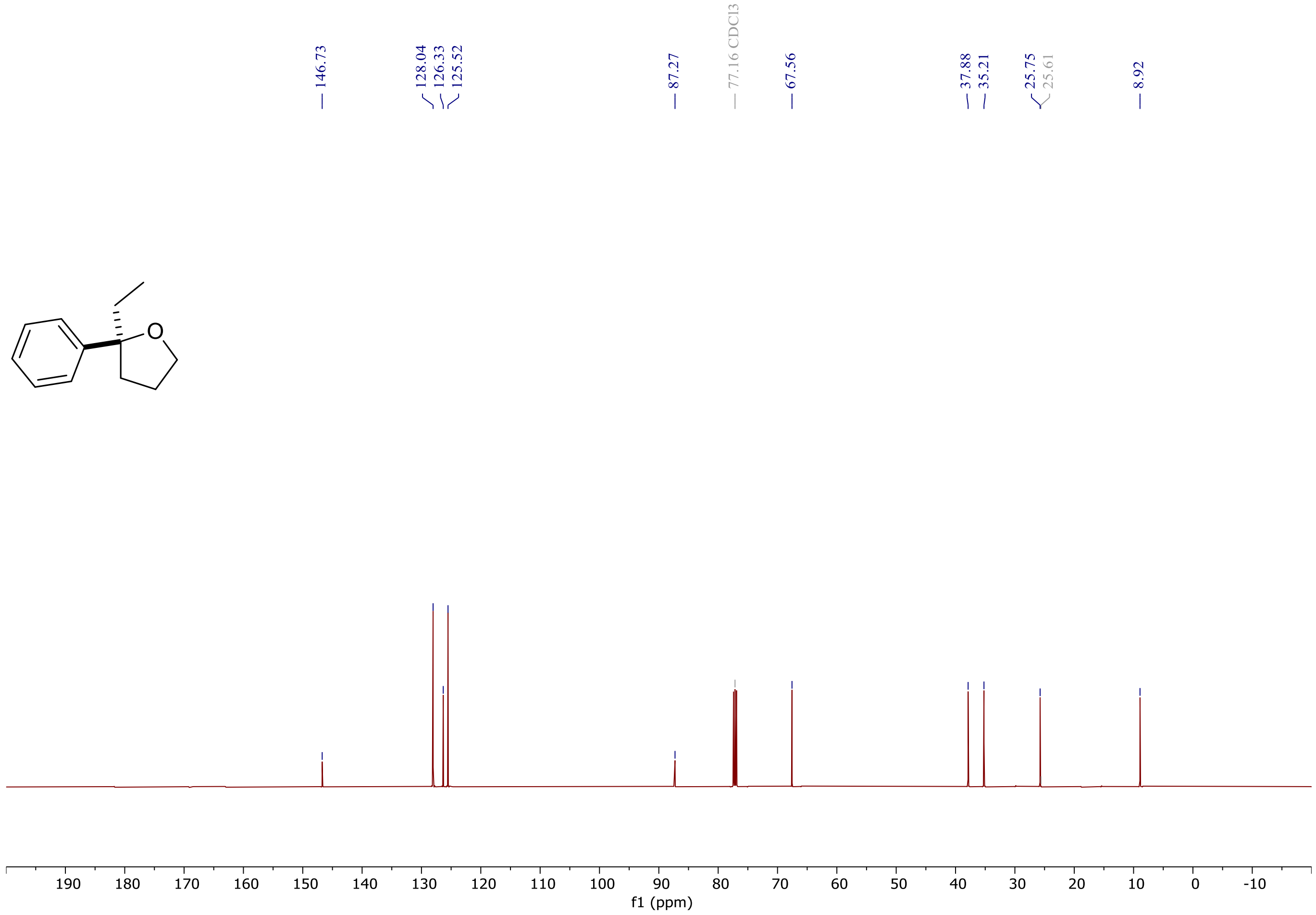

146.73
128.04
126.33
125.52
87.27
77.16 CDCl3
67.56
37.88
35.21
25.75
25.61
8.92
O
190 180 170 160 150 140 130 120 110 100 90 80 70 60 50 40 30 20 10 0 -10
f1 (ppm)

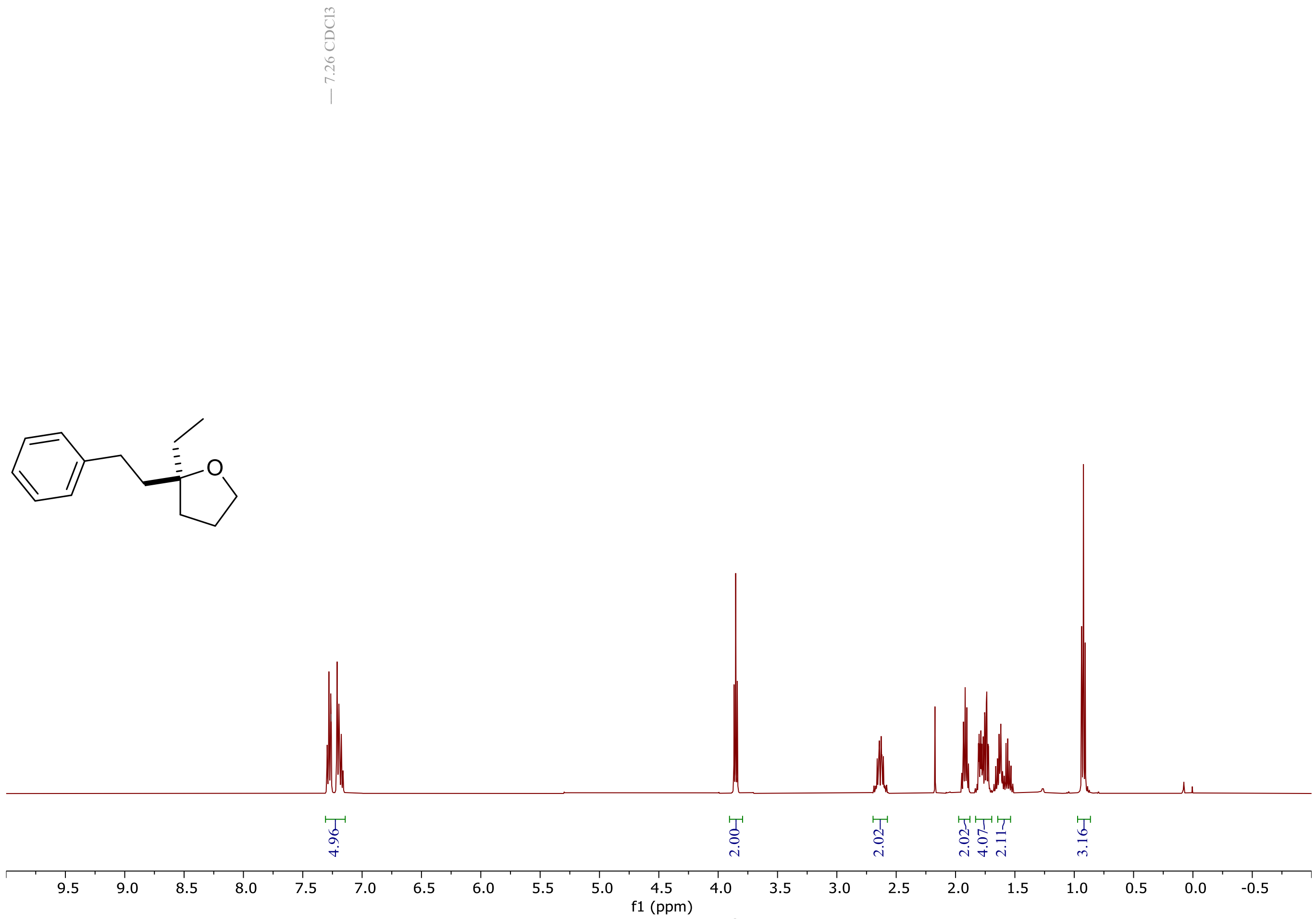
7.26 CDCl3
O
4.96
2.00
2.02
2.02
4.07
2.11
3.16
9.5 9.0 8.5 8.0 7.5 7.0 6.5 6.0 5.5 5.0 4.5 4.0 3.5 3.0 2.5 2.0 1.5 1.0 0.5 0.0 -0.5
f1 (ppm)

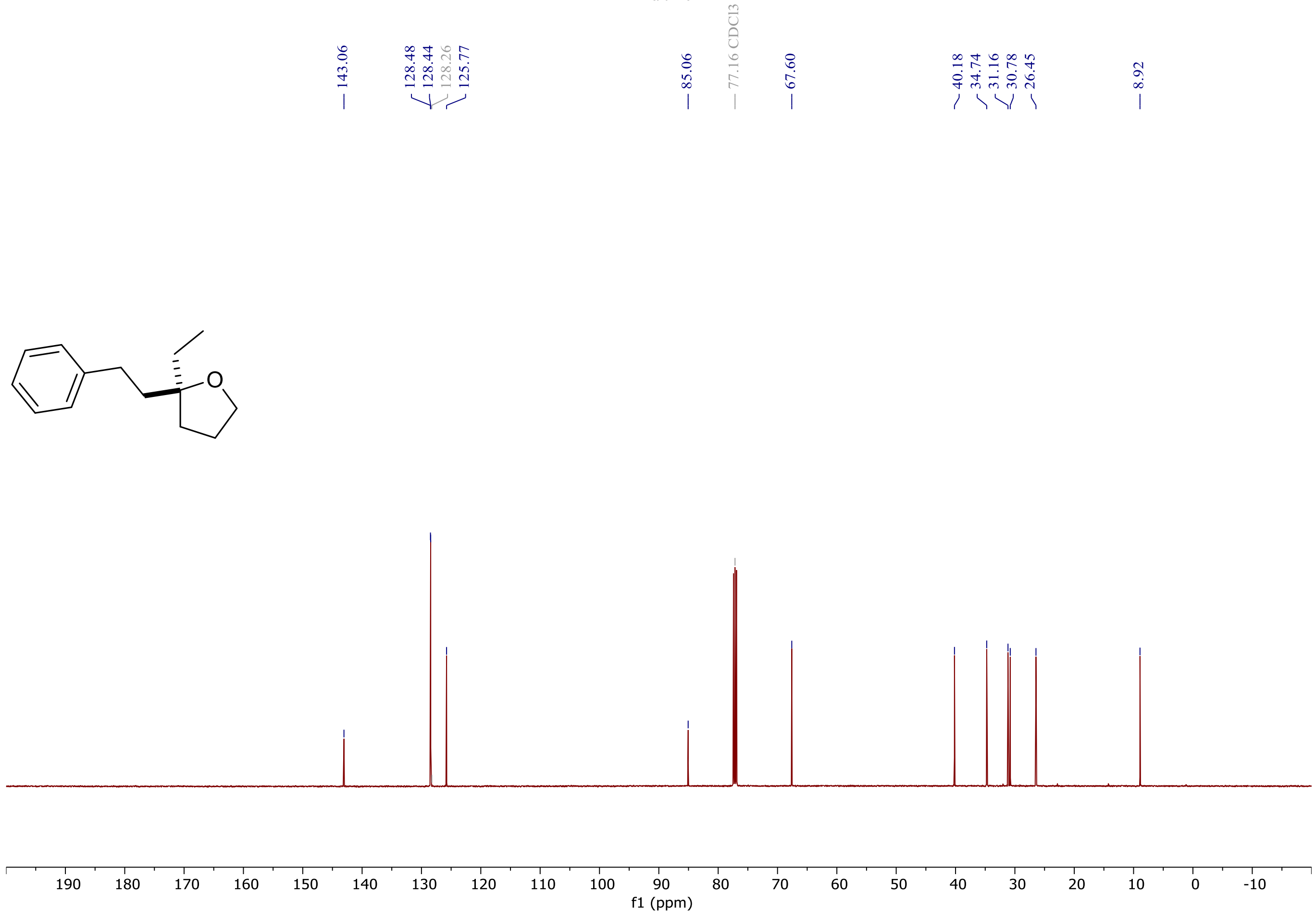
143.06
128.48
128.44
128.26
125.77
85.06
77.16 CDCl3
67.60
40.18
34.74
31.16
30.78
26.45
8.92
O
190 180 170 160 150 140 130 120 110 100 90 80 70 60 50 40 30 20 10 0 -10
f1 (ppm)

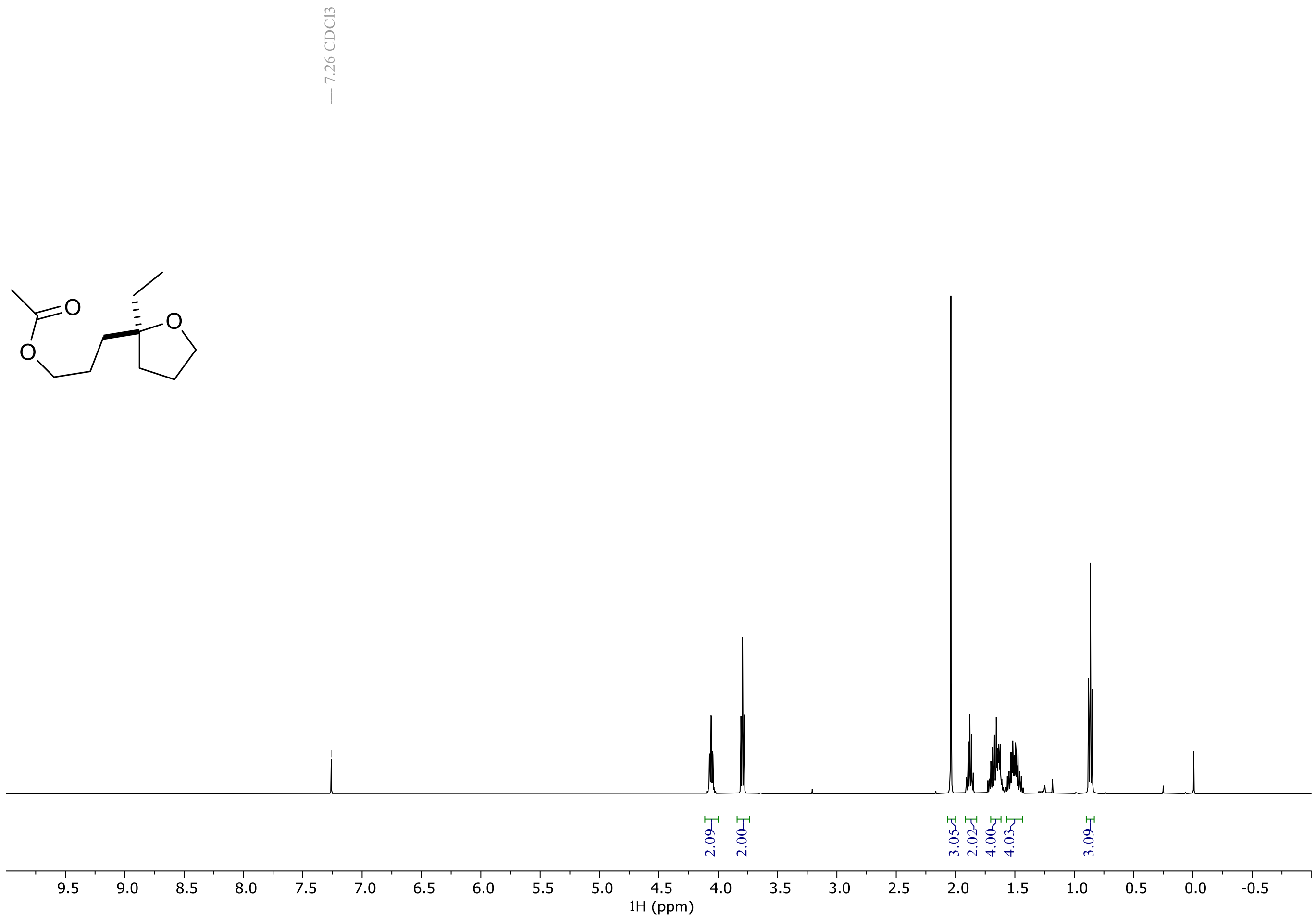

7.26 CDCl3
O
O
O
2.09
2.00
3.05
2.02
4.00
4.03
3.09
9.5 9.0 8.5 8.0 7.5 7.0 6.5 6.0 5.5 5.0 4.5 4.0 3.5 3.0 2.5 2.0 1.5 1.0 0.5 0.0 -0.5
1H (ppm)

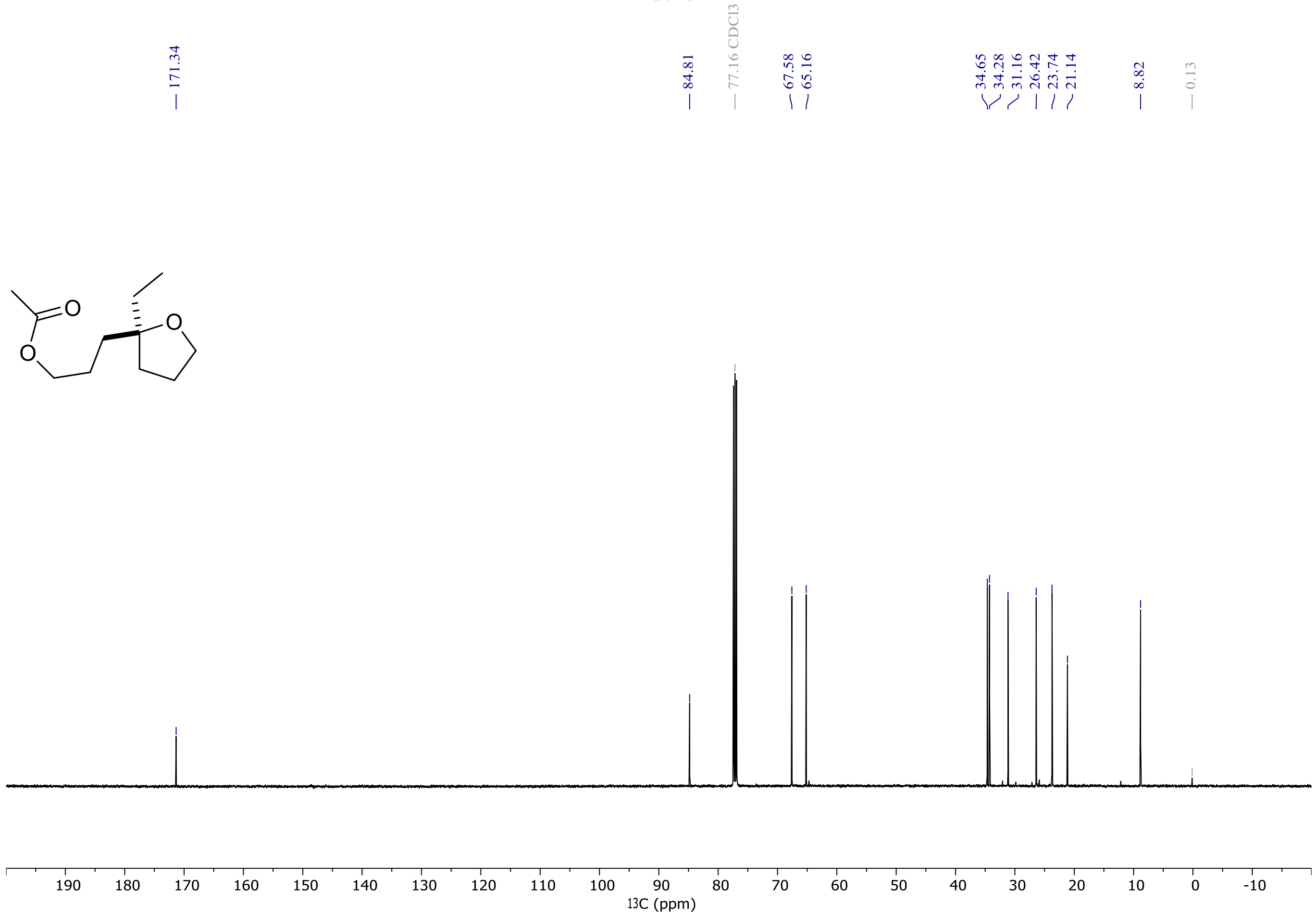

171.34
84.81
77.16 CDCl3
67.58
65.16
34.65
34.28
31.16
26.42
23.74
21.14
8.82
0.13
O
O
O
190 180 170 160 150 140 130 120 110 100 90 80 70 60 50 40 30 20 10 0 -10
13C (ppm)

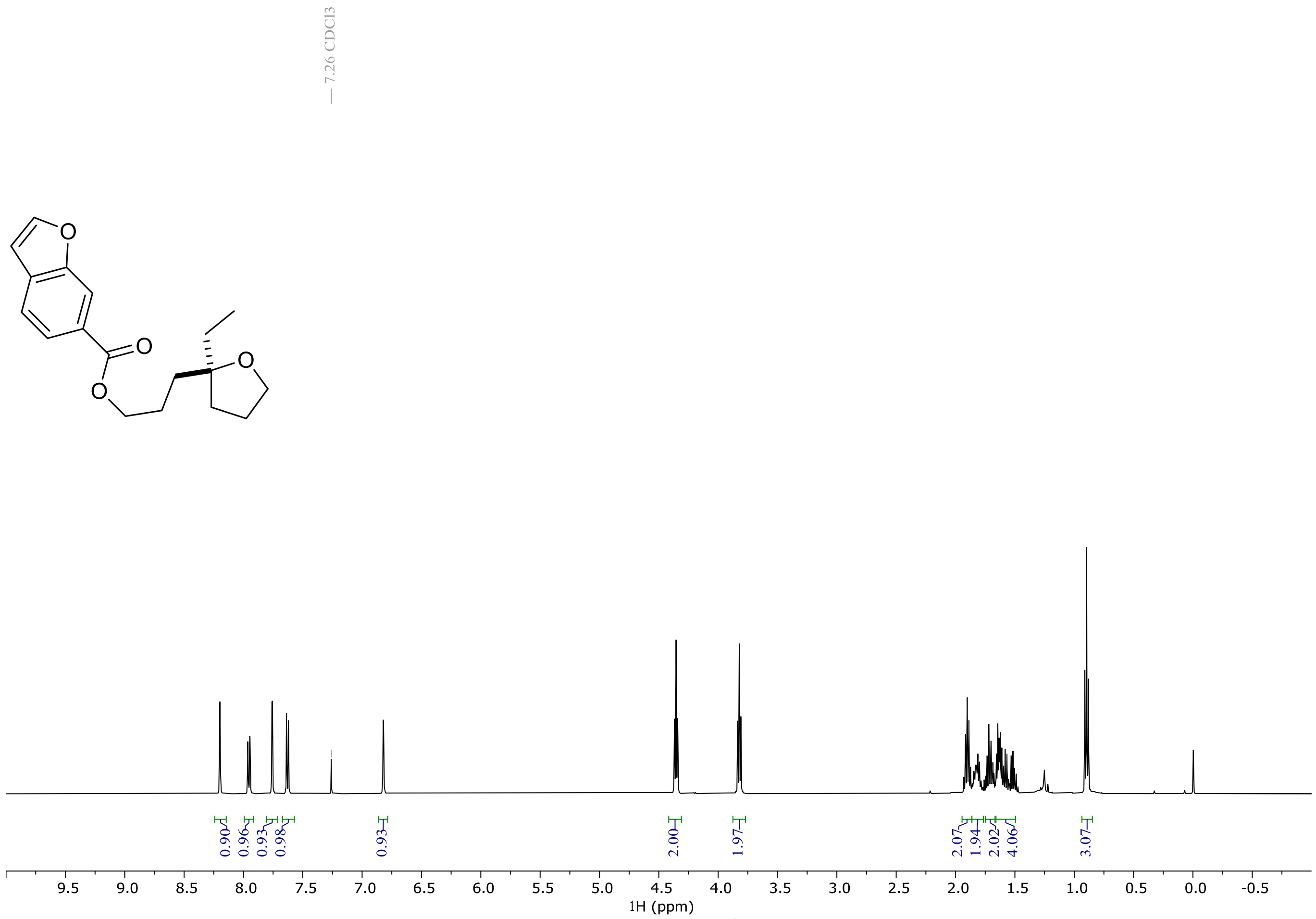
7.26 CDCl3
0.90
0.96
0.93
0.98
0.93
2.00
1.97
2.07
1.94
2.02
4.06
3.07
1H (ppm)

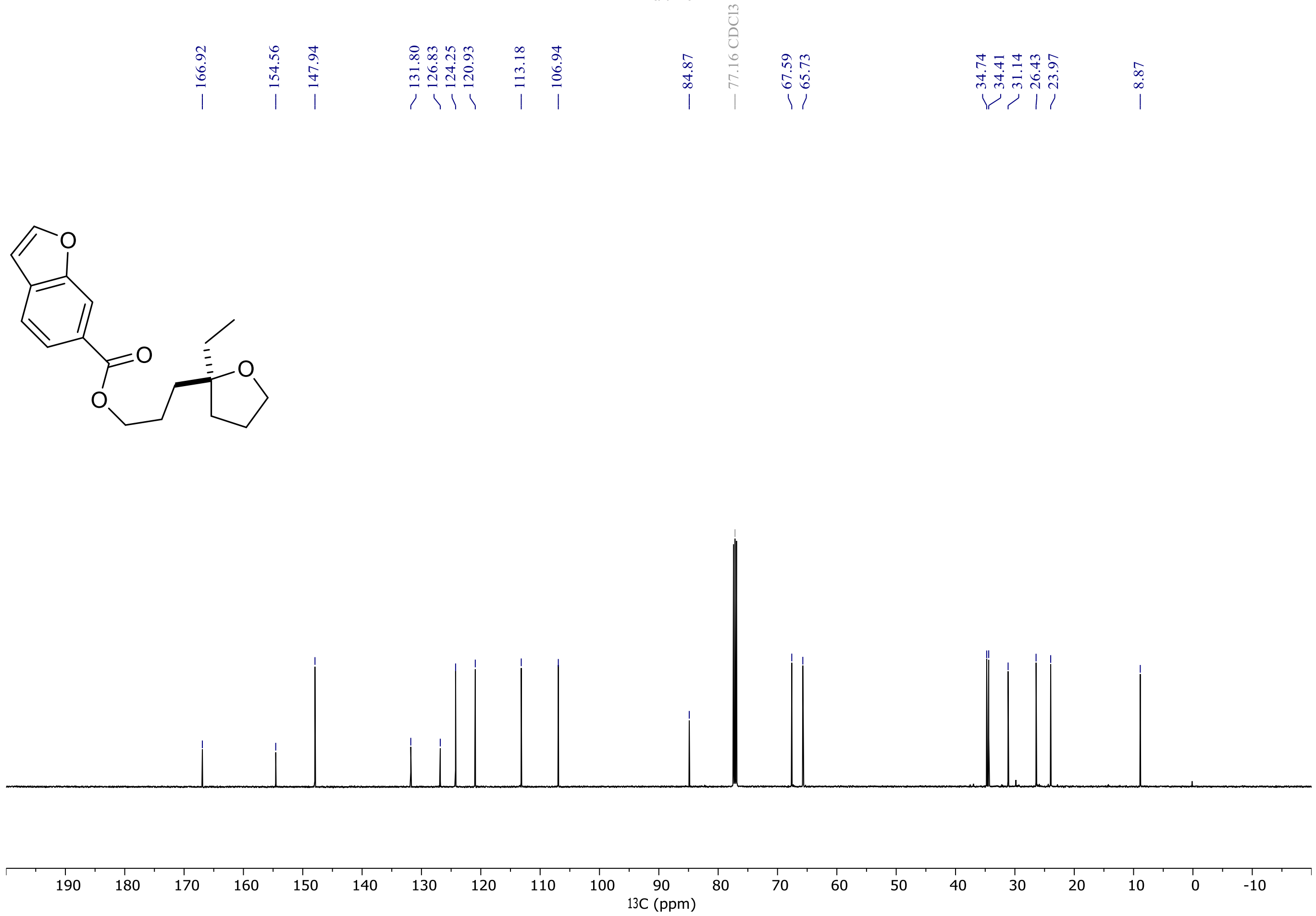
166.92
154.56
147.94
131.80
126.83
124.25
120.93
113.18
106.94
84.87
77.16 CDCl3
67.59
65.73
34.74
34.41
31.14
26.43
23.97
8.87
13C (ppm)

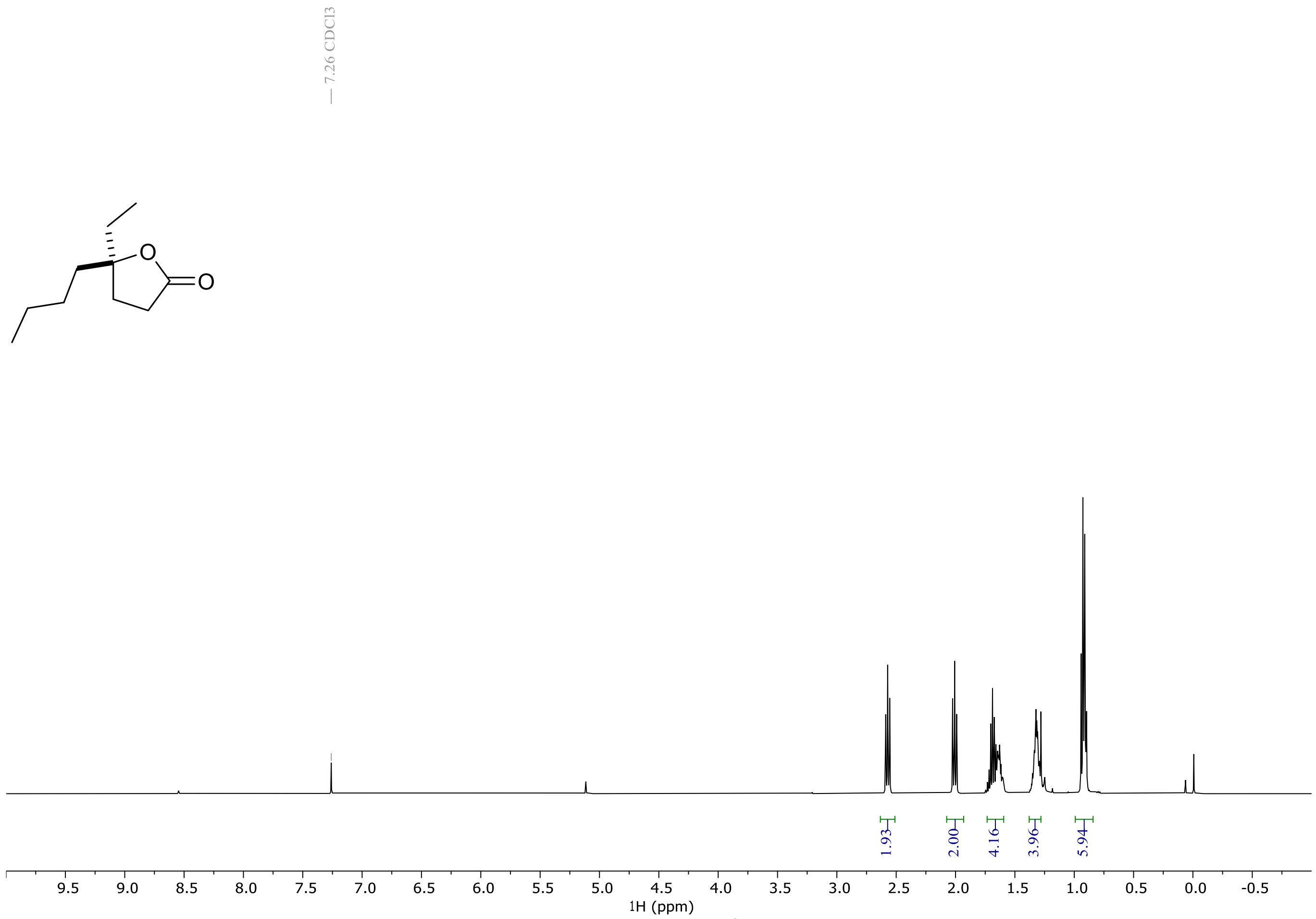

7.26 CDCl3
O
O
1.93
2.00
4.16
3.96
5.94
9.5 9.0 8.5 8.0 7.5 7.0 6.5 6.0 5.5 5.0 4.5 4.0 3.5 3.0 2.5 2.0 1.5 1.0 0.5 0.0 -0.5
1H (ppm)

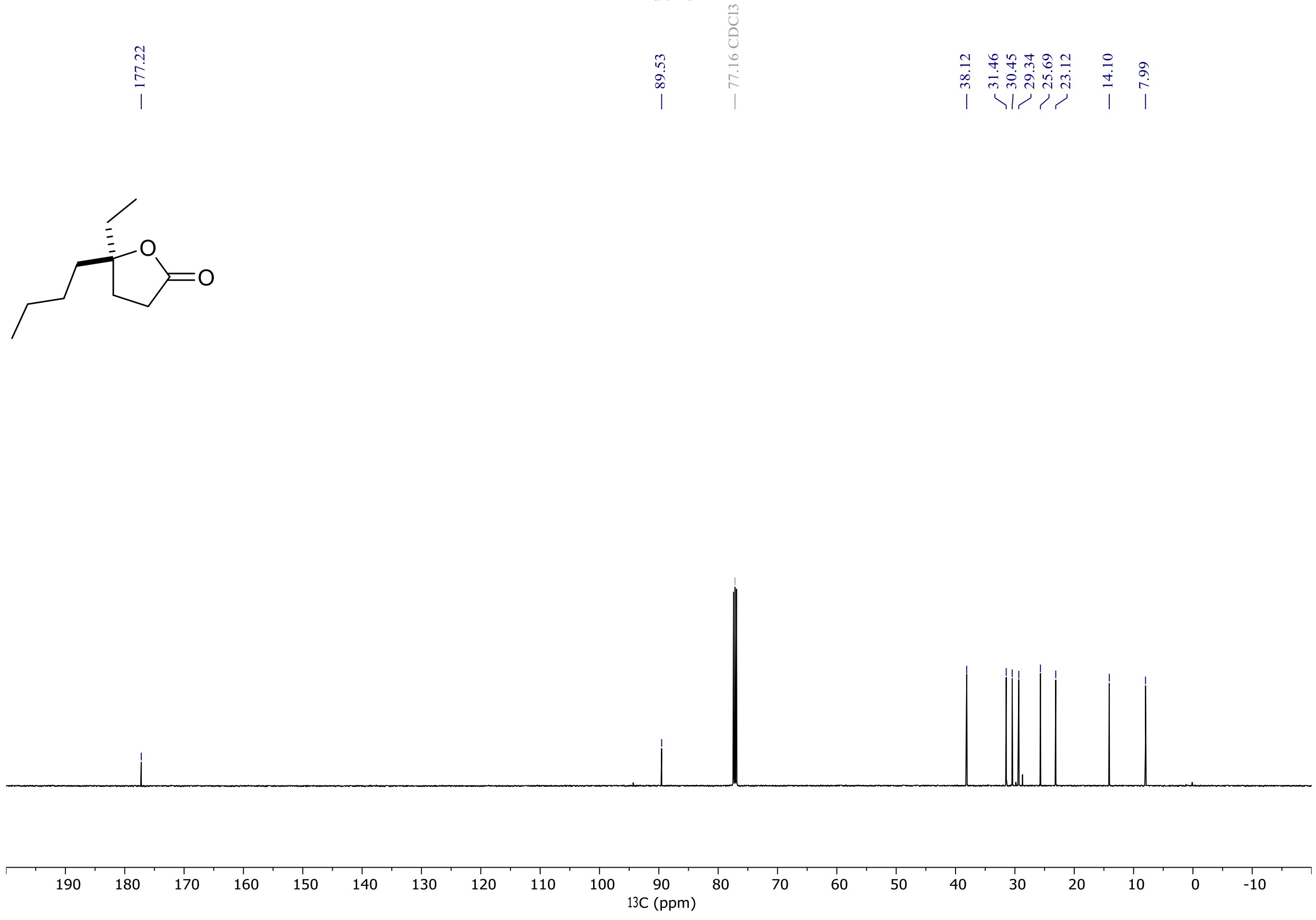

177.22
89.53
77.16 CDCl3
38.12
31.46
30.45
29.34
25.69
23.12
14.10
7.99
O
O
190 180 170 160 150 140 130 120 110 100 90 80 70 60 50 40 30 20 10 0 -10
13C (ppm)

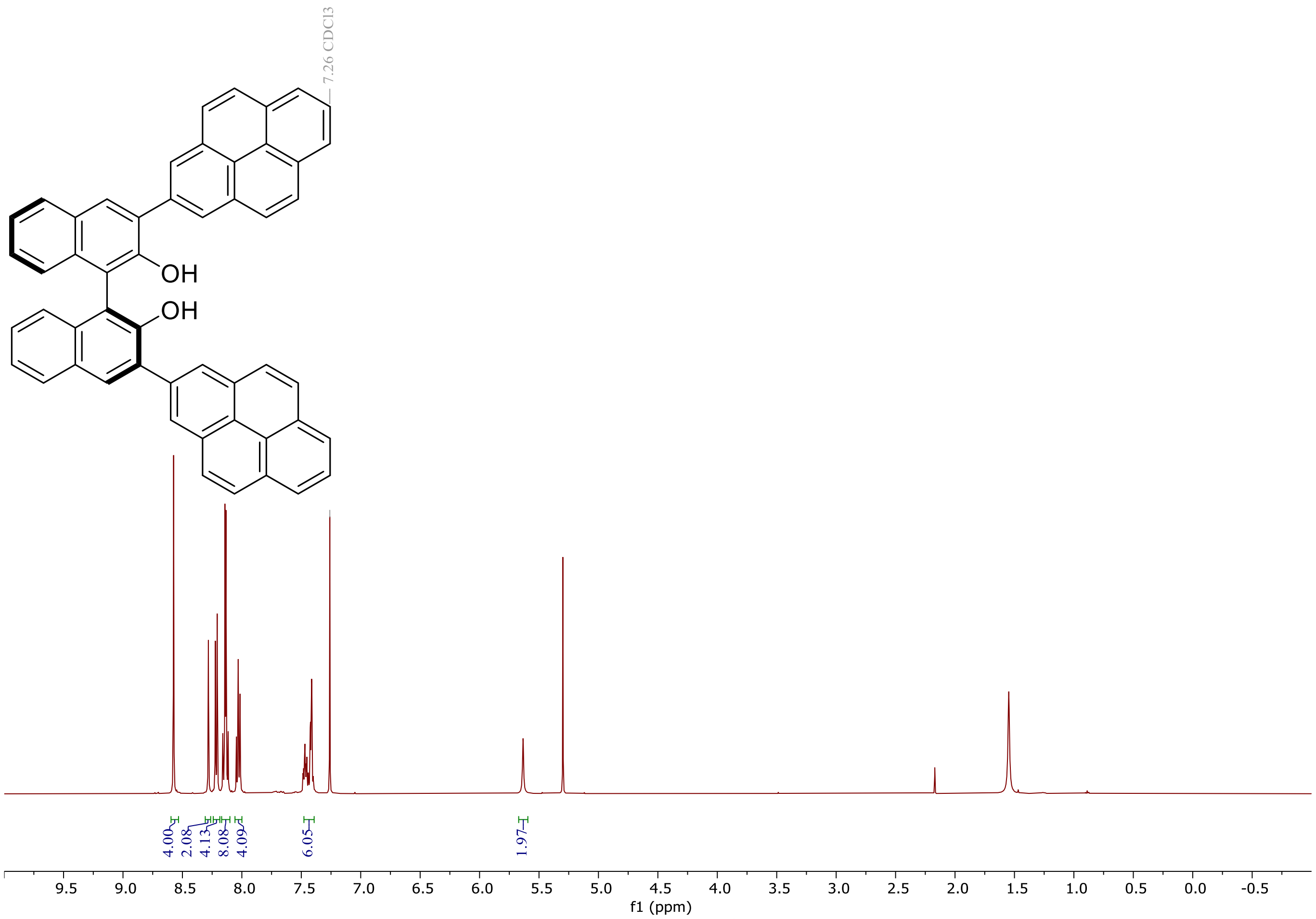
7.26 CDCl3
OH
OH
4.00
2.08
4.13
8.08
4.09
6.05
1.97
f1 (ppm)

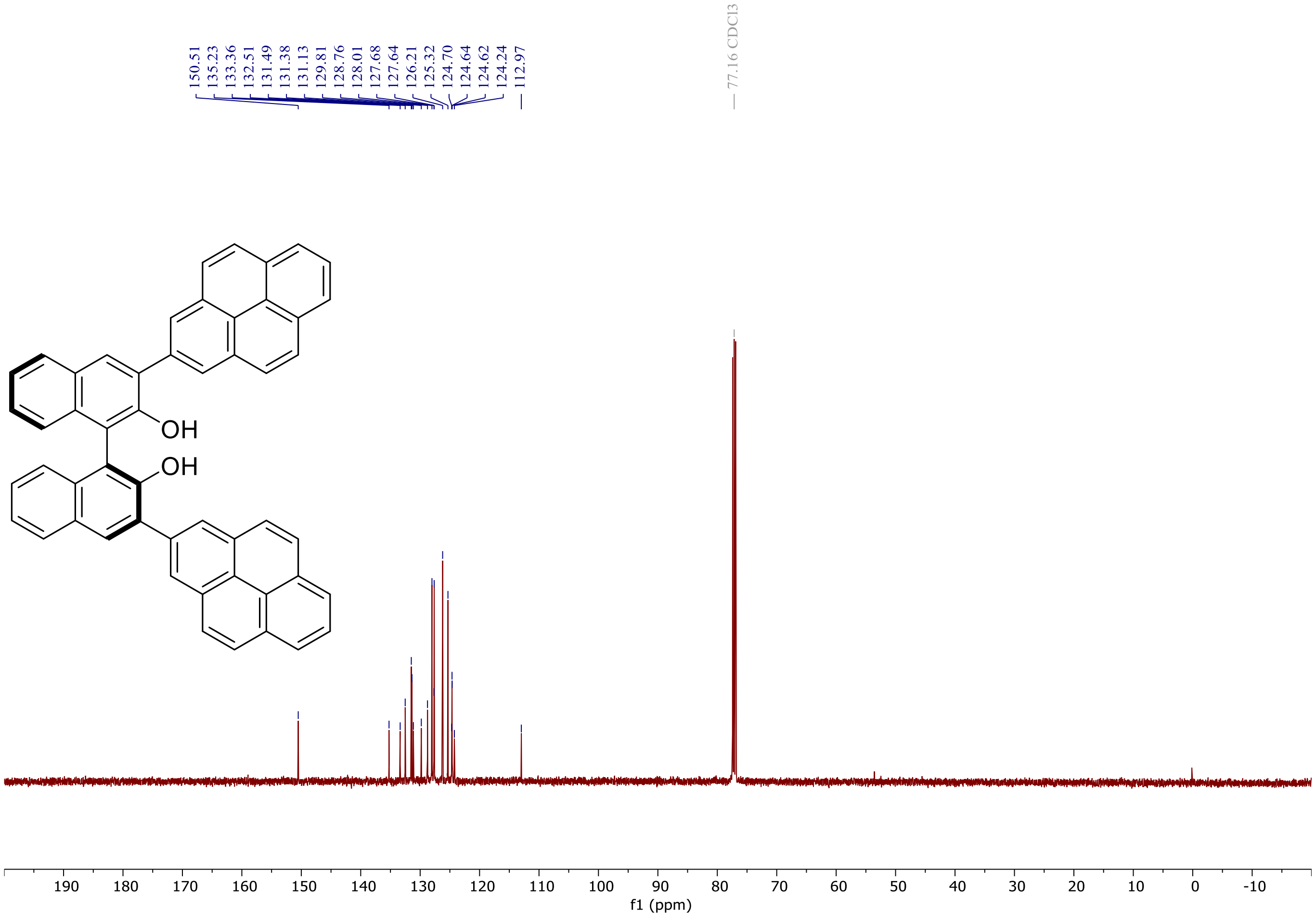
150.51
135.23
133.36
132.51
131.49
131.38
131.13
129.81
128.76
128.01
127.68
127.64
126.21
125.32
124.70
124.64
124.62
124.24
112.97
77.16 CDCl3
OH
OH
f1 (ppm)

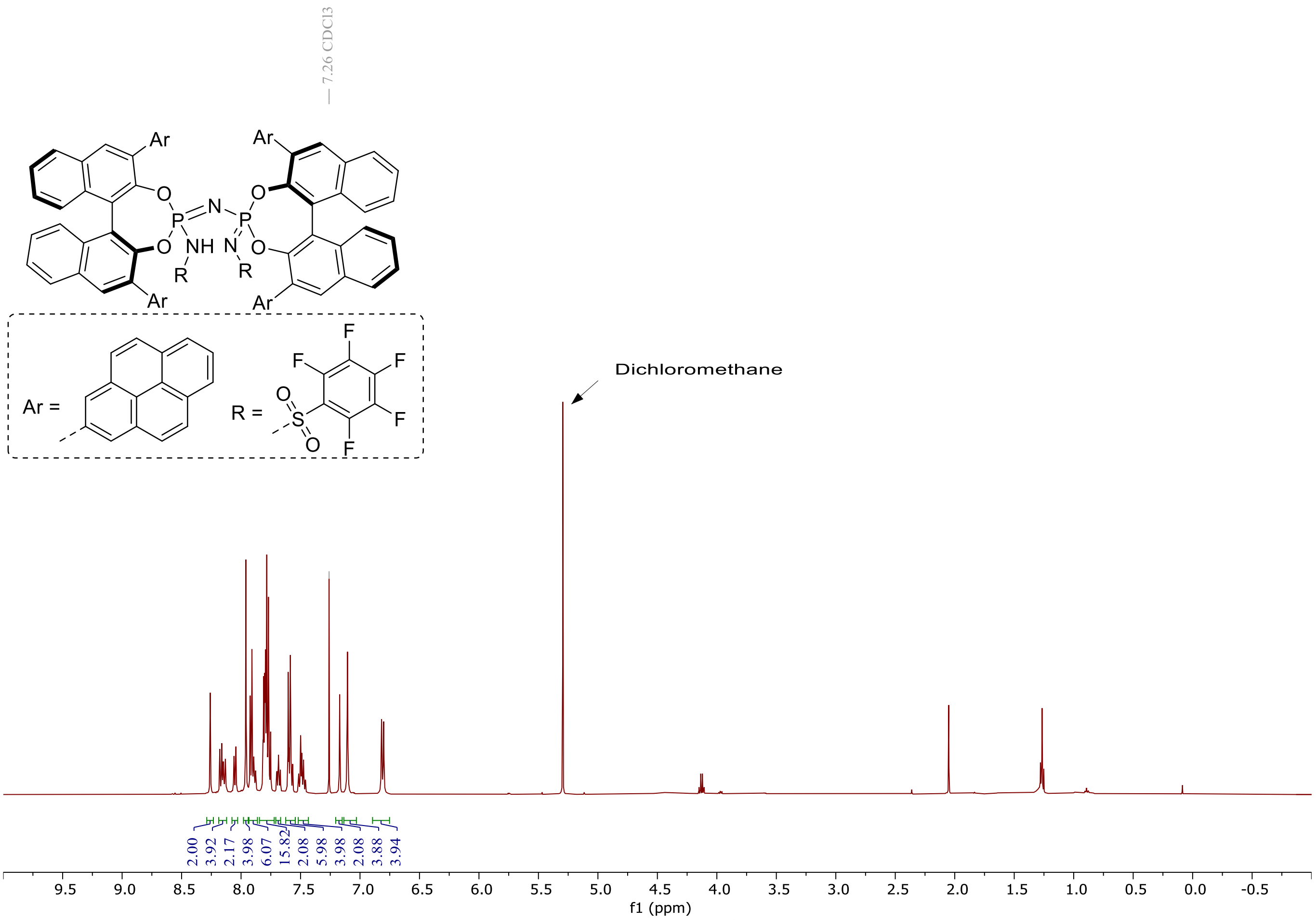

7.26 CDCl3
Ar
O
P
N
NH
R
Ar =
R =
F
S
Dichloromethane
2.00
3.92
2.17
3.98
6.07
15.82
2.08
5.98
3.98
2.08
3.88
3.94
9.5
9.0
8.5
8.0
7.5
7.0
6.5
6.0
5.5
5.0
4.5
4.0
3.5
3.0
2.5
2.0
1.5
1.0
0.5
0.0
-0.5
f1 (ppm)

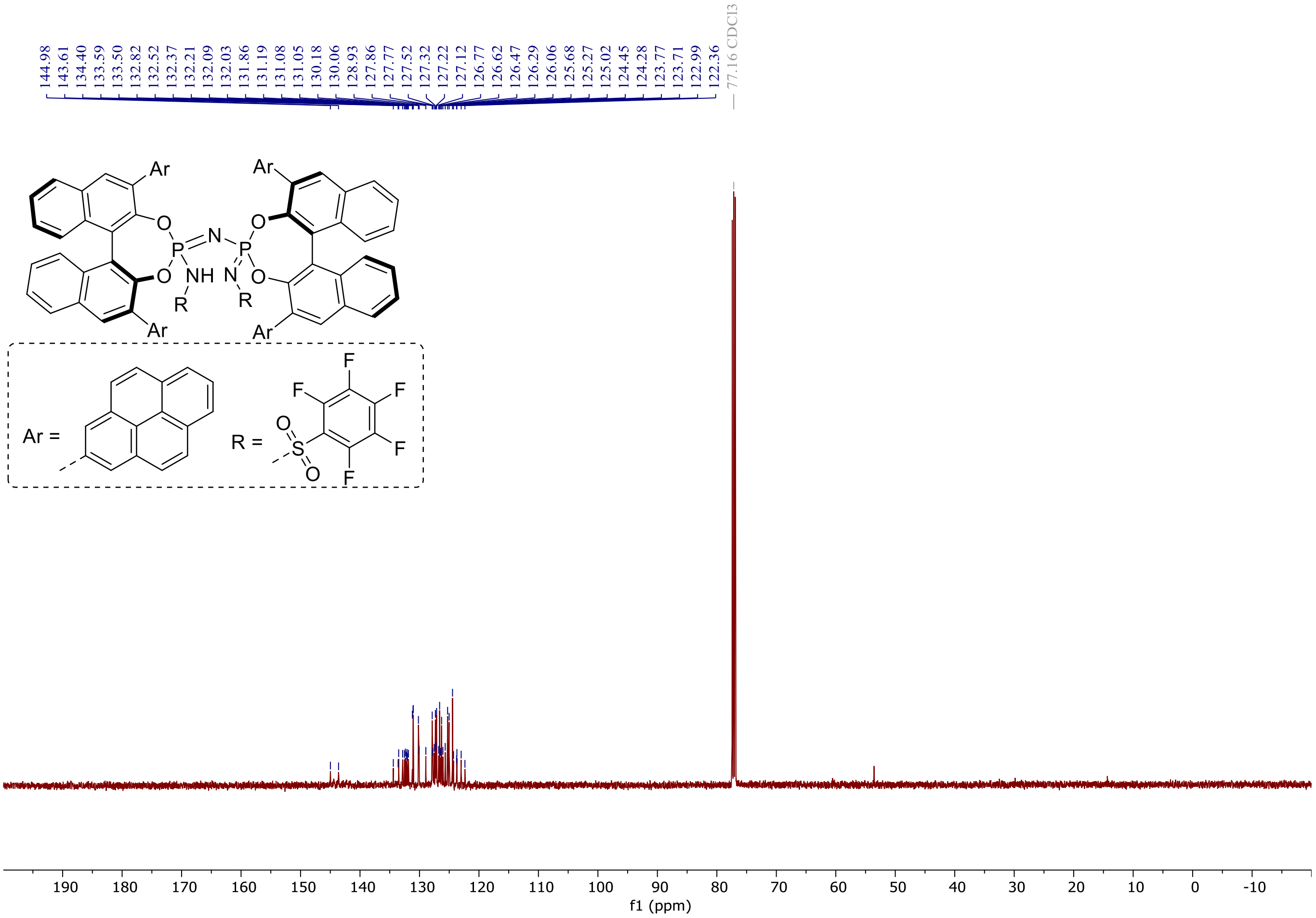

144.98
143.61
134.40
133.59
133.50
132.82
132.52
132.37
132.21
132.09
132.03
131.86
131.19
131.08
131.05
130.18
130.06
128.93
127.86
127.77
127.52
127.32
127.22
127.12
126.77
126.62
126.47
126.29
126.06
125.68
125.27
125.02
124.45
124.28
123.77
123.71
122.99
122.36
77.16 CDCl3
Ar
O
P
N
NH
R
Ar =
R =
F
S
190
180
170
160
150
140
130
120
110
100
90
80
70
60
50
40
30
20
10
0
-10
f1 (ppm)

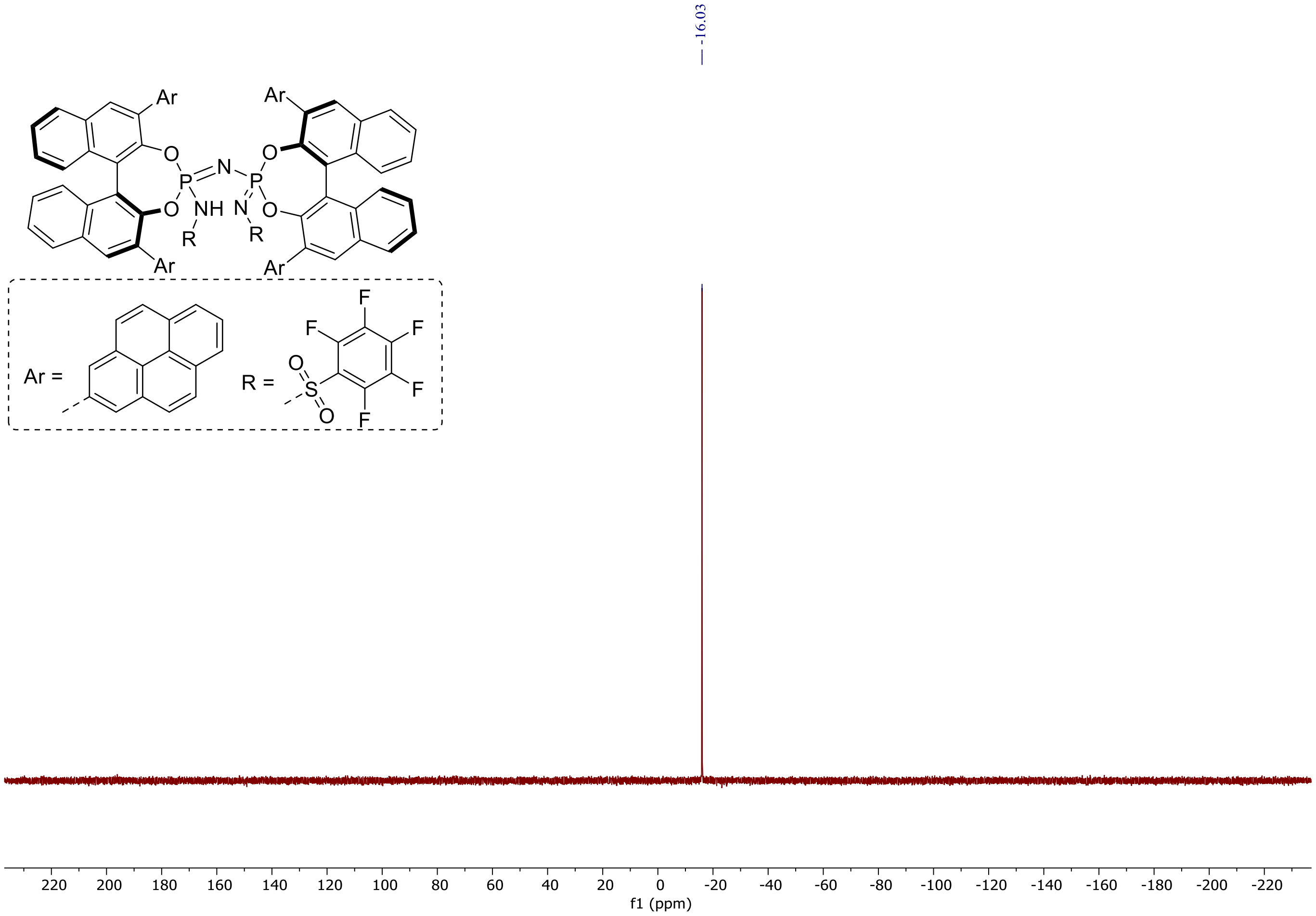
-16.03
Ar
O
P
N
NH
R
Ar =
R =
S
F
220 200 180 160 140 120 100 80 60 40 20 0 -20 -40 -60 -80 -100 -120 -140 -160 -180 -200 -220
f1 (ppm)

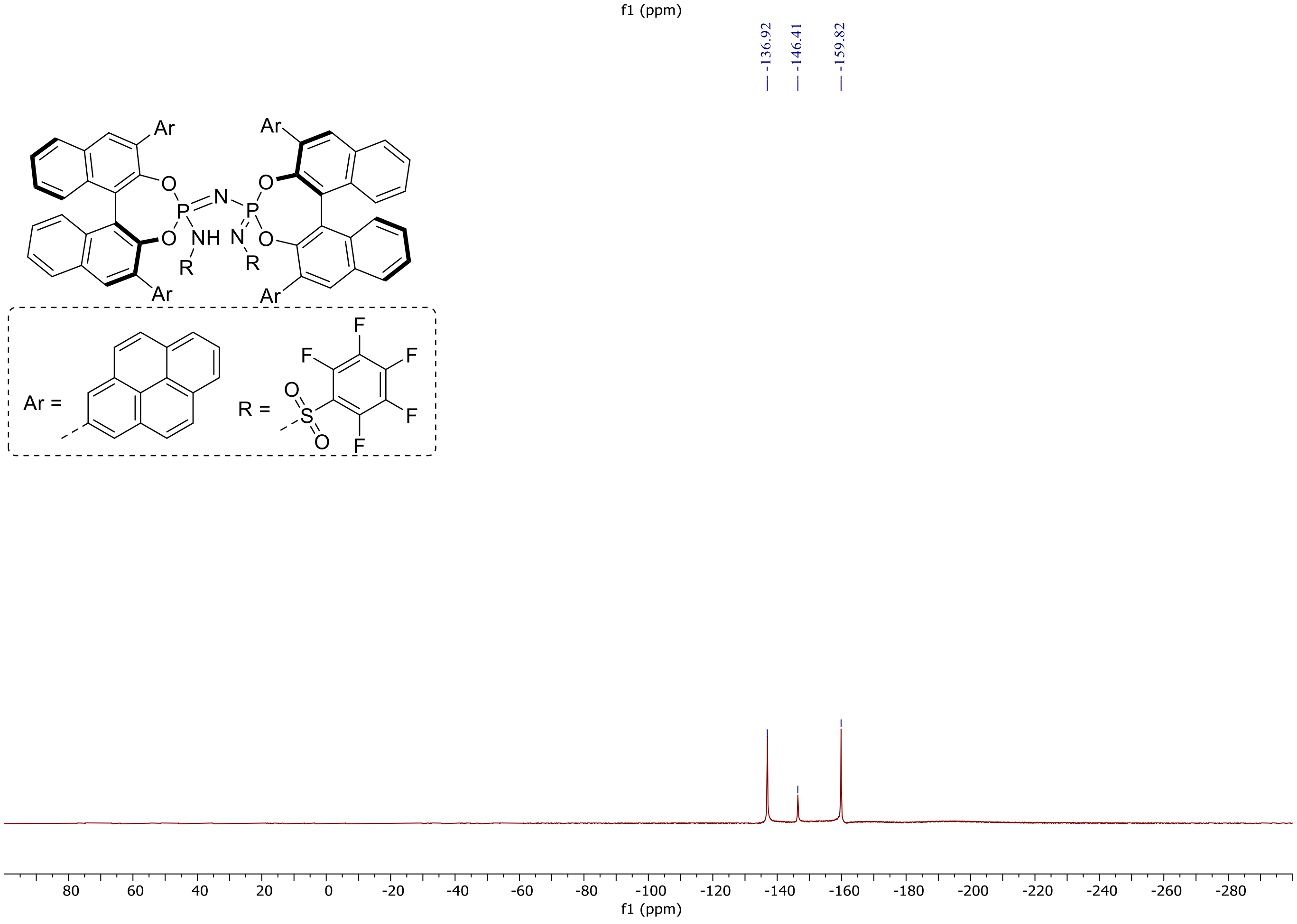
-136.92
-146.41
-159.82
Ar
O
P
N
NH
R
Ar =
R =
S
F
80 60 40 20 0 -20 -40 -60 -80 -100 -120 -140 -160 -180 -200 -220 -240 -260 -280
f1 (ppm)

9. Computational Studies

9. 1. General

All DFT studies were carried out with a development version of ORCA 5.0.[4] Initial conformers were generated using CREST at the GFN2-xTB level.[5] To ensure adequate exploration of the conformational space, multiple distinct arrangements of the key intermediate were constructed manually and used as independent starting points for conformational sampling.[6] For transition-state conformational sampling, distance constraints were applied to the two bonds being formed or broken in order to maintain the system along the relevant reaction coordinate.[7] The ten lowest-energy conformers were subsequently geometry-optimized at the PBE0/def2-SVP level of theory, followed by single-point energy calculations at the PBE0-D3/def2-TZVP level.[8-10] Solvation effects were treated using the conductor-like polarizable continuum model (CPCM) with toluene as the solvent (ε = 2.4, refrac = 1.497). The DefGrid3 grid was used to avoid the emergence of spurious imaginary frequencies.[11, 12] Similar levels of theory have been successfully used in the study of other confinement catalysts.[7, 13] The RIJCOSX approximations were used for all PBE0 calculations, using the def2/J auxiliary basis set.[14-16]

9. 2. Independent gradient model based on Hirshfeld partition (IGMH)

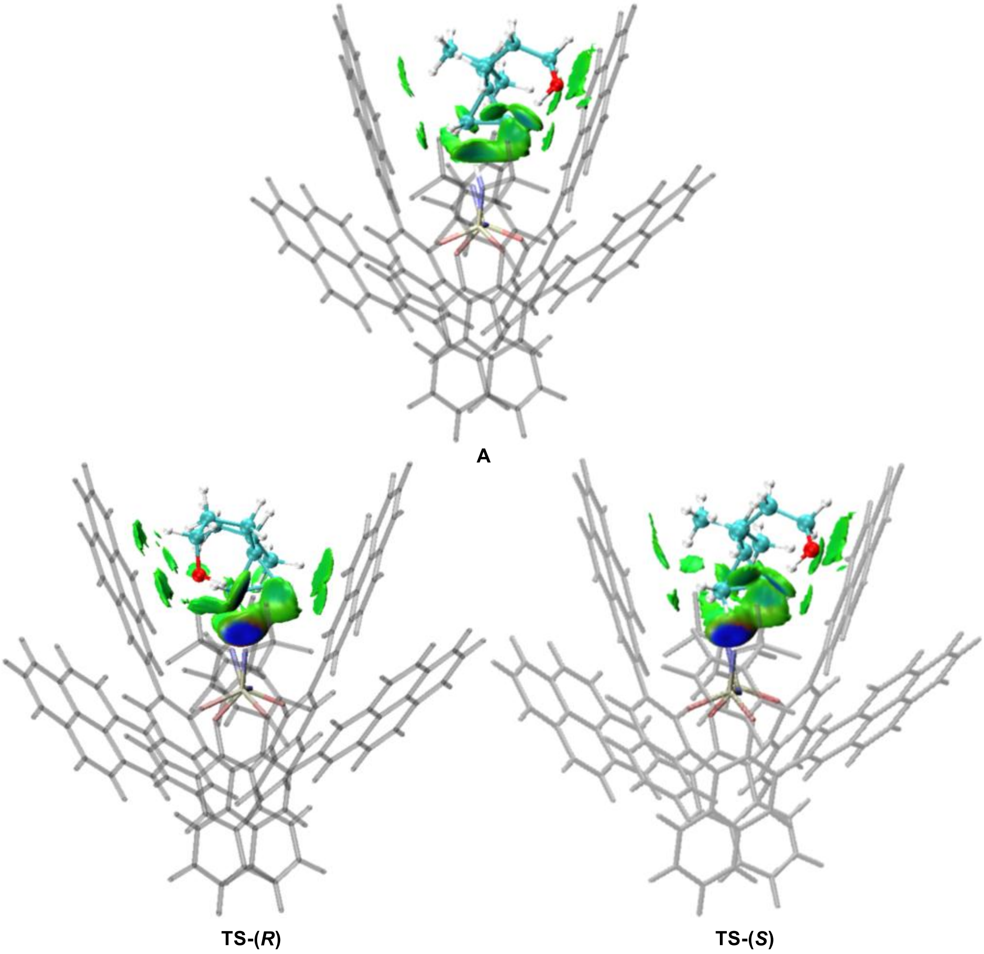

Figure S11. Independent gradient model based on Hirshfeld partition (IGMH).

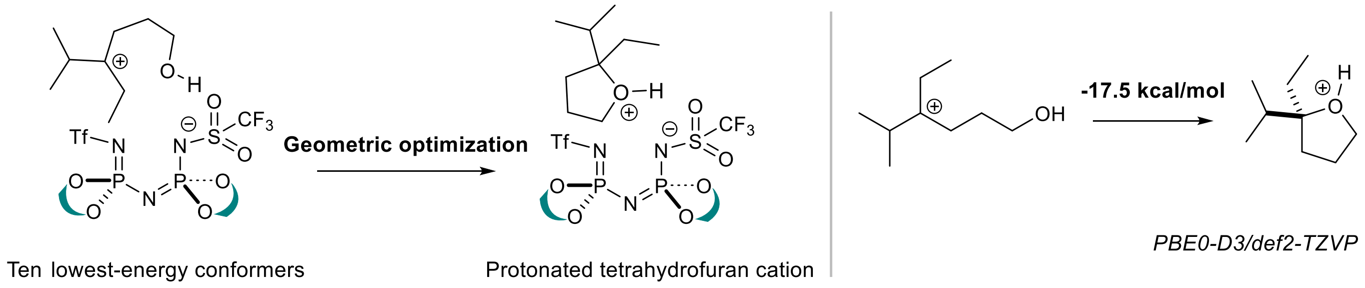

Figure S12. Geometric optimization of tertiary carbocation intermediate **INT-A** by DFT calculation.

Table S2: Electronic energies in toluene solvent (epsilon = 2.4, refrac = 1.497) (E_{ele}, at PBE0/def2-TZVP, 298.15K), relative Gibbs free energies in toluene solvent with translational entropy correction (G(PBE0/def2-TZVP) = E_{ele} (PBE0/def2-TZVP) + G_{corr}(PBE0/def2-SVP)) and frequency of transition state.

**Intermediate**	**E_{ele} (a. u.)**	**G (a. u.)**	**Imaginary frequency**
**IDPi 2f**	-7686.0804528	-7684.8052530	
**A**	-8114.2491730	-8112.7294653	
**TS-(*S*)**	-8114.2238643	-8112.7081764	-515.91 *i*
**TS-(*R*)**	-8114.2217866	-8112.7053330	-404.44 *i*
**1k**	-427.91471691	-428.12578232	
**3k-(*S*)**	-427.94623758	-428.16004033	
**3k-(*R*)**	-427.94550388	-428.15956599	

9. 3 List of atomic coordinates

IDPi 2f

P	-0.45250700	13.45977100	4.50517800
O	-0.78531500	13.59757600	2.90976000
O	-0.85271400	11.91670900	4.79918300
C	-2.00747300	13.07518700	2.47452300
C	-3.06374300	14.00613500	2.23793500
C	-4.26975400	13.48246500	1.78395500
C	-4.47018300	12.08331200	1.62330700
C	-5.73094200	11.56917600	1.19461700
C	-5.95650200	10.20358600	1.11866400
C	-4.92909200	9.29515600	1.48867800
C	-3.68569500	9.76133200	1.89242400
C	-3.40598500	11.15944000	1.94224500
C	-2.12525100	11.68629100	2.33907400
C	-0.95874200	10.81240900	2.67476600
C	-0.38317400	9.85751900	1.75841700
C	-0.86862900	9.66391500	0.43072900
C	-0.27188500	8.75226100	-0.42915100
C	0.83596600	7.97471800	0.00015800
C	1.34251400	8.14536700	1.27937700
C	0.77103000	9.09507400	2.17898700
C	1.34832800	9.31460900	3.46170300
C	0.82308100	10.24838200	4.34813000
C	-0.33646300	10.96176200	3.91541000
C	-2.89836700	15.45080900	2.55821600
H	-5.10375800	14.16581500	1.56376800
H	-6.52842300	12.28470900	0.94118100
H	-6.93524100	9.82112200	0.79200500
H	-5.12194800	8.21202000	1.46236700
H	-2.90556100	9.04738200	2.18977200
H	-1.73114800	10.24801500	0.08447300
H	-0.66462400	8.63043300	-1.44975200
H	1.29202900	7.24341200	-0.68398700
H	2.20739200	7.55762400	1.62279100
H	2.24189700	8.73889100	3.74633600
C	1.45406500	10.59599700	5.64920100
C	-1.75902800	16.17680000	2.15308500
C	-1.58354600	17.52836600	2.51216400
C	-2.58125100	18.17953800	3.30986600
C	-3.74075300	17.44606600	3.72884700
C	-3.88090900	16.09605000	3.33922200
C	2.84014700	10.84408100	5.71091700
C	3.44621900	11.31754800	6.89489100
C	2.63523000	11.54070500	8.05816000
C	1.22813500	11.26532600	8.00452300
C	0.66669700	10.78700400	6.80499500
H	-0.98282900	15.68733800	1.55309700
H	-4.74502400	15.52145500	3.70238600
H	3.45753200	10.72710000	4.80804300
H	-0.41444300	10.59778700	6.77520400
N	1.05142300	13.82262300	4.73017600
P	2.10437800	15.00256600	4.49903800
O	2.74807200	15.02983900	2.97670100
O	3.36898000	14.47757500	5.40623100
C	3.26716800	13.82048800	2.50119400

C	2.57600100	13.17234800	1.42571700
C	2.98237800	11.88209800	1.09537700
C	4.06397000	11.23969400	1.75906900
C	4.43010700	9.89660000	1.44701300
C	5.49377700	9.27983500	2.08845900
C	6.24527700	9.99342000	3.06020300
C	5.92924800	11.30978200	3.36812700
C	4.83300700	11.96934000	2.73702600
C	4.43726700	13.31207900	3.07527500
C	5.25470800	14.19380100	3.95582100
C	6.61168400	14.53877400	3.60533500
C	7.25944800	14.05860300	2.42732800
C	8.57109600	14.40887200	2.13759700
C	9.30182400	15.26256000	3.00594300
C	8.69058500	15.77837700	4.13813400
C	7.33890700	15.44979700	4.45699900
C	6.68361200	16.04624800	5.56993900
C	5.35801900	15.76534600	5.88144100
C	4.67708900	14.80787900	5.06855600
C	1.55037800	13.88504200	0.62135900
H	2.47502900	11.35033500	0.27620400
H	3.84105300	9.35839700	0.68952500
H	5.75897400	8.23971400	1.84573800
H	7.08538400	9.50088000	3.57255800
H	6.52264800	11.85623300	4.11442400
H	6.70739300	13.40812300	1.73622000
H	9.04567600	14.02600700	1.22137600
H	10.34362800	15.52608700	2.76894200
H	9.23342100	16.46463000	4.80657000
H	7.23653900	16.77049800	6.18758600
C	4.63457000	16.51564900	6.94106100
C	1.75912300	15.23046000	0.24874000
C	0.83295300	15.92338200	-0.55657800
C	-0.36788400	15.25704000	-0.97512200
C	-0.57349600	13.88125000	-0.61929200
C	0.40107600	13.21725500	0.15537500
C	4.57921600	17.92098500	6.84459300
C	3.83165700	18.68809600	7.76157000
C	3.11923100	18.01803400	8.81121500
C	3.22189600	16.59329800	8.94220300
C	3.97348700	15.86408800	7.99877000
H	2.66383100	15.75781500	0.58316700
H	0.23027600	12.17016700	0.44011700
H	5.08254000	18.42720200	6.00664100
H	4.01737400	14.76894000	8.08260600
N	-1.54456700	14.25610800	5.45202200
N	1.68836500	16.53431800	4.71872500
S	-2.88724600	13.62042000	6.31937100
O	-4.03193000	13.44141700	5.41432200
O	-2.44182300	12.49931900	7.16171700
S	0.63316000	16.99205600	5.84492500
O	-0.79525600	16.80830800	5.38539700
O	0.90534200	16.46722100	7.20219400
H	-1.37912800	15.31679400	5.48974000
C	-2.40451300	19.53912900	3.71209800
C	-3.37922100	20.17318800	4.55717300
C	-1.24855100	20.27318200	3.27651200

C	-3.18263100	21.51994700	4.94143800
C	-1.11385500	21.62689600	3.66017400
C	-2.06822200	22.23874900	4.48662200
H	-3.92371900	22.00116300	5.59822000
H	-0.23362500	22.19344700	3.31828900
H	-1.93057300	23.28673200	4.79291800
C	-4.70623100	18.10903300	4.56838000
H	-5.59133900	17.54509600	4.89576100
C	-4.52668400	19.41022400	4.97424300
H	-5.26584900	19.89267100	5.63211200
C	-0.25514200	19.58929900	2.48878100
H	0.65052800	20.14105600	2.19176600
C	-0.40901100	18.27046000	2.13578900
H	0.37099500	17.75294000	1.56103800
C	3.22092000	12.04393500	9.26061100
C	2.40269400	12.25708800	10.42388900
C	4.62560300	12.34466800	9.30510400
C	3.00796400	12.74476900	11.60472300
C	5.18059900	12.84656300	10.50484600
C	4.37950400	13.03715200	11.63962900
H	2.38283900	12.90927200	12.49602700
H	6.25466900	13.08621500	10.53643800
H	4.82940700	13.42786800	12.56486900
C	0.98990600	11.98892400	10.33371100
H	0.36486100	12.18136600	11.21959300
C	0.42443300	11.51808300	9.17362800
H	-0.65984800	11.34316100	9.10659600
C	5.41374200	12.12532700	8.11925100
H	6.48748900	12.36683900	8.15335400
C	4.85074000	11.62672300	6.96858700
H	5.46722500	11.46095400	6.07360000
C	2.26832100	18.76025100	9.68691700
C	1.52292200	18.08034300	10.70939700
C	2.13003500	20.18158400	9.52257400
C	0.64583700	18.82937500	11.52626800
C	1.23223500	20.88521500	10.35667600
C	0.49827900	20.21161100	11.34383600
H	0.06329300	18.30756300	12.30114100
H	1.11034100	21.97018700	10.21512400
H	-0.20124800	20.77374700	11.98114800
C	2.89950200	20.83395800	8.49455000
H	2.79844300	21.92486200	8.38004700
C	3.71517900	20.11934300	7.65058200
H	4.27393700	20.62952300	6.85016300
C	1.68104600	16.65554400	10.84486400
H	1.10994900	16.13812800	11.63134600
C	2.49292900	15.94296400	9.99721900
H	2.57962500	14.85237800	10.09600700
C	-1.36571800	15.96937600	-1.70862800
C	-1.15721400	17.34944600	-2.05147600
C	-2.58605400	15.31129900	-2.08668300
C	-2.16885400	18.03895400	-2.75738400
C	-3.56863900	16.04409500	-2.79030200
C	-3.35926600	17.39132600	-3.11842600
H	-2.01143500	19.09703600	-3.01800200
H	-4.50557600	15.54173000	-3.07695100
H	-4.13696800	17.94634000	-3.66470600

C	1.03225200	17.29657500	-0.94558200
H	1.96411200	17.79583000	-0.63773100
C	0.07727100	17.97961200	-1.65898400
H	0.23584700	19.03372500	-1.93453900
C	-1.79693000	13.24194700	-1.03137900
H	-1.95556500	12.19049800	-0.75371600
C	-2.76221500	13.92779600	-1.72740400
H	-3.69955800	13.42722200	-2.01586200
C	-3.11389100	15.07632300	7.35055900
C	-4.23513000	15.91839600	7.21897000
C	-2.08883300	15.41323500	8.26136300
C	-4.30156300	17.11789500	7.94532900
C	-2.16053800	16.60635700	8.99077800
C	-3.26188000	17.46258900	8.82329000
C	0.83902100	18.78569100	5.86778700
C	-0.00103000	19.47616000	6.76546300
C	1.71026800	19.53675700	5.05618000
C	0.00891200	20.87237900	6.85174800
C	1.72213700	20.93838100	5.14019900
C	0.87360500	21.60586600	6.02896100
F	-3.32071800	18.60016600	9.50661300
F	-1.19237400	16.93970500	9.83911700
F	-1.02608600	14.63232100	8.42014700
F	-5.25359400	15.62439400	6.41268500
F	-5.35517500	17.92627700	7.81295800
F	-0.83650100	18.81211500	7.56983500
F	2.55247100	18.99093700	4.18160900
F	-0.80119600	21.50456600	7.70283900
F	0.87985700	22.93779600	6.08820300
F	2.53113000	21.64297900	4.34025800

A

O	1.28594100	2.92132700	-2.09159700
O	-0.07113400	1.87981900	-0.21410800
O	2.03672100	-1.52250300	0.11448700
O	3.02341800	-1.27606700	-2.21916300
S	-0.01235900	-3.66568800	-1.03456800
S	-0.85687600	2.86535800	-4.18043900
O	-0.18069100	-3.38568700	0.41321400
O	-1.18263500	-4.20951400	-1.75534800
O	0.42329600	2.84100200	-4.89598200
O	-2.11013900	2.52926600	-4.88534900
N	0.55698900	-2.40334900	-1.88517900
P	1.57826300	-1.26564300	-1.44402100
N	1.11360200	0.21561000	-1.78517100
P	0.42019200	1.58599200	-1.73900900
C	4.01380600	2.35007500	-2.69730500
C	3.50871100	2.78071800	-3.94104400
C	3.86879600	2.13528200	-5.14083400
C	4.75706900	1.00865900	-5.09104600
C	5.29887600	0.58786600	-3.82904700
C	4.92232100	1.27346200	-2.65458100
H	2.81456300	3.62796500	-3.98894500
H	5.30874200	0.92259500	-1.68666000
C	-2.94121300	2.13469400	0.13424700
C	-2.59254600	0.76641500	0.18393200
C	-3.50599500	-0.24488800	-0.17510400

C	-4.83287200	0.11271000	-0.57870600
C	-5.21241900	1.49650300	-0.59605600
C	-4.26218700	2.47439600	-0.24098700
H	-1.59634700	0.45837200	0.51435500
H	-4.55233200	3.53120500	-0.32706300
C	3.29987200	-3.22185100	-4.31713800
C	2.85693100	-2.00338100	-4.87916800
C	2.07310600	-1.96758100	-6.04867600
C	1.73092300	-3.19500600	-6.70468100
C	2.18572400	-4.43801500	-6.15157300
C	2.96645000	-4.42421100	-4.97828300
H	3.11978500	-1.05062900	-4.41030900
H	3.28611700	-5.38841600	-4.56117500
C	1.55250700	0.26688200	2.30041100
C	1.21471300	1.55048700	2.78255900
C	-0.07869800	1.84756600	3.25884200
C	-1.07807300	0.81852000	3.25267700
C	-0.71756500	-0.51055900	2.84955800
C	0.58835400	-0.76330700	2.38003400
H	1.95476500	2.36168500	2.74193600
H	0.82722700	-1.78045800	2.04562000
C	-0.57164800	3.13474700	0.13556700
C	2.24471700	3.32955300	-1.14829300
C	3.14556900	-0.89881600	0.67141300
C	3.90922200	-2.32070300	-2.00422000
C	-1.97565200	3.23428200	0.40857600
C	4.08812400	-3.27699800	-3.05662400
C	3.61171400	3.02849900	-1.43788100
C	2.91147100	0.02506800	1.74484700
C	0.06537600	7.90996400	0.89863600
C	0.59545900	6.68864400	0.50626300
C	-0.18597900	5.49726200	0.57134500
C	-1.56728900	5.60307400	0.97178800
C	-2.07370900	6.86983900	1.39341200
C	-1.27032800	7.99870000	1.37450300
H	0.68309700	8.81803100	0.83232500
H	1.62713800	6.63290100	0.13216500
C	0.33631900	4.19806800	0.23343300
C	-2.41582900	4.46809300	0.88934600
H	-3.12550500	6.93278600	1.71189500
H	-3.46559300	4.58146000	1.19507600
C	4.81776400	4.96576900	2.90524800
C	5.16930100	4.37564800	1.70186300
C	4.17859000	4.03188700	0.73365300
C	2.79118400	4.33226500	0.99741000
C	2.46803700	4.93237900	2.25085700
C	3.45167900	5.23584900	3.18203700
H	5.61517400	3.17764400	-0.65962800
H	6.22077600	4.14827500	1.47166200
C	4.55079700	3.38863300	-0.47711900
C	1.79721600	3.97019700	0.01048500
H	1.42043500	5.15556000	2.48318000
H	3.16670400	5.69198000	4.14208500
C	7.91654300	0.14530700	0.64521800
C	6.86331100	-0.61738000	0.15923000
C	5.54671000	-0.48242600	0.69319900
C	5.33300500	0.48536900	1.74065500

C	6.44314400	1.22900300	2.24268500
C	7.71131100	1.06729700	1.70627700
H	8.91861100	0.03157300	0.20496800
H	7.03899200	-1.33052600	-0.65740200
C	4.41985700	-1.23551300	0.19811500
C	4.02576500	0.68516400	2.25907500
H	6.26326000	1.94648600	3.05720000
H	3.89899700	1.38628200	3.09728100
C	7.26506400	-5.63105000	-0.13889100
C	6.62883600	-5.48175100	-1.36126800
C	5.72499700	-4.40099700	-1.59004600
C	5.49686500	-3.44086800	-0.53673500
C	6.13943400	-3.64509100	0.71916900
C	7.00538200	-4.71245800	0.91198700
H	5.23996700	-5.01598000	-3.61374600
H	6.79486400	-6.20697400	-2.17247800
H	5.93320800	-2.95128900	1.54534100
H	7.48468200	-4.85444600	1.89232000
C	5.02682100	-4.28048400	-2.82350800
C	4.60539000	-2.33681400	-0.79327400
H	-1.67452300	8.97125800	1.69259900
H	5.59019000	5.22106600	3.64622400
H	8.55717000	1.65437600	2.09476800
H	7.95438600	-6.47268100	0.02634200
C	-2.83174400	-1.41189400	-4.90375600
C	-3.91014800	-0.46763700	-5.44581900
C	-4.05665000	-0.42821700	-6.97640400
H	-3.69552500	0.55520400	-5.06892000
H	-4.89174200	-0.73953300	-4.99981200
H	-4.44283900	-1.40473300	-7.34159700
H	-4.82521300	0.32753700	-7.24875500
C	-2.31247600	-1.03015800	-3.52933800
H	-2.83843500	-0.19448900	-3.03227900
H	-1.99238300	-1.82340200	-2.83738600
C	-3.01861500	-2.90095200	-5.25330600
C	-4.14600100	-3.51404100	-4.40899500
H	-4.36541300	-4.55320200	-4.72931200
H	-3.85321400	-3.54286000	-3.34005200
H	-5.08793300	-2.93320500	-4.47999800
C	-1.74218900	-3.74440300	-5.15312400
H	-1.96609200	-4.80190600	-5.40245600
H	-0.96234500	-3.40017100	-5.85956100
H	-1.31857000	-3.73874500	-4.12921900
C	-1.07562200	4.44951300	-3.37251000
C	-0.08631100	5.45449300	-3.38598800
C	-2.31511700	4.70988300	-2.75053700
C	-0.33626600	6.69445600	-2.77661500
C	-2.56421200	5.94690100	-2.14277800
C	-1.58405000	6.95156600	-2.18732300
C	1.30291500	-4.92180400	-1.17619400
C	2.27154500	-5.09708900	-0.16738600
C	1.37862300	-5.73240100	-2.32721400
C	3.25881700	-6.08917000	-0.28117900
C	2.37184200	-6.71612900	-2.44901000
C	3.31996100	-6.88916100	-1.43067900
F	1.10897700	5.27760100	-3.94111800
F	0.60715100	7.63511600	-2.76377600

F	-1.82443300	8.13778700	-1.64372200
F	-3.72549500	6.17261200	-1.52732700
F	-3.26538300	3.78160800	-2.70644700
F	2.30719100	-4.33946100	0.92967200
F	4.16188600	-6.24834600	0.68808400
F	4.28788100	-7.79554600	-1.56441100
F	2.45055800	-7.46148300	-3.55981000
F	0.53165100	-5.59522400	-3.34675000
C	0.92745900	-3.17905600	-7.88491200
C	0.46252300	-1.92815200	-8.41897700
C	0.56880500	-4.41573200	-8.52305900
C	-0.32499000	-1.94089700	-9.59293900
C	-0.22599200	-4.37553400	-9.69098500
C	-0.66057500	-3.15041000	-10.21849500
H	-0.67970200	-0.98457000	-10.00635600
H	-0.50271300	-5.32146100	-10.18199100
H	-1.27768000	-3.13842000	-11.12992600
C	1.02380700	-5.65131900	-7.93679000
H	0.73054200	-6.59630700	-8.42022500
C	1.79584000	-5.66412900	-6.79949500
H	2.12000300	-6.61617400	-6.35186100
C	0.79536800	-0.71110600	-7.72234800
H	0.37856100	0.23855400	-8.09196600
C	1.56978000	-0.73021000	-6.58773000
H	1.79611700	0.20579600	-6.05625100
C	5.06608100	0.28630500	-6.28489700
C	5.90579200	-0.87796400	-6.22243600
C	4.50350800	0.70071500	-7.54025700
C	6.14820000	-1.61058000	-7.40625000
C	4.77768800	-0.06283200	-8.69686800
C	5.58528500	-1.20706000	-8.62555700
H	6.78140000	-2.50990500	-7.35704100
H	4.33630600	0.24614600	-9.65692500
H	5.77788600	-1.79511400	-9.53571800
C	3.65497700	1.86457000	-7.56715000
H	3.23161400	2.17866500	-8.53379800
C	3.34642400	2.55128300	-6.41795400
H	2.66593500	3.41508600	-6.44490700
C	6.17047100	-0.55837500	-3.80383800
H	6.58710200	-0.87636500	-2.83713300
C	6.45468000	-1.26353800	-4.94857700
H	7.09952300	-2.15479200	-4.90474300
C	-5.75862700	-0.89779300	-0.98089500
C	-5.35965700	-2.27871900	-0.97375500
C	-7.08130100	-0.52964300	-1.40490900
C	-6.28998900	-3.25762900	-1.39080700
C	-7.97522300	-1.54570500	-1.81245800
C	-7.57909200	-2.89169200	-1.80453100
H	-5.98395500	-4.31504800	-1.39548600
H	-8.98892100	-1.26705100	-2.13993100
H	-8.28737600	-3.66923400	-2.12911600
C	-2.42902000	1.12314100	3.60133500
C	-2.79284400	2.46522100	3.96222100
C	-3.43039600	0.09357000	3.55231300
C	-4.14968800	2.75777600	4.22769700
C	-4.77475600	0.43637400	3.82201700
C	-5.12697600	1.75452100	4.14684700

H	-4.43132000	3.78771600	4.49738400
H	-5.54639000	-0.34703400	3.76762700
H	-6.18061700	2.00261700	4.34638000
C	-4.01893500	-2.60877700	-0.56399300
H	-3.69492600	-3.66012000	-0.59215500
C	-3.13071900	-1.63448900	-0.17932700
H	-2.10607500	-1.90562500	0.10981100
C	-6.54823900	1.83697800	-1.01655400
H	-6.83839000	2.89922700	-1.02871200
C	-7.44123400	0.86617500	-1.40360200
H	-8.45683100	1.14228200	-1.72787500
C	-1.72612400	-1.53920400	2.87876800
H	-1.43785500	-2.55643100	2.57481300
C	-3.02700600	-1.24574700	3.21017900
H	-3.79625700	-2.03291700	3.18779200
C	-1.75613700	3.46297600	4.03115700
H	-2.03171600	4.48389800	4.33832800
C	-0.45552300	3.16468600	3.70559300
H	0.32107400	3.94016700	3.75897000
C	-1.44916500	-0.81489400	-4.75839200
H	-1.27720100	0.19224500	-5.16484500
H	-0.57196500	-1.47016300	-4.84918900
H	-3.33683800	-2.93182100	-6.31960100
C	-2.76624900	-0.12856100	-7.73865200
H	-2.00076000	-0.89441200	-7.47023600
H	-2.94933100	-0.23984400	-8.82867700
O	-2.26053100	1.18966800	-7.56321800
H	-2.23087700	1.40156400	-6.60819400
N	-0.84228200	1.79924300	-2.82722100
H	-1.38752800	0.93273600	-3.03286000

TS-(*S*)

O	1.28132200	2.93882000	-2.12105300
O	-0.11185600	1.89500100	-0.27498400
O	2.03663800	-1.56466900	0.10713000
O	3.01765400	-1.31886600	-2.22626800
S	-0.06015400	-3.60066900	-1.05167600
S	-0.87050800	2.70578600	-4.17597700
O	-0.22663300	-3.35281400	0.40196200
O	-1.23523600	-4.13053800	-1.77854100
O	0.38178900	2.73628900	-4.95554100
O	-2.12869200	2.39140400	-4.90910600
N	0.49035600	-2.31766900	-1.88399100
P	1.57743200	-1.22852600	-1.43907900
N	1.21455900	0.27720700	-1.73127000
P	0.39466600	1.59387500	-1.80664400
C	4.00424900	2.34573200	-2.69530200
C	3.50429400	2.77626200	-3.94141200
C	3.87180000	2.13259900	-5.13972100
C	4.76045800	1.00651800	-5.08707500
C	5.29343800	0.58297500	-3.82235100
C	4.91109100	1.26787200	-2.64942400
H	2.80529700	3.61933300	-3.99183400
H	5.29053800	0.91436900	-1.67958900
C	-2.97592600	2.14633800	0.11713000
C	-2.62220200	0.77962500	0.16406100
C	-3.54362800	-0.23601200	-0.15898600

C	-4.88457700	0.11359700	-0.52055400
C	-5.26734000	1.49628800	-0.53866100
C	-4.30817700	2.47880700	-0.22236200
H	-1.61477000	0.47823900	0.46533600
H	-4.60324400	3.53411600	-0.30885500
C	3.30192500	-3.23908000	-4.33618900
C	2.86534200	-2.01210400	-4.88577600
C	2.09821200	-1.95875400	-6.06587800
C	1.76841500	-3.17525200	-6.74809000
C	2.21619100	-4.42634100	-6.20786400
C	2.97741400	-4.43060000	-5.02136700
H	3.12344100	-1.06634200	-4.40040000
H	3.29217300	-5.40133300	-4.61579300
C	1.55179600	0.24085300	2.28388400
C	1.21651200	1.53062900	2.75138100
C	-0.07757400	1.83598800	3.22150500
C	-1.08058000	0.81055500	3.22180500
C	-0.72350200	-0.52315900	2.83173900
C	0.58321900	-0.78467300	2.36928700
H	1.95860200	2.33972000	2.70435100
H	0.81990100	-1.80525200	2.04428300
C	-0.60523400	3.14351800	0.08396200
C	2.22378600	3.33154400	-1.16769100
C	3.14435500	-0.94738500	0.67148100
C	3.89412700	-2.36466500	-2.00834300
C	-2.00725200	3.24818300	0.37125200
C	4.07880500	-3.31235400	-3.06873000
C	3.59416600	3.02228400	-1.43765100
C	2.91231000	-0.01451300	1.73751200
C	0.03699800	7.92052500	0.84444600
C	0.56575100	6.69651500	0.45838100
C	-0.21644200	5.50608300	0.52945100
C	-1.59639700	5.61569400	0.93267500
C	-2.10100000	6.88435500	1.34991200
C	-1.29759500	8.01316000	1.32226000
H	0.65532000	8.82779600	0.77182300
H	1.59683800	6.63741100	0.08298200
C	0.30470800	4.20591900	0.19313500
C	-2.44450200	4.47997400	0.85848300
H	-3.15239800	6.94941700	1.66960300
H	-3.49128400	4.59297800	1.17431900
C	4.76094200	4.94600100	2.92150300
C	5.12151600	4.34862400	1.72419700
C	4.14095600	4.01628200	0.74181700
C	2.75282500	4.33189200	0.98525600
C	2.42121800	4.94223400	2.23150900
C	3.39487900	5.23637700	3.17633800
H	5.58919100	3.15131600	-0.63269100
H	6.17338800	4.10810500	1.50900600
C	4.52401500	3.37044000	-0.46448600
C	1.76810600	3.97482000	-0.01325600
H	1.37375300	5.18169900	2.44696700
H	3.10165800	5.70097800	4.12989000
C	7.91951800	0.07575900	0.64161200
C	6.86350900	-0.68558200	0.15937900
C	5.54710900	-0.54220600	0.69143100
C	5.33623200	0.43287600	1.73311500

C	6.44900300	1.17562900	2.23067500
C	7.71695300	1.00572200	1.69614500
H	8.92144900	-0.04452800	0.20273900
H	7.03681200	-1.40373200	-0.65339100
C	4.41646700	-1.29099900	0.19859700
C	4.02919500	0.64315800	2.24886000
H	6.27097800	1.90078000	3.03886700
H	3.90493700	1.35309200	3.08001200
C	7.23011000	-5.70444100	-0.15531900
C	6.59901100	-5.54388400	-1.37902100
C	5.70366400	-4.45559300	-1.60472800
C	5.47737500	-3.50013700	-0.54679800
C	6.11569500	-3.71496400	0.70955500
C	6.97379200	-4.78919500	0.89934600
H	5.22893000	-5.04968400	-3.63689000
H	6.76341700	-6.26520900	-2.19407100
H	5.91221300	-3.02339300	1.53831600
H	7.44981600	-4.93903200	1.88012700
C	5.01243500	-4.32145500	-2.84079600
C	4.59176900	-2.39025500	-0.79733200
H	-1.70067000	8.98778900	1.63558400
H	5.52589000	5.19241500	3.67323200
H	8.56467000	1.59233300	2.08127700
H	7.91286500	-6.55183900	0.00773500
C	-2.68338500	-1.28959600	-4.92133500
C	-3.82323100	-0.41651700	-5.45167400
C	-3.96389900	-0.43609600	-6.98447300
H	-3.66161400	0.62610400	-5.11159700
H	-4.77707800	-0.74771700	-4.99118800
H	-4.28526100	-1.44475800	-7.32343300
H	-4.78060600	0.26215000	-7.26639100
C	-2.13325600	-0.74063200	-3.46292800
H	-3.00463400	-0.14203400	-3.14302200
H	-1.81568400	-1.53990800	-2.77714800
C	-2.88087700	-2.80570400	-5.04676100
C	-3.87654100	-3.34451100	-4.01004200
H	-4.18202200	-4.37795500	-4.26851800
H	-3.40869100	-3.37347000	-3.00707300
H	-4.79442800	-2.72710000	-3.94285300
C	-1.57953800	-3.61076800	-5.05592400
H	-1.80429200	-4.68959400	-5.17072100
H	-0.91549200	-3.31568100	-5.89152000
H	-1.02667600	-3.50577600	-4.10299300
C	-1.12753100	4.32624300	-3.42008700
C	-0.15636300	5.34636200	-3.44619100
C	-2.36636200	4.58024100	-2.79847500
C	-0.41818700	6.58893100	-2.84603500
C	-2.63128600	5.81921700	-2.20050000
C	-1.66416800	6.83515000	-2.25046000
C	1.24783000	-4.86571700	-1.21026500
C	2.20774500	-5.07138500	-0.19801900
C	1.31420300	-5.66911600	-2.36696700
C	3.18324300	-6.07425100	-0.32235900
C	2.29138900	-6.66721600	-2.49682200
C	3.23538100	-6.86393400	-1.47913400
F	1.03975000	5.18767100	-4.00991400
F	0.51396800	7.54356300	-2.84722100

F	-1.91634900	8.02183600	-1.70776400
F	-3.79641000	6.03669300	-1.58403100
F	-3.30902500	3.64105200	-2.74028300
F	2.24788300	-4.33567700	0.91275100
F	4.08125200	-6.25643400	0.64721700
F	4.19078800	-7.78200600	-1.62074600
F	2.35768800	-7.40598300	-3.61304400
F	0.47156900	-5.51036500	-3.38828100
C	0.98662800	-3.14048500	-7.94262400
C	0.53230500	-1.88099600	-8.46635600
C	0.64344600	-4.36665100	-8.60911300
C	-0.23116200	-1.87462600	-9.65621800
C	-0.12609500	-4.30738700	-9.79325700
C	-0.55167200	-3.07404300	-10.30890200
H	-0.57667700	-0.91162200	-10.06223400
H	-0.38847000	-5.24491600	-10.30774600
H	-1.14795700	-3.04734100	-11.23377400
C	1.09063900	-5.61116300	-8.03564100
H	0.81067100	-6.54831400	-8.54167200
C	1.84122500	-5.64177300	-6.88423200
H	2.16167200	-6.60092100	-6.44916800
C	0.85358200	-0.67518000	-7.74473100
H	0.45517700	0.28311300	-8.11297700
C	1.60518500	-0.71251700	-6.59476300
H	1.82290800	0.21602200	-6.04676200
C	5.07735200	0.28669200	-6.28049500
C	5.91500900	-0.87910500	-6.21477700
C	4.52390400	0.70498800	-7.53884200
C	6.16510500	-1.60897900	-7.39881100
C	4.80652300	-0.05553800	-8.69553500
C	5.61229400	-1.20097300	-8.62132500
H	6.79726900	-2.50896500	-7.34742200
H	4.37299200	0.25675500	-9.65816800
H	5.81142600	-1.78648400	-9.53179100
C	3.67401100	1.86806100	-7.56804700
H	3.25647900	2.18409800	-8.53666400
C	3.35544000	2.55016100	-6.41877900
H	2.67075300	3.41058300	-6.44606900
C	6.16181800	-0.56557300	-3.79336200
H	6.57048900	-0.88604200	-2.82405100
C	6.45317900	-1.26902000	-4.93747800
H	7.09533600	-2.16213400	-4.89050900
C	-5.82406000	-0.90412900	-0.87082000
C	-5.42515100	-2.28527000	-0.85106400
C	-7.16322700	-0.54355800	-1.24740900
C	-6.37040000	-3.27169800	-1.21356000
C	-8.07228600	-1.56729400	-1.59948100
C	-7.67530100	-2.91295700	-1.58287000
H	-6.06545200	-4.32959200	-1.20637100
H	-9.09935100	-1.29468600	-1.88842000
H	-8.39628000	-3.69626700	-1.86274500
C	-2.43131500	1.12263700	3.56521700
C	-2.79146800	2.46892700	3.91345000
C	-3.43625900	0.09622300	3.52312200
C	-4.14841700	2.76915100	4.16973300
C	-4.78065900	0.44664300	3.78258500
C	-5.12946200	1.76930900	4.09244100

H	-4.42707600	3.80266000	4.42861400
H	-5.55503400	-0.33438400	3.73211200
H	-6.18319500	2.02357000	4.28364800
C	-4.07078300	-2.60741400	-0.48102300
H	-3.75183700	-3.66061700	-0.48690300
C	-3.16817300	-1.62566700	-0.15219700
H	-2.13600600	-1.89116000	0.11427800
C	-6.61848400	1.82904600	-0.91381900
H	-6.91107300	2.89060800	-0.92784500
C	-7.52431400	0.85214700	-1.25335200
H	-8.55234900	1.12278100	-1.54100400
C	-1.73526500	-1.54829800	2.87079600
H	-1.44954700	-2.56934800	2.57784400
C	-3.03587100	-1.24786100	3.19700900
H	-3.80733100	-2.03308000	3.18197300
C	-1.75103900	3.46289600	3.97888600
H	-2.02323200	4.48691800	4.27850500
C	-0.45078500	3.15719400	3.65900900
H	0.32854000	3.92960200	3.71017600
C	-1.36925000	-0.69696300	-4.86634100
H	-1.18692300	0.29878900	-5.28876700
H	-0.48073100	-1.34035200	-4.79641800
H	-3.34734200	-2.91641500	-6.05167000
C	-2.69544100	-0.06809900	-7.75543200
H	-1.88736900	-0.79390700	-7.49271500
H	-2.88311400	-0.21062800	-8.84089800
O	-2.25885600	1.27255700	-7.60402600
H	-2.25002700	1.53184600	-6.65682400
N	-0.82365200	1.64789800	-2.90201500
H	-1.44960900	0.30936100	-3.22595300

TS-(*R*)

O	1.29676100	2.96042100	-2.15295000
O	-0.08085700	1.87872600	-0.31737400
O	1.92161700	-1.44170900	0.00765100
O	2.94397300	-1.28599800	-2.32680400
S	-0.08788300	-3.60770700	-1.11030000
S	-0.99995100	2.99263300	-4.09536500
O	-0.26790800	-3.32185500	0.33487600
O	-1.24492400	-4.18299400	-1.82932600
O	0.17919100	3.01041600	-4.98332300
O	-2.32667100	2.79038500	-4.74862200
N	0.44594000	-2.33804300	-1.97109400
P	1.48878100	-1.19399700	-1.56231300
N	1.05528700	0.26940200	-1.96825700
P	0.34288700	1.64983700	-1.88794600
C	3.99666800	2.30192500	-2.76253700
C	3.46523700	2.71757900	-4.00116600
C	3.82065200	2.07198400	-5.20237400
C	4.72706700	0.95939400	-5.16178500
C	5.27940100	0.54173900	-3.90364000
C	4.91067000	1.22957300	-2.72841400
H	2.75621600	3.55311000	-4.04452600
H	5.31057800	0.88656500	-1.76323100
C	-2.95351200	2.16312700	0.17800500
C	-2.61927600	0.79310900	0.09009800
C	-3.58139500	-0.18749900	-0.21928700

C	-4.94534600	0.19527400	-0.41707500
C	-5.30909300	1.57487800	-0.28232700
C	-4.30978600	2.52502600	0.00084400
H	-1.59510700	0.45616500	0.27225800
H	-4.60199400	3.58441700	0.01573700
C	3.32273700	-3.28743500	-4.37494600
C	2.87267000	-2.09849100	-4.99103000
C	2.16113500	-2.12047000	-6.20660400
C	1.89354900	-3.37380800	-6.84853900
C	2.35704100	-4.58616300	-6.23941600
C	3.06522100	-4.51581300	-5.02359700
H	3.07370200	-1.12576900	-4.53127400
H	3.39029600	-5.45877500	-4.56462100
C	1.42389100	0.29775300	2.23509600
C	1.09790600	1.56635100	2.76153900
C	-0.19331300	1.86050100	3.24441700
C	-1.20439800	0.84390800	3.19653100
C	-0.85846300	-0.47211400	2.74184300
C	0.44742400	-0.72315400	2.27130600
H	1.84743400	2.36942800	2.75142300
H	0.67663700	-1.73039700	1.90260200
C	-0.55540700	3.11894400	0.10030400
C	2.25492800	3.29873800	-1.19730700
C	3.02491500	-0.83692700	0.59078300
C	3.83115300	-2.31376100	-2.05791500
C	-1.95019200	3.23728200	0.42157000
C	4.05878500	-3.29199800	-3.08237200
C	3.61706100	2.98212900	-1.49716200
C	2.78563300	0.06152000	1.68464600
C	0.19733400	7.85733800	1.00831700
C	0.69278600	6.63885100	0.56607300
C	-0.11145900	5.46137700	0.61240900
C	-1.47912200	5.58184300	1.04783200
C	-1.95059200	6.84440400	1.52020100
C	-1.12584100	7.95768100	1.51688400
H	0.83119800	8.75504000	0.95379500
H	1.71356100	6.57496500	0.16449300
C	0.37431200	4.16313200	0.22337200
C	-2.34794600	4.46455400	0.95612200
H	-2.99346400	6.91730900	1.86496100
H	-3.38157900	4.58928800	1.30707100
C	4.88908300	4.86613000	2.85062800
C	5.22339600	4.30025300	1.63067700
C	4.21820300	3.96038500	0.67617900
C	2.83402200	4.24850100	0.96723800
C	2.52784700	4.81416900	2.24119300
C	3.52522200	5.11018200	3.16023700
H	5.63254700	3.11237700	-0.74283500
H	6.27256000	4.08659100	1.37814500
C	4.57140100	3.32713500	-0.54586000
C	1.82802200	3.91659900	-0.01800900
H	1.48209000	5.01785300	2.49879800
H	3.25283700	5.54064400	4.13573600
C	7.80780700	0.15316100	0.66003700
C	6.75246400	-0.58268000	0.13858600
C	5.43057900	-0.44638900	0.65907100
C	5.21270400	0.49551300	1.72785300

C	6.32481600	1.20841100	2.26796000
C	7.59867700	1.04521000	1.74564100
H	8.81443000	0.04063900	0.22990400
H	6.93003000	-1.27537900	-0.69517900
C	4.30389600	-1.18118700	0.13728500
C	3.89998000	0.69485100	2.23232200
H	6.14253400	1.90488800	3.09965300
H	3.76855300	1.37295200	3.08853100
C	7.17085200	-5.55555300	-0.02817600
C	6.57443700	-5.43401600	-1.27333200
C	5.66713600	-4.36728400	-1.54921800
C	5.39394500	-3.39024400	-0.52450600
C	5.99422800	-3.56809000	0.75649400
C	6.86369200	-4.62284600	0.99711700
H	5.25802800	-5.01720000	-3.57649000
H	6.77353600	-6.17121500	-2.06619200
H	5.75148400	-2.86513600	1.56443700
H	7.30834500	-4.74378300	1.99642600
C	5.00682500	-4.27516300	-2.80416700
C	4.50369000	-2.29653800	-0.83264400
H	-1.50331400	8.92780100	1.87313000
H	5.67313500	5.11826600	3.58048500
H	8.44609200	1.60915400	2.16393200
H	7.86269600	-6.38662100	0.17548800
C	-2.65858700	-1.17453000	-5.07635700
C	-3.94842000	-0.51249700	-5.58417300
C	-5.15472300	-0.44012400	-4.61725000
H	-4.22888800	-1.07320400	-6.49920100
H	-3.70155300	0.51320600	-5.93001100
H	-6.08052800	-0.76945200	-5.13401800
H	-5.01984500	-1.13624200	-3.76471900
C	-2.09756100	-0.56954100	-3.66348400
H	-2.99982600	-0.01132600	-3.29700600
H	-1.65960300	-1.34608600	-3.01711700
C	-2.59778600	-2.70902900	-5.20171500
C	-3.58481300	-3.42968100	-4.27284200
H	-3.51982100	-4.52384800	-4.43513500
H	-3.33370700	-3.23799000	-3.21167700
H	-4.63480100	-3.12632100	-4.44702800
C	-1.18951100	-3.29366600	-5.07996800
H	-1.23235800	-4.39636600	-5.16850000
H	-0.52151500	-2.92644400	-5.87996700
H	-0.72506200	-3.07501600	-4.09882200
C	-1.09722400	4.58962200	-3.24662200
C	-0.05986300	5.54216400	-3.29769500
C	-2.28600200	4.92123200	-2.56585800
C	-0.21990400	6.79980000	-2.69225600
C	-2.45492000	6.17929100	-1.97264300
C	-1.42797100	7.13123400	-2.06095200
C	1.24392300	-4.85182300	-1.22521000
C	2.19271500	-5.02091900	-0.19647100
C	1.33412100	-5.68173300	-2.36102400
C	3.17222600	-6.02430500	-0.27681500
C	2.31959100	-6.67609000	-2.45052700
C	3.24789800	-6.84159700	-1.41302300
F	1.10558600	5.30722300	-3.89829700
F	0.77628100	7.68737200	-2.72187000

F	-1.58503500	8.33587300	-1.52072700
F	-3.58416500	6.46709200	-1.32099200
F	-3.28670800	4.04819600	-2.46067700
F	2.21725600	-4.24737100	0.88937600
F	4.05518800	-6.17784900	0.71138400
F	4.21217700	-7.75575900	-1.51749600
F	2.40955700	-7.44162100	-3.54706900
F	0.50673600	-5.55206000	-3.39849300
C	1.14173200	-3.41661700	-8.06155100
C	0.65284100	-2.19727600	-8.64582900
C	0.85501700	-4.68028700	-8.68276300
C	-0.09568300	-2.26712700	-9.84302100
C	0.10467300	-4.69717400	-9.88046900
C	-0.36102200	-3.50303400	-10.45077600
H	-0.47150100	-1.33484900	-10.29253800
H	-0.11450100	-5.66364800	-10.36037600
H	-0.94513700	-3.53692800	-11.38313100
C	1.33165400	-5.88390000	-8.04892300
H	1.09899800	-6.85002400	-8.52336700
C	2.04699500	-5.84079300	-6.87593100
H	2.38615100	-6.76940000	-6.39174800
C	0.93636200	-0.95171200	-7.97962900
H	0.55648600	-0.01930400	-8.42589500
C	1.66048900	-0.91342800	-6.81178100
H	1.85829500	0.04658100	-6.31255700
C	5.04790700	0.25064800	-6.36073500
C	5.90140500	-0.90422600	-6.30538200
C	4.48493300	0.66982300	-7.61459200
C	6.15931300	-1.62126400	-7.49551700
C	4.77807500	-0.07618500	-8.77805800
C	5.60021700	-1.21045800	-8.71425200
H	6.80239100	-2.51387000	-7.45194100
H	4.33887500	0.23746400	-9.73768400
H	5.80688700	-1.78544000	-9.62971300
C	3.61041100	1.81503300	-7.63117800
H	3.18319000	2.13008300	-8.59600800
C	3.28248100	2.48205100	-6.47522800
H	2.57949600	3.32835500	-6.49205400
C	6.15688300	-0.60012300	-3.88363500
H	6.57370500	-0.92159400	-2.91783900
C	6.44769800	-1.29649600	-5.03222500
H	7.09841200	-2.18378300	-4.99263900
C	-5.92116300	-0.78210300	-0.77774500
C	-5.53325000	-2.15748200	-0.94116200
C	-7.28514000	-0.38571800	-0.99668700
C	-6.51194600	-3.09906000	-1.33354600
C	-8.22756600	-1.36576500	-1.38341300
C	-7.84003500	-2.70360900	-1.55296200
H	-6.21546800	-4.15104800	-1.46708200
H	-9.27284300	-1.06466000	-1.55493300
H	-8.58708200	-3.45184600	-1.85933600
C	-2.55238000	1.15069800	3.55378800
C	-2.90052100	2.48135400	3.96851100
C	-3.56661200	0.13716300	3.45721000
C	-4.25492500	2.77999000	4.24054700
C	-4.90808900	0.48629000	3.73355700
C	-5.24476000	1.79413600	4.11223800

H	-4.52487900	3.80150700	4.55117400
H	-5.68997700	-0.28301700	3.63876100
H	-6.29642200	2.04802500	4.31504300
C	-4.15753100	-2.51863900	-0.70952800
H	-3.85197200	-3.56960200	-0.82519700
C	-3.22313300	-1.57397400	-0.36050900
H	-2.17907600	-1.86831000	-0.18550400
C	-6.68608700	1.94250700	-0.48997600
H	-6.96765100	3.00169000	-0.38101700
C	-7.63212300	1.00342500	-0.82848900
H	-8.67969700	1.30273500	-0.98979800
C	-1.87970700	-1.48846800	2.72434300
H	-1.60255200	-2.49499300	2.37776200
C	-3.17853900	-1.19168100	3.06067800
H	-3.95837600	-1.96642500	2.99961600
C	-1.85226400	3.46346800	4.07768500
H	-2.11690300	4.47568600	4.42131500
C	-0.55498000	3.16420100	3.73987300
H	0.22865300	3.93145400	3.81637900
C	-1.44258100	-0.39084200	-5.11092000
H	-1.47608400	0.63496500	-5.50676600
H	-0.45974800	-0.87954000	-5.14520900
H	-2.93495000	-2.88053700	-6.25009300
C	-5.39467000	0.96012300	-4.04759800
H	-5.63898200	1.67171000	-4.87004000
H	-6.27240100	0.93136300	-3.36715800
O	-4.28454400	1.43147100	-3.29214900
H	-3.70823600	1.99390400	-3.86178000
N	-0.91390500	1.85047400	-2.91363600
H	-1.51386900	0.48980700	-3.38601600

Sub

C	-0.47306000	0.32938800	-1.10836200
H	0.41934700	0.86831400	-0.72193700
H	-0.10759100	-0.27363400	-1.96946100
C	-1.49884400	1.35431300	-1.59974000
H	-2.38572300	0.84685700	-2.03657500
H	-1.87448300	1.95338800	-0.74177600
C	-0.93790600	2.30367900	-2.64937500
H	-0.06219900	2.85294100	-2.21993300
H	-0.54320400	1.70755600	-3.51072800
C	0.02716300	-1.04784100	1.02317400
C	-1.01252200	-0.01013500	1.38446200
H	1.07392000	-0.72782600	0.89426900
H	-0.08356600	-2.05375000	1.45992100
H	-1.81165600	-0.30350800	2.08110100
H	-0.68748500	1.03751100	1.49393900
O	-1.96902200	3.19203400	-3.05420300
H	-1.59403800	3.78934600	-3.72645700
C	-0.98701400	-0.60294700	-0.01129700
C	-2.06137100	-1.61045800	-0.47073500
H	-2.78080500	-1.03983100	-1.10013800
C	-1.43652000	-2.70365100	-1.35223300
H	-2.21419700	-3.38030700	-1.76174500
H	-0.87835300	-2.27873600	-2.21049200
H	-0.72548100	-3.32181600	-0.76429400
C	-2.87232600	-2.24189000	0.66706300

H	-3.60171100	-2.97122700	0.26001900
H	-2.22153700	-2.79393000	1.37808900
H	-3.44304700	-1.48669700	1.24234300

3k-(*S*)

C	0.54405200	0.01917700	0.03867300
C	2.00026000	0.14680400	-0.43382100
C	2.35053400	1.55139100	0.05576900
C	0.00203300	1.48309900	0.04413200
H	-0.06224800	-0.65124800	-0.60094800
H	0.51605900	-0.39683600	1.06645800
H	2.06505900	0.10595100	-1.54019500
H	2.66401900	-0.63838500	-0.02048400
H	2.62514800	1.53238600	1.14064800
H	3.18512000	2.03023100	-0.49658500
O	1.17411500	2.31883900	-0.16740800
C	-0.60422800	1.84764300	1.43466100
H	0.20288600	1.60006600	2.16205900
C	-1.83067600	1.00107500	1.79919000
H	-1.63519100	-0.08642700	1.70559400
H	-2.13983800	1.19536600	2.84651600
H	-2.69809100	1.24543900	1.15155600
C	-0.96758700	1.77481100	-1.11687600
H	-1.89013400	1.17786600	-0.95931100
C	-0.39830600	1.50542100	-2.50978900
H	-1.12243300	1.79631300	-3.29709400
H	0.53144700	2.08698600	-2.66711700
H	-0.15823300	0.43309600	-2.66374400
H	-1.27140000	2.84045500	-1.05043200
C	-0.90244600	3.34476200	1.57861900
H	-1.77412800	3.65226100	0.96399900
H	-1.14073500	3.59457100	2.63261500
H	-0.02862000	3.94785600	1.26519000

3k-(*R*)

C	0.57110600	0.05701700	0.03837600
C	2.00733900	0.23692200	-0.46628600
C	2.36258800	1.58965100	0.14731800
C	-0.00381200	1.49898500	0.03378700
H	-0.03019300	-0.63196800	-0.58522300
H	0.57919900	-0.35308800	1.06886200
H	2.03177800	0.29941400	-1.57425000
H	2.69079000	-0.57732000	-0.15445300
H	2.69579300	1.46365700	1.20735200
H	3.16315800	2.13326800	-0.39702400
O	1.16726300	2.36378900	0.08347300
C	-0.74014800	1.80352900	-1.30900700
H	-0.06108900	1.41780600	-2.10324600
C	-0.91429900	3.30753100	-1.55542200
H	0.05158600	3.83598700	-1.44898400
H	-1.30789700	3.49106100	-2.57588800
H	-1.63372000	3.75851600	-0.83992600
C	-0.88793200	1.79757400	1.26329400
H	-1.39653500	2.77206800	1.10454800
C	-0.13276700	1.84581900	2.59168300
H	-0.81475900	2.10410900	3.42639300
H	0.33969400	0.87373500	2.84331000

H	0.66883200	2.60946400	2.55170600
H	-1.69401500	1.03477500	1.30735100
C	-2.08232800	1.07177000	-1.44195100
H	-2.83924600	1.48267500	-0.74250300
H	-2.48695400	1.18920300	-2.46785900
H	-1.99332500	-0.01556200	-1.24223500

References

1. Schwengers, S. A.; De, C. K.; Grossmann, O.; Grimm, J. A. A.; Sadlowski, N. R.; Gerosa, G. G.; List, B., Unified approach to imidodiphosphate-type Brønsted acids with tunable confinement and acidity. *J. Am. Chem. Soc.* **2021,** *143* (36), 14835–14844.
2. Parmar, D.; Matsubara, H.; Price, K.; Spain, M.; Procter, D. J., Lactone Radical Cyclizations and Cyclization Cascades Mediated by SmI_2-H_2O. *J. Am. Chem. Soc.* **2012,** *134* (30), 12751–12757.
3. Konno, T.; Chae, J.; Ishihara, T.; Yamanaka, H., A Facile Regiocontrol in the Palladium-Catalyzed Annulation of Fluorine- Containing Internal Alkynes with Variously Substituted 2-Iodoanilines: A New Regioselective Synthesis of 2- or 3-Fluoroalkylated Indole Derivatives. *J. Org. Chem.* **2004,** *69*, 8258-8265.
4. Neese, F., The ORCA program system. *WIREs. Comput. Mol. Sci.* **2012,** *2* (1), 73-78.
5. Bannwarth, C.; Ehlert, S.; Grimme, S., GFN2-xTB-An Accurate and Broadly Parametrized Self-Consistent Tight-Binding Quantum Chemical Method with Multipole Electrostatics and Density-Dependent Dispersion Contributions. *J. Chem. Theory. Comput.* **2019,** *15* (3), 1652-1671.
6. Pracht, P.; Bohle, F.; Grimme, S., Automated Exploration of the low-energy Chemical Space with Fast Quantum Chemical Methods. *Phys. Chem. Chem. Phys.* **2020,** *22* (14), 7169-7192.
7. Raut, R. K.; Matsutani, S.; Shi, F.; Kataoka, S.; Poje, M.; Mitschke, B.; Maeda, S.; Tsuji, N.; List, B., Catalytic asymmetric fragmentation of cyclopropanes. *Science* **2024,** *386*, 225–230.
8. Perdew, J. P.; Burke, K.; Ernzerhof, M., Generalized gradient approximation made simple. *Phys. Rev. Lett.* **1996,** *77*, 3865–3868.
9. Weigend, F.; Ahlrichs, R., Balanced basis sets of split valence, triple zeta valence and quadruple zeta valence quality for H to Rn: design and assessment of accuracy. *Phys. Chem. Chem. Phys.* **2005,** *7* (18), 3297–3305.
10. Grimme, S.; Antony, J.; Ehrlich, S.; Krieg, H., A consistent and accurate ab initio parametrization of density functional dispersion correction (DFT-D) for the 94 elements H-Pu. *J. Chem. Phys.* **2010,** *132* (15), 154104.
11. Kruse, H.; Grimme, S., A geometrical correction for the inter- and intra-molecular basis set superposition error in Hartree-Fock and density functional theory calculations for large systems. *J. Chem. Phys.* **2012,** *136* (15), 154101.
12. Helmich-Paris, B.; de Souza, B.; Neese, F.; Izsak, R., An improved chain of spheres for exchange algorithm. *J. Chem. Phys.* **2021,** *155* (10), 104109.
13. Wakchaure, V. N.; DeSnoo, W.; Laconsay, C. J.; Leutzsch, M.; Tsuji, N.; Tantillo, D. J.; List, B., Catalytic asymmetric cationic shifts of aliphatic hydrocarbons. *Nature* **2024,** *625* (7994), 287–292.
14. Becke, A. D., Density-functional exchange-energy approximation with correct asymptotic behavior. *Phys. Rev. A* **1988,** *38* (6), 3098–3100.
15. Stephens, P. J.; Devlin, F. J.; Chabalowski, C. F.; Frisch, M. J., Ab initio calculation of vibrational absorption and circular dichroism spectra using density functional force fields. *J. Phys. Chem.* **1994,** *98*, 11623–11627.
16. Becke, A. D., Density-functional thermochemistry. III. The role of exact exchange. *J. Chem. Phys.* **1993,** *98* (7), 5648–5652.
